# Using the Symptom Patient Similarity Network to Explore the Difference between the Chinese and Western Medicine Pathways of Ischemic Stroke and its Comorbidities

**DOI:** 10.1155/2021/4961738

**Published:** 2021-12-01

**Authors:** Lunzhong Zhang, Shu Han, Manli Zhao, Runshun Zhang, Xuebin Zhang, Jing Zhang, Xiaoqing Liu, Yuyao He, Zhao He, Yunfang Dong, Xiaoying Hou, Zijun Mou, Liyun He, Hong Zhou, Jie Yang, Xingyan Huang, Yanjie Hu, Yuefeng Zhang, Lili Zhang, Zhengguang Chen, Xiaozhen Li, Yan Tan, Kegang Cao, Wei Meng, Liqun Zhong

**Affiliations:** ^1^Neurology Department, Weifang Traditional Chinese Hospital, Weifang 261041, China; ^2^Dongzhimen Hospital Affiliated to Beijing University of Traditional Chinese Medicine, Beijing 100700, China; ^3^Guanganmen Hospital, China Academy of Chinese Medical Sciences, Beijing 100053, China; ^4^Institute of Basic Research in Clinical Medicine, China Academy of Chinese Medical Sciences, Beijing 100700, China; ^5^Beijing Zhong Teng Bai Mai Medical Technology Co., Ltd., Beijing, China; ^6^School of Life Sciences, Beijing University of Chinese Medicine, Beijing 100029, China

## Abstract

**Methods:**

Individualized treatment of traditional Chinese medicine (TCM) provides a theoretical basis for the study of the personalized classification of complex diseases. Utilizing the TCM clinical electronic medical records (EMRs) of 7170 in patients with IS, a patient similarity network (PSN) with shared symptoms was constructed. Next, patient subgroups were identified using community detection methods and enrichment analyses were performed. Finally, genetic data of symptoms, herbs, and drugs were used for pathway and GO analysis to explore the characteristics of pathways of subgroups and to compare the similarities and differences in genetic pathways of herbs and drugs from the perspective of molecular pathways of symptoms.

**Results:**

We identified 34 patient modules from the PSN, of which 7 modules include 98.48% of the whole cases. The 7 patient subgroups have their own characteristics of risk factors, complications, and comorbidities and the underlying genetic pathways of symptoms, drugs, and herbs. Each subgroup has the largest number of herb pathways. For specific symptom pathways, the number of herb pathways is more than that of drugs.

**Conclusion:**

The research of disease classification based on community detection of symptom-shared patient networks is practical; the common molecular pathway of symptoms and herbs reflects the rationality of TCM herbs on symptoms and the wide range of therapeutic targets.

## 1. Introduction

Ischemic stroke (IS) is not only a disease with high morbidity, high mortality, and high disability, but also has a great risk of recurrence [1, 2]. An important reason for these characteristics is that patients with IS are often accompanied by a variety of complex diseases, including risk factors, comorbidities, and systemic complications after stroke. The existence of these complex diseases has significantly increased the difficulty and cost of treatment, causing a higher risk of death. Therefore, there is an urgent need for a method to solve the complexity and heterogeneity of IS-related diseases to guide the management of early IS patients.

Thanks to plummeting costs of genetic testing, rapid advances in computational power, massive, linked databases, and new targeted therapies, making it increasingly possible to prevent or treat illnesses based on an individual patient's characteristics, the era of precision medicine is high [3–5]. Recent gene discovery efforts have expanded the number of known single-gene disorders associated with stroke and have linked common variants at approximately 35 genetic loci to stroke risk, which have highlighted novel mechanisms and pathways implicated in stroke related to large artery atherosclerosis, cardioembolism, and small vessel disease and defined shared genetic influences with related vascular traits. [6] In China, traditional Chinese medicine (TCM) has a significant effect on the treatment of acute IS, and its typical individualized medical treatment model also reflects the connotation of precision medicine, but the underlying mechanism has not been fully studied [7]. Therefore, detecting the clinical subtypes of IS in the real-world clinical settings by integrating both TCM and biomedical features would be an interesting research task.

Network medicine, which aims to gain an understanding of human disease from the perspectives of network science, has offered a new platform for identifying novel disease mechanisms and predicting drug efficacy, disease-phenotypic associations, and novel disease taxonomy [8–10]. Symptom-shared patient similarity networks (PSNs) have been established to study many complex diseases, such as hypertension [11] and liver diseases [12]. Li et al. have investigated the correlations between symptoms and TCM herbs and finally found that there are strong positive correlations between symptom similarity and herb similarity [13]. But so far, there is no report about the relationship between TCM herbs and potential molecular pathways in acute IS using symptom-shared PSN. For this purpose, the symptom-shared PSN, which is originated from Shu et al. [12], was used to clarify the characteristics of IS subgroups and compare the molecular enriched pathways between Chinese herbs and Western drugs.

## 2. Materials and Methods

### 2.1. Subjects

#### 2.1.1. Diagnostic Criteria

Diagnosis of “cerebral infarction” is referred to the “Guidelines for Diagnosis and Treatment of Acute Ischemic Stroke in China 2014” [14] formulated by the Cerebrovascular Disease Group of Neurological Branch of Chinese Medical Association in 2014 and the “Guidelines for the Early Management of Patient With Acute Ischemic Stroke 2019” [15] issued by AHA/ASA in 2019.

#### 2.1.2. Inclusion Criteria

Inclusion criteria were defined as follows: (1) the first Western medicine diagnosis is consistent with the “acute cerebral infarction”; (2) MRI report suggests a new responsible focus for ischemia; (3) both initial acute stroke and recurrent acute stroke are included; and (4) EMRs with more than or equal to 5 positive symptoms are obtained.

#### 2.1.3. Exclusion Criteria

Exclusion criteria were defined as follows: (1) the first Western medicine diagnosis included “cerebral hemorrhage”; (2) key content of the medical record is missing, such as chief complaint, symptoms, and history of present illness.

### 2.2. Data Extracting and Preprocessing

EMR data extraction uses “the Clinical Research Information Sharing System of TCM” [16]. Cases of all the inpatients from 2016 to 2018 in Weifang Hospital of traditional Chinese medicine in Shandong Province in China were collected and the diagnosis by “cerebral infarction (ICD-10 code I63)” as a keyword was retrieved. A total of 7170 EMR data were finally screened out, including gender, age, diagnosis, symptoms, herbs, and drugs.

The extracted data of diagnosis, symptoms, drugs, and herbs were standardized. According to ICD-10, the disease diagnosis level is standardized to subcategories. After manual review by physicians with rich clinical experience in TCM, referring to the Unified Medical Language System (UMLS) [17], 362 standardized symptoms were finally obtained.

Referred to the Anatomical Therapeutic Chemical (ATC) developed by the WHO Collaborating Centre for Drug Statistics Methodology and “Catalogue of Chinese Listed Chemical Drugs” published by the China Food and Drug Administration (CFDA), 459 generic names were unified, and then, their genetic target information was obtained using the DrugBank database [18–22]. 351 names of TCM herbs were standardized using Chinese Pharmacopoeia 2015 (CHPH, 2015 Edition) and using TCMSP [23] to obtain the information of corresponding genetic targets.

To better study the characteristics of the disease, the diagnosis has been divided into three categories based on the following principles: risk factors, including various metabolic syndrome and coronary heart disease, are the potential causes of ischemic stroke; the remaining diseases that have no exact causal relationship with ischemic stroke are called comorbidity [24], while complications refer to the diseases involving multiple systems that appear based on ischemic stroke and have an indirect relationship with it [24]. Furthermore, risk factors were divided into eight categories according to the related systems: (1) hypertension; (2) diabetes/impaired glucose tolerance; (3) hyperlipidemia; (4) hyperuricemia/gout; (5) heart disease including rheumatic heart disease, coronary heart disease, dilated cardiomyopathy, and all other heart diseases; (6) arteriosclerosis, arterial occlusion and stenosis, and arteriovenous malformations called as vascular diseases; (7) polycythemia vera, thrombocytosis, thrombocytopenia, and coagulation defects classified as blood diseases; and (8) fatty acid metabolism disorder, amyloidosis, glycoprotein metabolism disorder, and sulfur-loaded amino acid metabolism disorder (hyperhomocysteinemia) collectively referred to as other metabolic diseases.

### 2.3. Integration of Symptom-Gene Data

With 362 normalized symptom terms as keywords, part of symptom-gene information was obtained in the SymMap database [25]. Symptoms whose genetic information was not found were searched through the MalaCards database [26] to obtain the corresponding disease information, and then, the genetic information was obtained with disease as the keyword, so as to integrate the corresponding relationship between symptom and gene data.

### 2.4. Construction of Patient Similarity Network

The Jaccard coefficient [27] was used to calculate the similarities of patients based on their symptom phenotypes and constructed the patient similar network, which is defined as follows: Jaccard′sim(*A*, *B*)=*P*(*A*∩*B*)/*P*(*A* ∪ *B*), and this means that if two patients have more intersected symptom phenotypes, they would have higher symptom similarity. In particular, the symptom similarity of two patients would be 1 if they have identical symptom phenotypes. Supposing that patient A has 5 symptoms, patient B has 8 symptoms, and 2 of them are common, then the Jaccard′sim(*A*, *B*)=(*P*(*A*∩*B*)/*P*(*A* ∪ *B*))=(*P*(*A*∩*B*)/*P*(*A*)+*P*(*B*) − *P*(*A*∩*B*))=(2/5+8 − 2)=(2/11) Then, the edges were filtered by weight ≥0.5, which means that only the patient links with almost identical chief symptoms were kept while remaining most of the cases included at the same time. The Jaccard coefficient is a commonly used similarity measure, especially in network medicine research [28–31].

### 2.5. Community Detection Method to Identify the Patient Subgroups

Patients should be divided into some subgroups with more clear boundaries; thus, a nonoverlapping community detection method is more applicable for PSN [12]. An efficient community detection algorithm, namely, BGLL [32], was applied to obtain the patient subgroups from the whole networks. BGLL is an iterative algorithm based on modularity measurement, by continuous division to get the maximum gain of modularity, combines local optimization and multilevel clustering, and is extremely fast, which has linear complexity on typical and sparse data. Modularity proposed by Newman [33, 34] was designed to measure the strength of division of a network into subgroups. Networks with high modularity (ranges from 0 to 1 and usually appears between 0.3 and 0.7 in actual) have the better effect of community division. In this study, all the results were obtained by setting resolution limit = 1.0 and randomize value on.

### 2.6. Phenotype Enrichment Analysis

Relative risk (RR) is a classical statistical method, which is the ratio of the probability of an event occurring, in an exposed group that a certain condition is present versus a nonexposed group that lacks the condition [35]. In this study, RR was used to evaluate the specificity of diseases in the patient subgroups. The patient in a specific module was treated as an exposed group, and the left patient cases were regarded as the nonexposed group and a disease as an event. So, RR is defined as follows:

(1) $$\begin{array}{c} \text{RR}=\frac{\left(C_{ij}/C_{i}\right)}{\left(\left(C_{j}-C_{ij}\right)/\left(N-C_{i}\right)\right)}, \end{array}$$

where *C*_*i*_ is the number of patients in module (patient subgroup) *i*, *C*_*j*_ is the number of patients with disease *j*, *C*_*ij*_ represents the number of patients in module *i* and with disease *j*, and *N* is the total number of patients in the study. If RR > 1.5, it indicates that the distribution of disease *j* in module *i* is higher than the distribution in the whole. A similar method is used to identify the significant symptoms, drugs, and herbs in each patient subgroup. In addition, the true significant disease symptom, drug, or herb is filtered by the chi-square test (with *P* value <0.05), which is a statistical hypothesis test whose result is evaluated by reference to the chi-square distribution [36].

### 2.7. Pathway Enrichment Analysis

Pathway analysis of the gene data of symptoms, drugs, and herbs has been carried out. The KEGG pathway database is the main database in the Kyoto Encyclopedia of Genes and Genomes (KEGG), and it consists of manually drawn reference pathway maps together with organism-specific pathway maps [37]. The enriched KEGG pathways were obtained using the database for annotation, visualization, and integrated discovery (DAVID), which is a Web-based online bioinformatics resource that aims to provide tools for the functional interpretation of large lists of genes/proteins. Enriched medicines (drugs or herbs) were selected with *P* values less than 0.05 as the specific medicines of the module, and then the medicines target as the genes of the module, and the significant pathway through Fisher's exact test [38] was finally found. The Jaccard coefficient [27] was used to calculate the similarity between the pathways of drugs and herbs.

## 3. Results

### 3.1. Basic Statistical Characteristics of the 7170 Ischemic Stroke Disease Inpatient Cases

**Figure 1:**
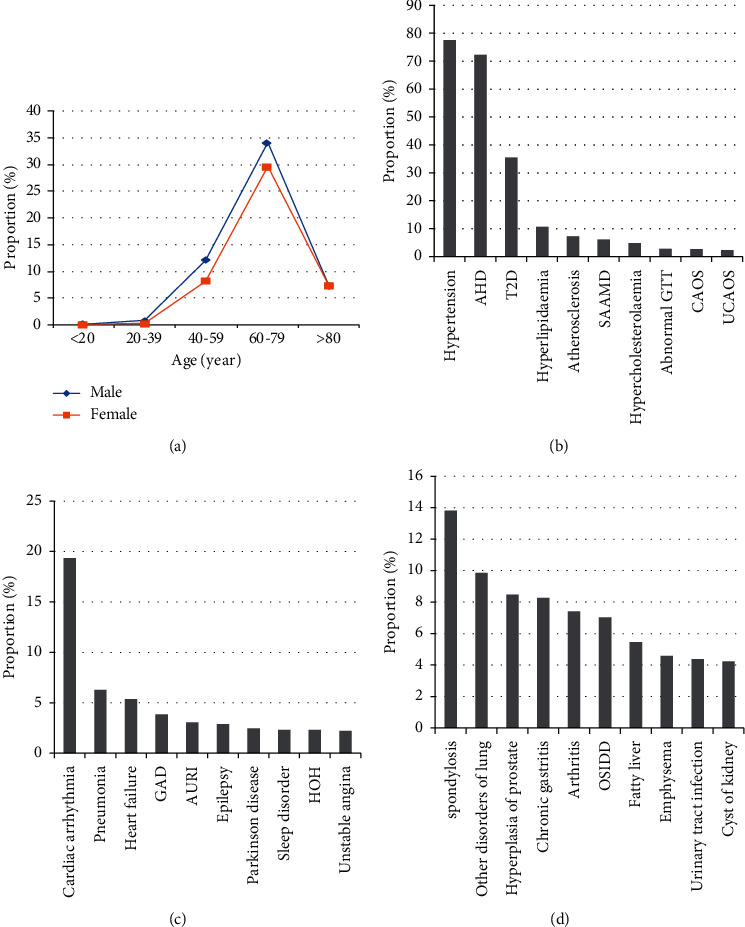
Basic clinical characteristics of 7170 patient cases. (a) The proportion of patients with age and gender in hospitalization. (b) The proportion of patients in the top 10 risk factors. AHD: atherosclerotic heart disease; T2D: type 2 diabetes mellitus; Atherosclerosis: atherosclerosis of other arteries; SAAMD: disorders of sulfur-bearing amino acid metabolism; Hypercholesterolemia: pure hypercholesterolemia; Abnormal GTT: abnormal glucose tolerance test; CAOS: occlusion and stenosis of carotid artery; UCAOS: occlusion and stenosis of unspecified cerebral artery. (c) The proportion of patients in the top 10 complications. GAD: generalized anxiety disorder; AURI: acute upper respiratory infection; HOH: hypoosmolality and hyponatremia. (d) The proportion of patients in the top 10 comorbidity diseases. The proportion in (b–d) means the percentage of patients with this disease in the total population.

In this study, 7170 patients (3913 males and 3257 females) with IS were included, of which 54.22% were males and 45.78% were females. The proportion of patients with ischemic stroke in different age groups is different. It gradually increases with age to 40 years and decreases after 60 years. The proportion of the 60–79 years of age group was the highest, and it was irrelevant to gender (Figure 1(a)).

Analysis of the risk factors for IS shows that the incidence rates of hypertension, arteriosclerotic heart disease (AHD), type 2 diabetes (T2D), and hyperlipidemia are all greater than 10%, among which the incidence rate of hypertension was up to 77.56%; AHD, as the main risk factor for long-term recurrence of IS [39], has the second highest incidence rate (72.30%); and T2D ranked third with an incidence rate of 35.38% (Figure 1(b)).

In IS complications, incidence rate of cardiac arrhythmia (including bradycardia, atrial fibrillation and flutter, ventricular premature beat, atrial premature beat, tachycardia) is 19.35% higher than others; incidence rates of pneumonia and heart failure are greater than 5% (Figure 1(c)). Among comorbidity diseases, spondylosis (ICD-10 code M47.9) is the most common, with an incidence rate of 13.82% ranking first. The incidence rate of other disorders of lung, hyperplasia of prostate, chronic gastritis, arthritis, other specified intervertebral disc displacement (OSIDD), and fatty liver rank second to seventh, all higher than 5% (Figure 1(d)).

### 3.2. Patient Similarity Network of Ischemic Stroke

**Figure 2:**
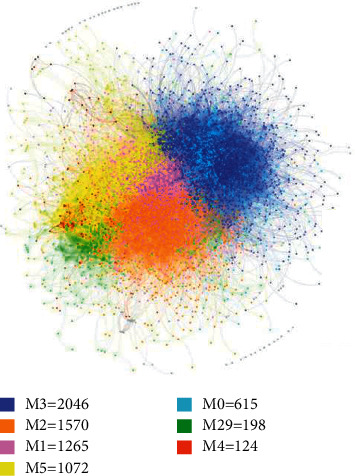
Patient similarity network. Different colors refer to different modules (modules with less than 100 patients are indicated in grey).

To identify the subtype of patients with ischemic stroke, by calculating the degree of the shared symptom phenotypes between patient pairs, a PSN with 6996 nodes (patients, and 174 of 7170 patients with low similarity have been eliminated) and 397775 edges was constructed. On this basis, a high-performance community detection method (see Materials and Methods) was used to explore the subgroup of ischemic stroke disease population. Finally, 34 modules (modularity: 0.478) were obtained, in which the numbers of patients for each module ranged from 2 to 2046. These modules represent the subgroup of stroke patients, among which 7 modules (such as M3 and M2) have 124 or more cases, accounting for 98.48% of all cases, and 27 modules have 20 or fewer cases, accounting for 1.52%. Considering a more comprehensive study of the diversity of ischemic stroke subgroups, we used four large modules (M3, M2, M1, and M5) and three small modules (M0, M29, and M4), which accounted for 85.09% and 13.39% of all cases, respectively, to illustrate the characteristic phenotype and genotype of ischemic stroke subgroups (Figure 2). The cases of the 7 subgroups were as follows: M3 (*n* *=* 2046, 29.25%), M2 (*n* *=* 1570, 22.44%), M1 (*n* *=* 1265, 18.08%), M5 (*n* *=* 1072, 15.32%), M0 (*n* *=* 615, 8.79%), M29 (*n* *=* 198, 2.83%), and M4 (*n* *=* 124, 1.77%).

### 3.3. Significant Disease of the Ischemic Stroke Subgroups

**Figure 3:**
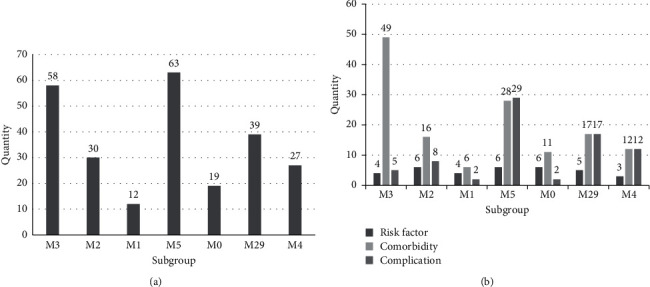
Quantitative statistics of diseases. (a) Total number of selected diseases in each subgroup. (b) Differential count of selected diseases in each subgroup.

The characteristic diseases in 7 subgroups (*P* < 0.05, *RR* > 1.5, Figure 3(a), Supplementary Materials—Table S1) were divided into three categories according to risk factors, complications, and comorbidities, as shown in Figure 3(b). In Figure 3, it shows that M5 has the most disease among the seven subgroups, with 63 diseases in total, and M1 has the least, with only 12 diseases. The proportion of risk factors in each subgroup is below 50%, among which M1 and M0 are more than 30% (33.33% and 31.58%, respectively), and the rest subgroups are less than 20%; the largest proportion of comorbidities is M3, which is as high as 84.48%, and it is more than 50% in M2, M1, and M0 (53.33%, 50%, and 57.89%, respectively), while the other three subgroups are all under 50%; the complications accounted for less than 50% in each subgroup as well, of which M5, M29, and M4 accounted for more than 40% (46.03%, 43.59%, and 44.44%, respectively), and the remaining subgroups are less than 30%. In addition, the number of complications and comorbidities in M5, M29, and M4 is relatively average.

As a whole, the disease characteristics of each module are analyzed in more detail. It can be found that the types of diseases in M3, M2, M5, M29, and M4 are very complex, involving more than 10 systems. Among them, patients in M2, M5, M29, and M4 are more susceptible to neurological diseases, and patients in M3 are more likely to suffer from digestive system diseases, while patients in M1 and M0 have a small number of selected diseases, not many systems are involved, and their characteristics are not obvious.

The results of 8 categories of risk factors are shown in Table 1. It can be seen that M3 patients were more likely to be associated with impaired glucose tolerance, hyperlipidemia, vascular factors, and other metabolic diseases; patients in M2 were more likely to be associated with hypertension, diabetes, vascular factors, and blood diseases; M1 patients were more likely to have gout, vascular factors, blood diseases, and other metabolic diseases; M5 patients were more prone to be associated with hyperlipidemia, heart disease, blood diseases, and other metabolic diseases; M0 is more likely to be related to hyperlipidemia, heart disease, vascular factors, and other metabolic diseases; M29 is more likely to be related to heart disease, vascular factors, and other metabolic diseases; and M4 is more likely to be related to hyperuricemia, heart disease, and vascular factors.

**Figure 4:**
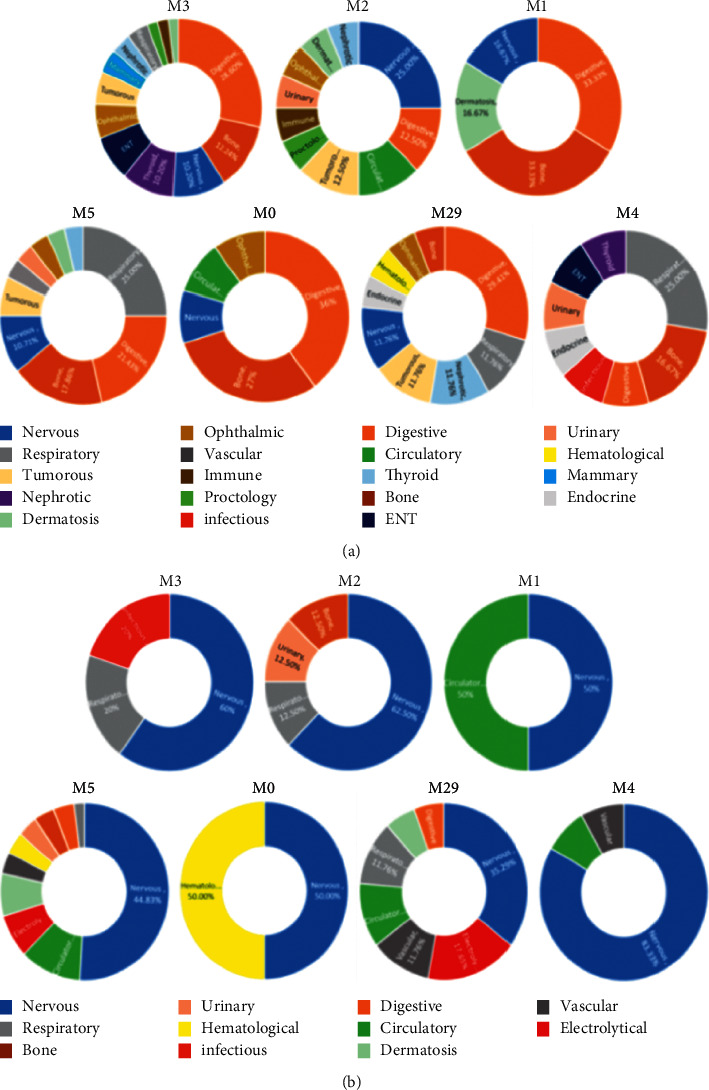
Characteristics of comorbidities and complications in each subgroup. (a) System distribution of comorbidities in each subgroup. (b) System distribution of complications in each subgroup.

The results of the characteristic comorbidities of each subgroup can be seen in Figure 4(a). It shows that patients in M2 are more likely to have nervous system diseases; patients in M3, M0, and M29 are more likely to have digestive system diseases; patients in M5 and M4 are more likely to have respiratory system diseases; and patients in M1 are more likely to have the digestive system and orthopedic diseases. In addition, compared with other subgroups, patients in M3 are more likely to have thyroid and otorhinolaryngologic diseases, and patients are more likely to have tumors in M29.

The results of complications analysis are shown in Figure 4(b). Patients in 5 subgroups except M1 and M0 were more likely to have nervous system diseases. Compared with other subgroups, M3 is most prone to syncope; M2 is most prone to skin sensory abnormality; M5 is most prone to epilepsy prone; M0 is most prone to autonomic nervous system disease; M29 is most prone to hydrocephalus; M4 is most prone to quadriplegia; M1 is most likely to be complicated with arrhythmia and mental disorder; and M0 is most likely to be complicated with nutritional anemia. In addition, bedsore and pneumonia of M29 were also more significant than that of other subgroups.

### 3.4. Characteristics of Pathways Specific to the Subgroups

After data preprocessing of the 7170 clinical cases, 362 symptoms, 459 drugs, and 351 herbs with their standard terminologies have been obtained. In enrichment analysis, the specificity of symptoms, drugs, and herbs of 7 subgroups was evaluated by calculating RR (Supplementary Materials—Table S2). Taking the largest module as an example, the top 10 significant symptoms, herbs, and drugs enriched in M3 are listed in Table 2.

Then, genetic pathways of symptom, drug, and herb specific to each of the 7 subgroups were identified by pathway analysis (Supplementary Materials—Table S3). By statistical analysis of the number of pathways, it can be found that the number of drug pathways is significantly smaller than that of symptom or herb pathways in the 7 subgroups (*P* < 0.01). The number of symptom pathways in M2 (*n* *=* 47) and M4 (*n* *=* 59) is less than the average (*n* *=* 94); the number of drug pathways in M1 (*n* *=* 28), M0 (*n* *=* 8), and M4 (*n* *=* 31) is relatively small, which is less than the average of 34, while the number of herb pathways is large than that of symptom and drug pathways, which is not significantly different in each subgroup (coefficient of variation, CV≈6.18) (Table 3).

**Figure 5:**
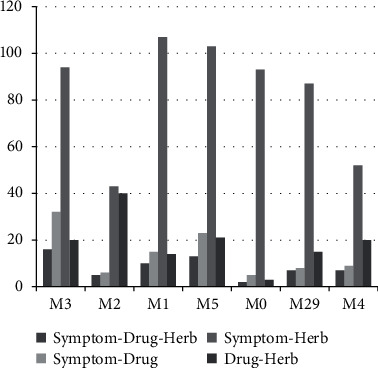
Number of symptom-drug-herb common pathway of each subgroup (*P* < 0.01).

Comparisons had been made between the pathways of symptom, drug, and herb, one and any of the others. It can be found that the number of symptom-herb pathways shared the most pathways in all subgroups (Figure 5, Table S3). Most of the pathway functions focus on cancer: pathways in cancer, microRNAs in cancer; infectious diseases such as hepatitis B and toxoplasmosis; and signal transduction such as c-AMP signaling pathway and PI3K-Akt signaling pathway. Subgroup M3 was selected with the largest number of common symptom-drug-herb pathways (*n* *=* 16), and the top 20 enriched pathways of symptom genes and the corresponding *P* values of drug and herb genetic pathways are shown in Table 4. It can be seen that, for the same symptom pathway, the number of herb pathways with statistical significance is significantly more than that of drug pathway.

## 4. Discussion

Through the reliable symptom-shared PSN, we found significant quantitative differences between TCM herbs and Western medicine-enriched genetic pathways in acute IS subgroups.

All the data were collected from EMRs of “the Clinical Research Information Sharing System of TCM.” From the overall data, the basic characteristics are consistent with the general IS population. For example, the distribution of age and sex is consistent with the report of China Stroke Statistics 2019 [40]. From the perspective of the 7 subgroups, the risk factors, comorbidities, and complications showed distinct characteristics, which are in accord with the heterogeneity of complex diseases. Patients in M29 were more likely to be associated with heart disease and vascular factors, together with bedsore and pneumonia, consistent with the report [40], while their comorbidities were prone to be digestive system diseases and tumors. The top 5 pathways are all about cancer, particularly lung cancer (microRNAs in cancer, pathways in cancer, non-small-cell lung cancer, small-cell lung cancer, proteoglycans in cancer, in Table S3). It obviously reflected the correlation between clinical manifestations and molecular mechanisms of the diseases [41], and the inherent consistent features showed the symptom-shared PSN is practical.

The most interesting subgroup is M3, conforming to the clinical practice in Western medicine and highly consistent with TCM theory as well. Through the enrichment analysis, the main symptoms in M3 are dizzy, headache, vertigo, tinnitus, etc., which are mainly differentiated as “liver Yang hyperactivity syndrome” (LYHS) in the theory of TCM [42]. And the prescription is Tian-Ma-Gou-Teng-Yin, composed of Tianma, Shijueming, Gouteng, Shouwuteng, Zhizi, Huangqin, Hujisheng, Duzhong, etc., which are in accord with most of the herbs detected in M3. The above consistent results are in perfect agreement with the “symptom-syndrome-prescription correspondence” principle in TCM theory [43]. Enriched drugs such as hydrochloride, betahistine, promethazine, and flunarizine are also the correct drug to treat the symptoms above. The symptom is a vital basis for clinical diagnosis and treatment as well as disease classification [41]. Meanwhile, TCM syndromes and their corresponding prescriptions heavily depended on symptoms [44, 45], which also bring us the enlightenment of classifying complex diseases according to the symptom phenotype. Considering the consistency between symptoms and medicine in M3, it proves again that the detected subgroups based on symptoms are in line with clinical practice.

On the other hand, we can infer the potential molecular pathway of LYHS from the result of pathways analysis. Using a systems biology approach with the combination of computational analysis and animal experiment, Li et al. [46] have found that “Cold ZHENG” and “Hot ZHENG” have their own corresponding genetic pathways. Zhai et al. [47] found that 46 genes were involved in the Qi deficiency and blood stasis syndrome of both stroke and coronary heart disease (CHD) by means of data mining, which also supported the existence of biological basis under TCM syndrome (“ZHENG” in Chinese). In our study, as mentioned before, the symptoms in M3 have corresponding syndrome LYHS and prescriptions in the theory of TCM. Therefore, it can be inferred that the potential molecular pathways of LYHS are possibly related to the enriched symptom pathways in M3 (Table 4). It is expected to predict more potential pathways of other acute IS syndromes through network methods in the future, strengthen the understanding of the underlying mechanism of TCM treatment of stroke, and provide ideas for TCM syndrome research.

Moreover, through pathway enrichment analysis, we distinguished the feature of genetic pathway of TCM herbs in 7 subgroups (Supplementary Materials—Table S3). In each group, the quantity of herb pathways was more than that of symptom and drug pathways (Table 3, *CV*≈6.18). The number of symptom-herb common pathways was larger than that of symptom-drug-shared pathways in all subgroups; in the same subgroup (such as M3), to the same symptom pathway (*P* < 0.01), the number of statistically significant pathways of herbs is obviously more than that of drugs. This may be due to the fact that Chinese herb is different from Western medicine with a single chemical composition, and the complex composition leads to a significantly richer number of herb pathways. It indicates that Chinese herbs play a more comprehensive role in the treatment of IS by acting on multiple targets. The key nodes of disease-herb-target network can be mined through network pharmacology methods to explain the mechanism of multicomponent and multitarget of herbs and provide ideas for solving the problems such as unclear active ingredients and mechanism of action of herbs [48]. Network pharmacology methods have been used by some scholars [49] to investigate the underlying molecular mechanisms of Lian Xia Ning Xin formula, a Chinese prescription, to treat CHD and disease phenotypes. In the future, on the basis of determining specific Chinese medicines or prescriptions, it may be possible to predict the TCM herb targets for the treatment of stroke.

There are several potential limitations in our research. First of all, the patients in this study were all from one hospital, which might induce sample bias. Actually, “the Clinical Research Information Sharing System of TCM” used in the study has been deployed in 60 Chinese medicine hospitals across China and has been used in studying the mechanism of TCM diagnosis or treatment of many diseases, such as CHD [45, 50], AECOPD [51, 52], and COVID-19 [53]. With appropriate designing strategies, we could integrate these data from the multiple resources to perform a large-scale retrospective cohort study with more reliable results. Secondly, due to the limitations of retrospective research, genetic testing could not be performed to clinically verify the results. Meanwhile, personalized medicine will increasingly rely on EMRs to store vast amounts of genomic data [54]. Supported by the sharing system, we will have the opportunity to carry out multicenter research to obtain more universal conclusions.

## 5. Conclusions

To our knowledge, this is the first study to demonstrate the utility of symptom-shared PSN for the classification of ischemic stroke and it can be used for other complex diseases as well. The distinct diseases of each subgroup and the underlying biological pathways of its diseases indicate that it is possible to further explore the molecular network mechanisms of complex diseases using this clinical disease subtyping approach. Meanwhile, the inherent consistency on symptom and TCM herb from both clinical and molecular features reflects the rationality of TCM on symptoms and the wide range of therapeutic targets.

## Figures and Tables

**Table 1:** Risk factors for each subgroup.

Risk factors	M3	M2	M1	M5	M0	M29	M4
Hypertension		√					
Diabetes/impaired glucose tolerance	√	√					
Hyperlipidemia	√			√	√		
Hyperuricemia/gout			√				√
Heart disease				√	√	√	√
Vascular factors	√	√	√		√	√	√
Blood diseases		√	√	√			
Other metabolic diseases	√		√	√	√	√	

**Table 2:** Top 10 significant symptoms, drugs, and herbs in M3 (*P* < 0.01, RR > 1.5).

		*P* value	RR
Symptoms	Dizzy	0	2.211773
	Sleep disorder insomnia	2.8E-154	2.213527
	Headache	1.4E-146	4.808182
	Queasy	8.3E-114	3.840246
	Vertigo	3.62E-98	7.746588
	Impaired sensations	9.88E-92	6.330342
	Stiffness muscle	1.36E-87	3.562796
	Palpitation	5.51E-57	1.758678
	Tinnitus	1.34E-39	3.047759
	Dream	4.04E-26	3.584229

Herbs	Tianma	2.44E-81	1.735151
	Shijueming	1.78E-75	3.024194
	Gouteng	1.3E-67	2.307379
	Shouwuteng	4.39E-50	2.812062
	Zhizi	1.81E-49	2.345902
	Gegen	2.39E-48	2.461614
	Huangqin	1E-43	1.749792
	Suanzaoren	1.53E-43	2.478363
	Hujisheng	1.59E-36	2.304421
	Duzhong	2.24E-31	2.475248

Drugs	Betahistine hydrochloride	1.63E-78	3.213303
	Gastrodin injection	2.72E-46	1.852636
	Betahistine mesylate	9.66E-28	2.628823
	Flupentixol	9.6E-22	2.049731
	Promethazine hydrochloride	2.87E-21	2.658477
	Flunarizine hydrochloride	1.72E-20	3.739003
	Ginseng glucose injection	6.07E-11	1.786732
	Vinpocetine	5.06E-09	1.808967
	Levothyroxine sodium	7.79E-09	3.400174
	Homogenin hydrobromide	1.77E-08	2.520161

**Table 3:** Statistics of symptom, drug, and herb pathways of each subgroup.

Subgroup	Symptom pathway	Drug pathway	Herb pathway	Drug-herb-shared pathway	Drug-herb Jaccard coefficient
M3	115	38	146	20	0.12
M2	47	55	158	40	0.23
M1	117	28	145	14	0.09
M5	118	41	146	21	0.13
M0	100	8	158	3	0.02
M29	102	38	129	15	0.10
M4	59	31	145	20	0.13
*Means*	94	34.14	146.71	19	—
*SD*	26.94	13.32	9.07	—	—
*CV*	28.66	39.03	6.18	—	—

Note: symptom pathway, drug pathway, and herb pathway are, respectively, the number of pathways with *P* value less than 0.01 for symptom, drug, and herb; the drug-herb-shared pathway is the number of the same pathways with *P* value less than 0.01 for drug and herb pathway; drug-herb Jaccard coefficient reflects the similarity of drug and herb pathways in the same subgroup; SD: standard deviation; CV: coefficient of variation.

**Table 4:** Top 20 enriched pathways of symptom genes in M3.

Symptom pathway in M3	Symptom pathway *P* value	Drug pathway *P* value	Herb pathway *P* value
AGE-RAGE SP in diabetic complications	3.3876E-17	ns	2.00999E-29
Neuroactive ligand-receptor interaction	1.32395E-16	9.33419E-48	ns
Alzheimer's disease	3.67245E-16	5.1187E-06	5.56899E-05
Calcium signaling pathway	5.41417E-16	7.04619E-24	ns
Amyotrophic lateral sclerosis (ALS)	3.09577E-15	0.000130295	3.13695E-10
Pathways in cancer	4.09088E-15	ns	1.4964E-32
Hepatitis B	3.44822E-14	ns	1.61268E-31
Bladder cancer	2.22937E-13	ns	7.08838E-17
Proteoglycans in cancer	3.97422E-13	ns	3.49664E-17
Malaria	4.0587E-13	ns	3.28241E-16
Pancreatic cancer	8.48407E-13	ns	4.62234E-21
cAMP signaling pathway	1.77595E-12	4.10807E-14	7.44568E-05
Colorectal cancer	1.48163E-11	ns	5.75226E-16
HIF-1 signaling pathway	2.86538E-11	ns	4.7202E-19
EGFR tyrosine kinase inhibitor resistance	3.02218E-11	ns	3.82288E-17
Endocrine resistance	3.49548E-11	ns	3.29809E-23
MicroRNAs in cancer	9.68694E-11	ns	9.46322E-11
Prostate cancer	3.02359E-10	ns	1.5772E-23
FoxO signaling pathway	3.21659E-10	ns	5.3789E-25
Glioma	4.70998E-10	ns	2.33281E-14

AGE-RAGE SP in diabetic complications: AGE-RAGE signaling pathway in diabetic complications; ns: not significant.

## Data Availability

The datasets used during the current study are available from the corresponding author on reasonable request.

## Supplementary Materials

Supplementary Materials Table S1: enriched diseases of each subgroup. Table S2: enriched symptoms, herbs, and drugs of each subgroup. Table S3: pathways of each subgroup.
