# Supplementary material for: Using the Symptom Patient Similarity Network to Explore the Difference between the Chinese and Western Medicine Pathways of Ischemic Stroke and its Comorbidities
Source: Evid Based Complement Alternat Med. 2021 Dec 1;2021:4961738. doi: 10.1155/2021/4961738 (PMC8654542; doi:10.1155/2021/4961738)
Supplement: Supplementary Materials — Table S1: enriched diseases of each subgroup. Table S2: enriched symptoms, herbs, and drugs of each subgroup. Table S3: pathways of each subgroup. [file 4961738.f1.zip › 4961738.f1/Table S1 Enriched diseases of each subgroup.docx]

**Table S1 Enriched diseases of each subgroup *(P*<0.05, *RR*>1.5)**

| M3 | *P_*value | *RR* | Overlap | Classification according to risk factors comorbidities or complications | Classification according to systems |
| --- | --- | --- | --- | --- | --- |
| Chronic diseases of tonsils and adenoids | 0.003267007 | 14.51612903 | 6 | Comorbidity | ENT |
| Esophagitis | 0.003267007 | 14.51612903 | 6 | Comorbidity | Digestive |
| Single neuropathy, other specific | 0.009679594 | 12.09677419 | 5 | Comorbidity | Nervous |
| Disorder of fatty acid metabolism | 0.009679594 | 12.09677419 | 5 | Risk factor | Metabolic |
| Fibrocystic breast disease of the breast | 0.000463064 | 10.88709677 | 9 | Comorbidity | Mammary |
| Breast lump | 0.00131054 | 9.677419355 | 8 | Comorbidity | Mammary |
| Sicca syndrome | 0.027972111 | 9.677419355 | 4 | Comorbidity | Immune |
| Chronic conjunctivitis | 0.027972111 | 9.677419355 | 4 | Comorbidity | Ophthalmic |
| Syncope and collapse | 0.027972111 | 9.677419355 | 4 | Complication | Nervous |
| Renal neoplasms with uncertain or unknown dynamics | 0.027972111 | 9.677419355 | 4 | Comorbidity | Tumorous |
| Renal failure | 0.027972111 | 9.677419355 | 4 | Comorbidity | Nephrotic |
| Background retinopathy and retinal vascular changes | 0.027972111 | 9.677419355 | 4 | Comorbidity | Ophthalmic |
| Autoimmune hepatitis | 0.027972111 | 9.677419355 | 4 | Comorbidity | Digestive |
| Autoimmune thyroiditis | 2.81159E-07 | 6.955645161 | 23 | Comorbidity | Thyroid |
| Tension headache | 1.81673E-06 | 6.912442396 | 20 | Comorbidity | Digestive |
| Tinnitus | 0.000498662 | 5.806451613 | 12 | Comorbidity | ENT |
| Other specific diseases of the anus and rectum | 0.009053507 | 5.64516129 | 7 | Comorbidity | Proctology |
| Peptic ulcer without bleeding | 0.022197195 | 4.838709677 | 6 | Comorbidity | Digestive |
| Adrenal tumors with uncertain or unknown dynamics | 0.000965347 | 4.493087558 | 13 | Comorbidity | Tumorous |
| Benign tumor of uterus | 7.5327E-07 | 4.166666667 | 31 | Comorbidity | Tumorous |
| Vertebro-basilar artery syndrome | 9.298E-278 | 3.928523625 | 1174 | Risk factor | Nervous |
| Conjunctival hemorrhage | 0.041912727 | 3.629032258 | 6 | Comorbidity | Ophthalmic |
| Urticaria | 0.041912727 | 3.629032258 | 6 | Comorbidity | Dermatosis |
| Migraine | 0.000751466 | 3.349875931 | 18 | Comorbidity | Nervous |
| Prosopalgia | 0.006923563 | 3.225806452 | 12 | Comorbidity | Nervous |
| Acute hepatitis B, without δ factor (co-infection), and without hepatic coma | 0.021605749 | 3.110599078 | 9 | Comorbidity | Digestive |
| Spondylosis | 2.32559E-79 | 3.036593431 | 551 | Comorbidity | Bone |
| Cervical disc displacement,others | 4.73745E-20 | 2.9296875 | 155 | Comorbidity | Bone |
| Generalized anxiety disorder | 6.97077E-18 | 2.832415421 | 144 | Complication | Nervous |
| Somnipathy | 7.57134E-11 | 2.729528536 | 88 | Complication | Nervous |
| Chronic pharyngitis | 0.000209132 | 2.698511166 | 29 | Comorbidity | ENT |
| Subclinical hypothyroidism | 2.0108E-05 | 2.419354839 | 46 | Comorbidity | Thyroid |
| Spondylodynia | 0.000824737 | 2.419354839 | 28 | Comorbidity | Bone |
| Gastroesophageal reflux disease with esophagitis | 0.002911265 | 2.419354839 | 22 | Comorbidity | Digestive |
| Gastroenteritis and colitis | 0.025609003 | 2.419354839 | 12 | Comorbidity | Digestive |
| Carpal tunnel syndrome | 0.040435637 | 2.419354839 | 10 | Comorbidity | Nervous |
| Chronic superficial gastritis | 0.000223594 | 2.35396687 | 36 | Comorbidity | Digestive |
| Gastritis,others | 0.003354994 | 2.318548387 | 23 | Comorbidity | Digestive |
| Gastroesophageal reflux disease | 5.64262E-05 | 2.271231073 | 46 | Comorbidity | Digestive |
| Perceptive nervous deafness | 0.010938803 | 2.188940092 | 19 | Comorbidity | ENT |
| Bacterial infection,others | 0.032224564 | 2.116935484 | 14 | Complication | Infectious |
| Hypothyroidism | 0.001186992 | 2.034457478 | 37 | Comorbidity | Thyroid |
| Spondylosis,others | 0.034954311 | 2.016129032 | 15 | Comorbidity | Bone |
| Nontoxic single thyroid nodule | 1.88992E-08 | 1.967741935 | 122 | Comorbidity | Thyroid |
| Gastritis | 0.000706357 | 1.88172043 | 49 | Comorbidity | Digestive |
| Acute or chronic gastric ulcers without bleeding | 0.01938547 | 1.85483871 | 23 | Comorbidity | Digestive |
| Acute bronchitis | 0.009735902 | 1.846349745 | 29 | Complication | Repiratory |
| Polyneuropathy | 0.000709378 | 1.814516129 | 54 | Comorbidity | Nervous |
| Hyperthyroidism | 0.035117663 | 1.792114695 | 20 | Comorbidity | Thyroid |
| Renal and perirenal abscesses | 0.044116726 | 1.728110599 | 20 | Comorbidity | Nephrotic |
| Liver diseases, other specific | 4.23943E-05 | 1.693548387 | 98 | Comorbidity | Digestive |
| Chronic gastritis | 2.99389E-10 | 1.661152468 | 241 | Comorbidity | Digestive |
| Pulmonary sarcoidosis | 0.001436483 | 1.559584111 | 78 | Comorbidity | Repiratory |
| Hyperlipemia | 3.80422E-10 | 1.553809789 | 298 | Risk factor | Metabolic |
| Disc displacement, other specified | 8.92672E-07 | 1.541745731 | 195 | Comorbidity | Bone |
| Abdominal, lower back and pelvic injuries | 0.000333461 | 1.535638401 | 106 | Comorbidity | Bone |
| Asthma | 0.042173077 | 1.518026565 | 32 | Comorbidity | Repiratory |
| Abnormal glucose tolerance | 0.003160795 | 1.512096774 | 75 | Risk factor | Metabolic |

| M2 | *P_*value | *RR* | Overlap | Classification according to risk factors comorbidities or complications | Classification according to systems |
| --- | --- | --- | --- | --- | --- |
| Tumours with undetermined or unknown dynamics,other special parts | 0.010378412 | 13.82420382 | 4 | Comorbidity | Tumorous |
| Paraesthesia of skin | 0.010378412 | 13.82420382 | 4 | Complication | Nervous |
| Polycythemia vera | 0.010378412 | 13.82420382 | 4 | Risk factor | Hematological |
| Senile cataract | 4.59436E-05 | 11.52016985 | 10 | Comorbidity | Ophthalmic |
| Pain | 0.037554071 | 10.36815287 | 3 | Complication | Bone |
| Malignant gastric tumor | 0.037554071 | 10.36815287 | 3 | Comorbidity | Tumorous |
| Spinal muscular atrophy and related syndromes,others | 0.037554071 | 10.36815287 | 3 | Comorbidity | Nervous |
| Left bundle branch block | 0.007881765 | 8.640127389 | 5 | Comorbidity | Circulatory |
| Seborrheic dermatitis | 0.007881765 | 8.640127389 | 5 | Comorbidity | Dermatosis |
| Renal aplasia | 0.025606655 | 6.912101911 | 4 | Comorbidity | Nephrotic |
| Appendicitis,others | 0.049239842 | 4.608067941 | 4 | Comorbidity | Digestive |
| Thrombopenia | 0.020442578 | 4.147261146 | 6 | Risk factor | Hematological |
| Schizophrenia | 0.003873992 | 3.840056617 | 10 | Comorbidity | Nervous |
| Hypostatic pneumonia | 0.001728305 | 3.770237406 | 12 | Complication | Repiratory |
| Obstacle of limb movement | 0.014192938 | 3.456050955 | 8 | Complication | Nervous |
| Occlusion and stenosis of basilar artery | 0.006334029 | 3.168046709 | 11 | Risk factor | Vascular |
| Occlusion and stenosis of anterior cerebral artery | 0.021628319 | 3.072045294 | 8 | Risk factor | Vascular |
| Abnormal tension | 0.021628319 | 3.072045294 | 8 | Comorbidity | Nervous |
| Neuromuscular dysfunction of bladder | 0.000266709 | 2.838898999 | 23 | Complication | Urinary |
| Hypertensive renal disease without renal failure | 0.013387129 | 2.715468608 | 11 | Risk factor | Metabolic |
| Haemorrhoids | 0.047397648 | 2.688039632 | 7 | Comorbidity | Proctology |
| Fascicular block,others and specified | 0.029502987 | 2.592038217 | 9 | Comorbidity | Circulatory |
| Carrier of viral hepatitis | 0.040250598 | 2.392650661 | 9 | Comorbidity | Digestive |
| Rheumatic fever without mention of heart involvement | 0.047969721 | 2.160031847 | 10 | Comorbidity | Immune |
| Vascular dementia | 0.000317744 | 1.97488626 | 44 | Complication | Nervous |
| Lalopathy,others and unspecified | 0.022368562 | 1.958428875 | 17 | Complication | Nervous |
| Paralepsy | 0.016441277 | 1.763291304 | 25 | Complication | Nervous |
| Bell's palsy | 0.014715805 | 1.76190833 | 26 | Comorbidity | Nervous |
| Type 2 diabetes with renal complications | 0.039685067 | 1.622227999 | 23 | Risk factor | Metabolic |
| Benign prostatic hyperplasia | 1.47224E-06 | 1.50839634 | 182 | Comorbidity | Urinary |

| M1 | *P_*value | *RR* | Overlap | Classification according to risk factors comorbidities or complications | Classification according to systems |
| --- | --- | --- | --- | --- | --- |
| Essential (hemorrhagic) thrombocythemia | 0.02040696 | 13.59130435 | 3 | Risk factor | Hematological |
| Tinea pedis | 0.02040696 | 13.59130435 | 3 | Comorbidity | Dermatosis |
| Supraventricular tachycardia | 0.044183527 | 6.795652174 | 3 | Complication | Circulatory |
| Other specific mental disorders caused by brain damage and dysfunction or physical diseases | 0.002107329 | 6.342608696 | 7 | Complication | Nervous |
| Toxic liver disease with hepatitis | 0.023435397 | 6.04057971 | 4 | Comorbidity | Digestive |
| Osteonecrosis | 0.00696217 | 5.436521739 | 6 | Comorbidity | Bone |
| Toxic liver disease with hepatitis | 0.040253446 | 4.530434783 | 4 | Comorbidity | Digestive |
| Arthropathy | 0.018639292 | 3.883229814 | 6 | Comorbidity | Bone |
| Organ-limited amyloidosis | 0.027726709 | 3.397826087 | 6 | Risk factor | Metabolic |
| Motor neuron disease | 0.000141882 | 2.380397937 | 31 | Comorbidity | Nervous |
| Gout | 0.032198331 | 2.166729679 | 11 | Risk factor | Metabolic |
| Atherosclerosis,others | 1.12746E-10 | 1.976364167 | 130 | Risk factor | Vascular |

| M5 | *P_*value | *RR* | Overlap | Classification according to risk factors comorbidities or complications | Classification according to systems |
| --- | --- | --- | --- | --- | --- |
| Injury of shoulder and upper arm | 1.85472E-06 | 24.86753731 | 9 | Comorbidity | Bone |
| Status epilepticus,others | 9.78454E-16 | 23.02549751 | 25 | Complication | Nervous |
| Dysphasia and aphasia | 2.34305E-20 | 22.79524254 | 33 | Complication | Nervous |
| Local infection of skin and subcutaneous tissue | 0.002408165 | 22.10447761 | 4 | Complication | Dermatosis |
| Hereditary ataxia | 3.04119E-25 | 19.80192786 | 43 | Comorbidity | Nervous |
| Cystolith | 0.000270764 | 16.57835821 | 6 | Comorbidity | Urinary |
| Other congenital malformations of cardiac septa | 0.012710999 | 16.57835821 | 3 | Risk factor | Circulatory |
| Dyslalia | 3.91975E-21 | 16.1532721 | 38 | Complication | Nervous |
| Injury of tendon of the rotator cuff of shoulder | 9.69212E-08 | 14.36791045 | 13 | Comorbidity | Bone |
| Asphyxia | 0.001341548 | 13.81529851 | 5 | Complication | Repiratory |
| Diseases of pancreas | 0.006346704 | 11.05223881 | 4 | Comorbidity | Digestive |
| Somatoform disorders,others | 1.22049E-26 | 8.70363806 | 63 | Complication | Nervous |
| Dysphagia | 0.000758172 | 7.736567164 | 7 | Complication | Digestive |
| Coagulation defect | 0.006150473 | 6.907649254 | 5 | Risk factor | Hematological |
| Dislocation of the shoulder joint | 0.006150473 | 6.907649254 | 5 | Comorbidity | Bone |
| Hemiplegia | 1.14429E-50 | 6.727449708 | 140 | Complication | Nervous |
| Dementia | 2.28767E-05 | 6.631343284 | 12 | Complication | Nervous |
| Arthralgia | 0.000695528 | 6.315565032 | 8 | Complication | Bone |
| Rheumatic heart disease | 0.000290185 | 5.526119403 | 10 | Risk factor | Circulatory |
| Allergic asthma | 0.010762865 | 5.526119403 | 5 | Comorbidity | Repiratory |
| Tumours of liver, gallbladder and bile ducts with uncertain or unknown dynamics | 0.022914302 | 5.526119403 | 4 | Comorbidity | Digestive |
| Fracture of neck of femur | 0.022914302 | 5.526119403 | 4 | Comorbidity | Bone |
| Chronic peptic ulcer with bleeding | 0.049983797 | 5.526119403 | 3 | Comorbidity | Digestive |
| Phlebitis and thrombophlebitis of other deep vessels of lower extremities | 1.8985E-07 | 5.065609453 | 22 | Complication | Vascular |
| Leukodermia | 0.000969763 | 4.973507463 | 9 | Comorbidity | Dermatosis |
| Pulmonary malignancy | 0.036325365 | 4.420895522 | 4 | Comorbidity | Tumorous |
| Chronic obstructive pulmonary disease | 8.58851E-05 | 4.144589552 | 15 | Comorbidity | Repiratory |
| Algoneurodystrophy | 1.69887E-05 | 4.144589552 | 18 | Complication | Nervous |
| Pulmonary embolism with no mention of acute cor pulmonale | 0.012693509 | 4.144589552 | 6 | Complication | Circulatory |
| Encephalothlipsis | 0.012693509 | 4.144589552 | 6 | Complication | Nervous |
| Chronic bronchitis | 3.47804E-17 | 3.821802204 | 74 | Comorbidity | Repiratory |
| Degenerative disease of nervous system | 0.00247344 | 3.684079602 | 10 | Complication | Nervous |
| Other disorders of lung | 1.26069E-63 | 3.649324134 | 280 | Comorbidity | Repiratory |
| Motor neuron disease | 3.97244E-08 | 3.516621438 | 35 | Comorbidity | Nervous |
| Alimentary anemia | 0.000139897 | 3.479408513 | 17 | Complication | Hematological |
| Pneumonia | 2.1997E-34 | 3.403769197 | 170 | Complication | Repiratory |
| Pleural conditions,other specified | 8.29098E-06 | 3.289356787 | 25 | Comorbidity | Repiratory |
| Ventricular tachycardia | 0.004578854 | 3.039365672 | 11 | Complication | Circulatory |
| Defects in glycoprotein degradation,others | 3.50851E-09 | 2.935750933 | 51 | Risk factor | Metabolic |
| Pure hypercholesterolaemia | 0.000621033 | 2.935750933 | 17 | Risk factor | Metabolic |
| Diaphragmatic hernia without obstruction or gangrene | 0.008491603 | 2.908483896 | 10 | Comorbidity | Digestive |
| Spondylosis,others | 0.007820199 | 2.763059701 | 11 | Comorbidity | Bone |
| Other specific mental disorders caused by brain damage and dysfunction or physical diseases | 3.22217E-09 | 2.763059701 | 56 | Complication | Nervous |
| Bronchitis | 0.000357204 | 2.698802499 | 21 | Complication | Repiratory |
| Atrial fibrillation and flutter | 0.000235606 | 2.647932214 | 23 | Complication | Circulatory |
| Tumors of the meningeswith uncertain or unknown dynamics | 0.035650474 | 2.456053068 | 8 | Comorbidity | Tumorous |
| Pneumonitis due to food and vomit | 0.035650474 | 2.456053068 | 8 | Complication | Repiratory |
| Abnormal results of liver function studies | 0.000268815 | 2.435239059 | 26 | Comorbidity | Digestive |
| Mild cognitive impairment | 3.86975E-05 | 2.408821278 | 34 | Complication | Nervous |
| Lalopathy,others and specified | 0.008724334 | 2.344414292 | 14 | Complication | Nervous |
| Bedsore | 0.019351238 | 2.337973594 | 11 | Complication | Dermatosis |
| Other disorders of electrolyte and fluid balance, not elsewhere classified | 0.000149033 | 2.314995966 | 31 | Complication | Electrolytical |
| Neuromuscular dysfunction of bladder | 0.007665258 | 2.302549751 | 15 | Complication | Urinary |
| Pulmonary emphysema | 6.44707E-10 | 2.286670098 | 84 | Comorbidity | Repiratory |
| Alzheimer disease | 0.006721192 | 2.267125909 | 16 | Comorbidity | Nervous |
| Bronchiectasia | 0.03956397 | 2.12543054 | 10 | Comorbidity | Repiratory |
| Old myocardial infarction | 0.000103415 | 1.989402985 | 45 | Risk factor | Circulatory |
| Lower extremity arterial embolism and thrombosis | 0.006672352 | 1.925768883 | 23 | Comorbidity | Vascular |
| Calculus of gallbladder without cholecystitis | 4.53659E-06 | 1.916091652 | 69 | Comorbidity | Digestive |
| Hypotonicity and hyponatremia | 0.001309276 | 1.826022064 | 38 | Complication | Electrolytical |
| Cataract | 0.002600861 | 1.790872029 | 35 | Comorbidity | Ophthalmic |
| Cyst of kidney, acquired | 0.00210541 | 1.515602368 | 65 | Comorbidity | Nephrotic |
| Vascular dementia | 0.042592843 | 1.512411626 | 26 | Complication | Nervous |

| M0 | *P_*value | *RR* | Overlap | Classification according to risk factors comorbidities or complications | Classification according to systems |
| --- | --- | --- | --- | --- | --- |
| Cholelithiasis,others | 0.021796128 | 20.75121951 | 2 | Comorbidity | Digestive |
| Fremitus | 0.021796128 | 20.75121951 | 2 | Comorbidity | Nervous |
| Iridocyclitis | 0.04106461 | 10.37560976 | 2 | Comorbidity | Ophthalmic |
| Disorders of autonomic nervous system | 5.2665E-28 | 7.480090754 | 62 | Complication | Nervous |
| Angina | 0.037988207 | 5.187804878 | 3 | Risk factor | Circulatory |
| Atherosclerosis,others | 6.10932E-31 | 3.857598499 | 116 | Risk factor | Vascular |
| Ankylosing Spondylitis | 0.036883379 | 3.772949002 | 4 | Comorbidity | Bone |
| Polyp of stomach and duodenum | 0.013319574 | 3.157794274 | 7 | Comorbidity | Digestive |
| Polyneuropathy | 9.56235E-07 | 3.101986422 | 29 | Comorbidity | Nervous |
| Other specified diseases of gallbladder | 0.005533894 | 2.717421603 | 11 | Comorbidity | Digestive |
| right bundle branch block,others and specified | 0.001733631 | 2.364062982 | 18 | Comorbidity | Circulatory |
| Alimentary anemia | 0.035608338 | 2.305691057 | 8 | Complication | Hematological |
| Pure hypercholesterolemia | 1.30743E-08 | 2.293555841 | 63 | Risk factor | Metabolic |
| Vertebro-basilar artery syndrome | 2.69298E-51 | 2.225242809 | 335 | Risk factor | Nervous |
| Chronic superficial gastritis | 0.024305201 | 2.041103559 | 12 | Comorbidity | Digestive |
| Spinal stenosis | 0.018619656 | 1.743799959 | 20 | Comorbidity | Bone |
| Disc displacement,other specified | 2.45135E-05 | 1.713182076 | 71 | Comorbidity | Bone |
| Atrial premature beats | 0.008220258 | 1.698930252 | 28 | Risk factor | Circulatory |
| Sulfur-loaded amino acid metabolism disorder | 0.000342306 | 1.64118224 | 59 | Risk factor | Metabolic |

| M29 | *P_*value | RR | Overlap | Classification according to risk factors comorbidities or complications | Classification according to systems |
| --- | --- | --- | --- | --- | --- |
| Malignant rectal tumor | 3.46E-07 | 85.83333333 | 5 | Comorbidity | Tumorous |
| Bladder tumor with uncertain or unknown dynamics | 3.46421E-07 | 85.83333333 | 5 | Comorbidity | Tumorous |
| Hereditary retinal degeneration | 0.002346522 | 68.66666667 | 2 | Comorbidity | Ophthalmic |
| Disorder of pituitary gland,others | 0.000419328 | 34.33333333 | 3 | Comorbidity | Endocrine |
| Humeral shaft fracture | 0.004605565 | 34.33333333 | 2 | Comorbidity | Bone |
| Cerebral atherosclerosis | 0.011090117 | 17.16666667 | 2 | Risk factor | Vascular |
| Toxic liver disease with hepatitis | 0.015238749 | 13.73333333 | 2 | Comorbidity | Digestive |
| Viral encephalitis | 0.015238749 | 13.73333333 | 2 | Comorbidity | Nervous |
| Bedsore | 4.70444E-08 | 12.71604938 | 10 | Complication | Dermatosis |
| Pneumonitis due to food and vomit | 5.44896E-06 | 12.64912281 | 7 | Complication | Repiratory |
| Hydrocephalus | 1.53163E-05 | 10.44927536 | 7 | Complication | Nervous |
| Abnormal results of kidney function studies | 0.000106653 | 9.363636364 | 6 | Comorbidity | Nephrotic |
| Encephalothlipsis | 0.006454106 | 9.363636364 | 3 | Complication | Nervous |
| Head injury | 0.007901409 | 8.583333333 | 3 | Complication | Nervous |
| Dilated cardiomyopathy | 0.030882542 | 8.583333333 | 2 | Risk factor | Circulatory |
| Ileus | 0.030882542 | 8.583333333 | 2 | Comorbidity | Digestive |
| Noninfective gastroenteritis and colitis | 0.008490407 | 5.493333333 | 4 | Comorbidity | Digestive |
| Pleural conditions,other specified | 0.000100283 | 5.327586207 | 9 | Comorbidity | Repiratory |
| Quadriplegia | 0.026064555 | 5.15 | 3 | Complication | Nervous |
| Venous embolism and thrombogenesis | 0.002170959 | 4.904761905 | 6 | Complication | Vascular |
| Gastrointestinal hemorrhage | 1.81829E-05 | 4.790697674 | 12 | Complication | Digestive |
| Epilepsy | 9.49951E-08 | 4.401709402 | 20 | Complication | Nervous |
| Alzheimer disease | 0.004355213 | 4.204081633 | 6 | Comorbidity | Nervous |
| Defects in glycoprotein degradation,others | 3.45155E-06 | 4.193384224 | 16 | Risk factor | Metabolic |
| Vascular dementia | 0.000148178 | 3.779816514 | 12 | Complication | Nervous |
| Hypotonicity and hyponatremia | 0.0001005 | 3.458033573 | 14 | Complication | Electrolytical |
| Phlebitis and thrombophlebitis of other deep vessels of lower extremities | 0.040295382 | 3.26984127 | 4 | Complication | Vascular |
| Other disorders of lung | 3.30508E-15 | 3.140568475 | 59 | Comorbidity | Repiratory |
| Calculus of gallbladder without cholecystitis | 2.00538E-05 | 2.91902834 | 21 | Comorbidity | Digestive |
| Atrial fibrillation and flutter | 2.35453E-05 | 2.641025641 | 24 | Complication | Circulatory |
| Other disorders of electrolyte and fluid balance, not elsewhere classified | 0.028917866 | 2.452380952 | 7 | Complication | Electrolytical |
| Kidney stone | 0.045638739 | 2.395348837 | 6 | Comorbidity | Nephrotic |
| Pneumonia | 5.05795E-05 | 2.29984051 | 28 | Complication | Repiratory |
| Heart failure | 0.000187843 | 2.295264624 | 24 | Complication | Circulatory |
| Anemia | 0.021533962 | 2.272058824 | 9 | Comorbidity | Hematological |
| Diseases of stomach and duodenum | 0.001731482 | 2.223021583 | 18 | Comorbidity | Digestive |
| Hypokalemia | 0.031426682 | 2.116438356 | 9 | Complication | Electrolytical |
| Occlusion and stenosis of carotid artery | 0.034517899 | 1.984585742 | 10 | Risk factor | Vascular |
| Occlusion and stenosis of cerebral artery | 0.047021476 | 1.955696203 | 9 | Risk factor | Vascular |

| M4 | *P_*value | *RR* | Overlap | Classification according to risk factors comorbidities or complications | Classification according to systems |
| --- | --- | --- | --- | --- | --- |
| Degenerative changes in both hip joints | 0.001826785 | 55.41935484 | 2 | Comorbidity | Bone |
| Hypertrophic olivary degeneration | 0.001826785 | 55.41935484 | 2 | Comorbidity | Nervous |
| HPV infection | 0.035137162 | 55.41935484 | 1 | Comorbidity | Infectious |
| Disorders of adrenal gland | 1.10617E-05 | 44.33548387 | 4 | Comorbidity | Endocrine |
| Orchitis, epididymitis and inflammation of epididymis - testis,without abscess | 0.003009332 | 36.94623656 | 2 | Comorbidity | Urinary |
| Quadriplegia | 8.54891E-11 | 35.62672811 | 9 | Complication | Nervous |
| Pulmonary embolism with no mention of acute cor pulmonale | 2.84259E-06 | 30.78853047 | 5 | Complication | Circulatory |
| Hydrocephalus | 4.72791E-11 | 27.70967742 | 10 | Complication | Nervous |
| Arteriovenous malformation of cerebral vessels | 0.004461721 | 27.70967742 | 2 | Risk factor | Vascular |
| Lalopathy,others and unspecified | 1.73602E-11 | 19.00092166 | 12 | Complication | Nervous |
| Other specified diseases of pericardium | 5.23552E-07 | 17.63343109 | 7 | Risk factor | Circulatory |
| Respiratory tuberculosis and sequelae of tuberculosis | 2.48433E-09 | 16.79374389 | 10 | Comorbidity | Repiratory |
| Chronic rhinitis | 0.010341387 | 15.83410138 | 2 | Comorbidity | ENT |
| Hemiplegia | 4.44204E-46 | 14.8887819 | 54 | Complication | Nervous |
| Tumours of trachea,bronchi and lungs with uncertain or unknown dynamics | 0.012777857 | 13.85483871 | 2 | Comorbidity | Repiratory |
| Algoneurodystrophy | 5.53267E-07 | 13.0398482 | 8 | Complication | Nervous |
| Venous embolism and thrombogenesis | 1.20845E-07 | 12.78908189 | 9 | Complication | Vascular |
| Diffuse brain injury | 0.002571042 | 12.78908189 | 3 | Complication | Nervous |
| Nontoxic goiter | 0.000537058 | 12.31541219 | 4 | Comorbidity | Thyroid |
| Hyperuricemia without signs of infectious arthritis or gravelly diseases | 7.50398E-08 | 9.37866005 | 11 | Risk factor | Metabolic |
| Injury of tendon of the rotator cuff of shoulder | 0.039623115 | 6.927419355 | 2 | Comorbidity | Bone |
| Epilepsy | 2.36404E-06 | 4.789326961 | 14 | Complication | Nervous |
| Mild cognitive impairment | 0.000791911 | 4.263027295 | 8 | Complication | Nervous |
| Somatoform disorders,others | 0.002249632 | 4.040994624 | 7 | Complication | Nervous |
| Abnormal results of liver function studies | 0.017140681 | 3.463709677 | 5 | Comorbidity | Digestive |
| Vascular dementia | 0.005520048 | 3.402942841 | 7 | Complication | Nervous |
| Other disorders of lung | 0.011544413 | 1.703962594 | 21 | Comorbidity | Repiratory |
