# Supplementary material for: Using the Symptom Patient Similarity Network to Explore the Difference between the Chinese and Western Medicine Pathways of Ischemic Stroke and its Comorbidities
Source: Evid Based Complement Alternat Med. 2021 Dec 1;2021:4961738. doi: 10.1155/2021/4961738 (PMC8654542; doi:10.1155/2021/4961738)
Supplement: Supplementary Materials — Table S1: enriched diseases of each subgroup. Table S2: enriched symptoms, herbs, and drugs of each subgroup. Table S3: pathways of each subgroup. [file 4961738.f1.zip › 4961738.f1/Table S2 Enriched symptoms, herbs and drugs of each subgroup.docx]

**Table S2 Enriched symptoms, herbs and drugs of each subgroup**

**M3** (*P*<0.05, *RR*>1.5)

| **Symptom** | **p_value** | **RR** | **overlap** | **Herb** | **p_value** | **RR** | **overlap** | **Drug** | **p_value** | **RR** | **overlap** |
| --- | --- | --- | --- | --- | --- | --- | --- | --- | --- | --- | --- |
| 头晕 | 0 | 2.211773 | 1822 | 天麻 | 2.44E-81 | 1.735151 | 1197 | 盐酸倍他司汀 | 1.63E-78 | 3.213303 | 514 |
| 眠差 | 2.8E-154 | 2.213527 | 1269 | 石决明 | 1.78E-75 | 3.024194 | 530 | 天麻素注射液 | 2.72E-46 | 1.852636 | 729 |
| 头痛 | 1.4E-146 | 4.808182 | 630 | 钩藤 | 1.3E-67 | 2.307379 | 680 | 甲磺酸倍他司汀 | 9.66E-28 | 2.628823 | 251 |
| 恶心 | 8.3E-114 | 3.840246 | 600 | 首乌藤 | 4.39E-50 | 2.812062 | 401 | 氟哌噻吨美利曲辛 | 9.6E-22 | 2.049731 | 305 |
| 视物旋转 | 3.62E-98 | 7.746588 | 333 | 炒栀子 | 1.81E-49 | 2.345902 | 511 | 盐酸异丙嗪 | 2.87E-21 | 2.658477 | 189 |
| 头胀 | 9.88E-92 | 6.330342 | 348 | 葛根 | 2.39E-48 | 2.461614 | 466 | 盐酸氟桂利嗪 | 1.72E-20 | 3.739003 | 119 |
| 颈部僵硬 | 1.36E-87 | 3.562796 | 511 | 黄芩 | 1E-43 | 1.749792 | 784 | 参芎葡萄糖注射液 | 6.07E-11 | 1.786732 | 209 |
| 心慌 | 5.51E-57 | 1.758678 | 937 | 酸枣仁 | 1.53E-43 | 2.478363 | 420 | 长春西汀 | 5.06E-09 | 1.808967 | 163 |
| 耳鸣 | 1.34E-39 | 3.047759 | 291 | 槲寄生 | 1.59E-36 | 2.304421 | 401 | 左甲状腺素钠 | 7.79E-09 | 3.400174 | 52 |
| 多梦 | 4.04E-26 | 3.584229 | 160 | 杜仲 | 2.24E-31 | 2.475248 | 310 | 氢溴酸高乌甲素 | 1.77E-08 | 2.520161 | 75 |
| 视物不清 | 1.73E-25 | 2.188607 | 313 | 延胡索 | 1.28E-29 | 2.886032 | 235 | 盐酸达克罗宁 | 7.23E-07 | 3.041475 | 44 |
| 呕吐 | 4.05E-20 | 2.895894 | 158 | 益母草 | 1.1E-24 | 2.617091 | 225 | 复方聚乙二醇电解质 | 1.35E-06 | 3.528226 | 35 |
| 饮食差 | 2.48E-19 | 1.520444 | 592 | 柴胡 | 1.42E-24 | 1.735549 | 500 | 薄芝糖肽 | 2.22E-06 | 2.889785 | 43 |
| 背部僵硬 | 2.88E-14 | 3.417819 | 89 | 茯神 | 3.22E-21 | 2.591222 | 196 | 丙泊酚中/长链脂肪乳 | 4.64E-06 | 2.073733 | 72 |
| 口干 | 2.44E-11 | 1.931144 | 178 | 炒蔓荆子 | 2.99E-20 | 3.36163 | 132 | 环磷腺苷葡胺 | 4.51E-05 | 1.627566 | 111 |
| 头重脚轻 | 1.57E-10 | 2.495756 | 98 | 制旋覆花 | 5.64E-17 | 2.836485 | 136 | 洛索洛芬钠 | 0.000144 | 3.629032 | 21 |
| 腰痛 | 1.44E-08 | 1.728111 | 175 | 合欢皮 | 9.29E-17 | 2.675953 | 146 | 尼莫地平 | 0.000764 | 1.918799 | 46 |
| 颈项痛 | 6.15E-08 | 2.572965 | 67 | 秦艽 | 8.9E-15 | 2.617663 | 132 | 盐酸曲唑酮 | 0.001428 | 1.796188 | 49 |
| 汗出 | 1.02E-07 | 1.922227 | 116 | 薄荷 | 2.95E-14 | 2.814352 | 114 | 氯硝西泮 | 0.004653 | 1.78028 | 39 |
| 嗳气 | 2.08E-07 | 3.056027 | 48 | 牡蛎 | 5.23E-13 | 1.634699 | 325 | 葛根素注射液 | 0.011229 | 2.903226 | 12 |
| 反酸 | 1.84E-06 | 1.728111 | 125 | 细辛 | 1.02E-12 | 2.382974 | 131 | 克拉霉素 | 0.013951 | 2.956989 | 11 |
| 膝关节疼痛 | 4.16E-06 | 1.60567 | 148 | 炒麦芽 | 4.48E-12 | 1.900922 | 198 | 吲哒帕胺 | 0.015894 | 1.873049 | 24 |
| 颈部不适 | 9.07E-06 | 4.83871 | 22 | 炒枳壳 | 4.76E-12 | 1.838026 | 215 | 卡马西平 | 0.020843 | 2.150538 | 16 |
| 入睡困难 | 1.29E-05 | 3.046595 | 34 | 藁本 | 1.05E-11 | 5.564516 | 46 | 铝碳酸镁 | 0.027193 | 2.688172 | 10 |
| 口苦 | 2.06E-05 | 1.925078 | 74 | 片姜黄 | 7.39E-11 | 2.12469 | 137 | 钆双胺 | 0.028274 | 1.652242 | 28 |
| 胸痛 | 5.96E-05 | 1.867111 | 71 | 炒苍术 | 9.17E-11 | 1.778183 | 208 | 盐酸帕罗西汀 | 0.032132 | 2.419355 | 11 |
| 大便不爽 | 0.000102 | 3.477823 | 23 | 威灵仙 | 1.07E-10 | 2.335929 | 112 | 缬沙坦氨氯地平 | 0.032584 | 1.680108 | 25 |
| 听力下降 | 0.000189 | 1.602005 | 98 | 香附 | 1.67E-09 | 1.867366 | 159 | 曲克芦丁脑蛋白水解物 | 0.034208 | 2.721774 | 9 |
| 腹胀 | 0.00038 | 1.688984 | 74 | 百合 | 7.89E-09 | 2.794772 | 67 | 普拉洛芬 | 0.034748 | 1.594575 | 29 |
| 干呕 | 0.000717 | 4.032258 | 15 | 干姜 | 3.11E-08 | 1.649949 | 193 |  |  |  |  |
| 咽痛 | 0.000717 | 2.782258 | 23 | 青皮 | 4.41E-08 | 5.846774 | 29 |  |  |  |  |
| 腹部不适 | 0.000717 | 2.782258 | 23 | 薤白 | 2.81E-07 | 2.322581 | 72 |  |  |  |  |
| 头部麻木 | 0.000862 | 2.016129 | 40 | 鸡内金 | 1.27E-06 | 2.202233 | 71 |  |  |  |  |
| 头如裹 | 0.000864 | 2.516129 | 26 | 山楂 | 7.25E-05 | 5.529954 | 16 |  |  |  |  |
| 肢体沉重 | 0.002044 | 2.688172 | 20 | 升麻 | 8.49E-05 | 1.961639 | 60 |  |  |  |  |
| 腰部僵硬 | 0.002752 | 3.387097 | 14 | 浮小麦 | 0.000107 | 2.945302 | 28 |  |  |  |  |
| 急躁易怒 | 0.003296 | 5.443548 | 9 | 木香 | 0.000546 | 1.600203 | 84 |  |  |  |  |
| 腹痛 | 0.004286 | 1.82686 | 37 | 珍珠母 | 0.000583 | 2.764977 | 24 |  |  |  |  |
| 痰少 | 0.005996 | 1.827957 | 34 | 合欢花 | 0.000836 | 4.233871 | 14 |  |  |  |  |
| 视物变形 | 0.00968 | 12.09677 | 5 | 豆蔻 | 0.000902 | 2.164686 | 34 |  |  |  |  |
| 面部肿胀 | 0.00968 | 12.09677 | 5 | 荷叶 | 0.001565 | 3.763441 | 14 |  |  |  |  |
| 干咳 | 0.011268 | 1.866359 | 27 | 猪苓 | 0.003063 | 1.552039 | 68 |  |  |  |  |
| 四肢逆冷 | 0.012333 | 1.799007 | 29 | 通草 | 0.004715 | 1.869501 | 34 |  |  |  |  |
| 视物黑矇 | 0.015453 | 3.870968 | 8 | 瓦楞子 | 0.00476 | 2.764977 | 16 |  |  |  |  |
| 胃脘不适 | 0.015462 | 1.754032 | 29 | 莲子 | 0.00481 | 1.961639 | 30 |  |  |  |  |
| 面部疼痛 | 0.018649 | 4.233871 | 7 | 墨旱莲 | 0.006924 | 3.225806 | 12 |  |  |  |  |
| 上肢疼痛 | 0.018708 | 2.268145 | 15 | 白扁豆 | 0.006979 | 1.624424 | 47 |  |  |  |  |
| 耳聋 | 0.025167 | 1.596774 | 33 | 广藿香 | 0.007604 | 1.896251 | 29 |  |  |  |  |
| 不能平卧 | 0.025711 | 6.048387 | 5 | 天山雪莲 | 0.008943 | 1.529477 | 55 |  |  |  |  |
| 腰膝酸软 | 0.027193 | 2.688172 | 10 | 紫苏叶 | 0.009258 | 1.88172 | 28 |  |  |  |  |
| 不能睁眼 | 0.027972 | 9.677419 | 4 | 徐长卿 | 0.00968 | 12.09677 | 5 |  |  |  |  |
| 眼眶疼痛 | 0.027972 | 9.677419 | 4 | 仙鹤草 | 0.015453 | 3.870968 | 8 |  |  |  |  |
| 眼干 | 0.037342 | 1.935484 | 16 | 降香 | 0.022197 | 4.83871 | 6 |  |  |  |  |
| 背痛 | 0.045258 | 1.693548 | 21 | 煅牡蛎 | 0.022519 | 1.88172 | 21 |  |  |  |  |
|  |  |  |  | 刺蒺藜 | 0.026131 | 1.915323 | 19 |  |  |  |  |
|  |  |  |  | 木贼 | 0.027972 | 9.677419 | 4 |  |  |  |  |
|  |  |  |  | 鳖甲 | 0.02821 | 2.037351 | 16 |  |  |  |  |
|  |  |  |  | 龙眼肉 | 0.030268 | 1.958525 | 17 |  |  |  |  |
|  |  |  |  | 淡竹叶 | 0.030726 | 1.517223 | 37 |  |  |  |  |
|  |  |  |  | 淡豆豉 | 0.031816 | 1.70778 | 24 |  |  |  |  |
|  |  |  |  | 青蒿 | 0.034208 | 2.721774 | 9 |  |  |  |  |
|  |  |  |  | 夏枯草 | 0.035794 | 1.53536 | 33 |  |  |  |  |
|  |  |  |  | 龙胆 | 0.037342 | 1.935484 | 16 |  |  |  |  |
|  |  |  |  | 煅龙骨 | 0.043356 | 1.99241 | 14 |  |  |  |  |

**M2** (*P*<0.05, *RR*>1.5)

| **Symptom** | **p_value** | **RR** | **overlap** | **Herb** | **p_value** | **RR** | **overlap** | **Drug** | **p_value** | **RR** | **overlap** |
| --- | --- | --- | --- | --- | --- | --- | --- | --- | --- | --- | --- |
| 不能站立 | 2.3215E-117 | 27.9953442 | 98 | 鹿角胶 | 2.97429E-24 | 16.50789293 | 28 | 石杉碱甲 | 2.06072E-12 | 23.75115207 | 12 |
| 日常生活能力重度下降 | 4.03156E-90 | 1385.483871 | 50 | 太子参 | 5.8934E-17 | 7.616878153 | 29 | 胞二磷胆碱 | 8.10391E-11 | 16.22029898 | 12 |
| 不能行走 | 6.91473E-90 | 15.01273589 | 94 | 天竺黄 | 9.17759E-16 | 6.46918539 | 30 | 替米沙坦 | 1.50795E-10 | 12.86520737 | 13 |
| 下肢活动不利 | 1.1006E-89 | 63.06340378 | 66 | 地龙 | 1.40546E-13 | 3.789357596 | 40 | 脑苷肌肽 | 2.1207E-09 | 4.360867266 | 24 |
| 痰白 | 4.65005E-38 | 8.92345544 | 57 | 川芎 | 6.0912E-11 | 1.928082867 | 74 | 丙戊酸钠 | 8.09448E-09 | 6.519924099 | 16 |
| 手乏力 | 2.34126E-37 | 6.913915613 | 64 | 党参 | 8.16385E-11 | 2.057117326 | 67 | 胞磷胆碱钠 | 2.28533E-08 | 4.61827957 | 20 |
| 不能端坐 | 7.60814E-36 | 20.24937965 | 38 | 白僵蚕 | 2.1958E-10 | 2.449208923 | 51 | 厄贝沙坦氢氯噻嗪 | 3.37636E-06 | 8.176626124 | 9 |
| 肩痛 | 1.84077E-27 | 14.99048123 | 33 | 全蝎 | 5.91447E-10 | 1.578077159 | 93 | 硝苯地平 | 3.15529E-05 | 2.158291457 | 31 |
| 不能持物 | 5.51511E-25 | 10.23126551 | 36 | 黄芪 | 1.15552E-09 | 2.560442472 | 45 | 托吡酯 | 0.000365607 | 13.85483871 | 4 |
| 手活动不利 | 6.76629E-21 | 13.85483871 | 26 | 赤芍 | 2.46154E-09 | 2.584633686 | 43 | 非布司他 | 0.000595507 | 23.75115207 | 3 |
| 肢体活动不利 | 8.02519E-19 | 1.56784557 | 118 | 穿山甲 | 5.79247E-09 | 5.038123167 | 20 | 银杏叶提取物注射液 | 0.001912485 | 2.154532154 | 18 |
| 日常生活能力中度下降 | 9.60441E-19 | 166.2580645 | 12 | 土鳖虫 | 9.97789E-08 | 7.917050691 | 12 | 盐酸消旋山莨菪碱 | 0.015569037 | 6.157706093 | 3 |
| 咳嗽 | 1.95581E-13 | 2.395118394 | 65 | 化橘红 | 1.36401E-06 | 1.829245209 | 54 | 双氯芬酸钠 | 0.023166293 | 1.776261373 | 15 |
| 尿等待 | 3.71345E-07 | 13.85483871 | 8 | 独活 | 1.65882E-06 | 3.216954634 | 22 | 脂肪乳 | 0.039623115 | 6.927419355 | 2 |
| 喘憋 | 0.000571107 | 2.485828581 | 17 | 厚朴 | 0.00017152 | 2.068989247 | 28 | 盐酸小檗碱 | 0.046154871 | 1.859709894 | 10 |
| 角膜溃疡 | 0.000924125 | 110.8387097 | 2 | 郁金 | 0.000296752 | 1.525303344 | 54 |  |  |  |  |
| 髋关节疼痛 | 0.001803457 | 8.526054591 | 4 | 鸡血藤 | 0.00092204 | 1.784008307 | 31 |  |  |  |  |
| 不能理解 | 0.003081876 | 11.87557604 | 3 | 炒苍耳子 | 0.004461721 | 27.70967742 | 2 |  |  |  |  |
| 足下垂 | 0.004461721 | 27.70967742 | 2 | 防风 | 0.010283254 | 1.646119451 | 24 |  |  |  |  |
| 肢体乏力 | 0.014104675 | 4.524028966 | 4 | 琥珀粉 | 0.043781163 | 6.519924099 | 2 |  |  |  |  |
| 不能转移 | 0.015437769 | 12.31541219 | 2 |  |  |  |  |  |  |  |  |
| 上肢疼痛 | 0.017018302 | 5.937788018 | 3 |  |  |  |  |  |  |  |  |
| 肘关节活动不利 | 0.035137162 | 55.41935484 | 1 |  |  |  |  |  |  |  |  |

**M1** (*P*<0.05, *RR*>1.5)

| **Symptom** | **p_value** | **RR** | **overlap** | **Herb** | **p_value** | **RR** | **overlap** | **Drug** | **p_value** | **RR** | **overlap** |
| --- | --- | --- | --- | --- | --- | --- | --- | --- | --- | --- | --- |
| 口角歪斜 | 1.8E-136 | 5.782636 | 411 | 桑枝 | 1.82E-49 | 2.436536 | 384 | 依达拉奉 | 9.08E-46 | 1.92278 | 525 |
| 肢体活动不利 | 1E-127 | 1.612795 | 1126 | 石菖蒲 | 3.43E-29 | 1.563791 | 583 | 疏血通注射液 | 1.55E-13 | 2.112261 | 145 |
| 言语不利 | 2E-114 | 2.150839 | 818 | 鸡血藤 | 1.27E-13 | 1.655351 | 266 | 肝素钠 | 2.36E-09 | 1.733193 | 158 |
| 肢体麻木 | 1.23E-47 | 2.273495 | 412 | 蜈蚣 | 1.51E-11 | 1.55106 | 278 | 奥扎格雷钠 | 4.65E-08 | 1.584317 | 178 |
| 手乏力 | 7.51E-41 | 3.113018 | 235 | 豨莶草 | 5.02E-11 | 2.254168 | 102 | 碘克沙醇 | 4.23E-07 | 2.139372 | 68 |
| 胸闷 | 3.17E-27 | 1.712351 | 446 | 蝉蜕 | 7.8E-11 | 2.243009 | 101 | 复方氯化钠 | 1.58E-05 | 1.648945 | 99 |
| 肢体反应迟钝 | 8.83E-14 | 1.899532 | 187 | 清半夏 | 2.67E-10 | 1.943751 | 130 | 格列吡嗪 | 8.79E-05 | 1.614082 | 88 |
| 下肢乏力 | 6.66E-09 | 2.046003 | 98 | 木瓜 | 8.74E-05 | 1.941615 | 51 | 丹参多酚酸盐 | 0.000245 | 1.538007 | 92 |
| 大便不调 | 4.89E-06 | 3.25262 | 28 | 络石藤 | 0.002915 | 11.32609 | 5 | 乳酸钠林格 | 0.000509 | 1.600303 | 71 |
| 伸舌歪斜 | 9.82E-05 | 4.027053 | 16 | 白芍 | 0.005928 | 2.677075 | 13 | 阿替普酶 | 0.000523 | 3.926377 | 13 |
| 走路歪斜 | 0.000546 | 2.013527 | 36 | 大腹皮 | 0.008027 | 2.265217 | 16 | 甲磺酸溴隐亭 | 0.000693 | 13.5913 | 6 |
| 善笑 | 0.001032 | 2.430965 | 22 | 泽兰 | 0.022714 | 1.998721 | 15 | 混合糖电解质 | 0.001193 | 6.04058 | 8 |
| 手指挛急 | 0.00104 | 7.928261 | 7 | 土鳖虫 | 0.032345 | 1.595224 | 25 | 尿激酶 | 0.001944 | 3.02029 | 14 |
| 痰少 | 0.001045 | 2.222477 | 26 |  |  |  |  | 硫酸阿托品 | 0.012261 | 2.131969 | 16 |
| 下颌抖动 | 0.003732 | 6.795652 | 6 |  |  |  |  | 氨甲环酸 | 0.01267 | 2.471146 | 12 |
| 失语 | 0.01007 | 1.941615 | 21 |  |  |  |  | 还原型谷胱甘肽 | 0.02051 | 2.373085 | 11 |
| 足内翻 | 0.01044 | 3.136455 | 9 |  |  |  |  | 盐酸多巴胺 | 0.02588 | 2.265217 | 11 |
| 肢体抽搐 | 0.011271 | 1.579418 | 38 |  |  |  |  | 盐酸瑞芬太尼 | 0.026053 | 2.384439 | 10 |
| 角弓反张 | 0.015471 | 2.664962 | 10 |  |  |  |  | 盐酸氯丙嗪 | 0.032937 | 2.265217 | 10 |
| 肩部僵硬 | 0.021642 | 4.530435 | 5 |  |  |  |  | 地佐辛 | 0.039537 | 2.076449 | 11 |
| 眼睑浮肿 | 0.044184 | 6.795652 | 3 |  |  |  |  | 拉氧头孢钠 | 0.044184 | 6.795652 | 3 |

**M5** (*P*<0.05, *RR*>1.5)

| **Symptom** | **p_value** | **RR** | **overlap** | **Herb** | **p_value** | **RR** | **overlap** | **Drug** | **p_value** | **RR** | **overlap** |
| --- | --- | --- | --- | --- | --- | --- | --- | --- | --- | --- | --- |
| 咳嗽 | 0 | 7.224588 | 889 | 川贝母 | 1.03E-32 | 2.54223 | 236 | 盐酸氨溴索 | 2.85E-46 | 3.419477 | 224 |
| 咳痰 | 0 | 12.10483 | 736 | 女贞子 | 8.82E-25 | 9.804405 | 55 | 盐酸左氧氟沙星 | 2.21E-31 | 2.246258 | 276 |
| 痰粘 | 4.7E-120 | 28.07866 | 188 | 山茱萸 | 2.73E-21 | 2.469438 | 164 | 开塞露 | 1.64E-29 | 1.952099 | 337 |
| 生活不能自理 | 1.9E-100 | 52.31393 | 142 | 苦杏仁 | 3.62E-16 | 2.313646 | 139 | 哌拉西林钠他唑巴坦钠 | 3.34E-25 | 4.047299 | 104 |
| 痰白 | 3.24E-72 | 5.944765 | 213 | 枸杞子 | 2.45E-13 | 2.321381 | 113 | 布地奈德 | 8.27E-18 | 2.928843 | 106 |
| 精神不振 | 1.84E-51 | 4.27898 | 199 | 鸡血藤 | 3.57E-13 | 1.682493 | 232 | 二羟丙茶碱 | 2.29E-17 | 2.933971 | 103 |
| 吞咽困难 | 6.95E-37 | 3.244694 | 192 | 熟地黄 | 1.76E-12 | 2.021396 | 139 | 吲哚美辛 | 3.27E-17 | 2.451987 | 134 |
| 痰黄 | 9.14E-29 | 12.3275 | 58 | 菟丝子 | 2.32E-12 | 3.186231 | 64 | 硫酸特布他林 | 1.68E-14 | 2.935751 | 85 |
| 不能端坐 | 2.52E-28 | 6.734958 | 78 | 桑叶 | 6.19E-09 | 2.76306 | 54 | 痰热清注射液 | 1.43E-12 | 4.835354 | 42 |
| 吞咽呛咳 | 2.53E-22 | 1.83 | 304 | 麦冬 | 1.23E-08 | 1.723066 | 140 | 氯化钾 | 1.61E-11 | 1.55896 | 257 |
| 痰难咯 | 8.28E-19 | 9.850909 | 41 | 黑芝麻 | 1.55E-08 | 3.525283 | 37 | 盐酸溴己新 | 6.66E-10 | 4.675947 | 33 |
| 不能言语 | 1.31E-15 | 2.868751 | 95 | 蜜紫菀 | 7.6E-08 | 3.245499 | 37 | 维生素C | 1.85E-09 | 1.6001 | 194 |
| 瘫痪 | 3.28E-11 | 7.033243 | 28 | 芦根 | 4.44E-06 | 3.341375 | 26 | 葡萄糖氯化钠 | 4.76E-09 | 1.93773 | 108 |
| 舌僵硬 | 3.59E-09 | 7.736567 | 21 | 薏苡仁 | 5.42E-06 | 1.637936 | 107 | 头孢西丁钠 | 7.96E-08 | 2.897843 | 43 |
| 强哭 | 5.55E-09 | 4.14459 | 33 | 鱼腥草 | 1.7E-05 | 4.14459 | 18 | 头孢哌酮钠舒巴坦钠 | 1.33E-07 | 2.042831 | 78 |
| 理解力下降 | 2.52E-08 | 4.662663 | 27 | 冬瓜仁 | 4.35E-05 | 9.947015 | 9 | 多索茶碱 | 4.06E-07 | 7.368159 | 16 |
| 失语 | 1.16E-07 | 3.908719 | 29 | 玄参 | 0.000104 | 1.594786 | 86 | 胞二磷胆碱 | 3.78E-06 | 3.921762 | 22 |
| 喉中痰鸣 | 3.19E-07 | 5.833126 | 19 | 浙贝母 | 0.000134 | 2.706671 | 24 | 丹参多酚酸盐 | 2.07E-05 | 1.689641 | 85 |
| 打鼾 | 1.54E-06 | 2.98954 | 33 | 蒲公英 | 0.000263 | 3.239449 | 17 | 头孢他啶 | 3.71E-05 | 4.6051 | 15 |
| 肢体乏力 | 3.78E-06 | 3.921762 | 22 | 蜜麻黄 | 0.000771 | 1.931459 | 36 | 甲泼尼龙琥珀酸钠 | 8.28E-05 | 2.504023 | 29 |
| 肢体反应迟钝 | 5.74E-06 | 1.526368 | 137 | 白前 | 0.001043 | 2.76306 | 17 | 孟鲁司特钠 | 0.000113 | 4.298093 | 14 |
| 感冒 | 7.28E-06 | 5.201054 | 16 | 紫苏子 | 0.001341 | 2.368337 | 21 | 盐酸坦洛新 | 0.000139 | 1.533259 | 96 |
| 痰多 | 7.81E-06 | 3.68408 | 22 | 前胡 | 0.002324 | 2.061985 | 25 | 复方氨基酸 | 0.000174 | 2.417677 | 28 |
| 喘憋 | 2.31E-05 | 1.648777 | 91 | 金银花 | 0.002329 | 1.824156 | 34 | 氟康唑 | 0.000223 | 4.225856 | 13 |
| 下肢活动不利 | 2.73E-05 | 2.350189 | 37 | 红景天 | 0.002408 | 22.10448 | 4 | 热毒宁注射液 | 0.000453 | 2.637466 | 21 |
| 大便失禁 | 0.000282 | 1.973614 | 40 | 生石膏 | 0.00311 | 1.82305 | 32 | 呋塞米 | 0.000527 | 1.691053 | 56 |
| 不能翻身 | 0.00059 | 5.526119 | 9 | 蜜枇杷叶 | 0.003269 | 3.014247 | 12 | 利伐沙班 | 0.000581 | 4.341951 | 11 |
| 言语减少 | 0.000931 | 3.68408 | 12 | 蜜百部 | 0.004168 | 2.060587 | 22 | 脂肪乳 | 0.00059 | 5.526119 | 9 |
| 消瘦 | 0.00097 | 4.973507 | 9 | 瓜蒌子 | 0.008315 | 4.736674 | 6 | 磷酸奥司他韦 | 0.00064 | 2.481115 | 22 |
| 发热 | 0.000995 | 1.84204 | 39 | 路路通 | 0.01261 | 2.532805 | 11 | 复方甲氧那明 | 0.000758 | 4.6051 | 10 |
| 小便失禁 | 0.001222 | 1.501663 | 75 | 紫草 | 0.012711 | 16.57836 | 3 | 头孢米诺钠 | 0.000758 | 7.736567 | 7 |
| 意识不清 | 0.001271 | 2.269656 | 23 | 鹿角胶 | 0.016119 | 1.646078 | 28 | 非那雄胺 | 0.00087 | 2.009498 | 32 |
| 手疼痛 | 0.001342 | 13.8153 | 5 | 射干 | 0.017019 | 2.947264 | 8 | 异丙托溴铵 | 0.001132 | 2.054583 | 29 |
| 两目斜视 | 0.002351 | 2.426101 | 18 | 透骨草 | 0.028165 | 8.289179 | 3 | 奥氮平 | 0.001349 | 1.611785 | 56 |
| 不能遵嘱活动 | 0.002408 | 22.10448 | 4 | 白芥子 | 0.031153 | 2.368337 | 9 | 注射用益气复脉（冻干粉） | 0.002794 | 1.907827 | 29 |
| 膝关节肿胀 | 0.002408 | 22.10448 | 4 | 白及 | 0.036968 | 3.453825 | 5 | 爱普列特 | 0.002947 | 6.631343 | 6 |
| 蚁走感 | 0.002408 | 22.10448 | 4 |  |  |  |  | 盐酸胺碘酮 | 0.003065 | 2.873582 | 13 |
| 呼吸困难 | 0.002478 | 5.526119 | 7 |  |  |  |  | 灭菌注射用水 | 0.004495 | 2.187422 | 19 |
| 咽痒 | 0.002506 | 3.377073 | 11 |  |  |  |  | 石杉碱甲 | 0.005068 | 2.660724 | 13 |
| 交流障碍 | 0.009904 | 2.394652 | 13 |  |  |  |  | 人血白蛋白 | 0.006159 | 2.162395 | 18 |
| 坐立困难 | 0.012711 | 16.57836 | 3 |  |  |  |  | 乙酰半胱氨酸 | 0.006278 | 2.368337 | 15 |
|  |  |  |  |  |  |  |  | 双歧杆菌三联活菌 | 0.007109 | 2.652537 | 12 |
|  |  |  |  |  |  |  |  | 复方阿嗪米特 | 0.007705 | 1.966924 | 21 |
|  |  |  |  |  |  |  |  | 青霉素钠 | 0.008315 | 4.736674 | 6 |
|  |  |  |  |  |  |  |  | 整蛋白型肠内营养剂 | 0.009047 | 3.108442 | 9 |
|  |  |  |  |  |  |  |  | 硫酸镁 | 0.015957 | 1.705592 | 25 |
|  |  |  |  |  |  |  |  | 茶碱 | 0.017767 | 2.511872 | 10 |
|  |  |  |  |  |  |  |  | 盐酸莫西沙星 | 0.018111 | 3.22357 | 7 |
|  |  |  |  |  |  |  |  | 丙戊酸钠 | 0.021416 | 1.532453 | 33 |
|  |  |  |  |  |  |  |  | 双岐杆菌三联活菌 | 0.024299 | 1.578891 | 28 |
|  |  |  |  |  |  |  |  | 盐酸左西替利嗪 | 0.024909 | 1.84204 | 17 |
|  |  |  |  |  |  |  |  | 硫酸阿米卡星 | 0.025115 | 2.486754 | 9 |
|  |  |  |  |  |  |  |  | 七叶皂苷钠 | 0.040104 | 1.890515 | 13 |
|  |  |  |  |  |  |  |  | 去乙酰毛花苷 | 0.04677 | 1.677572 | 17 |
|  |  |  |  |  |  |  |  | 复方脑肽节苷脂 | 0.049984 | 5.526119 | 3 |
|  |  |  |  |  |  |  |  | 硫酸羟氯喹 | 0.049984 | 5.526119 | 3 |

**M0** (*P*<0.05, *RR*>1.5)

| **Symptom** | **p_value** | **RR** | **overlap** | **Herb** | **p_value** | **RR** | **overlap** | **Drug** | **p_value** | **RR** | **overlap** |
| --- | --- | --- | --- | --- | --- | --- | --- | --- | --- | --- | --- |
| 行走不稳 | 8.3E-280 | 6.945894 | 482 | 天麻 | 3.85E-47 | 1.786557 | 421 | 盐酸倍他司汀 | 7.83E-19 | 2.172611 | 156 |
| 站立不稳 | 1.5E-254 | 14.76851 | 353 | 菊花 | 1.45E-26 | 3.616255 | 107 | 氯沙坦钾 | 9.02E-14 | 6.843487 | 31 |
| 头晕 | 9.3E-101 | 1.807509 | 566 | 清半夏 | 8.06E-25 | 3.405492 | 107 | 天麻素注射液 | 9.02E-14 | 1.611607 | 226 |
| 乏力 | 1.61E-29 | 3.546465 | 121 | 香橼 | 1.07E-20 | 41.50244 | 24 | 奥扎格雷钠 | 1.09E-07 | 1.767566 | 100 |
| 目赤 | 8E-15 | 55.33659 | 16 | 蜈蚣 | 8.33E-13 | 1.798141 | 161 | 银杏达莫注射液 | 2.18E-07 | 3.13226 | 32 |
| 耳鸣 | 1.57E-13 | 2.36808 | 97 | 延胡索 | 1.83E-12 | 2.467552 | 83 | 丹参酮IIA磺酸钠注射液 | 9.51E-05 | 1.556341 | 84 |
| 颈部僵硬 | 9.78E-11 | 1.836013 | 129 | 槲寄生 | 3.46E-09 | 1.773609 | 120 | 丹参多酚酸盐 | 0.000801 | 1.657446 | 50 |
| 恶心 | 1.13E-10 | 1.762364 | 142 | 莲子心 | 1.31E-08 | 4.244568 | 27 | 参芎葡萄糖注射液 | 0.001221 | 1.523691 | 63 |
| 头重脚轻 | 1.94E-09 | 3.063938 | 44 | 木瓜 | 1.38E-08 | 3.088922 | 39 | 丙泊酚中/长链脂肪乳 | 0.002213 | 1.980078 | 25 |
| 面色红 | 9.52E-08 | 6.299477 | 17 | 石决明 | 1.57E-08 | 1.666156 | 132 | 甲钴胺 | 0.002242 | 1.578897 | 49 |
| 焦虑 | 1.24E-07 | 2.465491 | 48 | 红芪 | 2.26E-08 | 1.570527 | 155 | 依降钙素 | 0.021796 | 20.75122 | 2 |
| 腰痛 | 6.89E-07 | 1.969308 | 67 | 杜仲 | 1.81E-07 | 1.808775 | 91 | 辛伐他汀 | 0.027644 | 2.075122 | 11 |
| 背痛 | 8.53E-05 | 3.925906 | 14 | 益母草 | 2.11E-06 | 1.89936 | 67 | 酮咯酸氨丁三醇 | 0.031844 | 2.829712 | 6 |
| 头部麻木 | 0.000178 | 2.857052 | 19 | 豨莶草 | 4.95E-06 | 2.067016 | 51 | 鍀[99mTC]亚甲基二膦酸盐 | 0.041065 | 10.37561 | 2 |
| 烦躁 | 0.000182 | 1.69517 | 58 | 炒蔓荆子 | 5.4E-06 | 2.287097 | 41 | 帕瑞昔布钠 | 0.064504 | 6.917073 | 2 |
| 头如裹 | 0.000341 | 3.549551 | 13 | 牛膝 | 1.15E-05 | 1.502065 | 119 | 妥布霉素 | 0.064676 | 2.305691 | 6 |
| 膝关节疼痛 | 0.000745 | 1.653613 | 51 | 牡蛎 | 1.96E-05 | 1.537127 | 104 | 尼莫地平 | 0.070312 | 1.613984 | 14 |
| 颈项痛 | 0.001979 | 2.11355 | 22 | 盐杜仲 | 6.06E-05 | 1.833024 | 53 | 恩替卡韦 | 0.091233 | 5.187805 | 2 |
| 气短 | 0.004002 | 2.01748 | 21 | 佛手 | 7.56E-05 | 2.369234 | 29 |  |  |  |  |
| 口苦 | 0.010749 | 1.741361 | 24 | 炒苍术 | 0.000241 | 1.611271 | 66 |  |  |  |  |
| 多汗 | 0.012666 | 2.105196 | 14 | 龟板胶 | 0.002528 | 31.12683 | 3 |  |  |  |  |
| 视物重影 | 0.020547 | 1.962953 | 14 | 野菊花 | 0.008074 | 6.917073 | 4 |  |  |  |  |
| 视物黑矇 | 0.02223 | 4.611382 | 4 | 藁本 | 0.011403 | 2.305691 | 12 |  |  |  |  |
| 背部僵硬 | 0.024503 | 1.663266 | 21 | 芡实 | 0.012473 | 1.859779 | 19 |  |  |  |  |
| 走路歪斜 | 0.02626 | 1.763854 | 17 | 瓦楞子 | 0.01332 | 3.157794 | 7 |  |  |  |  |
| 头部震颤 | 0.045864 | 3.458537 | 4 | 天冬 | 0.013525 | 1.842398 | 19 |  |  |  |  |
|  |  |  |  | 狗脊 | 0.025247 | 2.207577 | 10 |  |  |  |  |
|  |  |  |  | 刺蒺藜 | 0.031461 | 2.371568 | 8 |  |  |  |  |
|  |  |  |  | 川楝子 | 0.040357 | 1.693977 | 16 |  |  |  |  |

**M29** (*P*<0.05, *RR*>1.5)

| **Symptom** | **p_value** | **RR** | **overlap** | **Herb** | **p_value** | **RR** | **overlap** | **Drug** | **p_value** | **RR** | **overlap** |
| --- | --- | --- | --- | --- | --- | --- | --- | --- | --- | --- | --- |
| 小便失禁 | 7E-137 | 21.45833 | 135 | 人参 | 1.61E-06 | 11.94203 | 8 | 维生素C | 1.11E-28 | 3.795201 | 86 |
| 大便失禁 | 2.8E-109 | 51.21858 | 91 | 益智仁 | 0.000644 | 2.995526 | 13 | 氯化钾 | 1.19E-26 | 3.109555 | 97 |
| 不能言语 | 1.37E-53 | 12.22602 | 73 | 芒硝 | 0.000713 | 6.242424 | 6 | 吲哚美辛 | 1.15E-17 | 4.347115 | 49 |
| 多寐 | 1.07E-47 | 10.28551 | 71 | 诃子肉 | 0.000719 | 25.75 | 3 | 维生素B6 | 2.94E-17 | 2.921986 | 72 |
| 意识不清 | 3.32E-31 | 24.63043 | 33 | 西红花 | 0.002347 | 68.66667 | 2 | 开塞露 | 5.71E-16 | 2.419845 | 85 |
| 肢体反应迟钝 | 9.56E-19 | 3.661422 | 61 | 赤芍 | 0.002428 | 1.562297 | 42 | 盐酸氨溴索 | 1.25E-15 | 3.484962 | 54 |
| 两目斜视 | 8.39E-16 | 16.30833 | 19 | 附子 | 0.003088 | 2.607595 | 12 | 复方氯化钠 | 3.01E-15 | 4.382979 | 42 |
| 呼吸急促 | 4.61E-13 | 20.89855 | 14 | 酒大黄 | 0.005489 | 3.483092 | 7 | 头孢哌酮钠舒巴坦钠 | 1.17E-13 | 4.730971 | 35 |
| 昏迷 | 2.23E-11 | 26.97619 | 11 | 鱼腥草 | 0.006272 | 4.63964 | 5 | 葡萄糖氯化钠 | 1.7E-13 | 3.855615 | 42 |
| 呕吐 | 1.98E-09 | 3.814815 | 29 | 皂角刺 | 0.009525 | 7.923077 | 3 | 异丙托溴铵 | 1.26E-12 | 8.383721 | 21 |
| 发热 | 3.42E-07 | 4.478261 | 18 | 补骨脂 | 0.010765 | 5.08642 | 4 | 灭菌注射用水 | 2.35E-12 | 11.67333 | 17 |
| 表情淡漠 | 8.25E-07 | 8.803419 | 10 | 车前子 | 0.011838 | 2.719472 | 8 | 葡萄糖 | 4.12E-12 | 2.022311 | 88 |
| 精神不振 | 1.29E-06 | 2.591195 | 32 | 羚羊角 | 0.015239 | 13.73333 | 2 | 甘露醇 | 9.88E-12 | 5.297143 | 27 |
| 咀嚼肌无力 | 1.98E-06 | 42.91667 | 5 | 法半夏 | 0.019943 | 11.44444 | 2 | 胰岛素 | 9.96E-12 | 2.466363 | 63 |
| 交流障碍 | 2.38E-06 | 9.088235 | 9 | 肉豆蔻 | 0.020397 | 5.722222 | 3 | 布地奈德 | 1.68E-11 | 4.150183 | 33 |
| 瘫痪 | 6.91E-05 | 6.539683 | 8 | 麦冬 | 0.025787 | 1.521868 | 25 | 哌拉西林钠他唑巴坦钠 | 3.74E-11 | 4.588326 | 29 |
| 失语 | 0.000749 | 4.430108 | 8 | 穿山甲 | 0.039309 | 1.807018 | 12 | 盐酸乌拉地尔 | 6.26E-11 | 15.3908 | 13 |
| 哈欠 | 0.002347 | 68.66667 | 2 | 桑叶 | 0.040002 | 2.019608 | 9 | 呋塞米 | 9.79E-11 | 4.556082 | 28 |
| 大便不调 | 0.002703 | 4.005556 | 7 | 覆盆子 | 0.043105 | 3.193798 | 4 | 硫酸特布他林 | 1.77E-10 | 4.430108 | 28 |
| 喉中痰鸣 | 0.003602 | 5.364583 | 5 | 五味子 | 0.044728 | 1.645367 | 15 | 头孢呋辛钠 | 4.58E-10 | 6.242424 | 20 |
| 便秘 | 0.008953 | 1.976019 | 16 | 前胡 | 0.045639 | 2.395349 | 6 | 苯巴比妥钠 | 4.81E-10 | 5.229581 | 23 |
| 大便黏液 | 0.009525 | 7.923077 | 3 | 蜜款冬花 | 0.046022 | 3.121212 | 4 | 丙戊酸钠 | 1.27E-09 | 5.503817 | 21 |
| 运动迟缓 | 0.013491 | 2.472 | 9 |  |  |  |  | 人血白蛋白 | 1.84E-09 | 9.613333 | 14 |
| 痰多 | 0.01911 | 3.433333 | 5 |  |  |  |  | 奥美拉唑钠 | 1.36E-08 | 5.669725 | 18 |
| 声音低沉 | 0.020517 | 3.366013 | 5 |  |  |  |  | 二羟丙茶碱 | 1.45E-08 | 3.57373 | 28 |
| 肢体抽搐 | 0.023303 | 2.23913 | 9 |  |  |  |  | 地塞米松磷酸钠 | 2.58E-08 | 3.704011 | 26 |
| 言语减少 | 0.051783 | 3.814815 | 3 |  |  |  |  | 地佐辛 | 2.6E-08 | 13.73333 | 10 |
| 独立坐不稳 | 0.055807 | 34.33333 | 1 |  |  |  |  | 泮托拉唑钠 | 5.3E-08 | 2.246946 | 50 |
| 睡眠倒错 | 0.055807 | 34.33333 | 1 |  |  |  |  | 白眉蛇毒血凝酶 | 3.6E-07 | 15.25926 | 8 |
| 不能理解 | 0.08207 | 4.577778 | 2 |  |  |  |  | 地西泮 | 4.33E-07 | 4.669333 | 17 |
| 半身不遂 | 0.082537 | 17.16667 | 1 |  |  |  |  | 盐酸艾司洛尔 | 4.94E-07 | 20.02778 | 7 |
| 理解力下降 | 0.085083 | 2.49697 | 4 |  |  |  |  | 去乙酰毛花苷 | 7.65E-07 | 6.754098 | 12 |
|  |  |  |  |  |  |  |  | 双岐杆菌三联活菌 | 2.29E-06 | 4.63964 | 15 |
|  |  |  |  |  |  |  |  | 肝素钙 | 3.67E-06 | 5.189922 | 13 |
|  |  |  |  |  |  |  |  | 复方氨基酸 | 9.42E-06 | 5.15 | 12 |
|  |  |  |  |  |  |  |  | 哌拉西林钠舒巴坦钠 | 1.16E-05 | 24.52381 | 5 |
|  |  |  |  |  |  |  |  | 胞磷胆碱钠 | 1.26E-05 | 3.016736 | 21 |
|  |  |  |  |  |  |  |  | 盐酸左氧氟沙星 | 1.57E-05 | 1.85688 | 49 |
|  |  |  |  |  |  |  |  | 硫酸阿托品 | 6.91E-05 | 6.539683 | 8 |
|  |  |  |  |  |  |  |  | 硝普钠 | 7.02E-05 | 27.46667 | 4 |
|  |  |  |  |  |  |  |  | 醒脑静注射液 | 7.02E-05 | 27.46667 | 4 |
|  |  |  |  |  |  |  |  | 疏血通注射液 | 7.49E-05 | 2.246106 | 28 |
|  |  |  |  |  |  |  |  | 地高辛 | 9.23E-05 | 6.242424 | 8 |
|  |  |  |  |  |  |  |  | 钠钾镁钙 | 0.00011 | 7.282828 | 7 |
|  |  |  |  |  |  |  |  | 盐酸瑞芬太尼 | 0.000131 | 8.956522 | 6 |
|  |  |  |  |  |  |  |  | 氟康唑 | 0.00016 | 8.583333 | 6 |
|  |  |  |  |  |  |  |  | 咪达唑仑 | 0.000189 | 11.44444 | 5 |
|  |  |  |  |  |  |  |  | 氨甲环酸 | 0.00033 | 7.357143 | 6 |
|  |  |  |  |  |  |  |  | 肝素钠封管 | 0.000419 | 34.33333 | 3 |
|  |  |  |  |  |  |  |  | 肠内营养乳剂(TPF-T) | 0.000473 | 9.035088 | 5 |
|  |  |  |  |  |  |  |  | 奥美拉唑镁 | 0.000499 | 13.73333 | 4 |
|  |  |  |  |  |  |  |  | 参麦注射液 | 0.000713 | 6.242424 | 6 |
|  |  |  |  |  |  |  |  | 左西孟旦 | 0.000719 | 25.75 | 3 |
|  |  |  |  |  |  |  |  | 蒙脱石散 | 0.000837 | 3.284058 | 11 |
|  |  |  |  |  |  |  |  | 痰热清注射液 | 0.000946 | 3.814815 | 9 |
|  |  |  |  |  |  |  |  | 尼可刹米 | 0.001109 | 10.5641 | 4 |
|  |  |  |  |  |  |  |  | 单唾液酸四己糖神经节苷脂钠 | 0.001294 | 3.333333 | 10 |
|  |  |  |  |  |  |  |  | 乳糖酸阿奇霉素 | 0.001654 | 17.16667 | 3 |
|  |  |  |  |  |  |  |  | 热毒宁注射液 | 0.002269 | 4.143678 | 7 |
|  |  |  |  |  |  |  |  | 酒石酸布托啡诺 | 0.003115 | 12.875 | 3 |
|  |  |  |  |  |  |  |  | 酚磺乙胺 | 0.003115 | 12.875 | 3 |
|  |  |  |  |  |  |  |  | 依诺肝素钠 | 0.003469 | 3.814815 | 7 |
|  |  |  |  |  |  |  |  | 脑蛋白水解物 | 0.003829 | 3.059406 | 9 |
|  |  |  |  |  |  |  |  | 盐酸胺碘酮 | 0.004054 | 5.20202 | 5 |
|  |  |  |  |  |  |  |  | 多种微量元素 | 0.004606 | 34.33333 | 2 |
|  |  |  |  |  |  |  |  | 肝素钠 | 0.005389 | 1.704044 | 27 |
|  |  |  |  |  |  |  |  | 盐酸多奈哌齐 | 0.005652 | 4.768519 | 5 |
|  |  |  |  |  |  |  |  | 混合糖电解质 | 0.006454 | 9.363636 | 3 |
|  |  |  |  |  |  |  |  | 糜蛋白酶 | 0.007482 | 5.722222 | 4 |
|  |  |  |  |  |  |  |  | 阿替普酶 | 0.007482 | 5.722222 | 4 |
|  |  |  |  |  |  |  |  | 醋甲唑胺 | 0.007533 | 22.88889 | 2 |
|  |  |  |  |  |  |  |  | 盐酸右美托咪定 | 0.00849 | 5.493333 | 4 |
|  |  |  |  |  |  |  |  | 马来酸依那普利 | 0.008931 | 1.698969 | 24 |
|  |  |  |  |  |  |  |  | 螺内酯 | 0.009148 | 2.089855 | 14 |
|  |  |  |  |  |  |  |  | 丙泊酚 | 0.009525 | 7.923077 | 3 |
|  |  |  |  |  |  |  |  | 盐酸氯丙嗪 | 0.009584 | 5.282051 | 4 |
|  |  |  |  |  |  |  |  | 辛伐他汀 | 0.010571 | 3.433333 | 6 |
|  |  |  |  |  |  |  |  | 头孢克洛 | 0.01109 | 17.16667 | 2 |
|  |  |  |  |  |  |  |  | 盐酸利多卡因 | 0.013361 | 1.638171 | 24 |
|  |  |  |  |  |  |  |  | 盐酸多巴胺 | 0.013401 | 4.735632 | 4 |
|  |  |  |  |  |  |  |  | 厄贝沙坦氢氯噻嗪 | 0.013906 | 3.21875 | 6 |
|  |  |  |  |  |  |  |  | 硝酸甘油 | 0.014859 | 4.577778 | 4 |
|  |  |  |  |  |  |  |  | 硫酸庆大霉素 | 0.015239 | 13.73333 | 2 |
|  |  |  |  |  |  |  |  | 美罗培南 | 0.015239 | 13.73333 | 2 |
|  |  |  |  |  |  |  |  | 盐酸溴己新 | 0.015823 | 3.121212 | 6 |
|  |  |  |  |  |  |  |  | 尿激酶 | 0.016414 | 4.430108 | 4 |
|  |  |  |  |  |  |  |  | 托吡酯 | 0.017848 | 6.058824 | 3 |
|  |  |  |  |  |  |  |  | 盐酸替罗非班 | 0.020397 | 5.722222 | 3 |
|  |  |  |  |  |  |  |  | 舒血宁注射液 | 0.02621 | 1.998236 | 11 |
|  |  |  |  |  |  |  |  | 盐酸小檗碱 | 0.027866 | 1.757679 | 15 |
|  |  |  |  |  |  |  |  | 琥珀酸美托洛尔 | 0.028851 | 1.62272 | 19 |
|  |  |  |  |  |  |  |  | 比阿培南 | 0.029182 | 4.904762 | 3 |
|  |  |  |  |  |  |  |  | 硫酸镁 | 0.030249 | 2.427609 | 7 |
|  |  |  |  |  |  |  |  | 甲硝唑 | 0.030883 | 8.583333 | 2 |
|  |  |  |  |  |  |  |  | 整蛋白型肠内营养剂 | 0.032488 | 4.681818 | 3 |
|  |  |  |  |  |  |  |  | 人胰岛素 | 0.033266 | 2.215054 | 8 |
|  |  |  |  |  |  |  |  | 多索茶碱 | 0.043521 | 4.12 | 3 |
|  |  |  |  |  |  |  |  | 盐酸贝那普利 | 0.043521 | 4.12 | 3 |
|  |  |  |  |  |  |  |  | 奥氮平 | 0.048425 | 1.745763 | 12 |
|  |  |  |  |  |  |  |  | 环磷腺苷葡胺 | 0.048961 | 1.697085 | 13 |

**M4**(*P*<0.05, *RR*>1.5)

| **Symptom** | **p_value** | **RR** | **overlap** | **Herb** | **p_value** | **RR** | **overlap** | **Drug** | **p_value** | **RR** | **overlap** |
| --- | --- | --- | --- | --- | --- | --- | --- | --- | --- | --- | --- |
| 不能站立 | 2.3215E-117 | 27.9953442 | 98 | 鹿角胶 | 2.97429E-24 | 16.50789293 | 28 | 石杉碱甲 | 2.06072E-12 | 23.75115207 | 12 |
| 日常生活能力重度下降 | 4.03156E-90 | 1385.483871 | 50 | 太子参 | 5.8934E-17 | 7.616878153 | 29 | 胞二磷胆碱 | 8.10391E-11 | 16.22029898 | 12 |
| 不能行走 | 6.91473E-90 | 15.01273589 | 94 | 天竺黄 | 9.17759E-16 | 6.46918539 | 30 | 替米沙坦 | 1.50795E-10 | 12.86520737 | 13 |
| 下肢活动不利 | 1.1006E-89 | 63.06340378 | 66 | 地龙 | 1.40546E-13 | 3.789357596 | 40 | 脑苷肌肽 | 2.1207E-09 | 4.360867266 | 24 |
| 痰白 | 4.65005E-38 | 8.92345544 | 57 | 川芎 | 6.0912E-11 | 1.928082867 | 74 | 丙戊酸钠 | 8.09448E-09 | 6.519924099 | 16 |
| 手乏力 | 2.34126E-37 | 6.913915613 | 64 | 党参 | 8.16385E-11 | 2.057117326 | 67 | 胞磷胆碱钠 | 2.28533E-08 | 4.61827957 | 20 |
| 不能端坐 | 7.60814E-36 | 20.24937965 | 38 | 白僵蚕 | 2.1958E-10 | 2.449208923 | 51 | 厄贝沙坦氢氯噻嗪 | 3.37636E-06 | 8.176626124 | 9 |
| 肩痛 | 1.84077E-27 | 14.99048123 | 33 | 全蝎 | 5.91447E-10 | 1.578077159 | 93 | 硝苯地平 | 3.15529E-05 | 2.158291457 | 31 |
| 不能持物 | 5.51511E-25 | 10.23126551 | 36 | 黄芪 | 1.15552E-09 | 2.560442472 | 45 | 托吡酯 | 0.000365607 | 13.85483871 | 4 |
| 手活动不利 | 6.76629E-21 | 13.85483871 | 26 | 赤芍 | 2.46154E-09 | 2.584633686 | 43 | 非布司他 | 0.000595507 | 23.75115207 | 3 |
| 肢体活动不利 | 8.02519E-19 | 1.56784557 | 118 | 穿山甲 | 5.79247E-09 | 5.038123167 | 20 | 银杏叶提取物注射液 | 0.001912485 | 2.154532154 | 18 |
| 日常生活能力中度下降 | 9.60441E-19 | 166.2580645 | 12 | 土鳖虫 | 9.97789E-08 | 7.917050691 | 12 | 盐酸消旋山莨菪碱 | 0.015569037 | 6.157706093 | 3 |
| 咳嗽 | 1.95581E-13 | 2.395118394 | 65 | 化橘红 | 1.36401E-06 | 1.829245209 | 54 | 双氯芬酸钠 | 0.023166293 | 1.776261373 | 15 |
| 尿等待 | 3.71345E-07 | 13.85483871 | 8 | 独活 | 1.65882E-06 | 3.216954634 | 22 | 脂肪乳 | 0.039623115 | 6.927419355 | 2 |
| 喘憋 | 0.000571107 | 2.485828581 | 17 | 厚朴 | 0.00017152 | 2.068989247 | 28 | 盐酸小檗碱 | 0.046154871 | 1.859709894 | 10 |
| 角膜溃疡 | 0.000924125 | 110.8387097 | 2 | 郁金 | 0.000296752 | 1.525303344 | 54 |  |  |  |  |
| 髋关节疼痛 | 0.001803457 | 8.526054591 | 4 | 鸡血藤 | 0.00092204 | 1.784008307 | 31 |  |  |  |  |
| 不能理解 | 0.003081876 | 11.87557604 | 3 | 炒苍耳子 | 0.004461721 | 27.70967742 | 2 |  |  |  |  |
| 足下垂 | 0.004461721 | 27.70967742 | 2 | 防风 | 0.010283254 | 1.646119451 | 24 |  |  |  |  |
| 肢体乏力 | 0.014104675 | 4.524028966 | 4 | 琥珀粉 | 0.043781163 | 6.519924099 | 2 |  |  |  |  |
| 不能转移 | 0.015437769 | 12.31541219 | 2 |  |  |  |  |  |  |  |  |
| 上肢疼痛 | 0.017018302 | 5.937788018 | 3 |  |  |  |  |  |  |  |  |
| 肘关节活动不利 | 0.035137162 | 55.41935484 | 1 |  |  |  |  |  |  |  |  |
