# Supplementary material for: Using the Symptom Patient Similarity Network to Explore the Difference between the Chinese and Western Medicine Pathways of Ischemic Stroke and its Comorbidities
Source: Evid Based Complement Alternat Med. 2021 Dec 1;2021:4961738. doi: 10.1155/2021/4961738 (PMC8654542; doi:10.1155/2021/4961738)
Supplement: Supplementary Materials — Table S1: enriched diseases of each subgroup. Table S2: enriched symptoms, herbs, and drugs of each subgroup. Table S3: pathways of each subgroup. [file 4961738.f1.zip › 4961738.f1/Table S3 Pathways of each subgroup.docx]

**Table S3 Pathways of each subgroup**

Pathways in M3

| **symptom** | **overlap** | **p_value** | **drug** | **overlap** | **p_value** | **herb** | **overlap** | **p_value** |
| --- | --- | --- | --- | --- | --- | --- | --- | --- |
| AGE-RAGE signaling pathway in diabetic complications | 56 | 3.4E-17 | Neuroactive ligand-receptor interaction | 47 | 9.33419E-48 | Pathways in cancer | 81 | 1.4964E-32 |
| Neuroactive ligand-receptor interaction | 108 | 1.3E-16 | Nicotine addiction | 27 | 1.19665E-46 | Hepatitis B | 50 | 1.61268E-31 |
| Alzheimer's disease | 76 | 3.7E-16 | Retrograde endocannabinoid signaling | 25 | 3.84057E-29 | AGE-RAGE signaling pathway in diabetic complications | 41 | 2.00999E-29 |
| Calcium signaling pathway | 79 | 5.4E-16 | GABAergic synapse | 24 | 4.09009E-29 | FoxO signaling pathway | 42 | 5.3789E-25 |
| Amyotrophic lateral sclerosis (ALS) | 35 | 3.1E-15 | Morphine addiction | 22 | 2.02543E-25 | Prostate cancer | 34 | 1.5772E-23 |
| Pathways in cancer | 136 | 4.1E-15 | Calcium signaling pathway | 26 | 7.04619E-24 | Endocrine resistance | 35 | 3.29809E-23 |
| Hepatitis B | 66 | 3.4E-14 | cAMP signaling pathway | 19 | 4.10807E-14 | PI3K-Akt signaling pathway | 61 | 1.92265E-21 |
| Bladder cancer | 29 | 2.2E-13 | Adrenergic signaling in cardiomyocytes | 16 | 7.33299E-13 | Apoptosis | 39 | 4.60628E-21 |
| Proteoglycans in cancer | 81 | 4E-13 | Serotonergic synapse | 14 | 2.98443E-12 | Pancreatic cancer | 28 | 4.62234E-21 |
| Malaria | 32 | 4.1E-13 | Amphetamine addiction | 11 | 3.48951E-11 | Platinum drug resistance | 29 | 2.08312E-20 |
| Pancreatic cancer | 38 | 8.5E-13 | Cocaine addiction | 9 | 8.13164E-10 | HTLV-I infection | 50 | 3.48651E-19 |
| cAMP signaling pathway | 78 | 1.8E-12 | Circadian entrainment | 11 | 1.69441E-09 | TNF signaling pathway | 33 | 4.37731E-19 |
| Colorectal cancer | 35 | 1.5E-11 | Arrhythmogenic right ventricular cardiomyopathy (ARVC) | 10 | 2.08188E-09 | HIF-1 signaling pathway | 32 | 4.7202E-19 |
| HIF-1 signaling pathway | 48 | 2.9E-11 | Oxytocin signaling pathway | 13 | 3.42631E-09 | Chagas disease (American trypanosomiasis) | 32 | 6.54223E-19 |
| EGFR tyrosine kinase inhibitor resistance | 41 | 3E-11 | Cardiac muscle contraction | 10 | 3.53134E-09 | Toxoplasmosis | 33 | 4.78274E-18 |
| Endocrine resistance | 46 | 3.5E-11 | Hypertrophic cardiomyopathy (HCM) | 10 | 6.55769E-09 | Leishmaniasis | 26 | 1.51056E-17 |
| MicroRNAs in cancer | 100 | 9.7E-11 | Cholinergic synapse | 11 | 9.03357E-09 | Proteoglycans in cancer | 42 | 3.49664E-17 |
| Prostate cancer | 42 | 3E-10 | Renin secretion | 9 | 9.66512E-09 | EGFR tyrosine kinase inhibitor resistance | 27 | 3.82288E-17 |
| FoxO signaling pathway | 55 | 3.2E-10 | Dilated cardiomyopathy | 10 | 1.30651E-08 | Bladder cancer | 20 | 7.08838E-17 |
| Glioma | 34 | 4.7E-10 | Aldosterone synthesis and secretion | 9 | 7.98868E-08 | Tuberculosis | 38 | 2.32877E-16 |
| Nicotine addiction | 25 | 6.3E-10 | Taste transduction | 9 | 9.90482E-08 | Malaria | 21 | 3.28241E-16 |
| Long-term potentiation | 34 | 8E-10 | MAPK signaling pathway | 14 | 1.5655E-07 | Rheumatoid arthritis | 27 | 5.73123E-16 |
| Serotonergic synapse | 47 | 3.5E-09 | Nitrogen metabolism | 5 | 4.46116E-07 | Colorectal cancer | 23 | 5.75226E-16 |
| Circadian entrainment | 42 | 3.6E-09 | cGMP-PKG signaling pathway | 11 | 6.14226E-07 | p53 signaling pathway | 24 | 7.35021E-16 |
| Neurotrophin signaling pathway | 49 | 4.9E-09 | Glutamatergic synapse | 9 | 1.52329E-06 | Insulin resistance | 29 | 2.28079E-15 |
| Renal cell carcinoma | 33 | 6.3E-09 | Long-term potentiation | 7 | 3.26773E-06 | MAPK signaling pathway | 44 | 4.83835E-15 |
| Chagas disease (American trypanosomiasis) | 44 | 8.2E-09 | Alzheimer's disease | 10 | 5.1187E-06 | T cell receptor signaling pathway | 28 | 6.57451E-15 |
| PI3K-Akt signaling pathway | 104 | 1.1E-08 | Bile secretion | 7 | 5.3629E-06 | Small cell lung cancer | 25 | 2.1387E-14 |
| Influenza A | 63 | 1.3E-08 | Alcoholism | 10 | 8.9879E-06 | Glioma | 22 | 2.33281E-14 |
| Estrogen signaling pathway | 42 | 1.6E-08 | Vascular smooth muscle contraction | 8 | 2.08678E-05 | Chronic myeloid leukemia | 23 | 3.47113E-14 |
| Thyroid cancer | 19 | 2.4E-08 | Dopaminergic synapse | 8 | 3.52297E-05 | Influenza A | 35 | 3.62E-14 |
| Apoptosis | 53 | 2.6E-08 | Amyotrophic lateral sclerosis (ALS) | 5 | 0.000130295 | Osteoclast differentiation | 30 | 5.95934E-14 |
| Dopaminergic synapse | 50 | 2.7E-08 | Insulin secretion | 6 | 0.000172177 | Prolactin signaling pathway | 22 | 2.57296E-13 |
| Amphetamine addiction | 32 | 2.7E-08 | Gastric acid secretion | 5 | 0.00074889 | Cell cycle | 28 | 6.09694E-13 |
| Melanoma | 33 | 3.9E-08 | Type II diabetes mellitus | 4 | 0.001184839 | Pertussis | 22 | 6.54818E-13 |
| Oxytocin signaling pathway | 57 | 4.5E-08 | GnRH signaling pathway | 5 | 0.001898324 | Focal adhesion | 36 | 7.10525E-13 |
| Long-term depression | 29 | 8.8E-08 | Inflammatory mediator regulation of TRP channels | 5 | 0.0026279 | NF-kappa B signaling pathway | 24 | 9.91946E-13 |
| Chronic myeloid leukemia | 33 | 8.9E-08 | Chemical carcinogenesis | 4 | 0.008282903 | Melanoma | 21 | 1.8464E-12 |
| Rap1 signaling pathway | 70 | 9.3E-08 | Gap junction | 4 | 0.010566411 | Toll-like receptor signaling pathway | 25 | 3.86275E-12 |
| Hematopoietic cell lineage | 37 | 1.1E-07 | Salivary secretion | 4 | 0.010982692 | Measles | 28 | 5.57939E-12 |
| Retrograde endocannabinoid signaling | 41 | 1.1E-07 | Pancreatic secretion | 4 | 0.014194571 | VEGF signaling pathway | 19 | 7.66028E-12 |
| Renin secretion | 30 | 1.3E-07 | One carbon pool by folate | 2 | 0.015902911 | Non-small cell lung cancer | 18 | 1.51008E-11 |
| TNF signaling pathway | 43 | 1.9E-07 | Regulation of lipolysis in adipocytes | 3 | 0.017095413 | Viral carcinogenesis | 34 | 2.21021E-11 |
| Pertussis | 33 | 2E-07 | Arginine biosynthesis | 2 | 0.017466564 | Transcriptional misregulation in cancer | 31 | 5.58716E-11 |
| cGMP-PKG signaling pathway | 58 | 2E-07 | Proximal tubule bicarbonate reclamation | 2 | 0.020779988 | African trypanosomiasis | 14 | 6.08435E-11 |
| Adrenergic signaling in cardiomyocytes | 53 | 2.2E-07 | Arachidonic acid metabolism | 3 | 0.022372592 | MicroRNAs in cancer | 41 | 9.46322E-11 |
| Cytokine-cytokine receptor interaction | 82 | 2.3E-07 | Vitamin digestion and absorption | 2 | 0.0225271 | Amyotrophic lateral sclerosis (ALS) | 16 | 3.13695E-10 |
| Non-small cell lung cancer | 27 | 2.6E-07 | Collecting duct acid secretion | 2 | 0.028114491 | Cytokine-cytokine receptor interaction | 37 | 4.86001E-10 |
| Hypertrophic cardiomyopathy (HCM) | 35 | 3E-07 | Metabolism of xenobiotics by cytochrome P450 | 3 | 0.03409738 | Apoptosis - multiple species | 13 | 5.79814E-10 |
| GnRH signaling pathway | 37 | 4.2E-07 | Thiamine metabolism | 1 | 0.038376649 | AMPK signaling pathway | 24 | 7.10696E-10 |
| Rheumatoid arthritis | 36 | 7E-07 | Rap1 signaling pathway | 5 | 0.054845354 | Chemokine signaling pathway | 30 | 7.94879E-10 |
| Endometrial cancer | 25 | 7.7E-07 | Regulation of actin cytoskeleton | 5 | 0.058530949 | Adipocytokine signaling pathway | 18 | 9.24228E-10 |
| Tuberculosis | 59 | 7.9E-07 | Drug metabolism - other enzymes | 2 | 0.073662263 | B cell receptor signaling pathway | 18 | 1.51974E-09 |
| African trypanosomiasis | 19 | 8.8E-07 | Arginine and proline metabolism | 2 | 0.082163746 | Amoebiasis | 21 | 1.7033E-09 |
| Inflammatory mediator regulation of TRP channels | 38 | 1.2E-06 | Steroid hormone biosynthesis | 2 | 0.109287009 | Ras signaling pathway | 33 | 1.72399E-09 |
| MAPK signaling pathway | 77 | 1.6E-06 | VEGF signaling pathway | 2 | 0.118793875 | NOD-like receptor signaling pathway | 16 | 1.97658E-09 |
| Focal adhesion | 64 | 2.4E-06 | Retinol metabolism | 2 | 0.131775222 | Neurotrophin signaling pathway | 23 | 2.33439E-09 |
| Viral myocarditis | 26 | 2.5E-06 | Drug metabolism - cytochrome P450 | 2 | 0.145064656 | Central carbon metabolism in cancer | 17 | 3.40613E-09 |
| Inflammatory bowel disease (IBD) | 28 | 2.7E-06 | Selenocompound metabolism | 1 | 0.15330746 | ErbB signaling pathway | 19 | 7.51167E-09 |
| Sphingolipid signaling pathway | 43 | 3E-06 | Leishmaniasis | 2 | 0.155208464 | Thyroid hormone signaling pathway | 22 | 8.94929E-09 |
| Cocaine addiction | 23 | 3.6E-06 | Pertussis | 2 | 0.165485053 | Legionellosis | 15 | 9.84812E-09 |
| Amoebiasis | 37 | 4.7E-06 | Ras signaling pathway | 4 | 0.181946007 | Estrogen signaling pathway | 20 | 1.01945E-08 |
| Toxoplasmosis | 42 | 4.7E-06 | Terpenoid backbone biosynthesis | 1 | 0.193794211 | Inflammatory bowel disease (IBD) | 16 | 1.5907E-08 |
| Ras signaling pathway | 69 | 5E-06 | Renin-angiotensin system | 1 | 0.201658494 | Epstein-Barr virus infection | 29 | 1.64767E-08 |
| Gastric acid secretion | 30 | 5.5E-06 | Small cell lung cancer | 2 | 0.204052791 | Ovarian steroidogenesis | 14 | 2.12517E-08 |
| Central carbon metabolism in cancer | 28 | 5.6E-06 | Ascorbate and aldarate metabolism | 1 | 0.232363241 | Salmonella infection | 18 | 3.13714E-08 |
| VEGF signaling pathway | 26 | 7.8E-06 | Phototransduction | 1 | 0.232363241 | Epithelial cell signaling in Helicobacter pylori infection | 16 | 3.18777E-08 |
| T cell receptor signaling pathway | 38 | 8.4E-06 | Glyoxylate and dicarboxylate metabolism | 1 | 0.239854913 | Endometrial cancer | 14 | 3.69283E-08 |
| Leishmaniasis | 29 | 9.1E-06 | Linoleic acid metabolism | 1 | 0.247274188 | Non-alcoholic fatty liver disease (NAFLD) | 24 | 4.8906E-08 |
| Glutamatergic synapse | 40 | 1.2E-05 | Estrogen signaling pathway | 2 | 0.250724465 | Rap1 signaling pathway | 29 | 5.53997E-08 |
| ErbB signaling pathway | 33 | 1.4E-05 | AGE-RAGE signaling pathway in diabetic complications | 2 | 0.257952279 | PPAR signaling pathway | 16 | 7.57265E-08 |
| Cholinergic synapse | 39 | 1.4E-05 | HIF-1 signaling pathway | 2 | 0.265183983 | Glucagon signaling pathway | 19 | 8.02448E-08 |
| Measles | 45 | 1.6E-05 | Propanoate metabolism | 1 | 0.269104522 | Metabolism of xenobiotics by cytochrome P450 | 16 | 9.30703E-08 |
| Fc epsilon RI signaling pathway | 27 | 1.8E-05 | Pentose and glucuronate interconversions | 1 | 0.297239354 | Jak-STAT signaling pathway | 24 | 1.18771E-07 |
| Platinum drug resistance | 29 | 2.3E-05 | Carbon metabolism | 2 | 0.301305301 | Renal cell carcinoma | 15 | 1.7474E-07 |
| Prolactin signaling pathway | 28 | 2.8E-05 | Aldosterone-regulated sodium reabsorption | 1 | 0.317634277 | Insulin signaling pathway | 22 | 1.92077E-07 |
| Non-alcoholic fatty liver disease (NAFLD) | 48 | 3.4E-05 | Thyroid hormone signaling pathway | 2 | 0.319276539 | Drug metabolism - cytochrome P450 | 15 | 2.63879E-07 |
| Prion diseases | 17 | 3.9E-05 | Sphingolipid signaling pathway | 2 | 0.326437108 | Allograft rejection | 11 | 3.61876E-07 |
| Vascular smooth muscle contraction | 40 | 4.5E-05 | Platelet activation | 2 | 0.333578637 | Hepatitis C | 21 | 3.80391E-07 |
| Adherens junction | 28 | 5E-05 | AMPK signaling pathway | 2 | 0.337141653 | Oxytocin signaling pathway | 23 | 4.25598E-07 |
| Toll-like receptor signaling pathway | 36 | 6.9E-05 | Porphyrin and chlorophyll metabolism | 1 | 0.337443007 | Intestinal immune network for IgA production | 12 | 6.63385E-07 |
| Circadian rhythm | 15 | 7.2E-05 | Pathways in cancer | 5 | 0.343974654 | Longevity regulating pathway | 17 | 6.98343E-07 |
| Osteoclast differentiation | 42 | 8.2E-05 | ABC transporters | 1 | 0.350331423 | Type II diabetes mellitus | 12 | 8.49292E-07 |
| Legionellosis | 22 | 0.00012 | Cysteine and methionine metabolism | 1 | 0.356682229 | Acute myeloid leukemia | 13 | 9.28643E-07 |
| Dilated cardiomyopathy | 31 | 0.00013 | Carbohydrate digestion and absorption | 1 | 0.362971562 | Leukocyte transendothelial migration | 19 | 1.00589E-06 |
| Thyroid hormone signaling pathway | 38 | 0.00016 | Valine, leucine and isoleucine degradation | 1 | 0.375368158 | Steroid hormone biosynthesis | 13 | 1.14908E-06 |
| Apoptosis - multiple species | 15 | 0.00028 | Systemic lupus erythematosus | 2 | 0.375924951 | Progesterone-mediated oocyte maturation | 17 | 1.28586E-06 |
| Salivary secretion | 30 | 0.00031 | Ovarian steroidogenesis | 1 | 0.387525858 | Platelet activation | 19 | 1.69394E-06 |
| Insulin signaling pathway | 42 | 0.00035 | Vibrio cholerae infection | 1 | 0.393516551 | Prion diseases | 10 | 1.86875E-06 |
| Allograft rejection | 16 | 0.00036 | Parkinson's disease | 2 | 0.403558439 | Type I diabetes mellitus | 11 | 1.91009E-06 |
| Salmonella infection | 29 | 0.00039 | Starch and sucrose metabolism | 1 | 0.428261548 | GnRH signaling pathway | 16 | 2.19284E-06 |
| Type II diabetes mellitus | 19 | 0.00041 | Synaptic vesicle cycle | 1 | 0.461034614 | Herpes simplex infection | 24 | 2.26277E-06 |
| Longevity regulating pathway - multiple species | 23 | 0.00055 | Glioma | 1 | 0.471540104 | Chemical carcinogenesis | 15 | 2.7217E-06 |
| Melanogenesis | 32 | 0.00057 | Tuberculosis | 2 | 0.516823941 | Adherens junction | 14 | 3.86774E-06 |
| Cell adhesion molecules (CAMs) | 42 | 0.00067 | Biosynthesis of amino acids | 1 | 0.521102218 | Inflammatory mediator regulation of TRP channels | 16 | 5.98553E-06 |
| Autoimmune thyroid disease | 19 | 0.00129 | Metabolic pathways | 12 | 0.55478822 | Fc epsilon RI signaling pathway | 13 | 6.40274E-06 |
| Gap junction | 28 | 0.00136 | Peroxisome | 1 | 0.557414269 | Viral myocarditis | 12 | 7.24479E-06 |
| GABAergic synapse | 28 | 0.00136 | Huntington's disease | 2 | 0.563744352 | Graft-versus-host disease | 10 | 8.97058E-06 |
| B cell receptor signaling pathway | 24 | 0.00143 | Salmonella infection | 1 | 0.570316695 | Autoimmune thyroid disease | 11 | 1.39499E-05 |
| Jak-STAT signaling pathway | 44 | 0.00169 | Protein digestion and absorption | 1 | 0.586942303 | Glutathione metabolism | 11 | 1.39499E-05 |
| NOD-like receptor signaling pathway | 20 | 0.00173 | NF-kappa B signaling pathway | 1 | 0.595014637 | Caffeine metabolism | 4 | 1.87239E-05 |
| Acute myeloid leukemia | 20 | 0.00173 | Phosphatidylinositol signaling system | 1 | 0.618306208 | Longevity regulating pathway - multiple species | 12 | 2.09236E-05 |
| p53 signaling pathway | 23 | 0.00179 | Amoebiasis | 1 | 0.622057042 | Sphingolipid signaling pathway | 17 | 2.16059E-05 |
| Hepatitis C | 38 | 0.00206 | Melanogenesis | 1 | 0.625771379 | Amphetamine addiction | 12 | 3.38389E-05 |
| Arrhythmogenic right ventricular cardiomyopathy (ARVC) | 24 | 0.00218 | Glucagon signaling pathway | 1 | 0.62944957 | Galactose metabolism | 8 | 4.63476E-05 |
| Type I diabetes mellitus | 16 | 0.00246 | Choline metabolism in cancer | 1 | 0.62944957 | Alzheimer's disease | 20 | 5.56899E-05 |
| Taste transduction | 26 | 0.00254 | Chagas disease (American trypanosomiasis) | 1 | 0.640270734 | cAMP signaling pathway | 22 | 7.44568E-05 |
| Longevity regulating pathway | 28 | 0.00399 | PI3K-Akt signaling pathway | 3 | 0.648123924 | Arachidonic acid metabolism | 11 | 7.84934E-05 |
| Small cell lung cancer | 26 | 0.00435 | Insulin resistance | 1 | 0.657614953 | TGF-beta signaling pathway | 13 | 7.91488E-05 |
| Natural killer cell mediated cytotoxicity | 37 | 0.00441 | TNF signaling pathway | 1 | 0.660983112 | Natural killer cell mediated cytotoxicity | 17 | 9.03735E-05 |
| Morphine addiction | 27 | 0.00492 | Toxoplasmosis | 1 | 0.686773468 | Oocyte meiosis | 16 | 0.000107274 |
| Choline metabolism in cancer | 29 | 0.00609 | Neurotrophin signaling pathway | 1 | 0.692911441 | Choline metabolism in cancer | 14 | 0.00014473 |
| Phospholipase D signaling pathway | 38 | 0.00781 | Oocyte meiosis | 1 | 0.701895783 | Thyroid cancer | 7 | 0.00022025 |
| Cardiac muscle contraction | 23 | 0.00976 | Oxidative phosphorylation | 1 | 0.730005038 | Aldosterone-regulated sodium reabsorption | 8 | 0.000264162 |
| Adipocytokine signaling pathway | 21 | 0.01073 | Insulin signaling pathway | 1 | 0.745593662 | Insulin secretion | 12 | 0.00035909 |
| Signaling pathways regulating pluripotency of stem cells | 37 | 0.01187 | Phospholipase D signaling pathway | 1 | 0.755487546 | Serotonergic synapse | 14 | 0.000433655 |
| Asthma | 11 | 0.01278 | MicroRNAs in cancer | 2 | 0.792945603 | Melanogenesis | 13 | 0.000470571 |
| Herpes simplex infection | 46 | 0.0133 | HTLV-I infection | 1 | 0.922355513 | Gap junction | 12 | 0.000496801 |
| Epstein-Barr virus infection | 49 | 0.01436 | Olfactory transduction | 1 | 0.984286247 | Shigellosis | 10 | 0.000549631 |
| Renin-angiotensin system | 9 | 0.01495 | Glycolysis / Gluconeogenesis | 0 | 1 | Signaling pathways regulating pluripotency of stem cells | 16 | 0.000566425 |
| Regulation of actin cytoskeleton | 52 | 0.01531 | Citrate cycle (TCA cycle) | 0 | 1 | Wnt signaling pathway | 16 | 0.000566425 |
| Aldosterone synthesis and secretion | 23 | 0.01557 | Pentose phosphate pathway | 0 | 1 | Dopaminergic synapse | 15 | 0.000599126 |
| Chemokine signaling pathway | 46 | 0.0161 | Fructose and mannose metabolism | 0 | 1 | Carbohydrate digestion and absorption | 8 | 0.000852474 |
| Intestinal immune network for IgA production | 15 | 0.0162 | Galactose metabolism | 0 | 1 | Aldosterone synthesis and secretion | 11 | 0.000874712 |
| Insulin resistance | 29 | 0.01815 | Fatty acid biosynthesis | 0 | 1 | Bile secretion | 10 | 0.001117352 |
| Dorso-ventral axis formation | 10 | 0.02098 | Fatty acid elongation | 0 | 1 | Fatty acid metabolism | 8 | 0.001140451 |
| RIG-I-like receptor signaling pathway | 20 | 0.02159 | Fatty acid degradation | 0 | 1 | Long-term depression | 9 | 0.001243288 |
| HTLV-I infection | 60 | 0.0218 | Synthesis and degradation of ketone bodies | 0 | 1 | mTOR signaling pathway | 16 | 0.001274635 |
| Complement and coagulation cascades | 22 | 0.02213 | Steroid biosynthesis | 0 | 1 | Cocaine addiction | 8 | 0.001310976 |
| Graft-versus-host disease | 13 | 0.02563 | Primary bile acid biosynthesis | 0 | 1 | Hematopoietic cell lineage | 11 | 0.001590508 |
| ECM-receptor interaction | 22 | 0.0332 | Ubiquinone and other terpenoid-quinone biosynthesis | 0 | 1 | Tryptophan metabolism | 7 | 0.00172274 |
| Citrate cycle (TCA cycle) | 10 | 0.03402 | Purine metabolism | 0 | 1 | Arginine biosynthesis | 5 | 0.001926225 |
| Taurine and hypotaurine metabolism | 5 | 0.03455 | Caffeine metabolism | 0 | 1 | Fat digestion and absorption | 7 | 0.001999311 |
| Glucagon signaling pathway | 26 | 0.0359 | Pyrimidine metabolism | 0 | 1 | cGMP-PKG signaling pathway | 16 | 0.003142646 |
| Regulation of lipolysis in adipocytes | 16 | 0.03734 | Alanine, aspartate and glutamate metabolism | 0 | 1 | Regulation of lipolysis in adipocytes | 8 | 0.00314709 |
| TGF-beta signaling pathway | 22 | 0.0426 | Glycine, serine and threonine metabolism | 0 | 1 | RIG-I-like receptor signaling pathway | 9 | 0.003696096 |
| Caffeine metabolism | 3 | 0.04477 | Valine, leucine and isoleucine biosynthesis | 0 | 1 | Tyrosine metabolism | 6 | 0.004074599 |
| Tryptophan metabolism | 12 | 0.0471 | Lysine biosynthesis | 0 | 1 | Huntington's disease | 17 | 0.005607705 |
| Pancreatic secretion | 24 | 0.05764 | Lysine degradation | 0 | 1 | Cytosolic DNA-sensing pathway | 8 | 0.006547329 |
| Transcriptional misregulation in cancer | 41 | 0.06231 | Histidine metabolism | 0 | 1 | Vascular smooth muscle contraction | 12 | 0.007091212 |
| Mineral absorption | 14 | 0.07663 | Tyrosine metabolism | 0 | 1 | Renin secretion | 8 | 0.007199853 |
| Insulin secretion | 21 | 0.0802 | Phenylalanine metabolism | 0 | 1 | Fc gamma R-mediated phagocytosis | 10 | 0.007622292 |
| Aldosterone-regulated sodium reabsorption | 11 | 0.08262 | Tryptophan metabolism | 0 | 1 | Hippo signaling pathway | 14 | 0.008262178 |
| Progesterone-mediated oocyte maturation | 23 | 0.11035 | Phenylalanine, tyrosine and tryptophan biosynthesis | 0 | 1 | Linoleic acid metabolism | 5 | 0.008387862 |
| Leukocyte transendothelial migration | 27 | 0.11389 | beta-Alanine metabolism | 0 | 1 | Long-term potentiation | 8 | 0.008650627 |
| mTOR signaling pathway | 34 | 0.11568 | Taurine and hypotaurine metabolism | 0 | 1 | Asthma | 5 | 0.009705683 |
| Alcoholism | 39 | 0.12232 | D-Glutamine and D-glutamate metabolism | 0 | 1 | Cholinergic synapse | 11 | 0.010380265 |
| Notch signaling pathway | 12 | 0.14862 | D-Arginine and D-ornithine metabolism | 0 | 1 | Peroxisome | 9 | 0.011260251 |
| NF-kappa B signaling pathway | 21 | 0.15194 | Glutathione metabolism | 0 | 1 | Cell adhesion molecules (CAMs) | 13 | 0.01127452 |
| Epithelial cell signaling in Helicobacter pylori infection | 16 | 0.15989 | N-Glycan biosynthesis | 0 | 1 | Phospholipase D signaling pathway | 13 | 0.01127452 |
| Tyrosine metabolism | 9 | 0.17154 | Other glycan degradation | 0 | 1 | Carbon metabolism | 11 | 0.011785503 |
| Regulation of autophagy | 10 | 0.17718 | Mucin type O-Glycan biosynthesis | 0 | 1 | Axon guidance | 15 | 0.011943669 |
| Shigellosis | 15 | 0.19007 | Other types of O-glycan biosynthesis | 0 | 1 | Starch and sucrose metabolism | 7 | 0.012786086 |
| Wnt signaling pathway | 30 | 0.20628 | Amino sugar and nucleotide sugar metabolism | 0 | 1 | Thyroid hormone synthesis | 8 | 0.013225157 |
| Viral carcinogenesis | 42 | 0.21582 | Butirosin and neomycin biosynthesis | 0 | 1 | Drug metabolism - other enzymes | 6 | 0.015600627 |
| Porphyrin and chlorophyll metabolism | 10 | 0.22105 | Glycosaminoglycan degradation | 0 | 1 | Renin-angiotensin system | 4 | 0.017522333 |
| Antigen processing and presentation | 17 | 0.22393 | Glycosaminoglycan biosynthesis - chondroitin sulfate / dermatan sulfate | 0 | 1 | Vitamin digestion and absorption | 4 | 0.020306039 |
| AMPK signaling pathway | 26 | 0.22568 | Glycosaminoglycan biosynthesis - keratan sulfate | 0 | 1 | Arginine and proline metabolism | 6 | 0.020841803 |
| ABC transporters | 10 | 0.26866 | Glycosaminoglycan biosynthesis - heparan sulfate / heparin | 0 | 1 | Retinol metabolism | 7 | 0.024904849 |
| Protein digestion and absorption | 19 | 0.27135 | Glycerolipid metabolism | 0 | 1 | Circadian entrainment | 9 | 0.025316321 |
| Staphylococcus aureus infection | 12 | 0.28945 | Inositol phosphate metabolism | 0 | 1 | Maturity onset diabetes of the young | 4 | 0.026649037 |
| Thyroid hormone synthesis | 15 | 0.30198 | Glycosylphosphatidylinositol(GPI)-anchor biosynthesis | 0 | 1 | Hypertrophic cardiomyopathy (HCM) | 8 | 0.031019549 |
| Cell cycle | 25 | 0.3152 | Glycerophospholipid metabolism | 0 | 1 | Dorso-ventral axis formation | 4 | 0.034053233 |
| Huntington's disease | 38 | 0.3176 | Ether lipid metabolism | 0 | 1 | Staphylococcus aureus infection | 6 | 0.034637752 |
| Hedgehog signaling pathway | 10 | 0.31908 | alpha-Linolenic acid metabolism | 0 | 1 | Retrograde endocannabinoid signaling | 9 | 0.035732904 |
| Arginine biosynthesis | 5 | 0.33052 | Sphingolipid metabolism | 0 | 1 | Phenylalanine metabolism | 3 | 0.037457339 |
| Bacterial invasion of epithelial cells | 16 | 0.33806 | Glycosphingolipid biosynthesis - lacto and neolacto series | 0 | 1 | Metabolic pathways | 68 | 0.038456805 |
| Endocrine and other factor-regulated calcium reabsorption | 10 | 0.34502 | Glycosphingolipid biosynthesis - globo series | 0 | 1 | 2-Oxocarboxylic acid metabolism | 3 | 0.043505727 |
| Endocytosis | 50 | 0.35079 | Glycosphingolipid biosynthesis - ganglio series | 0 | 1 | Fatty acid degradation | 5 | 0.044663146 |
| Cytosolic DNA-sensing pathway | 13 | 0.35358 | Pyruvate metabolism | 0 | 1 | Calcium signaling pathway | 13 | 0.056437785 |
| Platelet activation | 24 | 0.36787 | Butanoate metabolism | 0 | 1 | Regulation of actin cytoskeleton | 15 | 0.056823559 |
| Primary bile acid biosynthesis | 4 | 0.3739 | Riboflavin metabolism | 0 | 1 | Bacterial invasion of epithelial cells | 7 | 0.058179287 |
| Axon guidance | 34 | 0.3748 | Vitamin B6 metabolism | 0 | 1 | Complement and coagulation cascades | 7 | 0.061513106 |
| Fanconi anemia pathway | 11 | 0.39016 | Nicotinate and nicotinamide metabolism | 0 | 1 | Alanine, aspartate and glutamate metabolism | 4 | 0.068461712 |
| Parkinson's disease | 27 | 0.43011 | Pantothenate and CoA biosynthesis | 0 | 1 | ECM-receptor interaction | 7 | 0.072203021 |
| Linoleic acid metabolism | 6 | 0.43542 | Biotin metabolism | 0 | 1 | Mineral absorption | 5 | 0.080481896 |
| Glutathione metabolism | 10 | 0.47706 | Lipoic acid metabolism | 0 | 1 | Taurine and hypotaurine metabolism | 2 | 0.083407686 |
| Primary immunodeficiency | 7 | 0.48832 | Folate biosynthesis | 0 | 1 | Tight junction | 10 | 0.091297193 |
| Fc gamma R-mediated phagocytosis | 17 | 0.51175 | Sulfur metabolism | 0 | 1 | GABAergic synapse | 7 | 0.096677354 |
| Phagosome | 28 | 0.51617 | Aminoacyl-tRNA biosynthesis | 0 | 1 | alpha-Linolenic acid metabolism | 3 | 0.097819118 |
| Hippo signaling pathway | 28 | 0.51617 | Biosynthesis of unsaturated fatty acids | 0 | 1 | Alcoholism | 12 | 0.102954044 |
| Riboflavin metabolism | 1 | 0.55161 | 2-Oxocarboxylic acid metabolism | 0 | 1 | Protein digestion and absorption | 7 | 0.105739642 |
| Tight junction | 25 | 0.55725 | Fatty acid metabolism | 0 | 1 | Fatty acid biosynthesis | 2 | 0.111644562 |
| Phototransduction | 5 | 0.55925 | EGFR tyrosine kinase inhibitor resistance | 0 | 1 | Ascorbate and aldarate metabolism | 3 | 0.116712824 |
| Oocyte meiosis | 22 | 0.56948 | Endocrine resistance | 0 | 1 | Adrenergic signaling in cardiomyocytes | 10 | 0.123993843 |
| Ovarian steroidogenesis | 9 | 0.57 | Platinum drug resistance | 0 | 1 | ABC transporters | 4 | 0.130907622 |
| Carbon metabolism | 20 | 0.59017 | Ribosome biogenesis in eukaryotes | 0 | 1 | Pancreatic secretion | 7 | 0.13553134 |
| Protein processing in endoplasmic reticulum | 29 | 0.61061 | Ribosome | 0 | 1 | Pentose phosphate pathway | 3 | 0.136840315 |
| Phenylalanine metabolism | 3 | 0.62033 | RNA transport | 0 | 1 | Hedgehog signaling pathway | 4 | 0.147182266 |
| Nitrogen metabolism | 3 | 0.62033 | mRNA surveillance pathway | 0 | 1 | beta-Alanine metabolism | 3 | 0.158046926 |
| Ubiquinone and other terpenoid-quinone biosynthesis | 2 | 0.62079 | RNA degradation | 0 | 1 | Valine, leucine and isoleucine degradation | 4 | 0.164192388 |
| Alanine, aspartate and glutamate metabolism | 6 | 0.63107 | RNA polymerase | 0 | 1 | Riboflavin metabolism | 1 | 0.166579651 |
| Butirosin and neomycin biosynthesis | 1 | 0.6331 | Basal transcription factors | 0 | 1 | Propanoate metabolism | 3 | 0.16900647 |
| Vitamin digestion and absorption | 4 | 0.65803 | DNA replication | 0 | 1 | Base excision repair | 3 | 0.180177915 |
| 2-Oxocarboxylic acid metabolism | 3 | 0.66067 | Spliceosome | 0 | 1 | Endocytosis | 15 | 0.183794634 |
| Arginine and proline metabolism | 8 | 0.68809 | Proteasome | 0 | 1 | Other glycan degradation | 2 | 0.189934245 |
| Phosphatidylinositol signaling system | 16 | 0.72188 | Protein export | 0 | 1 | Protein processing in endoplasmic reticulum | 10 | 0.200577775 |
| Biosynthesis of amino acids | 12 | 0.73215 | PPAR signaling pathway | 0 | 1 | Butirosin and neomycin biosynthesis | 1 | 0.203702694 |
| Cysteine and methionine metabolism | 7 | 0.73365 | Base excision repair | 0 | 1 | Phenylalanine, tyrosine and tryptophan biosynthesis | 1 | 0.203702694 |
| Glycosphingolipid biosynthesis - globo series | 2 | 0.75209 | Nucleotide excision repair | 0 | 1 | Dilated cardiomyopathy | 6 | 0.204626938 |
| Bile secretion | 11 | 0.76598 | Mismatch repair | 0 | 1 | Inositol phosphate metabolism | 5 | 0.208553116 |
| Glyoxylate and dicarboxylate metabolism | 4 | 0.77535 | Homologous recombination | 0 | 1 | Pentose and glucuronate interconversions | 3 | 0.214776083 |
| Pentose phosphate pathway | 4 | 0.79912 | Non-homologous end-joining | 0 | 1 | Primary immunodeficiency | 3 | 0.214776083 |
| Pathogenic Escherichia coli infection | 8 | 0.80656 | Fanconi anemia pathway | 0 | 1 | Steroid biosynthesis | 2 | 0.223000703 |
| Basal cell carcinoma | 8 | 0.80656 | ErbB signaling pathway | 0 | 1 | Basal cell carcinoma | 4 | 0.228494642 |
| Protein export | 3 | 0.81568 | Cytokine-cytokine receptor interaction | 0 | 1 | Gastric acid secretion | 5 | 0.232993237 |
| Arachidonic acid metabolism | 9 | 0.81802 | Chemokine signaling pathway | 0 | 1 | Glutamatergic synapse | 7 | 0.24501026 |
| Drug metabolism - cytochrome P450 | 10 | 0.82859 | FoxO signaling pathway | 0 | 1 | Systemic lupus erythematosus | 8 | 0.247155469 |
| Vasopressin-regulated water reabsorption | 6 | 0.83568 | Cell cycle | 0 | 1 | Phosphatidylinositol signaling system | 6 | 0.270903393 |
| Histidine metabolism | 3 | 0.8382 | p53 signaling pathway | 0 | 1 | Biosynthesis of unsaturated fatty acids | 2 | 0.273237362 |
| Galactose metabolism | 4 | 0.84053 | Ubiquitin mediated proteolysis | 0 | 1 | Mismatch repair | 2 | 0.273237362 |
| beta-Alanine metabolism | 4 | 0.84053 | Sulfur relay system | 0 | 1 | Porphyrin and chlorophyll metabolism | 3 | 0.287256149 |
| Lysine degradation | 7 | 0.85776 | SNARE interactions in vesicular transport | 0 | 1 | Histidine metabolism | 2 | 0.289996902 |
| Other glycan degradation | 2 | 0.86491 | Regulation of autophagy | 0 | 1 | Proteasome | 3 | 0.311911492 |
| Pantothenate and CoA biosynthesis | 2 | 0.86491 | Protein processing in endoplasmic reticulum | 0 | 1 | Cysteine and methionine metabolism | 3 | 0.324265975 |
| Sulfur metabolism | 1 | 0.86545 | Lysosome | 0 | 1 | Phototransduction | 2 | 0.339872154 |
| Carbohydrate digestion and absorption | 6 | 0.86583 | Endocytosis | 0 | 1 | Glycolysis / Gluconeogenesis | 4 | 0.34883931 |
| Base excision repair | 4 | 0.87451 | Phagosome | 0 | 1 | Endocrine and other factor-regulated calcium reabsorption | 3 | 0.348962989 |
| Lysosome | 18 | 0.87472 | mTOR signaling pathway | 0 | 1 | Sphingolipid metabolism | 3 | 0.348962989 |
| Glycine, serine and threonine metabolism | 5 | 0.876 | Apoptosis | 0 | 1 | Butanoate metabolism | 2 | 0.356275245 |
| Maturity onset diabetes of the young | 3 | 0.87618 | Longevity regulating pathway | 0 | 1 | Glyoxylate and dicarboxylate metabolism | 2 | 0.356275245 |
| Glycosaminoglycan degradation | 2 | 0.88457 | Longevity regulating pathway - multiple species | 0 | 1 | Salivary secretion | 5 | 0.363622501 |
| Collecting duct acid secretion | 3 | 0.89203 | Apoptosis - multiple species | 0 | 1 | Phagosome | 8 | 0.372430677 |
| Chemical carcinogenesis | 11 | 0.90095 | Wnt signaling pathway | 0 | 1 | Circadian rhythm | 2 | 0.388616202 |
| Steroid biosynthesis | 2 | 0.90155 | Dorso-ventral axis formation | 0 | 1 | Citrate cycle (TCA cycle) | 2 | 0.388616202 |
| One carbon pool by folate | 2 | 0.90155 | Notch signaling pathway | 0 | 1 | Biosynthesis of amino acids | 4 | 0.430283825 |
| Butanoate metabolism | 3 | 0.90603 | Hedgehog signaling pathway | 0 | 1 | Fructose and mannose metabolism | 2 | 0.435704743 |
| Hippo signaling pathway -multiple species | 3 | 0.90603 | TGF-beta signaling pathway | 0 | 1 | Pathogenic Escherichia coli infection | 3 | 0.445942102 |
| PPAR signaling pathway | 9 | 0.92574 | Axon guidance | 0 | 1 | Antigen processing and presentation | 4 | 0.450294553 |
| Folate biosynthesis | 1 | 0.93972 | Osteoclast differentiation | 0 | 1 | Folate biosynthesis | 1 | 0.471691416 |
| Sphingolipid metabolism | 5 | 0.94579 | Hippo signaling pathway | 0 | 1 | Glycerolipid metabolism | 3 | 0.492318883 |
| Pyruvate metabolism | 4 | 0.94886 | Hippo signaling pathway -multiple species | 0 | 1 | Glycosaminoglycan biosynthesis - keratan sulfate | 1 | 0.495246576 |
| Glycosphingolipid biosynthesis - ganglio series | 1 | 0.95069 | Focal adhesion | 0 | 1 | Glycosphingolipid biosynthesis - ganglio series | 1 | 0.495246576 |
| Synaptic vesicle cycle | 7 | 0.95497 | ECM-receptor interaction | 0 | 1 | Glycine, serine and threonine metabolism | 2 | 0.537272565 |
| alpha-Linolenic acid metabolism | 2 | 0.95658 | Cell adhesion molecules (CAMs) | 0 | 1 | Pyruvate metabolism | 2 | 0.537272565 |
| Systemic lupus erythematosus | 17 | 0.96632 | Adherens junction | 0 | 1 | Regulation of autophagy | 2 | 0.537272565 |
| Selenocompound metabolism | 1 | 0.967 | Tight junction | 0 | 1 | Selenocompound metabolism | 1 | 0.539259257 |
| Metabolism of xenobiotics by cytochrome P450 | 8 | 0.96731 | Signaling pathways regulating pluripotency of stem cells | 0 | 1 | Glycosaminoglycan degradation | 1 | 0.579441771 |
| Proteasome | 4 | 0.9704 | Complement and coagulation cascades | 0 | 1 | Morphine addiction | 4 | 0.582096377 |
| Ether lipid metabolism | 4 | 0.97427 | Antigen processing and presentation | 0 | 1 | Vasopressin-regulated water reabsorption | 2 | 0.589403917 |
| Peroxisome | 9 | 0.97613 | Toll-like receptor signaling pathway | 0 | 1 | Ether lipid metabolism | 2 | 0.601735914 |
| Drug metabolism - other enzymes | 4 | 0.97766 | NOD-like receptor signaling pathway | 0 | 1 | Parkinson's disease | 6 | 0.610590032 |
| Nicotinate and nicotinamide metabolism | 2 | 0.97793 | RIG-I-like receptor signaling pathway | 0 | 1 | Terpenoid backbone biosynthesis | 1 | 0.633253711 |
| Oxidative phosphorylation | 16 | 0.97948 | Cytosolic DNA-sensing pathway | 0 | 1 | Amino sugar and nucleotide sugar metabolism | 2 | 0.637046786 |
| Glycosaminoglycan biosynthesis - chondroitin sulfate / dermatan sulfate | 1 | 0.98194 | Jak-STAT signaling pathway | 0 | 1 | Arrhythmogenic right ventricular cardiomyopathy (ARVC) | 3 | 0.646406789 |
| Inositol phosphate metabolism | 7 | 0.98234 | Hematopoietic cell lineage | 0 | 1 | Proximal tubule bicarbonate reclamation | 1 | 0.649618091 |
| Starch and sucrose metabolism | 5 | 0.98526 | Natural killer cell mediated cytotoxicity | 0 | 1 | Glycosaminoglycan biosynthesis - heparan sulfate / heparin | 1 | 0.665253794 |
| Glycerophospholipid metabolism | 10 | 0.98592 | T cell receptor signaling pathway | 0 | 1 | Fatty acid elongation | 1 | 0.680193202 |
| Steroid hormone biosynthesis | 5 | 0.98715 | B cell receptor signaling pathway | 0 | 1 | Glycosphingolipid biosynthesis - lacto and neolacto series | 1 | 0.694467257 |
| Terpenoid backbone biosynthesis | 1 | 0.98791 | Fc epsilon RI signaling pathway | 0 | 1 | Neuroactive ligand-receptor interaction | 11 | 0.695136108 |
| Fructose and mannose metabolism | 2 | 0.98894 | Fc gamma R-mediated phagocytosis | 0 | 1 | Homologous recombination | 1 | 0.733586517 |
| Proximal tubule bicarbonate reclamation | 1 | 0.99011 | Leukocyte transendothelial migration | 0 | 1 | Aminoacyl-tRNA biosynthesis | 2 | 0.799274116 |
| SNARE interactions in vesicular transport | 2 | 0.99072 | Intestinal immune network for IgA production | 0 | 1 | Glycerophospholipid metabolism | 3 | 0.801584092 |
| Glycosaminoglycan biosynthesis - heparan sulfate / heparin | 1 | 0.99191 | Circadian rhythm | 0 | 1 | Lysosome | 4 | 0.803742871 |
| Amino sugar and nucleotide sugar metabolism | 3 | 0.99562 | Long-term depression | 0 | 1 | DNA replication | 1 | 0.806513549 |
| Glycolysis / Gluconeogenesis | 5 | 0.9964 | Progesterone-mediated oocyte maturation | 0 | 1 | Ubiquitin mediated proteolysis | 4 | 0.865148264 |
| Glycerolipid metabolism | 4 | 0.99675 | Prolactin signaling pathway | 0 | 1 | Nucleotide excision repair | 1 | 0.883002723 |
| Fat digestion and absorption | 2 | 0.99732 | Thyroid hormone synthesis | 0 | 1 | Notch signaling pathway | 1 | 0.888235828 |
| Vibrio cholerae infection | 3 | 0.99732 | Adipocytokine signaling pathway | 0 | 1 | Vibrio cholerae infection | 1 | 0.902574792 |
| Other types of O-glycan biosynthesis | 1 | 0.99802 | Non-alcoholic fatty liver disease (NAFLD) | 0 | 1 | Lysine degradation | 1 | 0.906934157 |
| RNA polymerase | 1 | 0.99802 | Type I diabetes mellitus | 0 | 1 | Fanconi anemia pathway | 1 | 0.915077583 |
| Propanoate metabolism | 1 | 0.99838 | Maturity onset diabetes of the young | 0 | 1 | Ribosome | 3 | 0.945446188 |
| Valine, leucine and isoleucine degradation | 2 | 0.99924 | Endocrine and other factor-regulated calcium reabsorption | 0 | 1 | Pyrimidine metabolism | 2 | 0.949544321 |
| Pentose and glucuronate interconversions | 1 | 0.99928 | Vasopressin-regulated water reabsorption | 0 | 1 | Purine metabolism | 4 | 0.954602795 |
| Ubiquitin mediated proteolysis | 12 | 0.9994 | Fat digestion and absorption | 0 | 1 | RNA degradation | 1 | 0.970411482 |
| Retinol metabolism | 3 | 0.99975 | Mineral absorption | 0 | 1 | Ribosome biogenesis in eukaryotes | 1 | 0.977535375 |
| Fatty acid degradation | 1 | 0.99986 | Prion diseases | 0 | 1 | Taste transduction | 1 | 0.977535375 |
| Basal transcription factors | 1 | 0.99988 | Bacterial invasion of epithelial cells | 0 | 1 | mRNA surveillance pathway | 1 | 0.984444305 |
| Purine metabolism | 15 | 0.99989 | Epithelial cell signaling in Helicobacter pylori infection | 0 | 1 | RNA transport | 2 | 0.996466706 |
| Nucleotide excision repair | 1 | 0.99992 | Pathogenic Escherichia coli infection | 0 | 1 | Oxidative phosphorylation | 1 | 0.99775173 |
| Fatty acid metabolism | 1 | 0.99994 | Shigellosis | 0 | 1 | Spliceosome | 1 | 0.997853136 |
| N-Glycan biosynthesis | 1 | 0.99995 | Legionellosis | 0 | 1 | Olfactory transduction | 1 | 0.999999996 |
| Aminoacyl-tRNA biosynthesis | 2 | 0.99997 | African trypanosomiasis | 0 | 1 | Basal transcription factors | 0 | 1 |
| mRNA surveillance pathway | 4 | 0.99998 | Malaria | 0 | 1 | Biotin metabolism | 0 | 1 |
| RNA transport | 12 | 0.99999 | Staphylococcus aureus infection | 0 | 1 | Cardiac muscle contraction | 0 | 1 |
| RNA degradation | 2 | 1 | Hepatitis C | 0 | 1 | Collecting duct acid secretion | 0 | 1 |
| Pyrimidine metabolism | 4 | 1 | Hepatitis B | 0 | 1 | D-Arginine and D-ornithine metabolism | 0 | 1 |
| Spliceosome | 5 | 1 | Measles | 0 | 1 | D-Glutamine and D-glutamate metabolism | 0 | 1 |
| Ribosome | 5 | 1 | Influenza A | 0 | 1 | Glycosaminoglycan biosynthesis - chondroitin sulfate / dermatan sulfate | 0 | 1 |
| Olfactory transduction | 11 | 1 | Herpes simplex infection | 0 | 1 | Glycosphingolipid biosynthesis - globo series | 0 | 1 |
| Metabolic pathways | 114 | 1 | Epstein-Barr virus infection | 0 | 1 | Glycosylphosphatidylinositol(GPI)-anchor biosynthesis | 0 | 1 |
| Ascorbate and aldarate metabolism | 0 | 1 | Transcriptional misregulation in cancer | 0 | 1 | Hippo signaling pathway -multiple species | 0 | 1 |
| Fatty acid biosynthesis | 0 | 1 | Viral carcinogenesis | 0 | 1 | Lipoic acid metabolism | 0 | 1 |
| Fatty acid elongation | 0 | 1 | Proteoglycans in cancer | 0 | 1 | Lysine biosynthesis | 0 | 1 |
| Synthesis and degradation of ketone bodies | 0 | 1 | Colorectal cancer | 0 | 1 | Mucin type O-Glycan biosynthesis | 0 | 1 |
| Valine, leucine and isoleucine biosynthesis | 0 | 1 | Renal cell carcinoma | 0 | 1 | N-Glycan biosynthesis | 0 | 1 |
| Lysine biosynthesis | 0 | 1 | Pancreatic cancer | 0 | 1 | Nicotinate and nicotinamide metabolism | 0 | 1 |
| Phenylalanine, tyrosine and tryptophan biosynthesis | 0 | 1 | Endometrial cancer | 0 | 1 | Nicotine addiction | 0 | 1 |
| D-Glutamine and D-glutamate metabolism | 0 | 1 | Prostate cancer | 0 | 1 | Nitrogen metabolism | 0 | 1 |
| D-Arginine and D-ornithine metabolism | 0 | 1 | Thyroid cancer | 0 | 1 | Non-homologous end-joining | 0 | 1 |
| Mucin type O-Glycan biosynthesis | 0 | 1 | Basal cell carcinoma | 0 | 1 | One carbon pool by folate | 0 | 1 |
| Glycosaminoglycan biosynthesis - keratan sulfate | 0 | 1 | Melanoma | 0 | 1 | Other types of O-glycan biosynthesis | 0 | 1 |
| Glycosylphosphatidylinositol(GPI)-anchor biosynthesis | 0 | 1 | Bladder cancer | 0 | 1 | Pantothenate and CoA biosynthesis | 0 | 1 |
| Glycosphingolipid biosynthesis - lacto and neolacto series | 0 | 1 | Chronic myeloid leukemia | 0 | 1 | Primary bile acid biosynthesis | 0 | 1 |
| Thiamine metabolism | 0 | 1 | Acute myeloid leukemia | 0 | 1 | Protein export | 0 | 1 |
| Vitamin B6 metabolism | 0 | 1 | Non-small cell lung cancer | 0 | 1 | RNA polymerase | 0 | 1 |
| Biotin metabolism | 0 | 1 | Central carbon metabolism in cancer | 0 | 1 | SNARE interactions in vesicular transport | 0 | 1 |
| Lipoic acid metabolism | 0 | 1 | Asthma | 0 | 1 | Sulfur metabolism | 0 | 1 |
| Biosynthesis of unsaturated fatty acids | 0 | 1 | Autoimmune thyroid disease | 0 | 1 | Sulfur relay system | 0 | 1 |
| Ribosome biogenesis in eukaryotes | 0 | 1 | Inflammatory bowel disease (IBD) | 0 | 1 | Synaptic vesicle cycle | 0 | 1 |
| DNA replication | 0 | 1 | Rheumatoid arthritis | 0 | 1 | Synthesis and degradation of ketone bodies | 0 | 1 |
| Mismatch repair | 0 | 1 | Allograft rejection | 0 | 1 | Thiamine metabolism | 0 | 1 |
| Homologous recombination | 0 | 1 | Graft-versus-host disease | 0 | 1 | Ubiquinone and other terpenoid-quinone biosynthesis | 0 | 1 |
| Non-homologous end-joining | 0 | 1 | Primary immunodeficiency | 0 | 1 | Valine, leucine and isoleucine biosynthesis | 0 | 1 |
| Sulfur relay system | 0 | 1 | Viral myocarditis | 0 | 1 | Vitamin B6 metabolism | 0 | 1 |

Pathways in M2

| **Symptom** | **overlap** | **p_value** | **Drug** | **overlap** | **p_value** | **Herb** | **overlap** | **p_value** |
| --- | --- | --- | --- | --- | --- | --- | --- | --- |
| Malaria | 22 | 4.63809E-10 | Neuroactive ligand-receptor interaction | 54 | 8.3891E-43 | Pathways in cancer | 86 | 5.73779E-41 |
| AGE-RAGE signaling pathway in diabetic complications | 33 | 6.33968E-10 | Calcium signaling pathway | 33 | 4.264E-25 | Hepatitis B | 54 | 8.48284E-39 |
| Hypertrophic cardiomyopathy (HCM) | 29 | 1.17581E-09 | Nicotine addiction | 18 | 6.28069E-22 | AGE-RAGE signaling pathway in diabetic complications | 46 | 4.4129E-38 |
| Proteoglycans in cancer | 50 | 3.32868E-09 | GABAergic synapse | 23 | 2.20909E-21 | PI3K-Akt signaling pathway | 69 | 8.0513E-31 |
| Viral myocarditis | 23 | 3.67205E-09 | Serotonergic synapse | 24 | 4.71771E-20 | Apoptosis | 45 | 2.43923E-29 |
| HIF-1 signaling pathway | 31 | 1.91482E-08 | Retrograde endocannabinoid signaling | 23 | 6.7615E-20 | Pancreatic cancer | 31 | 1.86383E-26 |
| Amyotrophic lateral sclerosis (ALS) | 19 | 2.71595E-07 | Hypertrophic cardiomyopathy (HCM) | 19 | 1.18247E-16 | FoxO signaling pathway | 41 | 5.41437E-26 |
| Hematopoietic cell lineage | 26 | 3.10774E-07 | Morphine addiction | 19 | 7.45642E-16 | Toxoplasmosis | 37 | 7.72192E-24 |
| Amoebiasis | 28 | 3.92702E-07 | Oxytocin signaling pathway | 22 | 2.3114E-14 | Chagas disease (American trypanosomiasis) | 35 | 8.91011E-24 |
| Dilated cardiomyopathy | 26 | 5.09231E-07 | cAMP signaling pathway | 22 | 3.21197E-12 | Tuberculosis | 44 | 1.4396E-23 |
| ECM-receptor interaction | 23 | 4.94182E-06 | Adrenergic signaling in cardiomyocytes | 19 | 7.43897E-12 | Colorectal cancer | 28 | 2.10153E-23 |
| Focal adhesion | 42 | 6.47117E-06 | Type II diabetes mellitus | 12 | 1.94858E-11 | Platinum drug resistance | 30 | 4.30117E-23 |
| Rheumatoid arthritis | 24 | 6.55462E-06 | MAPK signaling pathway | 23 | 6.8134E-11 | Prostate cancer | 32 | 7.45908E-23 |
| Arrhythmogenic right ventricular cardiomyopathy (ARVC) | 21 | 1.03706E-05 | Taste transduction | 14 | 1.16455E-10 | Small cell lung cancer | 31 | 3.27127E-22 |
| African trypanosomiasis | 13 | 1.52498E-05 | Insulin resistance | 15 | 4.90147E-10 | Focal adhesion | 44 | 5.33737E-21 |
| Toxoplasmosis | 28 | 1.65718E-05 | Adipocytokine signaling pathway | 12 | 2.14009E-09 | HTLV-I infection | 49 | 1.17503E-20 |
| Bladder cancer | 14 | 3.22184E-05 | Dilated cardiomyopathy | 13 | 3.47815E-09 | Endocrine resistance | 31 | 2.03306E-20 |
| PI3K-Akt signaling pathway | 59 | 3.28762E-05 | cGMP-PKG signaling pathway | 17 | 3.87205E-09 | Leishmaniasis | 27 | 5.10855E-20 |
| Legionellosis | 16 | 8.32631E-05 | Arrhythmogenic right ventricular cardiomyopathy (ARVC) | 12 | 4.15747E-09 | TNF signaling pathway | 32 | 1.20035E-19 |
| Apoptosis | 29 | 0.000165506 | Cardiac muscle contraction | 12 | 7.7498E-09 | Chronic myeloid leukemia | 26 | 1.16498E-18 |
| Type I diabetes mellitus | 13 | 0.000249896 | Longevity regulating pathway | 12 | 6.70553E-08 | HIF-1 signaling pathway | 30 | 1.6625E-18 |
| Leishmaniasis | 18 | 0.000260803 | Complement and coagulation cascades | 11 | 9.67186E-08 | EGFR tyrosine kinase inhibitor resistance | 27 | 1.8058E-18 |
| TNF signaling pathway | 24 | 0.000261571 | Renin secretion | 10 | 1.22885E-07 | Proteoglycans in cancer | 41 | 2.84293E-18 |
| Thyroid cancer | 10 | 0.000398338 | AMPK signaling pathway | 13 | 1.82486E-07 | Toll-like receptor signaling pathway | 30 | 4.09115E-18 |
| Inflammatory bowel disease (IBD) | 16 | 0.000678556 | Circadian rhythm | 7 | 5.88845E-07 | NF-kappa B signaling pathway | 28 | 6.48947E-18 |
| Cytokine-cytokine receptor interaction | 44 | 0.000844246 | Insulin signaling pathway | 13 | 7.60986E-07 | Bladder cancer | 20 | 6.84439E-18 |
| Allograft rejection | 11 | 0.000867699 | Aldosterone synthesis and secretion | 10 | 1.18312E-06 | Measles | 33 | 1.11707E-17 |
| Salmonella infection | 19 | 0.000942431 | Glucagon signaling pathway | 11 | 1.22462E-06 | Malaria | 21 | 2.89641E-17 |
| EGFR tyrosine kinase inhibitor resistance | 18 | 0.001183084 | Longevity regulating pathway - multiple species | 9 | 1.34792E-06 | Pertussis | 25 | 3.43933E-17 |
| Colorectal cancer | 15 | 0.001196581 | Cholinergic synapse | 11 | 3.14063E-06 | MAPK signaling pathway | 44 | 5.65963E-17 |
| Dorso-ventral axis formation | 9 | 0.001378181 | Steroid hormone biosynthesis | 8 | 6.15996E-06 | Glioma | 23 | 1.4233E-16 |
| Phagosome | 28 | 0.001665321 | Vascular smooth muscle contraction | 11 | 6.73481E-06 | p53 signaling pathway | 23 | 6.51541E-16 |
| Hepatitis B | 27 | 0.001705771 | Carbohydrate digestion and absorption | 7 | 1.22665E-05 | Apoptosis - multiple species | 17 | 6.57633E-16 |
| Chagas disease (American trypanosomiasis) | 21 | 0.001766512 | Dopaminergic synapse | 11 | 1.34972E-05 | Insulin resistance | 28 | 8.87842E-16 |
| Pathways in cancer | 59 | 0.002098465 | Insulin secretion | 9 | 1.47703E-05 | Adipocytokine signaling pathway | 23 | 9.35582E-16 |
| Type II diabetes mellitus | 12 | 0.002687593 | FoxO signaling pathway | 11 | 1.80347E-05 | Influenza A | 35 | 9.50911E-16 |
| Influenza A | 30 | 0.003285261 | Salivary secretion | 9 | 2.14937E-05 | Osteoclast differentiation | 30 | 2.43717E-15 |
| Tuberculosis | 30 | 0.003904115 | Non-alcoholic fatty liver disease (NAFLD) | 11 | 5.86853E-05 | Non-alcoholic fatty liver disease (NAFLD) | 32 | 2.9926E-15 |
| Thyroid hormone signaling pathway | 22 | 0.003953157 | Prostate cancer | 8 | 0.000144096 | Non-small cell lung cancer | 20 | 1.15511E-14 |
| Mineral absorption | 12 | 0.005430304 | Galactose metabolism | 5 | 0.000170596 | Transcriptional misregulation in cancer | 34 | 1.2574E-14 |
| Chronic myeloid leukemia | 15 | 0.006423742 | Regulation of lipolysis in adipocytes | 6 | 0.000384864 | Prolactin signaling pathway | 22 | 2.2353E-14 |
| Renin-angiotensin system | 7 | 0.006562552 | Starch and sucrose metabolism | 6 | 0.000424065 | T cell receptor signaling pathway | 26 | 2.53633E-14 |
| Intestinal immune network for IgA production | 11 | 0.006819459 | Pathways in cancer | 17 | 0.000498311 | Rheumatoid arthritis | 24 | 3.26739E-14 |
| Graft-versus-host disease | 10 | 0.007158393 | Amphetamine addiction | 6 | 0.001011777 | Amoebiasis | 25 | 4.95462E-14 |
| Pantothenate and CoA biosynthesis | 6 | 0.007199555 | Cocaine addiction | 5 | 0.001490481 | Hepatitis C | 28 | 2.0445E-13 |
| Prion diseases | 9 | 0.007317619 | Butirosin and neomycin biosynthesis | 2 | 0.002860031 | Inflammatory bowel disease (IBD) | 20 | 2.9994E-13 |
| Leukocyte transendothelial migration | 21 | 0.008282671 | Small cell lung cancer | 6 | 0.003643223 | VEGF signaling pathway | 19 | 9.29348E-13 |
| Cell adhesion molecules (CAMs) | 24 | 0.010377957 | Gap junction | 6 | 0.004082634 | Cell cycle | 26 | 1.67571E-12 |
| Longevity regulating pathway - multiple species | 13 | 0.01185711 | Neurotrophin signaling pathway | 7 | 0.004689018 | Rap1 signaling pathway | 34 | 1.80896E-12 |
| Small cell lung cancer | 16 | 0.013094533 | GnRH signaling pathway | 6 | 0.004813711 | Melanoma | 20 | 1.91583E-12 |
| Glioma | 13 | 0.013472513 | Platelet activation | 7 | 0.005131007 | MicroRNAs in cancer | 41 | 2.08936E-12 |
| Autoimmune thyroid disease | 11 | 0.014749864 | Central carbon metabolism in cancer | 5 | 0.005886963 | Thyroid hormone signaling pathway | 25 | 3.55365E-12 |
| Endometrial cancer | 11 | 0.014749864 | Circadian entrainment | 6 | 0.005932824 | Ras signaling pathway | 35 | 3.58215E-12 |
| Arginine biosynthesis | 6 | 0.016120786 | Bile secretion | 5 | 0.007511743 | African trypanosomiasis | 14 | 1.22827E-11 |
| Endocrine resistance | 17 | 0.018973372 | Chronic myeloid leukemia | 5 | 0.008431097 | Sphingolipid signaling pathway | 24 | 3.54878E-11 |
| Platinum drug resistance | 14 | 0.018987501 | Apoptosis | 7 | 0.010641695 | Epstein-Barr virus infection | 31 | 5.02319E-11 |
| Protein digestion and absorption | 16 | 0.019767241 | Hepatitis B | 7 | 0.01319143 | Salmonella infection | 20 | 8.99186E-11 |
| MicroRNAs in cancer | 42 | 0.021336349 | NF-kappa B signaling pathway | 5 | 0.021289898 | ErbB signaling pathway | 20 | 1.40459E-10 |
| p53 signaling pathway | 13 | 0.02167089 | Glioma | 4 | 0.025658289 | Neurotrophin signaling pathway | 23 | 2.26091E-10 |
| Antigen processing and presentation | 14 | 0.023502537 | Osteoclast differentiation | 6 | 0.025790512 | Type II diabetes mellitus | 15 | 2.29502E-10 |
| NF-kappa B signaling pathway | 16 | 0.023949319 | Pancreatic cancer | 4 | 0.026957331 | Cytokine-cytokine receptor interaction | 35 | 2.69698E-10 |
| Porphyrin and chlorophyll metabolism | 9 | 0.024168989 | Inflammatory mediator regulation of TRP channels | 5 | 0.027097809 | NOD-like receptor signaling pathway | 16 | 3.45623E-10 |
| Non-small cell lung cancer | 11 | 0.024975892 | Epithelial cell signaling in Helicobacter pylori infection | 4 | 0.029671868 | Viral myocarditis | 16 | 4.60239E-10 |
| Complement and coagulation cascades | 14 | 0.028773064 | HIF-1 signaling pathway | 5 | 0.032641653 | Jak-STAT signaling pathway | 26 | 5.02149E-10 |
| Asthma | 7 | 0.02898237 | B cell receptor signaling pathway | 4 | 0.035570952 | Fc epsilon RI signaling pathway | 17 | 5.50274E-10 |
| Regulation of actin cytoskeleton | 31 | 0.031761701 | Leishmaniasis | 4 | 0.035570952 | Amyotrophic lateral sclerosis (ALS) | 15 | 6.00291E-10 |
| Jak-STAT signaling pathway | 24 | 0.031901353 | Gastric acid secretion | 4 | 0.038757162 | Herpes simplex infection | 28 | 7.83444E-10 |
| cGMP-PKG signaling pathway | 25 | 0.033887427 | TNF signaling pathway | 5 | 0.041516599 | Endometrial cancer | 15 | 8.13571E-10 |
| Prostate cancer | 15 | 0.036233241 | mTOR signaling pathway | 6 | 0.049107689 | Legionellosis | 15 | 1.93742E-09 |
| T cell receptor signaling pathway | 17 | 0.038060746 | Ovarian steroidogenesis | 3 | 0.054841585 | Viral carcinogenesis | 29 | 1.95988E-09 |
| Long-term depression | 11 | 0.039565565 | Epstein-Barr virus infection | 7 | 0.058045311 | Allograft rejection | 12 | 9.32805E-09 |
| Toll-like receptor signaling pathway | 17 | 0.041179861 | Oocyte meiosis | 5 | 0.061538114 | Platelet activation | 21 | 1.05676E-08 |
| Aldosterone-regulated sodium reabsorption | 8 | 0.041310223 | Legionellosis | 3 | 0.069038196 | Choline metabolism in cancer | 19 | 1.18492E-08 |
| Transcriptional misregulation in cancer | 26 | 0.042800233 | PI3K-Akt signaling pathway | 10 | 0.069895914 | AMPK signaling pathway | 21 | 1.22863E-08 |
| Estrogen signaling pathway | 16 | 0.043892851 | Alzheimer's disease | 6 | 0.07067997 | GnRH signaling pathway | 18 | 1.26253E-08 |
| VEGF signaling pathway | 11 | 0.043981308 | Maturity onset diabetes of the young | 2 | 0.073421285 | B cell receptor signaling pathway | 16 | 1.42417E-08 |
| TGF-beta signaling pathway | 14 | 0.045659702 | NOD-like receptor signaling pathway | 3 | 0.07513816 | Insulin signaling pathway | 22 | 2.32536E-08 |
| Staphylococcus aureus infection | 10 | 0.050776011 | HTLV-I infection | 8 | 0.078250125 | Oxytocin signaling pathway | 23 | 4.95017E-08 |
| FoxO signaling pathway | 20 | 0.051434071 | Collecting duct acid secretion | 2 | 0.078423523 | Estrogen signaling pathway | 18 | 5.02109E-08 |
| Natural killer cell mediated cytotoxicity | 20 | 0.054889485 | Pancreatic secretion | 4 | 0.084131636 | Type I diabetes mellitus | 12 | 6.22527E-08 |
| Melanoma | 12 | 0.056005825 | Endocrine resistance | 4 | 0.086629051 | TGF-beta signaling pathway | 16 | 1.42909E-07 |
| Measles | 20 | 0.058505379 | Linoleic acid metabolism | 2 | 0.088733633 | Hematopoietic cell lineage | 16 | 2.37681E-07 |
| Alzheimer's disease | 24 | 0.058755505 | Thyroid cancer | 2 | 0.088733633 | Acute myeloid leukemia | 13 | 2.37691E-07 |
| ErbB signaling pathway | 14 | 0.063421964 | Arachidonic acid metabolism | 3 | 0.09137844 | Renal cell carcinoma | 14 | 2.61492E-07 |
| Calcium signaling pathway | 25 | 0.067843477 | Colorectal cancer | 3 | 0.09137844 | Central carbon metabolism in cancer | 14 | 2.61492E-07 |
| Primary immunodeficiency | 7 | 0.069736316 | Cytosolic DNA-sensing pathway | 3 | 0.094788578 | Ovarian steroidogenesis | 12 | 3.83205E-07 |
| Pancreatic cancer | 11 | 0.071105304 | Vitamin B6 metabolism | 1 | 0.099168192 | Chemical carcinogenesis | 15 | 6.0978E-07 |
| Inflammatory mediator regulation of TRP channels | 15 | 0.073822123 | Ras signaling pathway | 7 | 0.099701332 | Leukocyte transendothelial migration | 18 | 7.83908E-07 |
| Central carbon metabolism in cancer | 11 | 0.077580466 | Shigellosis | 3 | 0.101762941 | cAMP signaling pathway | 24 | 1.02835E-06 |
| Fc epsilon RI signaling pathway | 11 | 0.077580466 | Chagas disease (American trypanosomiasis) | 4 | 0.105095152 | Thyroid cancer | 9 | 1.06572E-06 |
| Renal cell carcinoma | 11 | 0.077580466 | Long-term potentiation | 3 | 0.105325113 | Shigellosis | 13 | 1.19522E-06 |
| Pertussis | 12 | 0.078767263 | T cell receptor signaling pathway | 4 | 0.107869481 | Natural killer cell mediated cytotoxicity | 19 | 1.22286E-06 |
| Oxytocin signaling pathway | 22 | 0.080380641 | Toll-like receptor signaling pathway | 4 | 0.110676749 | Inflammatory mediator regulation of TRP channels | 16 | 1.27604E-06 |
| Longevity regulating pathway | 14 | 0.097841419 | RIG-I-like receptor signaling pathway | 3 | 0.120049359 | Intestinal immune network for IgA production | 11 | 1.54515E-06 |
| D-Arginine and D-ornithine metabolism | 1 | 0.102544333 | Melanoma | 3 | 0.123844016 | Amphetamine addiction | 13 | 1.71848E-06 |
| Neurotrophin signaling pathway | 17 | 0.105416427 | Prolactin signaling pathway | 3 | 0.127681976 | Epithelial cell signaling in Helicobacter pylori infection | 13 | 2.0496E-06 |
| Sphingolipid signaling pathway | 17 | 0.105416427 | Metabolism of xenobiotics by cytochrome P450 | 3 | 0.131562163 | Hippo signaling pathway | 20 | 2.36436E-06 |
| Adrenergic signaling in cardiomyocytes | 20 | 0.12106808 | Pertussis | 3 | 0.139444912 | Drug metabolism - cytochrome P450 | 13 | 2.43618E-06 |
| Osteoclast differentiation | 18 | 0.121389561 | Viral carcinogenesis | 6 | 0.14354054 | Graft-versus-host disease | 10 | 3.10429E-06 |
| Acute myeloid leukemia | 9 | 0.124958845 | Aldosterone-regulated sodium reabsorption | 2 | 0.145189509 | Chemokine signaling pathway | 22 | 4.39844E-06 |
| NOD-like receptor signaling pathway | 9 | 0.124958845 | Regulation of autophagy | 2 | 0.151184688 | Autoimmune thyroid disease | 11 | 4.48366E-06 |
| Ras signaling pathway | 29 | 0.130426801 | Sphingolipid signaling pathway | 4 | 0.153199211 | Metabolism of xenobiotics by cytochrome P450 | 13 | 4.70936E-06 |
| Herpes simplex infection | 24 | 0.135071857 | Bladder cancer | 2 | 0.157228292 | Prion diseases | 9 | 6.08341E-06 |
| Biosynthesis of amino acids | 11 | 0.142237127 | Rap1 signaling pathway | 6 | 0.157780431 | Cholinergic synapse | 16 | 6.85591E-06 |
| Fc gamma R-mediated phagocytosis | 13 | 0.145335328 | Endocytosis | 7 | 0.162889286 | Serotonergic synapse | 16 | 7.71409E-06 |
| Serotonergic synapse | 15 | 0.170929908 | Regulation of actin cytoskeleton | 6 | 0.167606287 | Alzheimer's disease | 20 | 1.00276E-05 |
| MAPK signaling pathway | 31 | 0.18018631 | Chemical carcinogenesis | 3 | 0.168206304 | Wnt signaling pathway | 18 | 1.16793E-05 |
| Platelet activation | 16 | 0.182360306 | Hepatitis C | 4 | 0.197307465 | Asthma | 8 | 1.51003E-05 |
| Bile secretion | 10 | 0.187907353 | Amino sugar and nucleotide sugar metabolism | 2 | 0.200614179 | Aldosterone-regulated sodium reabsorption | 9 | 1.58648E-05 |
| HTLV-I infection | 31 | 0.19809363 | Herpes simplex infection | 5 | 0.215277528 | RIG-I-like receptor signaling pathway | 12 | 1.64062E-05 |
| Adherens junction | 10 | 0.223915334 | Progesterone-mediated oocyte maturation | 3 | 0.239171269 | Longevity regulating pathway | 14 | 1.75841E-05 |
| Insulin resistance | 14 | 0.224672854 | Non-small cell lung cancer | 2 | 0.251592527 | Progesterone-mediated oocyte maturation | 14 | 2.8506E-05 |
| Long-term potentiation | 9 | 0.231935001 | AGE-RAGE signaling pathway in diabetic complications | 3 | 0.253036272 | Adherens junction | 12 | 2.9333E-05 |
| Cocaine addiction | 7 | 0.232935813 | Primary bile acid biosynthesis | 1 | 0.256258999 | Signaling pathways regulating pluripotency of stem cells | 17 | 4.30638E-05 |
| Amphetamine addiction | 9 | 0.245514915 | Phenylalanine metabolism | 1 | 0.256258999 | Dopaminergic synapse | 16 | 4.66993E-05 |
| Apoptosis - multiple species | 5 | 0.246071631 | Nitrogen metabolism | 1 | 0.256258999 | Vascular smooth muscle contraction | 15 | 7.33437E-05 |
| Rap1 signaling pathway | 25 | 0.25045594 | Acute myeloid leukemia | 2 | 0.258009049 | Hypertrophic cardiomyopathy (HCM) | 12 | 9.3903E-05 |
| Bacterial invasion of epithelial cells | 10 | 0.275619095 | Long-term depression | 2 | 0.277266061 | mTOR signaling pathway | 17 | 0.000110761 |
| Cardiac muscle contraction | 10 | 0.275619095 | Synaptic vesicle cycle | 2 | 0.296494604 | Regulation of actin cytoskeleton | 21 | 0.000118514 |
| Lysosome | 15 | 0.277433234 | Retinol metabolism | 2 | 0.30927455 | Melanogenesis | 13 | 0.00014743 |
| 2-Oxocarboxylic acid metabolism | 3 | 0.279016532 | Inflammatory bowel disease (IBD) | 2 | 0.30927455 | Cytosolic DNA-sensing pathway | 10 | 0.000162674 |
| Adipocytokine signaling pathway | 9 | 0.287740621 | Glutamatergic synapse | 3 | 0.314133583 | Gap junction | 12 | 0.000166632 |
| Gap junction | 11 | 0.289711726 | Terpenoid backbone biosynthesis | 1 | 0.318345863 | Glutathione metabolism | 9 | 0.000174698 |
| Epstein-Barr virus infection | 23 | 0.311406182 | Glycolysis / Gluconeogenesis | 2 | 0.322008342 | Long-term potentiation | 10 | 0.000241635 |
| Taurine and hypotaurine metabolism | 2 | 0.31349003 | Renal cell carcinoma | 2 | 0.322008342 | Fc gamma R-mediated phagocytosis | 12 | 0.000255328 |
| Prolactin signaling pathway | 9 | 0.31689267 | Protein export | 1 | 0.330130303 | cGMP-PKG signaling pathway | 17 | 0.000320499 |
| Glyoxylate and dicarboxylate metabolism | 4 | 0.32204668 | Renin-angiotensin system | 1 | 0.330130303 | ECM-receptor interaction | 11 | 0.000358294 |
| Carbohydrate digestion and absorption | 6 | 0.331450357 | Toxoplasmosis | 3 | 0.333078076 | Steroid hormone biosynthesis | 9 | 0.000410136 |
| Renin secretion | 8 | 0.332472071 | Drug metabolism - cytochrome P450 | 2 | 0.334684228 | PPAR signaling pathway | 10 | 0.000497382 |
| Pathogenic Escherichia coli infection | 7 | 0.33252746 | Histidine metabolism | 1 | 0.341712132 | Long-term depression | 9 | 0.000531045 |
| One carbon pool by folate | 3 | 0.337587831 | Thyroid hormone synthesis | 2 | 0.347291308 | Caffeine metabolism | 3 | 0.000577145 |
| Endocytosis | 29 | 0.343560107 | alpha-Linolenic acid metabolism | 1 | 0.353094815 | Cocaine addiction | 8 | 0.000602639 |
| Nicotinate and nicotinamide metabolism | 4 | 0.346305473 | Lysosome | 3 | 0.356731334 | Glucagon signaling pathway | 12 | 0.000609291 |
| Shigellosis | 8 | 0.348448394 | Influenza A | 4 | 0.357051385 | Huntington's disease | 18 | 0.000626412 |
| Cholinergic synapse | 13 | 0.349349823 | Adherens junction | 2 | 0.366051041 | Arachidonic acid metabolism | 9 | 0.000679734 |
| Regulation of lipolysis in adipocytes | 7 | 0.349797929 | Platinum drug resistance | 2 | 0.372259428 | Tryptophan metabolism | 7 | 0.000860185 |
| Endocrine and other factor-regulated calcium reabsorption | 6 | 0.350367015 | Alcoholism | 4 | 0.372774313 | Galactose metabolism | 6 | 0.001165517 |
| Tight junction | 16 | 0.351064387 | Transcriptional misregulation in cancer | 4 | 0.372774313 | Tight junction | 14 | 0.001177054 |
| Riboflavin metabolism | 1 | 0.351330743 | Phototransduction | 1 | 0.375276303 | Phospholipase D signaling pathway | 14 | 0.001548064 |
| AMPK signaling pathway | 14 | 0.38168698 | Measles | 3 | 0.412914641 | Cell adhesion molecules (CAMs) | 14 | 0.001548064 |
| Circadian entrainment | 11 | 0.382547656 | Fructose and mannose metabolism | 1 | 0.437383723 | Bile secretion | 9 | 0.001825577 |
| Signaling pathways regulating pluripotency of stem cells | 16 | 0.38408248 | Apoptosis - multiple species | 1 | 0.437383723 | Insulin secretion | 10 | 0.001841048 |
| Hepatitis C | 15 | 0.388736589 | Salmonella infection | 2 | 0.438750365 | Carbohydrate digestion and absorption | 7 | 0.002014741 |
| Glycine, serine and threonine metabolism | 5 | 0.391955328 | Phospholipase D signaling pathway | 3 | 0.449539611 | Tyrosine metabolism | 6 | 0.002248803 |
| Regulation of autophagy | 5 | 0.391955328 | Tyrosine metabolism | 1 | 0.456689853 | Dilated cardiomyopathy | 10 | 0.002601885 |
| Other types of O-glycan biosynthesis | 4 | 0.394811096 | Prion diseases | 1 | 0.456689853 | Longevity regulating pathway - multiple species | 8 | 0.003533963 |
| Phospholipase D signaling pathway | 16 | 0.395180669 | Tryptophan metabolism | 1 | 0.502121149 | Mineral absorption | 7 | 0.004111673 |
| Systemic lupus erythematosus | 15 | 0.400236092 | Fat digestion and absorption | 1 | 0.51074347 | Aldosterone synthesis and secretion | 9 | 0.004525643 |
| Ovarian steroidogenesis | 6 | 0.407432596 | Estrogen signaling pathway | 2 | 0.5121405 | Calcium signaling pathway | 15 | 0.004854639 |
| Butirosin and neomycin biosynthesis | 1 | 0.417873752 | Type I diabetes mellitus | 1 | 0.527545149 | Pathogenic Escherichia coli infection | 7 | 0.005637181 |
| Caffeine metabolism | 1 | 0.417873752 | ABC transporters | 1 | 0.535729551 | Staphylococcus aureus infection | 7 | 0.005637181 |
| Protein processing in endoplasmic reticulum | 18 | 0.427582404 | Ether lipid metabolism | 1 | 0.543772951 | Regulation of lipolysis in adipocytes | 7 | 0.006229363 |
| Salivary secretion | 10 | 0.430058976 | Drug metabolism - other enzymes | 1 | 0.551677766 | Retrograde endocannabinoid signaling | 10 | 0.006475054 |
| Insulin signaling pathway | 15 | 0.457930624 | Hedgehog signaling pathway | 1 | 0.551677766 | Endocytosis | 19 | 0.007299827 |
| PPAR signaling pathway | 8 | 0.461343827 | Sphingolipid metabolism | 1 | 0.55944637 | Phagosome | 13 | 0.00754462 |
| Choline metabolism in cancer | 11 | 0.464040436 | Carbon metabolism | 2 | 0.5838474 | Taurine and hypotaurine metabolism | 3 | 0.007973312 |
| Cytosolic DNA-sensing pathway | 7 | 0.471288109 | Amyotrophic lateral sclerosis (ALS) | 1 | 0.589204768 | Arginine biosynthesis | 4 | 0.008425082 |
| Synaptic vesicle cycle | 7 | 0.471288109 | Vibrio cholerae infection | 1 | 0.589204768 | Oocyte meiosis | 11 | 0.009342409 |
| Non-alcoholic fatty liver disease (NAFLD) | 16 | 0.484320593 | Tuberculosis | 3 | 0.592683 | Bacterial invasion of epithelial cells | 8 | 0.011621284 |
| Alanine, aspartate and glutamate metabolism | 4 | 0.489409322 | Mineral absorption | 1 | 0.596326545 | Circadian entrainment | 9 | 0.012596746 |
| Tyrosine metabolism | 4 | 0.489409322 | Endometrial cancer | 1 | 0.596326545 | Renin secretion | 7 | 0.012759924 |
| Gastric acid secretion | 8 | 0.49314042 | Thyroid hormone signaling pathway | 2 | 0.607517001 | Maturity onset diabetes of the young | 4 | 0.01802495 |
| Cysteine and methionine metabolism | 5 | 0.495915853 | Staphylococcus aureus infection | 1 | 0.616963526 | Thyroid hormone synthesis | 7 | 0.021629718 |
| Hippo signaling pathway | 16 | 0.506385783 | Basal cell carcinoma | 1 | 0.616963526 | GABAergic synapse | 8 | 0.022720361 |
| Maturity onset diabetes of the young | 3 | 0.507141222 | Chemokine signaling pathway | 3 | 0.629992232 | Dorso-ventral axis formation | 4 | 0.02321081 |
| Insulin secretion | 9 | 0.512039735 | Cell cycle | 2 | 0.634562435 | Starch and sucrose metabolism | 6 | 0.02435042 |
| Drug metabolism - other enzymes | 5 | 0.515972621 | Viral myocarditis | 1 | 0.636551076 | Protein digestion and absorption | 8 | 0.025615529 |
| Hedgehog signaling pathway | 5 | 0.515972621 | VEGF signaling pathway | 1 | 0.655142277 | Linoleic acid metabolism | 4 | 0.02610938 |
| Nitrogen metabolism | 2 | 0.532511754 | p53 signaling pathway | 1 | 0.700223156 | Arrhythmogenic right ventricular cardiomyopathy (ARVC) | 7 | 0.026493028 |
| Primary bile acid biosynthesis | 2 | 0.532511754 | Wnt signaling pathway | 2 | 0.706984891 | Complement and coagulation cascades | 7 | 0.036164562 |
| Phosphatidylinositol signaling system | 10 | 0.555942496 | Parkinson's disease | 2 | 0.706984891 | Glutamatergic synapse | 9 | 0.036375957 |
| Dopaminergic synapse | 13 | 0.569790105 | PPAR signaling pathway | 1 | 0.715573473 | Endocrine and other factor-regulated calcium reabsorption | 5 | 0.03699625 |
| Arginine and proline metabolism | 5 | 0.573961546 | Antigen processing and presentation | 1 | 0.739439515 | Systemic lupus erythematosus | 10 | 0.039440544 |
| Vascular smooth muscle contraction | 12 | 0.581586987 | EGFR tyrosine kinase inhibitor resistance | 1 | 0.757089719 | Arginine and proline metabolism | 5 | 0.043173477 |
| GnRH signaling pathway | 9 | 0.597062127 | MicroRNAs in cancer | 4 | 0.763074761 | Peroxisome | 7 | 0.045396985 |
| Aldosterone synthesis and secretion | 8 | 0.599079503 | Hematopoietic cell lineage | 1 | 0.781360053 | Alanine, aspartate and glutamate metabolism | 4 | 0.047913112 |
| Inositol phosphate metabolism | 7 | 0.601771064 | ErbB signaling pathway | 1 | 0.78516378 | Alcoholism | 12 | 0.051461778 |
| Citrate cycle (TCA cycle) | 3 | 0.606761894 | Rheumatoid arthritis | 1 | 0.788901697 | Primary immunodeficiency | 4 | 0.052288564 |
| Huntington's disease | 19 | 0.610652003 | Protein digestion and absorption | 1 | 0.792574935 | Renin-angiotensin system | 3 | 0.060403076 |
| B cell receptor signaling pathway | 7 | 0.616893997 | Glycerophospholipid metabolism | 1 | 0.810009238 | Salivary secretion | 7 | 0.061865481 |
| Fat digestion and absorption | 4 | 0.617922977 | Phosphatidylinositol signaling system | 1 | 0.819762044 | Basal cell carcinoma | 5 | 0.065218782 |
| Glycosaminoglycan biosynthesis - chondroitin sulfate / dermatan sulfate | 2 | 0.622834181 | Amoebiasis | 1 | 0.822901039 | Adrenergic signaling in cardiomyocytes | 10 | 0.068413446 |
| Peroxisome | 8 | 0.627264637 | Melanogenesis | 1 | 0.825985667 | Carbon metabolism | 8 | 0.078494138 |
| beta-Alanine metabolism | 3 | 0.629470909 | Choline metabolism in cancer | 1 | 0.829016862 | Pancreatic secretion | 7 | 0.08515737 |
| Galactose metabolism | 3 | 0.629470909 | Huntington's disease | 2 | 0.849942195 | Fatty acid biosynthesis | 2 | 0.091161056 |
| Sulfur metabolism | 1 | 0.661222396 | Focal adhesion | 2 | 0.869186743 | Drug metabolism - other enzymes | 4 | 0.107196516 |
| Pancreatic secretion | 9 | 0.66202788 | Proteoglycans in cancer | 2 | 0.872754413 | Hedgehog signaling pathway | 4 | 0.107196516 |
| ABC transporters | 4 | 0.674017848 | Metabolic pathways | 17 | 0.87444688 | Protein processing in endoplasmic reticulum | 10 | 0.118243313 |
| Vasopressin-regulated water reabsorption | 4 | 0.674017848 | Oxidative phosphorylation | 1 | 0.902648828 | Neuroactive ligand-receptor interaction | 15 | 0.134582938 |
| Ubiquinone and other terpenoid-quinone biosynthesis | 1 | 0.695995633 | Signaling pathways regulating pluripotency of stem cells | 1 | 0.916937477 | Base excision repair | 3 | 0.140219239 |
| Proximal tubule bicarbonate reclamation | 2 | 0.699046271 | Hippo signaling pathway | 1 | 0.931598311 | Phenylalanine metabolism | 2 | 0.143576695 |
| Starch and sucrose metabolism | 5 | 0.708436864 | Protein processing in endoplasmic reticulum | 1 | 0.944670476 | Riboflavin metabolism | 1 | 0.148953392 |
| Parkinson's disease | 13 | 0.708607687 | Olfactory transduction | 1 | 0.999382158 | 2-Oxocarboxylic acid metabolism | 2 | 0.157505902 |
| Epithelial cell signaling in Helicobacter pylori infection | 6 | 0.710426568 | Citrate cycle (TCA cycle) | 0 | 1 | Axon guidance | 10 | 0.158963069 |
| mTOR signaling pathway | 14 | 0.71379989 | Pentose phosphate pathway | 0 | 1 | Gastric acid secretion | 5 | 0.167878658 |
| Vitamin digestion and absorption | 2 | 0.721444211 | Pentose and glucuronate interconversions | 0 | 1 | Glycosaminoglycan degradation | 2 | 0.171673529 |
| Sphingolipid metabolism | 4 | 0.724138822 | Ascorbate and aldarate metabolism | 0 | 1 | Butirosin and neomycin biosynthesis | 1 | 0.182594841 |
| Axon guidance | 16 | 0.732056682 | Fatty acid biosynthesis | 0 | 1 | Parkinson's disease | 8 | 0.199552219 |
| Carbon metabolism | 10 | 0.734606643 | Fatty acid elongation | 0 | 1 | Nicotine addiction | 3 | 0.209161672 |
| Chemokine signaling pathway | 17 | 0.736505134 | Fatty acid degradation | 0 | 1 | Fat digestion and absorption | 3 | 0.219573328 |
| RIG-I-like receptor signaling pathway | 6 | 0.736621815 | Synthesis and degradation of ketone bodies | 0 | 1 | Mismatch repair | 2 | 0.229960435 |
| Glutamatergic synapse | 10 | 0.744603902 | Steroid biosynthesis | 0 | 1 | Proximal tubule bicarbonate reclamation | 2 | 0.229960435 |
| cAMP signaling pathway | 18 | 0.748424102 | Ubiquinone and other terpenoid-quinone biosynthesis | 0 | 1 | Metabolic pathways | 54 | 0.239255099 |
| N-Glycan biosynthesis | 4 | 0.754228262 | Arginine biosynthesis | 0 | 1 | Histidine metabolism | 2 | 0.24476728 |
| Non-homologous end-joining | 1 | 0.755208854 | Purine metabolism | 0 | 1 | Vitamin digestion and absorption | 2 | 0.24476728 |
| Folate biosynthesis | 1 | 0.780342289 | Caffeine metabolism | 0 | 1 | Proteasome | 3 | 0.251378007 |
| Vibrio cholerae infection | 4 | 0.781722767 | Pyrimidine metabolism | 0 | 1 | Retinol metabolism | 4 | 0.254883251 |
| Tryptophan metabolism | 3 | 0.792624342 | Alanine, aspartate and glutamate metabolism | 0 | 1 | Glycolysis / Gluconeogenesis | 4 | 0.272469209 |
| Butanoate metabolism | 2 | 0.797375356 | Glycine, serine and threonine metabolism | 0 | 1 | Phototransduction | 2 | 0.289295526 |
| Wnt signaling pathway | 12 | 0.800489494 | Cysteine and methionine metabolism | 0 | 1 | Morphine addiction | 5 | 0.29077438 |
| Glycosaminoglycan biosynthesis - keratan sulfate | 1 | 0.802897375 | Valine, leucine and isoleucine degradation | 0 | 1 | Glyoxylate and dicarboxylate metabolism | 2 | 0.304094634 |
| Glycosphingolipid biosynthesis - ganglio series | 1 | 0.802897375 | Valine, leucine and isoleucine biosynthesis | 0 | 1 | Butanoate metabolism | 2 | 0.304094634 |
| Linoleic acid metabolism | 2 | 0.813244283 | Lysine biosynthesis | 0 | 1 | Pentose phosphate pathway | 2 | 0.318836748 |
| Pentose phosphate pathway | 2 | 0.813244283 | Lysine degradation | 0 | 1 | Sulfur metabolism | 1 | 0.331915091 |
| Melanogenesis | 8 | 0.817656797 | Arginine and proline metabolism | 0 | 1 | Citrate cycle (TCA cycle) | 2 | 0.333503299 |
| Retrograde endocannabinoid signaling | 8 | 0.826017582 | Phenylalanine, tyrosine and tryptophan biosynthesis | 0 | 1 | Phosphatidylinositol signaling system | 5 | 0.345557709 |
| Circadian rhythm | 2 | 0.827993025 | beta-Alanine metabolism | 0 | 1 | beta-Alanine metabolism | 2 | 0.348077265 |
| Basal cell carcinoma | 4 | 0.829330165 | Taurine and hypotaurine metabolism | 0 | 1 | Lysosome | 6 | 0.358818557 |
| Phenylalanine metabolism | 1 | 0.841302559 | Selenocompound metabolism | 0 | 1 | Antigen processing and presentation | 4 | 0.362476991 |
| Selenocompound metabolism | 1 | 0.841302559 | D-Glutamine and D-glutamate metabolism | 0 | 1 | Folate biosynthesis | 1 | 0.431518886 |
| Basal transcription factors | 3 | 0.8540681 | D-Arginine and D-ornithine metabolism | 0 | 1 | Glycine, serine and threonine metabolism | 2 | 0.472923635 |
| Viral carcinogenesis | 17 | 0.854576733 | Glutathione metabolism | 0 | 1 | Pyruvate metabolism | 2 | 0.472923635 |
| Fructose and mannose metabolism | 2 | 0.866138669 | N-Glycan biosynthesis | 0 | 1 | Regulation of autophagy | 2 | 0.472923635 |
| Glycerophospholipid metabolism | 7 | 0.867727155 | Other glycan degradation | 0 | 1 | Selenocompound metabolism | 1 | 0.496370456 |
| Glycerolipid metabolism | 4 | 0.86797237 | Mucin type O-Glycan biosynthesis | 0 | 1 | Porphyrin and chlorophyll metabolism | 2 | 0.498707363 |
| SNARE interactions in vesicular transport | 2 | 0.877020269 | Other types of O-glycan biosynthesis | 0 | 1 | Other glycan degradation | 1 | 0.516303665 |
| Amino sugar and nucleotide sugar metabolism | 3 | 0.882757886 | Glycosaminoglycan degradation | 0 | 1 | Pantothenate and CoA biosynthesis | 1 | 0.516303665 |
| Notch signaling pathway | 3 | 0.882757886 | Glycosaminoglycan biosynthesis - chondroitin sulfate / dermatan sulfate | 0 | 1 | Glycerophospholipid metabolism | 4 | 0.520863499 |
| Progesterone-mediated oocyte maturation | 7 | 0.887291689 | Glycosaminoglycan biosynthesis - keratan sulfate | 0 | 1 | ABC transporters | 2 | 0.523670338 |
| GABAergic synapse | 6 | 0.899436534 | Glycosaminoglycan biosynthesis - heparan sulfate / heparin | 0 | 1 | Sphingolipid metabolism | 2 | 0.559517618 |
| Terpenoid backbone biosynthesis | 1 | 0.907704671 | Glycerolipid metabolism | 0 | 1 | Amino sugar and nucleotide sugar metabolism | 2 | 0.571032015 |
| Lysine degradation | 3 | 0.913154092 | Inositol phosphate metabolism | 0 | 1 | Fatty acid metabolism | 2 | 0.571032015 |
| Fanconi anemia pathway | 3 | 0.925494877 | Glycosylphosphatidylinositol(GPI)-anchor biosynthesis | 0 | 1 | Biosynthesis of amino acids | 3 | 0.573783783 |
| Histidine metabolism | 1 | 0.925699792 | Glycosphingolipid biosynthesis - lacto and neolacto series | 0 | 1 | Terpenoid backbone biosynthesis | 1 | 0.588469616 |
| Nicotine addiction | 2 | 0.92685816 | Glycosphingolipid biosynthesis - globo series | 0 | 1 | Vibrio cholerae infection | 2 | 0.604255583 |
| alpha-Linolenic acid metabolism | 1 | 0.933336522 | Glycosphingolipid biosynthesis - ganglio series | 0 | 1 | Protein export | 1 | 0.604765488 |
| Glycosylphosphatidylinositol(GPI)-anchor biosynthesis | 1 | 0.933336522 | Pyruvate metabolism | 0 | 1 | Glycosaminoglycan biosynthesis - heparan sulfate / heparin | 1 | 0.620417584 |
| Glycosphingolipid biosynthesis - lacto and neolacto series | 1 | 0.940188994 | Glyoxylate and dicarboxylate metabolism | 0 | 1 | alpha-Linolenic acid metabolism | 1 | 0.635451277 |
| Spliceosome | 9 | 0.940863217 | Propanoate metabolism | 0 | 1 | Glycerolipid metabolism | 2 | 0.683225956 |
| Ascorbate and aldarate metabolism | 1 | 0.946337681 | Butanoate metabolism | 0 | 1 | Circadian rhythm | 1 | 0.702164064 |
| Collecting duct acid secretion | 1 | 0.946337681 | One carbon pool by folate | 0 | 1 | Propanoate metabolism | 1 | 0.725303211 |
| Cell cycle | 8 | 0.947853269 | Thiamine metabolism | 0 | 1 | Fructose and mannose metabolism | 1 | 0.736191231 |
| Hippo signaling pathway -multiple species | 1 | 0.951854804 | Riboflavin metabolism | 0 | 1 | Pentose and glucuronate interconversions | 1 | 0.766339254 |
| Ether lipid metabolism | 2 | 0.953134723 | Nicotinate and nicotinamide metabolism | 0 | 1 | DNA replication | 1 | 0.766339254 |
| Glucagon signaling pathway | 6 | 0.954670266 | Pantothenate and CoA biosynthesis | 0 | 1 | Inositol phosphate metabolism | 2 | 0.777039395 |
| Homologous recombination | 1 | 0.95680518 | Biotin metabolism | 0 | 1 | Cardiac muscle contraction | 2 | 0.819766903 |
| Nucleotide excision repair | 2 | 0.960880176 | Lipoic acid metabolism | 0 | 1 | Vasopressin-regulated water reabsorption | 1 | 0.830968807 |
| RNA degradation | 4 | 0.962635768 | Folate biosynthesis | 0 | 1 | Cysteine and methionine metabolism | 1 | 0.837676395 |
| Neuroactive ligand-receptor interaction | 20 | 0.96814369 | Porphyrin and chlorophyll metabolism | 0 | 1 | Ether lipid metabolism | 1 | 0.837676395 |
| Retinol metabolism | 3 | 0.968958439 | Sulfur metabolism | 0 | 1 | Taste transduction | 2 | 0.845643489 |
| Aminoacyl-tRNA biosynthesis | 3 | 0.971402282 | Aminoacyl-tRNA biosynthesis | 0 | 1 | Nucleotide excision repair | 1 | 0.850305405 |
| Base excision repair | 1 | 0.972016734 | Biosynthesis of unsaturated fatty acids | 0 | 1 | Notch signaling pathway | 1 | 0.856247392 |
| Oocyte meiosis | 7 | 0.974422599 | 2-Oxocarboxylic acid metabolism | 0 | 1 | N-Glycan biosynthesis | 1 | 0.86195407 |
| Glutathione metabolism | 2 | 0.975236441 | Fatty acid metabolism | 0 | 1 | Lysine degradation | 1 | 0.877753397 |
| Taste transduction | 4 | 0.976227413 | Biosynthesis of amino acids | 0 | 1 | RNA transport | 4 | 0.910931784 |
| Pentose and glucuronate interconversions | 1 | 0.979795509 | Ribosome biogenesis in eukaryotes | 0 | 1 | Ubiquitin mediated proteolysis | 3 | 0.911993253 |
| Alcoholism | 11 | 0.980662768 | Ribosome | 0 | 1 | RNA degradation | 1 | 0.95566599 |
| Thyroid hormone synthesis | 3 | 0.981119391 | RNA transport | 0 | 1 | Ribosome biogenesis in eukaryotes | 1 | 0.96525801 |
| Pyrimidine metabolism | 5 | 0.985580348 | mRNA surveillance pathway | 0 | 1 | Purine metabolism | 3 | 0.970740095 |
| Steroid hormone biosynthesis | 2 | 0.985825824 | RNA degradation | 0 | 1 | mRNA surveillance pathway | 1 | 0.974905402 |
| Pyruvate metabolism | 1 | 0.986914817 | RNA polymerase | 0 | 1 | Pyrimidine metabolism | 1 | 0.985216751 |
| Morphine addiction | 4 | 0.987241703 | Basal transcription factors | 0 | 1 | Oxidative phosphorylation | 1 | 0.99547056 |
| Arachidonic acid metabolism | 2 | 0.990275584 | DNA replication | 0 | 1 | Spliceosome | 1 | 0.995651861 |
| Proteasome | 1 | 0.991527052 | Spliceosome | 0 | 1 | Olfactory transduction | 2 | 0.99999931 |
| Chemical carcinogenesis | 3 | 0.992625892 | Proteasome | 0 | 1 | Ascorbate and aldarate metabolism | 0 | 1 |
| Ribosome biogenesis in eukaryotes | 3 | 0.993240835 | Base excision repair | 0 | 1 | Fatty acid elongation | 0 | 1 |
| RNA transport | 9 | 0.993533336 | Nucleotide excision repair | 0 | 1 | Fatty acid degradation | 0 | 1 |
| Glycolysis / Gluconeogenesis | 2 | 0.993956694 | Mismatch repair | 0 | 1 | Synthesis and degradation of ketone bodies | 0 | 1 |
| Drug metabolism - cytochrome P450 | 2 | 0.995010459 | Homologous recombination | 0 | 1 | Steroid biosynthesis | 0 | 1 |
| Ubiquitin mediated proteolysis | 6 | 0.996354284 | Non-homologous end-joining | 0 | 1 | Primary bile acid biosynthesis | 0 | 1 |
| Metabolism of xenobiotics by cytochrome P450 | 2 | 0.996605885 | Fanconi anemia pathway | 0 | 1 | Ubiquinone and other terpenoid-quinone biosynthesis | 0 | 1 |
| mRNA surveillance pathway | 3 | 0.996660239 | Cytokine-cytokine receptor interaction | 0 | 1 | Valine, leucine and isoleucine degradation | 0 | 1 |
| Purine metabolism | 7 | 0.999314687 | Ubiquitin mediated proteolysis | 0 | 1 | Valine, leucine and isoleucine biosynthesis | 0 | 1 |
| Metabolic pathways | 95 | 0.999621698 | Sulfur relay system | 0 | 1 | Lysine biosynthesis | 0 | 1 |
| Oxidative phosphorylation | 4 | 0.999627447 | SNARE interactions in vesicular transport | 0 | 1 | Phenylalanine, tyrosine and tryptophan biosynthesis | 0 | 1 |
| Olfactory transduction | 1 | 1 | Phagosome | 0 | 1 | D-Glutamine and D-glutamate metabolism | 0 | 1 |
| Biosynthesis of unsaturated fatty acids | 0 | 1 | Peroxisome | 0 | 1 | D-Arginine and D-ornithine metabolism | 0 | 1 |
| Biotin metabolism | 0 | 1 | Dorso-ventral axis formation | 0 | 1 | Mucin type O-Glycan biosynthesis | 0 | 1 |
| D-Glutamine and D-glutamate metabolism | 0 | 1 | Notch signaling pathway | 0 | 1 | Other types of O-glycan biosynthesis | 0 | 1 |
| DNA replication | 0 | 1 | TGF-beta signaling pathway | 0 | 1 | Glycosaminoglycan biosynthesis - chondroitin sulfate / dermatan sulfate | 0 | 1 |
| Fatty acid biosynthesis | 0 | 1 | Axon guidance | 0 | 1 | Glycosaminoglycan biosynthesis - keratan sulfate | 0 | 1 |
| Fatty acid degradation | 0 | 1 | Hippo signaling pathway -multiple species | 0 | 1 | Glycosylphosphatidylinositol(GPI)-anchor biosynthesis | 0 | 1 |
| Fatty acid elongation | 0 | 1 | ECM-receptor interaction | 0 | 1 | Glycosphingolipid biosynthesis - lacto and neolacto series | 0 | 1 |
| Fatty acid metabolism | 0 | 1 | Cell adhesion molecules (CAMs) | 0 | 1 | Glycosphingolipid biosynthesis - globo series | 0 | 1 |
| Glycosaminoglycan biosynthesis - heparan sulfate / heparin | 0 | 1 | Tight junction | 0 | 1 | Glycosphingolipid biosynthesis - ganglio series | 0 | 1 |
| Glycosaminoglycan degradation | 0 | 1 | Jak-STAT signaling pathway | 0 | 1 | One carbon pool by folate | 0 | 1 |
| Glycosphingolipid biosynthesis - globo series | 0 | 1 | Natural killer cell mediated cytotoxicity | 0 | 1 | Thiamine metabolism | 0 | 1 |
| Lipoic acid metabolism | 0 | 1 | Fc epsilon RI signaling pathway | 0 | 1 | Vitamin B6 metabolism | 0 | 1 |
| Lysine biosynthesis | 0 | 1 | Fc gamma R-mediated phagocytosis | 0 | 1 | Nicotinate and nicotinamide metabolism | 0 | 1 |
| Mismatch repair | 0 | 1 | Leukocyte transendothelial migration | 0 | 1 | Biotin metabolism | 0 | 1 |
| Mucin type O-Glycan biosynthesis | 0 | 1 | Intestinal immune network for IgA production | 0 | 1 | Lipoic acid metabolism | 0 | 1 |
| Other glycan degradation | 0 | 1 | Endocrine and other factor-regulated calcium reabsorption | 0 | 1 | Nitrogen metabolism | 0 | 1 |
| Phenylalanine, tyrosine and tryptophan biosynthesis | 0 | 1 | Vasopressin-regulated water reabsorption | 0 | 1 | Aminoacyl-tRNA biosynthesis | 0 | 1 |
| Phototransduction | 0 | 1 | Proximal tubule bicarbonate reclamation | 0 | 1 | Biosynthesis of unsaturated fatty acids | 0 | 1 |
| Propanoate metabolism | 0 | 1 | Vitamin digestion and absorption | 0 | 1 | Ribosome | 0 | 1 |
| Protein export | 0 | 1 | Bacterial invasion of epithelial cells | 0 | 1 | RNA polymerase | 0 | 1 |
| Ribosome | 0 | 1 | Pathogenic Escherichia coli infection | 0 | 1 | Basal transcription factors | 0 | 1 |
| RNA polymerase | 0 | 1 | African trypanosomiasis | 0 | 1 | Homologous recombination | 0 | 1 |
| Steroid biosynthesis | 0 | 1 | Malaria | 0 | 1 | Non-homologous end-joining | 0 | 1 |
| Sulfur relay system | 0 | 1 | Asthma | 0 | 1 | Fanconi anemia pathway | 0 | 1 |
| Synthesis and degradation of ketone bodies | 0 | 1 | Autoimmune thyroid disease | 0 | 1 | Sulfur relay system | 0 | 1 |
| Thiamine metabolism | 0 | 1 | Systemic lupus erythematosus | 0 | 1 | SNARE interactions in vesicular transport | 0 | 1 |
| Valine, leucine and isoleucine biosynthesis | 0 | 1 | Allograft rejection | 0 | 1 | Hippo signaling pathway -multiple species | 0 | 1 |
| Valine, leucine and isoleucine degradation | 0 | 1 | Graft-versus-host disease | 0 | 1 | Synaptic vesicle cycle | 0 | 1 |
| Vitamin B6 metabolism | 0 | 1 | Primary immunodeficiency | 0 | 1 | Collecting duct acid secretion | 0 | 1 |

Pathways in M1

| **Symptom** | **overlap** | **p_value** | **Drug** | **overlap** | **p_value** | **Herb** | **overlap** | **p_value** |
| --- | --- | --- | --- | --- | --- | --- | --- | --- |
| MicroRNAs in cancer | 180 | 8.91047E-54 | Neuroactive ligand-receptor interaction | 29 | 8.20244E-28 | Pathways in cancer | 72 | 1.49453E-36 |
| Pathways in cancer | 195 | 5.2495E-40 | Calcium signaling pathway | 24 | 1.77712E-25 | Hepatitis B | 46 | 1.22661E-34 |
| Pancreatic cancer | 56 | 1.17829E-29 | Complement and coagulation cascades | 10 | 1.00012E-10 | AGE-RAGE signaling pathway in diabetic complications | 37 | 1.02802E-30 |
| Prostate cancer | 65 | 3.84728E-27 | Serotonergic synapse | 11 | 1.78758E-10 | FoxO signaling pathway | 40 | 2.76319E-29 |
| AGE-RAGE signaling pathway in diabetic complications | 69 | 6.60151E-26 | cAMP signaling pathway | 13 | 5.83982E-10 | Endocrine resistance | 33 | 2.93242E-26 |
| Colorectal cancer | 51 | 7.80618E-26 | Adrenergic signaling in cardiomyocytes | 11 | 3.60061E-09 | Apoptosis | 38 | 5.00831E-26 |
| Proteoglycans in cancer | 108 | 1.1824E-25 | Renin secretion | 8 | 9.32786E-09 | PI3K-Akt signaling pathway | 54 | 2.44678E-24 |
| Non-small cell lung cancer | 47 | 1.1887E-24 | cGMP-PKG signaling pathway | 11 | 1.28563E-08 | TNF signaling pathway | 33 | 2.97338E-24 |
| Focal adhesion | 105 | 3.9281E-24 | Hypertrophic cardiomyopathy (HCM) | 8 | 7.51101E-08 | Prostate cancer | 30 | 8.07567E-24 |
| Small cell lung cancer | 60 | 1.9396E-23 | Cholinergic synapse | 8 | 7.24066E-07 | Pancreatic cancer | 26 | 8.09024E-23 |
| Chronic myeloid leukemia | 54 | 3.34623E-23 | Cardiac muscle contraction | 7 | 8.46053E-07 | Chagas disease (American trypanosomiasis) | 30 | 1.40195E-21 |
| Hepatitis B | 83 | 7.28228E-23 | Taste transduction | 7 | 1.29466E-06 | Platinum drug resistance | 26 | 3.86101E-21 |
| Endocrine resistance | 63 | 5.99265E-22 | Vascular smooth muscle contraction | 8 | 1.31331E-06 | Proteoglycans in cancer | 39 | 1.35102E-20 |
| EGFR tyrosine kinase inhibitor resistance | 56 | 1.17874E-21 | Dilated cardiomyopathy | 7 | 2.08093E-06 | Tuberculosis | 36 | 4.32794E-20 |
| FoxO signaling pathway | 76 | 3.16533E-21 | Dopaminergic synapse | 8 | 2.27056E-06 | Leishmaniasis | 24 | 3.88973E-19 |
| HIF-1 signaling pathway | 64 | 9.36408E-21 | Arrhythmogenic right ventricular cardiomyopathy (ARVC) | 6 | 9.78846E-06 | Toxoplasmosis | 29 | 9.08855E-19 |
| Bladder cancer | 36 | 1.55921E-20 | Oxytocin signaling pathway | 8 | 9.79725E-06 | Bladder cancer | 19 | 1.28074E-18 |
| Apoptosis | 77 | 4.11072E-20 | Gap junction | 6 | 2.65905E-05 | HIF-1 signaling pathway | 27 | 2.47124E-18 |
| Glioma | 47 | 1.13614E-19 | Salivary secretion | 6 | 2.83623E-05 | EGFR tyrosine kinase inhibitor resistance | 24 | 8.79772E-18 |
| PI3K-Akt signaling pathway | 140 | 1.15523E-19 | Aldosterone synthesis and secretion | 5 | 0.000208248 | HTLV-I infection | 40 | 9.03015E-18 |
| Endometrial cancer | 39 | 1.84439E-17 | Insulin secretion | 5 | 0.000261111 | NF-kappa B signaling pathway | 25 | 1.80694E-17 |
| Melanoma | 46 | 2.57601E-16 | Circadian entrainment | 5 | 0.000437844 | p53 signaling pathway | 22 | 3.64605E-17 |
| ErbB signaling pathway | 52 | 8.7956E-16 | Inflammatory mediator regulation of TRP channels | 5 | 0.000505245 | MAPK signaling pathway | 39 | 3.88684E-17 |
| Toxoplasmosis | 62 | 3.48675E-15 | Amphetamine addiction | 4 | 0.001057133 | Colorectal cancer | 21 | 4.70427E-17 |
| Renal cell carcinoma | 43 | 4.0718E-15 | MAPK signaling pathway | 7 | 0.00159804 | Chronic myeloid leukemia | 22 | 1.42158E-16 |
| Hypertrophic cardiomyopathy (HCM) | 48 | 3.62149E-14 | GnRH signaling pathway | 4 | 0.003265528 | Pertussis | 22 | 2.70857E-16 |
| TNF signaling pathway | 57 | 9.98942E-14 | Morphine addiction | 4 | 0.003265528 | Small cell lung cancer | 23 | 5.36805E-16 |
| p53 signaling pathway | 42 | 1.24988E-13 | Type II diabetes mellitus | 3 | 0.004093227 | Glioma | 20 | 2.25735E-15 |
| Prolactin signaling pathway | 43 | 1.64601E-13 | Regulation of actin cytoskeleton | 5 | 0.014844872 | Influenza A | 30 | 8.99315E-15 |
| Platinum drug resistance | 44 | 2.09328E-13 | GABAergic synapse | 3 | 0.021455788 | Insulin resistance | 24 | 1.45404E-14 |
| Acute myeloid leukemia | 36 | 1.45585E-12 | Alzheimer's disease | 4 | 0.02660918 | Osteoclast differentiation | 26 | 1.58473E-14 |
| Chagas disease (American trypanosomiasis) | 53 | 1.74634E-12 | Retrograde endocannabinoid signaling | 3 | 0.0306377 | Viral carcinogenesis | 32 | 1.73337E-14 |
| Neurotrophin signaling pathway | 57 | 1.06426E-11 | Cocaine addiction | 2 | 0.043047228 | Measles | 26 | 3.37549E-14 |
| Ras signaling pathway | 89 | 2.11875E-11 | Platelet activation | 3 | 0.04916385 | Cell cycle | 25 | 3.53419E-14 |
| T cell receptor signaling pathway | 51 | 4.62665E-11 | Staphylococcus aureus infection | 2 | 0.052982335 | Toll-like receptor signaling pathway | 23 | 7.33809E-14 |
| Thyroid hormone signaling pathway | 55 | 6.1746E-11 | Long-term potentiation | 2 | 0.07303181 | VEGF signaling pathway | 18 | 1.32875E-13 |
| Dilated cardiomyopathy | 45 | 1.16914E-10 | Gastric acid secretion | 2 | 0.088900871 | Melanoma | 19 | 1.97465E-13 |
| Fc epsilon RI signaling pathway | 37 | 1.97423E-10 | Purine metabolism | 3 | 0.112785905 | MicroRNAs in cancer | 37 | 2.54645E-13 |
| Amoebiasis | 47 | 7.04599E-10 | Alcoholism | 3 | 0.120049359 | Prolactin signaling pathway | 19 | 2.60915E-13 |
| VEGF signaling pathway | 34 | 7.70559E-10 | Transcriptional misregulation in cancer | 3 | 0.120049359 | Malaria | 16 | 5.31924E-13 |
| Central carbon metabolism in cancer | 36 | 9.65793E-10 | Estrogen signaling pathway | 2 | 0.14377305 | T cell receptor signaling pathway | 22 | 5.57285E-13 |
| Rap1 signaling pathway | 78 | 6.94941E-09 | Renin-angiotensin system | 1 | 0.144318788 | Transcriptional misregulation in cancer | 28 | 7.77021E-13 |
| Arrhythmogenic right ventricular cardiomyopathy (ARVC) | 37 | 7.76641E-09 | Chagas disease (American trypanosomiasis) | 2 | 0.15547331 | Focal adhesion | 29 | 3.13224E-12 |
| Thyroid cancer | 20 | 1.5766E-08 | Phototransduction | 1 | 0.167230579 | Non-small cell lung cancer | 16 | 5.45009E-12 |
| Longevity regulating pathway - multiple species | 33 | 1.87925E-08 | Thyroid cancer | 1 | 0.178458622 | African trypanosomiasis | 13 | 8.24444E-12 |
| Insulin signaling pathway | 56 | 3.0834E-08 | Glutamatergic synapse | 2 | 0.179384089 | Rheumatoid arthritis | 19 | 1.56641E-11 |
| Choline metabolism in cancer | 44 | 6.23982E-08 | AMPK signaling pathway | 2 | 0.201357652 | Adipocytokine signaling pathway | 17 | 2.08755E-11 |
| Measles | 54 | 7.2157E-08 | Aldosterone-regulated sodium reabsorption | 1 | 0.232398803 | Neurotrophin signaling pathway | 21 | 7.1806E-11 |
| cAMP signaling pathway | 72 | 7.25733E-08 | Parkinson's disease | 2 | 0.248603292 | Salmonella infection | 18 | 7.72252E-11 |
| Adherens junction | 35 | 1.17697E-07 | ABC transporters | 1 | 0.258044247 | NOD-like receptor signaling pathway | 15 | 9.37972E-11 |
| HTLV-I infection | 87 | 1.36575E-07 | Hedgehog signaling pathway | 1 | 0.268064274 | Apoptosis - multiple species | 12 | 1.07304E-10 |
| Longevity regulating pathway | 41 | 1.67739E-07 | Carbohydrate digestion and absorption | 1 | 0.268064274 | Estrogen signaling pathway | 19 | 1.12307E-10 |
| Toll-like receptor signaling pathway | 44 | 3.36457E-07 | Sphingolipid metabolism | 1 | 0.273024148 | Ras signaling pathway | 28 | 2.90166E-10 |
| Apoptosis - multiple species | 20 | 3.91017E-07 | Malaria | 1 | 0.282844716 | Epstein-Barr virus infection | 26 | 3.68818E-10 |
| Osteoclast differentiation | 51 | 4.49938E-07 | Long-term depression | 1 | 0.334565413 | Thyroid hormone signaling pathway | 20 | 3.74072E-10 |
| Leishmaniasis | 33 | 6.69794E-07 | Glioma | 1 | 0.356842137 | Jak-STAT signaling pathway | 23 | 4.11962E-10 |
| Sphingolipid signaling pathway | 47 | 1.02824E-06 | p53 signaling pathway | 1 | 0.374132827 | AMPK signaling pathway | 20 | 8.0369E-10 |
| Cell cycle | 48 | 1.1824E-06 | Thyroid hormone synthesis | 1 | 0.382605524 | Amoebiasis | 18 | 8.8737E-10 |
| Viral myocarditis | 28 | 1.28E-06 | Bile secretion | 1 | 0.382605524 | Amyotrophic lateral sclerosis (ALS) | 13 | 2.64809E-09 |
| Malaria | 25 | 1.28866E-06 | PPAR signaling pathway | 1 | 0.386799373 | B cell receptor signaling pathway | 15 | 3.24448E-09 |
| B cell receptor signaling pathway | 32 | 2.27414E-06 | Pertussis | 1 | 0.399213157 | Hepatitis C | 20 | 3.30942E-09 |
| Insulin resistance | 43 | 2.34361E-06 | Proteoglycans in cancer | 2 | 0.404037625 | Sphingolipid signaling pathway | 19 | 3.41322E-09 |
| Estrogen signaling pathway | 40 | 2.58503E-06 | Rap1 signaling pathway | 2 | 0.418207301 | Non-alcoholic fatty liver disease (NAFLD) | 21 | 5.6549E-09 |
| Influenza A | 61 | 2.97764E-06 | Ras signaling pathway | 2 | 0.457424491 | Inflammatory bowel disease (IBD) | 14 | 6.93079E-09 |
| Leukocyte transendothelial migration | 45 | 4.05058E-06 | NF-kappa B signaling pathway | 1 | 0.465018212 | Legionellosis | 13 | 7.22699E-09 |
| Regulation of actin cytoskeleton | 71 | 4.47392E-06 | Longevity regulating pathway | 1 | 0.472276688 | ErbB signaling pathway | 16 | 7.80754E-09 |
| MAPK signaling pathway | 81 | 5.3622E-06 | Pancreatic secretion | 1 | 0.479438071 | Herpes simplex infection | 23 | 9.48227E-09 |
| Pertussis | 32 | 6.59878E-06 | Phosphatidylinositol signaling system | 1 | 0.486503641 | Fc epsilon RI signaling pathway | 14 | 1.05232E-08 |
| NOD-like receptor signaling pathway | 26 | 1.12689E-05 | Melanogenesis | 1 | 0.493474662 | Central carbon metabolism in cancer | 14 | 1.05232E-08 |
| Adipocytokine signaling pathway | 30 | 1.14357E-05 | Glucagon signaling pathway | 1 | 0.496925108 | Endometrial cancer | 12 | 3.70356E-08 |
| TGF-beta signaling pathway | 34 | 1.36842E-05 | AGE-RAGE signaling pathway in diabetic complications | 1 | 0.496925108 | Progesterone-mediated oocyte maturation | 16 | 3.87542E-08 |
| Chemokine signaling pathway | 62 | 1.54121E-05 | HIF-1 signaling pathway | 1 | 0.503756638 | GnRH signaling pathway | 15 | 9.10585E-08 |
| Inflammatory bowel disease (IBD) | 28 | 1.98182E-05 | Olfactory transduction | 3 | 0.536648865 | Epithelial cell signaling in Helicobacter pylori infection | 13 | 1.09223E-07 |
| Tuberculosis | 59 | 2.05052E-05 | Sphingolipid signaling pathway | 1 | 0.558251181 | Acute myeloid leukemia | 12 | 1.10656E-07 |
| ECM-receptor interaction | 33 | 2.10196E-05 | Neurotrophin signaling pathway | 1 | 0.558251181 | Longevity regulating pathway | 15 | 1.42072E-07 |
| Salmonella infection | 34 | 2.47143E-05 | Oocyte meiosis | 1 | 0.567235624 | Ovarian steroidogenesis | 11 | 2.34486E-07 |
| Hepatitis C | 47 | 2.64565E-05 | Lysosome | 1 | 0.567235624 | Oxytocin signaling pathway | 19 | 2.92169E-07 |
| Hematopoietic cell lineage | 34 | 3.28422E-05 | Osteoclast differentiation | 1 | 0.590322948 | Rap1 signaling pathway | 22 | 4.7089E-07 |
| Natural killer cell mediated cytotoxicity | 47 | 3.2941E-05 | MicroRNAs in cancer | 2 | 0.60351869 | Renal cell carcinoma | 12 | 7.13287E-07 |
| Jak-STAT signaling pathway | 53 | 4.37418E-05 | Insulin signaling pathway | 1 | 0.612195264 | Insulin signaling pathway | 17 | 1.07209E-06 |
| Viral carcinogenesis | 65 | 4.71667E-05 | Cell adhesion molecules (CAMs) | 1 | 0.622695628 | Choline metabolism in cancer | 14 | 2.16033E-06 |
| Adrenergic signaling in cardiomyocytes | 50 | 5.78372E-05 | Hippo signaling pathway | 1 | 0.647736059 | cAMP signaling pathway | 20 | 2.80999E-06 |
| Oxytocin signaling pathway | 52 | 7.50844E-05 | PI3K-Akt signaling pathway | 2 | 0.673685617 | Galactose metabolism | 8 | 2.96723E-06 |
| Signaling pathways regulating pluripotency of stem cells | 48 | 7.94004E-05 | Influenza A | 1 | 0.697202006 | Platelet activation | 15 | 4.34448E-06 |
| Cytokine-cytokine receptor interaction | 78 | 0.000147239 | Tuberculosis | 1 | 0.701343593 | Type I diabetes mellitus | 9 | 4.67078E-06 |
| Amyotrophic lateral sclerosis (ALS) | 22 | 0.000147268 | Huntington's disease | 1 | 0.732526873 | Drug metabolism - cytochrome P450 | 11 | 6.81968E-06 |
| Type II diabetes mellitus | 21 | 0.00016273 | Pathways in cancer | 1 | 0.935441359 | Serotonergic synapse | 14 | 7.47519E-06 |
| Shigellosis | 26 | 0.000173008 | Metabolic pathways | 1 | 0.999869069 | Prion diseases | 8 | 7.95699E-06 |
| Legionellosis | 23 | 0.000184872 | Glycolysis / Gluconeogenesis | 0 | 1 | Steroid hormone biosynthesis | 10 | 8.72163E-06 |
| Inflammatory mediator regulation of TRP channels | 35 | 0.000214588 | Citrate cycle (TCA cycle) | 0 | 1 | Glucagon signaling pathway | 13 | 1.14782E-05 |
| NF-kappa B signaling pathway | 33 | 0.000293092 | Pentose phosphate pathway | 0 | 1 | Metabolism of xenobiotics by cytochrome P450 | 11 | 1.19335E-05 |
| Bacterial invasion of epithelial cells | 29 | 0.000335007 | Pentose and glucuronate interconversions | 0 | 1 | Type II diabetes mellitus | 9 | 1.21433E-05 |
| African trypanosomiasis | 16 | 0.000354776 | Fructose and mannose metabolism | 0 | 1 | Adherens junction | 11 | 1.36409E-05 |
| Transcriptional misregulation in cancer | 55 | 0.00042526 | Galactose metabolism | 0 | 1 | Cytokine-cytokine receptor interaction | 22 | 2.00596E-05 |
| GnRH signaling pathway | 32 | 0.000538609 | Ascorbate and aldarate metabolism | 0 | 1 | Longevity regulating pathway - multiple species | 10 | 2.15243E-05 |
| Fc gamma R-mediated phagocytosis | 32 | 0.000669552 | Fatty acid biosynthesis | 0 | 1 | Oocyte meiosis | 14 | 2.21869E-05 |
| Aldosterone-regulated sodium reabsorption | 17 | 0.000709819 | Fatty acid elongation | 0 | 1 | Shigellosis | 10 | 2.47593E-05 |
| AMPK signaling pathway | 40 | 0.000732478 | Fatty acid degradation | 0 | 1 | Graft-versus-host disease | 8 | 2.75335E-05 |
| Phospholipase D signaling pathway | 45 | 0.000782945 | Synthesis and degradation of ketone bodies | 0 | 1 | Amphetamine addiction | 10 | 3.2495E-05 |
| Rheumatoid arthritis | 31 | 0.000785286 | Steroid biosynthesis | 0 | 1 | Inflammatory mediator regulation of TRP channels | 12 | 4.13956E-05 |
| Non-alcoholic fatty liver disease (NAFLD) | 47 | 0.000790715 | Primary bile acid biosynthesis | 0 | 1 | TGF-beta signaling pathway | 11 | 4.61895E-05 |
| mTOR signaling pathway | 47 | 0.001081866 | Ubiquinone and other terpenoid-quinone biosynthesis | 0 | 1 | Chemokine signaling pathway | 17 | 5.76822E-05 |
| Dorso-ventral axis formation | 13 | 0.001476096 | Steroid hormone biosynthesis | 0 | 1 | Natural killer cell mediated cytotoxicity | 14 | 5.8085E-05 |
| Regulation of lipolysis in adipocytes | 21 | 0.001880582 | Oxidative phosphorylation | 0 | 1 | Leukocyte transendothelial migration | 13 | 6.14143E-05 |
| Epithelial cell signaling in Helicobacter pylori infection | 24 | 0.00241033 | Arginine biosynthesis | 0 | 1 | Allograft rejection | 7 | 0.0001087 |
| Epstein-Barr virus infection | 57 | 0.002464894 | Caffeine metabolism | 0 | 1 | Dopaminergic synapse | 13 | 0.000153848 |
| cGMP-PKG signaling pathway | 49 | 0.002520964 | Pyrimidine metabolism | 0 | 1 | Glutathione metabolism | 8 | 0.000161925 |
| Gap junction | 29 | 0.002981615 | Alanine, aspartate and glutamate metabolism | 0 | 1 | Chemical carcinogenesis | 10 | 0.000187019 |
| Allograft rejection | 15 | 0.003426971 | Glycine, serine and threonine metabolism | 0 | 1 | Thyroid cancer | 6 | 0.000203295 |
| Hippo signaling pathway | 45 | 0.003509009 | Cysteine and methionine metabolism | 0 | 1 | mTOR signaling pathway | 14 | 0.000241605 |
| Calcium signaling pathway | 51 | 0.004061146 | Valine, leucine and isoleucine degradation | 0 | 1 | RIG-I-like receptor signaling pathway | 9 | 0.000261135 |
| Progesterone-mediated oocyte maturation | 31 | 0.004408506 | Valine, leucine and isoleucine biosynthesis | 0 | 1 | Hematopoietic cell lineage | 10 | 0.000305707 |
| Platelet activation | 37 | 0.004416364 | Lysine biosynthesis | 0 | 1 | PPAR signaling pathway | 9 | 0.000323983 |
| Axon guidance | 50 | 0.004693607 | Lysine degradation | 0 | 1 | Viral myocarditis | 8 | 0.000351858 |
| Prion diseases | 14 | 0.005345858 | Arginine and proline metabolism | 0 | 1 | Signaling pathways regulating pluripotency of stem cells | 13 | 0.000398709 |
| Amphetamine addiction | 22 | 0.009426388 | Histidine metabolism | 0 | 1 | Long-term depression | 8 | 0.000445483 |
| Cholinergic synapse | 33 | 0.009539552 | Tyrosine metabolism | 0 | 1 | Fc gamma R-mediated phagocytosis | 10 | 0.000481678 |
| Serotonergic synapse | 33 | 0.010984505 | Phenylalanine metabolism | 0 | 1 | Intestinal immune network for IgA production | 7 | 0.000511185 |
| Renin secretion | 21 | 0.011138169 | Tryptophan metabolism | 0 | 1 | Arachidonic acid metabolism | 8 | 0.000558384 |
| Cocaine addiction | 17 | 0.011860783 | Phenylalanine, tyrosine and tryptophan biosynthesis | 0 | 1 | Cocaine addiction | 7 | 0.000662708 |
| Type I diabetes mellitus | 15 | 0.016636465 | beta-Alanine metabolism | 0 | 1 | Vitamin digestion and absorption | 5 | 0.000679229 |
| Dopaminergic synapse | 36 | 0.019517314 | Taurine and hypotaurine metabolism | 0 | 1 | Aldosterone synthesis and secretion | 9 | 0.000781167 |
| Melanogenesis | 29 | 0.020353526 | Selenocompound metabolism | 0 | 1 | Melanogenesis | 10 | 0.000932876 |
| Autoimmune thyroid disease | 17 | 0.021904462 | D-Glutamine and D-glutamate metabolism | 0 | 1 | Autoimmune thyroid disease | 7 | 0.000954512 |
| Long-term depression | 19 | 0.022457852 | D-Arginine and D-ornithine metabolism | 0 | 1 | Aldosterone-regulated sodium reabsorption | 6 | 0.001080132 |
| Renin-angiotensin system | 9 | 0.027743788 | Glutathione metabolism | 0 | 1 | Bile secretion | 8 | 0.001385849 |
| Long-term potentiation | 20 | 0.031186841 | Starch and sucrose metabolism | 0 | 1 | Gap junction | 9 | 0.001420095 |
| Pathogenic Escherichia coli infection | 17 | 0.037375066 | N-Glycan biosynthesis | 0 | 1 | Phospholipase D signaling pathway | 12 | 0.001424273 |
| Cell adhesion molecules (CAMs) | 37 | 0.05352104 | Other glycan degradation | 0 | 1 | Alzheimer's disease | 13 | 0.001912444 |
| RIG-I-like receptor signaling pathway | 20 | 0.055524574 | Mucin type O-Glycan biosynthesis | 0 | 1 | Cholinergic synapse | 10 | 0.002068968 |
| Caffeine metabolism | 3 | 0.058380011 | Other types of O-glycan biosynthesis | 0 | 1 | Carbohydrate digestion and absorption | 6 | 0.002593178 |
| Asthma | 10 | 0.061981421 | Amino sugar and nucleotide sugar metabolism | 0 | 1 | Steroid biosynthesis | 4 | 0.002810992 |
| Herpes simplex infection | 46 | 0.063001721 | Butirosin and neomycin biosynthesis | 0 | 1 | Cytosolic DNA-sensing pathway | 7 | 0.002966419 |
| Vascular smooth muscle contraction | 31 | 0.073523021 | Glycosaminoglycan degradation | 0 | 1 | Arginine and proline metabolism | 6 | 0.003586572 |
| Phagosome | 38 | 0.085757035 | Glycosaminoglycan biosynthesis - chondroitin sulfate / dermatan sulfate | 0 | 1 | Hypertrophic cardiomyopathy (HCM) | 8 | 0.003749019 |
| Neuroactive ligand-receptor interaction | 65 | 0.089242669 | Glycosaminoglycan biosynthesis - keratan sulfate | 0 | 1 | Long-term potentiation | 7 | 0.003863577 |
| Graft-versus-host disease | 12 | 0.10370214 | Glycosaminoglycan biosynthesis - heparan sulfate / heparin | 0 | 1 | Tyrosine metabolism | 5 | 0.003942341 |
| Homologous recombination | 9 | 0.109824939 | Glycerolipid metabolism | 0 | 1 | Wnt signaling pathway | 11 | 0.004140062 |
| Ovarian steroidogenesis | 14 | 0.112347932 | Inositol phosphate metabolism | 0 | 1 | Insulin secretion | 8 | 0.004340755 |
| Tight junction | 34 | 0.117310089 | Glycosylphosphatidylinositol(GPI)-anchor biosynthesis | 0 | 1 | Regulation of actin cytoskeleton | 14 | 0.006246026 |
| Intestinal immune network for IgA production | 13 | 0.132539516 | Glycerophospholipid metabolism | 0 | 1 | Huntington's disease | 13 | 0.006280009 |
| Wnt signaling pathway | 34 | 0.145742907 | Ether lipid metabolism | 0 | 1 | Starch and sucrose metabolism | 6 | 0.007596762 |
| Primary immunodeficiency | 10 | 0.169789447 | Arachidonic acid metabolism | 0 | 1 | Fat digestion and absorption | 5 | 0.007843012 |
| Carbohydrate digestion and absorption | 12 | 0.197926563 | Linoleic acid metabolism | 0 | 1 | Caffeine metabolism | 2 | 0.008752885 |
| Basal cell carcinoma | 14 | 0.199739109 | alpha-Linolenic acid metabolism | 0 | 1 | Dorso-ventral axis formation | 4 | 0.009807765 |
| Gastric acid secretion | 18 | 0.216494491 | Glycosphingolipid biosynthesis - lacto and neolacto series | 0 | 1 | Vascular smooth muscle contraction | 9 | 0.011155862 |
| Citrate cycle (TCA cycle) | 8 | 0.241861221 | Glycosphingolipid biosynthesis - globo series | 0 | 1 | Retrograde endocannabinoid signaling | 8 | 0.012008516 |
| Endocytosis | 57 | 0.246051535 | Glycosphingolipid biosynthesis - ganglio series | 0 | 1 | Asthma | 4 | 0.01251661 |
| Tyrosine metabolism | 9 | 0.257936539 | Pyruvate metabolism | 0 | 1 | Drug metabolism - other enzymes | 5 | 0.012680885 |
| Steroid hormone biosynthesis | 14 | 0.263626129 | Glyoxylate and dicarboxylate metabolism | 0 | 1 | Renin secretion | 6 | 0.013151809 |
| Base excision repair | 8 | 0.337689347 | Propanoate metabolism | 0 | 1 | cGMP-PKG signaling pathway | 11 | 0.013423218 |
| Protein digestion and absorption | 20 | 0.343540837 | Butanoate metabolism | 0 | 1 | Phenylalanine metabolism | 3 | 0.013975099 |
| Circadian entrainment | 21 | 0.347706209 | One carbon pool by folate | 0 | 1 | Metabolic pathways | 51 | 0.0158586 |
| Glucagon signaling pathway | 22 | 0.370718058 | Thiamine metabolism | 0 | 1 | Hippo signaling pathway | 10 | 0.018866702 |
| Antigen processing and presentation | 17 | 0.372559668 | Riboflavin metabolism | 0 | 1 | Thyroid hormone synthesis | 6 | 0.021069352 |
| Taurine and hypotaurine metabolism | 3 | 0.384776504 | Vitamin B6 metabolism | 0 | 1 | Arginine biosynthesis | 3 | 0.024989808 |
| Cardiac muscle contraction | 17 | 0.394728267 | Nicotinate and nicotinamide metabolism | 0 | 1 | Circadian entrainment | 7 | 0.026042433 |
| Circadian rhythm | 7 | 0.396512581 | Pantothenate and CoA biosynthesis | 0 | 1 | Regulation of lipolysis in adipocytes | 5 | 0.027654921 |
| Fanconi anemia pathway | 12 | 0.398130106 | Biotin metabolism | 0 | 1 | Cell adhesion molecules (CAMs) | 9 | 0.031157965 |
| Arginine biosynthesis | 5 | 0.416997421 | Lipoic acid metabolism | 0 | 1 | Tryptophan metabolism | 4 | 0.032948105 |
| Complement and coagulation cascades | 17 | 0.417025263 | Folate biosynthesis | 0 | 1 | Adrenergic signaling in cardiomyocytes | 9 | 0.037609219 |
| Salivary secretion | 19 | 0.422377111 | Retinol metabolism | 0 | 1 | Taurine and hypotaurine metabolism | 2 | 0.042655607 |
| Hedgehog signaling pathway | 10 | 0.445940477 | Porphyrin and chlorophyll metabolism | 0 | 1 | ABC transporters | 4 | 0.044558573 |
| Phenylalanine metabolism | 4 | 0.453909053 | Terpenoid backbone biosynthesis | 0 | 1 | Calcium signaling pathway | 10 | 0.047879745 |
| Aldosterone synthesis and secretion | 17 | 0.461714674 | Nitrogen metabolism | 0 | 1 | Alcoholism | 10 | 0.047879745 |
| Pantothenate and CoA biosynthesis | 4 | 0.501879812 | Sulfur metabolism | 0 | 1 | GABAergic synapse | 6 | 0.052194041 |
| Cytosolic DNA-sensing pathway | 13 | 0.505219696 | Aminoacyl-tRNA biosynthesis | 0 | 1 | Linoleic acid metabolism | 3 | 0.057490786 |
| ABC transporters | 9 | 0.534373167 | Metabolism of xenobiotics by cytochrome P450 | 0 | 1 | Tight junction | 8 | 0.062878792 |
| Insulin secretion | 17 | 0.54956299 | Drug metabolism - cytochrome P450 | 0 | 1 | Inositol phosphate metabolism | 5 | 0.065539142 |
| Staphylococcus aureus infection | 11 | 0.558751968 | Drug metabolism - other enzymes | 0 | 1 | Phosphatidylinositol signaling system | 6 | 0.079226577 |
| Cysteine and methionine metabolism | 9 | 0.563883104 | Biosynthesis of unsaturated fatty acids | 0 | 1 | Bacterial invasion of epithelial cells | 5 | 0.089857128 |
| Tryptophan metabolism | 8 | 0.567184252 | Carbon metabolism | 0 | 1 | Alanine, aspartate and glutamate metabolism | 3 | 0.090289816 |
| Bile secretion | 14 | 0.576685195 | 2-Oxocarboxylic acid metabolism | 0 | 1 | Endocytosis | 12 | 0.101775131 |
| One carbon pool by folate | 4 | 0.591576893 | Fatty acid metabolism | 0 | 1 | Other glycan degradation | 2 | 0.103293421 |
| Riboflavin metabolism | 1 | 0.591799798 | Biosynthesis of amino acids | 0 | 1 | Peroxisome | 5 | 0.109710382 |
| Alcoholism | 35 | 0.598594183 | EGFR tyrosine kinase inhibitor resistance | 0 | 1 | Riboflavin metabolism | 1 | 0.1167341 |
| Alzheimer's disease | 32 | 0.660417357 | Endocrine resistance | 0 | 1 | Dilated cardiomyopathy | 5 | 0.13607814 |
| Arginine and proline metabolism | 9 | 0.672717431 | Platinum drug resistance | 0 | 1 | Retinol metabolism | 4 | 0.136885418 |
| Butirosin and neomycin biosynthesis | 1 | 0.673738944 | Ribosome biogenesis in eukaryotes | 0 | 1 | Phenylalanine, tyrosine and tryptophan biosynthesis | 1 | 0.143729403 |
| Ubiquinone and other terpenoid-quinone biosynthesis | 2 | 0.679951337 | Ribosome | 0 | 1 | Butirosin and neomycin biosynthesis | 1 | 0.143729403 |
| Mismatch repair | 4 | 0.706414228 | RNA transport | 0 | 1 | Glycolysis / Gluconeogenesis | 4 | 0.148087531 |
| PPAR signaling pathway | 13 | 0.710431066 | mRNA surveillance pathway | 0 | 1 | Mismatch repair | 2 | 0.15495129 |
| Pentose phosphate pathway | 5 | 0.719332233 | RNA degradation | 0 | 1 | Renin-angiotensin system | 2 | 0.15495129 |
| Vibrio cholerae infection | 9 | 0.720525536 | RNA polymerase | 0 | 1 | Hedgehog signaling pathway | 3 | 0.165182131 |
| 2-Oxocarboxylic acid metabolism | 3 | 0.73114266 | Basal transcription factors | 0 | 1 | Histidine metabolism | 2 | 0.165804077 |
| Glutathione metabolism | 9 | 0.742621266 | DNA replication | 0 | 1 | Pancreatic secretion | 5 | 0.170000328 |
| Nucleotide excision repair | 8 | 0.753499743 | Spliceosome | 0 | 1 | Sphingolipid metabolism | 3 | 0.172722836 |
| Endocrine and other factor-regulated calcium reabsorption | 8 | 0.753499743 | Proteasome | 0 | 1 | Endocrine and other factor-regulated calcium reabsorption | 3 | 0.172722836 |
| Pancreatic secretion | 17 | 0.756298141 | Protein export | 0 | 1 | alpha-Linolenic acid metabolism | 2 | 0.176784127 |
| Oocyte meiosis | 22 | 0.760985629 | Base excision repair | 0 | 1 | Maturity onset diabetes of the young | 2 | 0.187873614 |
| Biosynthesis of amino acids | 13 | 0.765526228 | Nucleotide excision repair | 0 | 1 | Gastric acid secretion | 4 | 0.189832132 |
| Phosphatidylinositol signaling system | 17 | 0.786085929 | Mismatch repair | 0 | 1 | Ascorbate and aldarate metabolism | 2 | 0.199055674 |
| Glycosaminoglycan biosynthesis - chondroitin sulfate / dermatan sulfate | 3 | 0.796193932 | Homologous recombination | 0 | 1 | Glyoxylate and dicarboxylate metabolism | 2 | 0.210314366 |
| Folate biosynthesis | 2 | 0.803914565 | Non-homologous end-joining | 0 | 1 | Butanoate metabolism | 2 | 0.210314366 |
| Vasopressin-regulated water reabsorption | 7 | 0.80788171 | Fanconi anemia pathway | 0 | 1 | Mineral absorption | 3 | 0.211743887 |
| Carbon metabolism | 19 | 0.83789601 | ErbB signaling pathway | 0 | 1 | Complement and coagulation cascades | 4 | 0.221670008 |
| Regulation of autophagy | 6 | 0.84160989 | Cytokine-cytokine receptor interaction | 0 | 1 | Circadian rhythm | 2 | 0.233002254 |
| Hippo signaling pathway -multiple species | 4 | 0.842192409 | Chemokine signaling pathway | 0 | 1 | Pathogenic Escherichia coli infection | 3 | 0.236002455 |
| Drug metabolism - other enzymes | 7 | 0.843676179 | FoxO signaling pathway | 0 | 1 | Staphylococcus aureus infection | 3 | 0.236002455 |
| Nicotinate and nicotinamide metabolism | 4 | 0.861850037 | Phospholipase D signaling pathway | 0 | 1 | ECM-receptor interaction | 4 | 0.241408432 |
| Inositol phosphate metabolism | 11 | 0.869384253 | Cell cycle | 0 | 1 | beta-Alanine metabolism | 2 | 0.244403823 |
| Notch signaling pathway | 7 | 0.873913161 | Ubiquitin mediated proteolysis | 0 | 1 | Carbon metabolism | 5 | 0.263352928 |
| Primary bile acid biosynthesis | 2 | 0.883231371 | Sulfur relay system | 0 | 1 | Fructose and mannose metabolism | 2 | 0.267258983 |
| Nitrogen metabolism | 2 | 0.883231371 | SNARE interactions in vesicular transport | 0 | 1 | Base excision repair | 2 | 0.267258983 |
| Histidine metabolism | 3 | 0.887222387 | Regulation of autophagy | 0 | 1 | Glycerolipid metabolism | 3 | 0.269026682 |
| Vitamin digestion and absorption | 3 | 0.887222387 | Protein processing in endoplasmic reticulum | 0 | 1 | Glutamatergic synapse | 5 | 0.26919983 |
| Sulfur metabolism | 1 | 0.893618148 | Endocytosis | 0 | 1 | Axon guidance | 7 | 0.292349358 |
| Galactose metabolism | 4 | 0.894931399 | Phagosome | 0 | 1 | Pentose and glucuronate interconversions | 2 | 0.301503578 |
| beta-Alanine metabolism | 4 | 0.894931399 | Peroxisome | 0 | 1 | Fatty acid biosynthesis | 1 | 0.332086815 |
| Other types of O-glycan biosynthesis | 4 | 0.894931399 | mTOR signaling pathway | 0 | 1 | Glycine, serine and threonine metabolism | 2 | 0.346688316 |
| Parkinson's disease | 23 | 0.899539446 | Apoptosis | 0 | 1 | Folate biosynthesis | 1 | 0.352518011 |
| Ubiquitin mediated proteolysis | 22 | 0.903618434 | Longevity regulating pathway - multiple species | 0 | 1 | Porphyrin and chlorophyll metabolism | 2 | 0.368932736 |
| Mineral absorption | 7 | 0.919936661 | Apoptosis - multiple species | 0 | 1 | Glycosaminoglycan biosynthesis - keratan sulfate | 1 | 0.372326136 |
| Fructose and mannose metabolism | 4 | 0.920835534 | Wnt signaling pathway | 0 | 1 | Glycosphingolipid biosynthesis - ganglio series | 1 | 0.372326136 |
| Glycine, serine and threonine metabolism | 5 | 0.925861823 | Dorso-ventral axis formation | 0 | 1 | Vasopressin-regulated water reabsorption | 2 | 0.390874459 |
| Phototransduction | 3 | 0.929552892 | Notch signaling pathway | 0 | 1 | Protein processing in endoplasmic reticulum | 6 | 0.391335833 |
| Butanoate metabolism | 3 | 0.94003615 | TGF-beta signaling pathway | 0 | 1 | Cysteine and methionine metabolism | 2 | 0.401717775 |
| Metabolism of xenobiotics by cytochrome P450 | 10 | 0.940206283 | Axon guidance | 0 | 1 | Ether lipid metabolism | 2 | 0.401717775 |
| Alanine, aspartate and glutamate metabolism | 4 | 0.940856015 | VEGF signaling pathway | 0 | 1 | Antigen processing and presentation | 3 | 0.419571785 |
| Fatty acid biosynthesis | 1 | 0.945709654 | Hippo signaling pathway -multiple species | 0 | 1 | 2-Oxocarboxylic acid metabolism | 1 | 0.428198662 |
| Non-homologous end-joining | 1 | 0.945709654 | Focal adhesion | 0 | 1 | Fatty acid metabolism | 2 | 0.433679821 |
| Linoleic acid metabolism | 3 | 0.949057624 | ECM-receptor interaction | 0 | 1 | Glycosaminoglycan degradation | 1 | 0.445698278 |
| Terpenoid backbone biosynthesis | 2 | 0.95289487 | Adherens junction | 0 | 1 | Terpenoid backbone biosynthesis | 1 | 0.495058036 |
| Retrograde endocannabinoid signaling | 14 | 0.959533525 | Tight junction | 0 | 1 | Phagosome | 5 | 0.50348227 |
| Thyroid hormone synthesis | 9 | 0.96259497 | Signaling pathways regulating pluripotency of stem cells | 0 | 1 | Basal cell carcinoma | 2 | 0.504472547 |
| Glycosaminoglycan biosynthesis - keratan sulfate | 1 | 0.965333767 | Antigen processing and presentation | 0 | 1 | Proximal tubule bicarbonate reclamation | 1 | 0.510517434 |
| Sphingolipid metabolism | 5 | 0.972210581 | Toll-like receptor signaling pathway | 0 | 1 | Salivary secretion | 3 | 0.514488788 |
| Maturity onset diabetes of the young | 2 | 0.977848492 | NOD-like receptor signaling pathway | 0 | 1 | Glycosaminoglycan biosynthesis - heparan sulfate / heparin | 1 | 0.525504971 |
| Selenocompound metabolism | 1 | 0.97786658 | RIG-I-like receptor signaling pathway | 0 | 1 | Morphine addiction | 3 | 0.529512851 |
| SNARE interactions in vesicular transport | 3 | 0.978021093 | Cytosolic DNA-sensing pathway | 0 | 1 | Glycosphingolipid biosynthesis - lacto and neolacto series | 1 | 0.554121462 |
| Chemical carcinogenesis | 10 | 0.978357264 | Jak-STAT signaling pathway | 0 | 1 | Glycerophospholipid metabolism | 3 | 0.55876673 |
| Porphyrin and chlorophyll metabolism | 4 | 0.97984983 | Hematopoietic cell lineage | 0 | 1 | Phototransduction | 1 | 0.567777839 |
| Taste transduction | 10 | 0.980791938 | Natural killer cell mediated cytotoxicity | 0 | 1 | Systemic lupus erythematosus | 4 | 0.58922949 |
| Starch and sucrose metabolism | 6 | 0.982177368 | T cell receptor signaling pathway | 0 | 1 | Pentose phosphate pathway | 1 | 0.593852319 |
| Huntington's disease | 28 | 0.982219668 | B cell receptor signaling pathway | 0 | 1 | Homologous recombination | 1 | 0.593852319 |
| Other glycan degradation | 1 | 0.982315058 | Fc epsilon RI signaling pathway | 0 | 1 | Citrate cycle (TCA cycle) | 1 | 0.606295425 |
| Systemic lupus erythematosus | 18 | 0.983010315 | Fc gamma R-mediated phagocytosis | 0 | 1 | Ubiquitin mediated proteolysis | 4 | 0.606783864 |
| Protein processing in endoplasmic reticulum | 23 | 0.984407101 | TNF signaling pathway | 0 | 1 | Propanoate metabolism | 1 | 0.630053052 |
| Glyoxylate and dicarboxylate metabolism | 2 | 0.984917985 | Leukocyte transendothelial migration | 0 | 1 | Biosynthesis of amino acids | 2 | 0.672855584 |
| Lysine degradation | 5 | 0.986845475 | Intestinal immune network for IgA production | 0 | 1 | DNA replication | 1 | 0.67336592 |
| GABAergic synapse | 10 | 0.9896059 | Circadian rhythm | 0 | 1 | Primary immunodeficiency | 1 | 0.67336592 |
| Drug metabolism - cytochrome P450 | 7 | 0.991300254 | Synaptic vesicle cycle | 0 | 1 | Pyruvate metabolism | 1 | 0.711621891 |
| Peroxisome | 9 | 0.991621542 | Ovarian steroidogenesis | 0 | 1 | Regulation of autophagy | 1 | 0.711621891 |
| Amino sugar and nucleotide sugar metabolism | 4 | 0.992432419 | Progesterone-mediated oocyte maturation | 0 | 1 | Lysosome | 3 | 0.730419486 |
| Morphine addiction | 10 | 0.99290303 | Prolactin signaling pathway | 0 | 1 | Fatty acid degradation | 1 | 0.745409722 |
| Fat digestion and absorption | 3 | 0.993600923 | Thyroid hormone signaling pathway | 0 | 1 | Nucleotide excision repair | 1 | 0.768133775 |
| Protein export | 1 | 0.994242672 | Adipocytokine signaling pathway | 0 | 1 | Valine, leucine and isoleucine degradation | 1 | 0.775249817 |
| Proximal tubule bicarbonate reclamation | 1 | 0.994242672 | Regulation of lipolysis in adipocytes | 0 | 1 | Amino sugar and nucleotide sugar metabolism | 1 | 0.775249817 |
| Glutamatergic synapse | 13 | 0.995192759 | Insulin resistance | 0 | 1 | Notch signaling pathway | 1 | 0.775249817 |
| Glycosaminoglycan biosynthesis - heparan sulfate / heparin | 1 | 0.995400477 | Non-alcoholic fatty liver disease (NAFLD) | 0 | 1 | Vibrio cholerae infection | 1 | 0.795317951 |
| Glycolysis / Gluconeogenesis | 6 | 0.995957069 | Type I diabetes mellitus | 0 | 1 | Fanconi anemia pathway | 1 | 0.813599331 |
| Glycosylphosphatidylinositol(GPI)-anchor biosynthesis | 1 | 0.996325535 | Maturity onset diabetes of the young | 0 | 1 | Parkinson's disease | 3 | 0.813794504 |
| alpha-Linolenic acid metabolism | 1 | 0.996325535 | Endocrine and other factor-regulated calcium reabsorption | 0 | 1 | Neuroactive ligand-receptor interaction | 6 | 0.855187213 |
| Lysosome | 14 | 0.996420318 | Vasopressin-regulated water reabsorption | 0 | 1 | Aminoacyl-tRNA biosynthesis | 1 | 0.871826491 |
| Pyrimidine metabolism | 11 | 0.996847742 | Proximal tubule bicarbonate reclamation | 0 | 1 | Arrhythmogenic right ventricular cardiomyopathy (ARVC) | 1 | 0.900170564 |
| Pentose and glucuronate interconversions | 2 | 0.996873608 | Collecting duct acid secretion | 0 | 1 | RNA degradation | 1 | 0.909106218 |
| DNA replication | 2 | 0.996873608 | Protein digestion and absorption | 0 | 1 | Ribosome biogenesis in eukaryotes | 1 | 0.924655922 |
| Ether lipid metabolism | 3 | 0.996911196 | Fat digestion and absorption | 0 | 1 | Taste transduction | 1 | 0.924655922 |
| Basal transcription factors | 3 | 0.996911196 | Vitamin digestion and absorption | 0 | 1 | Protein digestion and absorption | 1 | 0.939476752 |
| Glycosphingolipid biosynthesis - lacto and neolacto series | 1 | 0.997064617 | Mineral absorption | 0 | 1 | mRNA surveillance pathway | 1 | 0.941341993 |
| Arachidonic acid metabolism | 5 | 0.997310064 | Amyotrophic lateral sclerosis (ALS) | 0 | 1 | Oxidative phosphorylation | 1 | 0.984291861 |
| N-Glycan biosynthesis | 3 | 0.998529348 | Prion diseases | 0 | 1 | Spliceosome | 1 | 0.984777996 |
| Pyruvate metabolism | 2 | 0.998600367 | Nicotine addiction | 0 | 1 | RNA transport | 1 | 0.995253645 |
| Nicotine addiction | 2 | 0.998600367 | Bacterial invasion of epithelial cells | 0 | 1 | Purine metabolism | 1 | 0.9956824 |
| Glycerolipid metabolism | 4 | 0.998862965 | Vibrio cholerae infection | 0 | 1 | Olfactory transduction | 1 | 0.999998106 |
| Propanoate metabolism | 1 | 0.999237453 | Epithelial cell signaling in Helicobacter pylori infection | 0 | 1 | Fatty acid elongation | 0 | 1 |
| Proteasome | 2 | 0.999378832 | Pathogenic Escherichia coli infection | 0 | 1 | Synthesis and degradation of ketone bodies | 0 | 1 |
| Synaptic vesicle cycle | 4 | 0.999443539 | Shigellosis | 0 | 1 | Primary bile acid biosynthesis | 0 | 1 |
| Glycerophospholipid metabolism | 8 | 0.999458639 | Salmonella infection | 0 | 1 | Ubiquinone and other terpenoid-quinone biosynthesis | 0 | 1 |
| Aminoacyl-tRNA biosynthesis | 4 | 0.999676791 | Legionellosis | 0 | 1 | Pyrimidine metabolism | 0 | 1 |
| Retinol metabolism | 3 | 0.999931887 | Leishmaniasis | 0 | 1 | Valine, leucine and isoleucine biosynthesis | 0 | 1 |
| Spliceosome | 11 | 0.999955829 | African trypanosomiasis | 0 | 1 | Lysine biosynthesis | 0 | 1 |
| RNA degradation | 4 | 0.999957893 | Toxoplasmosis | 0 | 1 | Lysine degradation | 0 | 1 |
| Purine metabolism | 16 | 0.999972868 | Amoebiasis | 0 | 1 | Selenocompound metabolism | 0 | 1 |
| Valine, leucine and isoleucine degradation | 1 | 0.999979139 | Hepatitis C | 0 | 1 | D-Glutamine and D-glutamate metabolism | 0 | 1 |
| Fatty acid metabolism | 1 | 0.999979139 | Hepatitis B | 0 | 1 | D-Arginine and D-ornithine metabolism | 0 | 1 |
| mRNA surveillance pathway | 3 | 0.999999633 | Measles | 0 | 1 | N-Glycan biosynthesis | 0 | 1 |
| Oxidative phosphorylation | 7 | 0.999999787 | HTLV-I infection | 0 | 1 | Mucin type O-Glycan biosynthesis | 0 | 1 |
| Ribosome biogenesis in eukaryotes | 2 | 0.999999829 | Herpes simplex infection | 0 | 1 | Other types of O-glycan biosynthesis | 0 | 1 |
| RNA transport | 11 | 0.999999886 | Epstein-Barr virus infection | 0 | 1 | Glycosaminoglycan biosynthesis - chondroitin sulfate / dermatan sulfate | 0 | 1 |
| Olfactory transduction | 6 | 1 | Viral carcinogenesis | 0 | 1 | Glycosylphosphatidylinositol(GPI)-anchor biosynthesis | 0 | 1 |
| Metabolic pathways | 127 | 1 | Chemical carcinogenesis | 0 | 1 | Glycosphingolipid biosynthesis - globo series | 0 | 1 |
| Ribosome | 1 | 1 | Colorectal cancer | 0 | 1 | One carbon pool by folate | 0 | 1 |
| Ascorbate and aldarate metabolism | 0 | 1 | Renal cell carcinoma | 0 | 1 | Thiamine metabolism | 0 | 1 |
| Fatty acid elongation | 0 | 1 | Pancreatic cancer | 0 | 1 | Vitamin B6 metabolism | 0 | 1 |
| Fatty acid degradation | 0 | 1 | Endometrial cancer | 0 | 1 | Nicotinate and nicotinamide metabolism | 0 | 1 |
| Synthesis and degradation of ketone bodies | 0 | 1 | Prostate cancer | 0 | 1 | Pantothenate and CoA biosynthesis | 0 | 1 |
| Steroid biosynthesis | 0 | 1 | Basal cell carcinoma | 0 | 1 | Biotin metabolism | 0 | 1 |
| Valine, leucine and isoleucine biosynthesis | 0 | 1 | Melanoma | 0 | 1 | Lipoic acid metabolism | 0 | 1 |
| Lysine biosynthesis | 0 | 1 | Bladder cancer | 0 | 1 | Nitrogen metabolism | 0 | 1 |
| Phenylalanine, tyrosine and tryptophan biosynthesis | 0 | 1 | Chronic myeloid leukemia | 0 | 1 | Sulfur metabolism | 0 | 1 |
| D-Glutamine and D-glutamate metabolism | 0 | 1 | Acute myeloid leukemia | 0 | 1 | Biosynthesis of unsaturated fatty acids | 0 | 1 |
| D-Arginine and D-ornithine metabolism | 0 | 1 | Small cell lung cancer | 0 | 1 | Ribosome | 0 | 1 |
| Mucin type O-Glycan biosynthesis | 0 | 1 | Non-small cell lung cancer | 0 | 1 | RNA polymerase | 0 | 1 |
| Glycosaminoglycan degradation | 0 | 1 | Central carbon metabolism in cancer | 0 | 1 | Basal transcription factors | 0 | 1 |
| Glycosphingolipid biosynthesis - globo series | 0 | 1 | Choline metabolism in cancer | 0 | 1 | Proteasome | 0 | 1 |
| Glycosphingolipid biosynthesis - ganglio series | 0 | 1 | Asthma | 0 | 1 | Protein export | 0 | 1 |
| Thiamine metabolism | 0 | 1 | Autoimmune thyroid disease | 0 | 1 | Non-homologous end-joining | 0 | 1 |
| Vitamin B6 metabolism | 0 | 1 | Inflammatory bowel disease (IBD) | 0 | 1 | Sulfur relay system | 0 | 1 |
| Biotin metabolism | 0 | 1 | Systemic lupus erythematosus | 0 | 1 | SNARE interactions in vesicular transport | 0 | 1 |
| Lipoic acid metabolism | 0 | 1 | Rheumatoid arthritis | 0 | 1 | Cardiac muscle contraction | 0 | 1 |
| Biosynthesis of unsaturated fatty acids | 0 | 1 | Allograft rejection | 0 | 1 | Hippo signaling pathway -multiple species | 0 | 1 |
| RNA polymerase | 0 | 1 | Graft-versus-host disease | 0 | 1 | Synaptic vesicle cycle | 0 | 1 |
| Sulfur relay system | 0 | 1 | Primary immunodeficiency | 0 | 1 | Collecting duct acid secretion | 0 | 1 |
| Collecting duct acid secretion | 0 | 1 | Viral myocarditis | 0 | 1 | Nicotine addiction | 0 | 1 |

Pathways in M5

| **symptom** | **overlap** | **p_value** | **drug** | **overlap** | **p_value** | **herb** | **overlap** | **p_value** |
| --- | --- | --- | --- | --- | --- | --- | --- | --- |
| MicroRNAs in cancer | 185 | 2.59834E-48 | Neuroactive ligand-receptor interaction | 58 | 4.5433E-53 | Pathways in cancer | 81 | 1.4964E-32 |
| Pathways in cancer | 188 | 1.51704E-27 | Nicotine addiction | 21 | 6.54648E-29 | Hepatitis B | 50 | 1.61268E-31 |
| Non-small cell lung cancer | 49 | 1.46532E-24 | Calcium signaling pathway | 30 | 7.36549E-24 | AGE-RAGE signaling pathway in diabetic complications | 41 | 2.00999E-29 |
| Proteoglycans in cancer | 114 | 2.46521E-24 | Morphine addiction | 22 | 2.41506E-21 | FoxO signaling pathway | 42 | 5.3789E-25 |
| Prostate cancer | 65 | 1.3122E-23 | cAMP signaling pathway | 26 | 6.25275E-18 | Prostate cancer | 34 | 1.5772E-23 |
| Pancreatic cancer | 52 | 9.78946E-22 | GABAergic synapse | 19 | 1.4929E-17 | Endocrine resistance | 35 | 3.29809E-23 |
| Small cell lung cancer | 61 | 3.76831E-21 | Retrograde endocannabinoid signaling | 19 | 2.32942E-16 | PI3K-Akt signaling pathway | 61 | 1.92265E-21 |
| AGE-RAGE signaling pathway in diabetic complications | 67 | 1.47025E-20 | Serotonergic synapse | 19 | 1.75251E-15 | Apoptosis | 39 | 4.60628E-21 |
| EGFR tyrosine kinase inhibitor resistance | 58 | 1.67886E-20 | Taste transduction | 13 | 1.72042E-10 | Pancreatic cancer | 28 | 4.62234E-21 |
| Bladder cancer | 37 | 7.32072E-20 | cGMP-PKG signaling pathway | 16 | 2.30807E-09 | Platinum drug resistance | 29 | 2.08312E-20 |
| Colorectal cancer | 48 | 1.4027E-19 | Adrenergic signaling in cardiomyocytes | 13 | 2.20687E-07 | HTLV-I infection | 50 | 3.48651E-19 |
| Endocrine resistance | 63 | 1.06991E-18 | Renin secretion | 8 | 3.711E-06 | TNF signaling pathway | 33 | 4.37731E-19 |
| Hepatitis B | 82 | 3.35692E-18 | Salivary secretion | 9 | 5.43898E-06 | HIF-1 signaling pathway | 32 | 4.7202E-19 |
| Chronic myeloid leukemia | 51 | 1.92776E-17 | Cocaine addiction | 7 | 6.19081E-06 | Chagas disease (American trypanosomiasis) | 32 | 6.54223E-19 |
| Endometrial cancer | 41 | 2.01325E-17 | Cardiac muscle contraction | 8 | 1.65548E-05 | Toxoplasmosis | 33 | 4.78274E-18 |
| Focal adhesion | 101 | 4.13772E-17 | Hypertrophic cardiomyopathy (HCM) | 8 | 2.6193E-05 | Leishmaniasis | 26 | 1.51056E-17 |
| PI3K-Akt signaling pathway | 146 | 9.24014E-17 | Dilated cardiomyopathy | 8 | 4.35751E-05 | Proteoglycans in cancer | 42 | 3.49664E-17 |
| Glioma | 46 | 3.31158E-16 | Amphetamine addiction | 7 | 5.03634E-05 | EGFR tyrosine kinase inhibitor resistance | 27 | 3.82288E-17 |
| HIF-1 signaling pathway | 62 | 4.68874E-16 | Type II diabetes mellitus | 6 | 6.37389E-05 | Bladder cancer | 20 | 7.08838E-17 |
| FoxO signaling pathway | 73 | 1.28283E-15 | Arrhythmogenic right ventricular cardiomyopathy (ARVC) | 7 | 9.57349E-05 | Tuberculosis | 38 | 2.32877E-16 |
| Apoptosis | 75 | 3.04598E-15 | Regulation of lipolysis in adipocytes | 6 | 0.00015326 | Malaria | 21 | 3.28241E-16 |
| Melanoma | 47 | 9.21556E-15 | Steroid hormone biosynthesis | 6 | 0.000186545 | Rheumatoid arthritis | 27 | 5.73123E-16 |
| Toxoplasmosis | 65 | 3.73588E-14 | Cholinergic synapse | 8 | 0.000208119 | Colorectal cancer | 23 | 5.75226E-16 |
| ErbB signaling pathway | 53 | 6.19663E-14 | Insulin secretion | 7 | 0.000229824 | p53 signaling pathway | 24 | 7.35021E-16 |
| Prolactin signaling pathway | 46 | 1.28478E-13 | Alcoholism | 10 | 0.000279101 | Insulin resistance | 29 | 2.28079E-15 |
| Acute myeloid leukemia | 37 | 1.60894E-11 | Vascular smooth muscle contraction | 8 | 0.000354829 | MAPK signaling pathway | 44 | 4.83835E-15 |
| TNF signaling pathway | 57 | 3.85758E-11 | Bile secretion | 6 | 0.000565285 | T cell receptor signaling pathway | 28 | 6.57451E-15 |
| Chagas disease (American trypanosomiasis) | 54 | 1.13943E-10 | Dopaminergic synapse | 8 | 0.000576706 | Small cell lung cancer | 25 | 2.1387E-14 |
| Platinum drug resistance | 43 | 1.39851E-10 | Gap junction | 6 | 0.00174767 | Glioma | 22 | 2.33281E-14 |
| Neurotrophin signaling pathway | 59 | 2.73842E-10 | Butirosin and neomycin biosynthesis | 2 | 0.002044226 | Chronic myeloid leukemia | 23 | 3.47113E-14 |
| p53 signaling pathway | 40 | 3.88098E-10 | Oxytocin signaling pathway | 8 | 0.002054979 | Influenza A | 35 | 3.62E-14 |
| Central carbon metabolism in cancer | 39 | 5.44016E-10 | Circadian entrainment | 6 | 0.00257962 | Osteoclast differentiation | 30 | 5.95934E-14 |
| VEGF signaling pathway | 36 | 1.4847E-09 | Long-term potentiation | 5 | 0.002674645 | Prolactin signaling pathway | 22 | 2.57296E-13 |
| Cocaine addiction | 31 | 1.8584E-09 | Adipocytokine signaling pathway | 5 | 0.003458128 | Cell cycle | 28 | 6.09694E-13 |
| cAMP signaling pathway | 83 | 2.45538E-09 | MAPK signaling pathway | 10 | 0.004066121 | Pertussis | 22 | 6.54818E-13 |
| Fc epsilon RI signaling pathway | 38 | 2.51925E-09 | Carbohydrate digestion and absorption | 4 | 0.004376404 | Focal adhesion | 36 | 7.10525E-13 |
| Thyroid hormone signaling pathway | 56 | 4.09771E-09 | Gastric acid secretion | 5 | 0.004394465 | NF-kappa B signaling pathway | 24 | 9.91946E-13 |
| Amoebiasis | 49 | 6.68999E-09 | Glutamatergic synapse | 6 | 0.006324081 | Melanoma | 21 | 1.8464E-12 |
| Amyotrophic lateral sclerosis (ALS) | 31 | 7.59332E-09 | Amyotrophic lateral sclerosis (ALS) | 4 | 0.006337393 | Toll-like receptor signaling pathway | 25 | 3.86275E-12 |
| Renal cell carcinoma | 37 | 1.0994E-08 | Collecting duct acid secretion | 3 | 0.006841218 | Measles | 28 | 5.57939E-12 |
| Ras signaling pathway | 90 | 1.37511E-08 | Linoleic acid metabolism | 3 | 0.008367295 | VEGF signaling pathway | 19 | 7.66028E-12 |
| Thyroid cancer | 21 | 2.23029E-08 | Arachidonic acid metabolism | 4 | 0.012514627 | Non-small cell lung cancer | 18 | 1.51008E-11 |
| T cell receptor signaling pathway | 50 | 2.40108E-08 | Pancreatic secretion | 5 | 0.012941199 | Viral carcinogenesis | 34 | 2.21021E-11 |
| Glutamatergic synapse | 53 | 2.57123E-08 | Central carbon metabolism in cancer | 4 | 0.016266719 | Transcriptional misregulation in cancer | 31 | 5.58716E-11 |
| Hypertrophic cardiomyopathy (HCM) | 42 | 3.52051E-08 | Chagas disease (American trypanosomiasis) | 5 | 0.017764653 | African trypanosomiasis | 14 | 6.08435E-11 |
| Serotonergic synapse | 52 | 3.67538E-08 | Insulin resistance | 5 | 0.021314174 | MicroRNAs in cancer | 41 | 9.46322E-11 |
| Nicotine addiction | 25 | 9.97283E-08 | Other glycan degradation | 2 | 0.027635317 | Amyotrophic lateral sclerosis (ALS) | 16 | 3.13695E-10 |
| Amphetamine addiction | 35 | 1.75581E-07 | Aldosterone synthesis and secretion | 4 | 0.030249272 | Cytokine-cytokine receptor interaction | 37 | 4.86001E-10 |
| Rap1 signaling pathway | 81 | 2.93398E-07 | Chemical carcinogenesis | 4 | 0.031452491 | Apoptosis - multiple species | 13 | 5.79814E-10 |
| Arrhythmogenic right ventricular cardiomyopathy (ARVC) | 37 | 3.37114E-07 | Alzheimer's disease | 6 | 0.035967712 | AMPK signaling pathway | 24 | 7.10696E-10 |
| Neuroactive ligand-receptor interaction | 100 | 4.10029E-07 | Small cell lung cancer | 4 | 0.036544559 | Chemokine signaling pathway | 30 | 7.94879E-10 |
| Dilated cardiomyopathy | 41 | 1.29262E-06 | Arginine biosynthesis | 2 | 0.036872684 | Adipocytokine signaling pathway | 18 | 9.24228E-10 |
| Estrogen signaling pathway | 44 | 1.80428E-06 | Proximal tubule bicarbonate reclamation | 2 | 0.043595243 | B cell receptor signaling pathway | 18 | 1.51974E-09 |
| Inflammatory bowel disease (IBD) | 32 | 3.19997E-06 | Ras signaling pathway | 7 | 0.048874913 | Amoebiasis | 21 | 1.7033E-09 |
| HTLV-I infection | 91 | 4.16313E-06 | Maturity onset diabetes of the young | 2 | 0.054448898 | Ras signaling pathway | 33 | 1.72399E-09 |
| Pertussis | 35 | 5.30123E-06 | Inflammatory mediator regulation of TRP channels | 4 | 0.054514908 | NOD-like receptor signaling pathway | 16 | 1.97658E-09 |
| Osteoclast differentiation | 53 | 5.49085E-06 | Glucagon signaling pathway | 4 | 0.059635954 | Neurotrophin signaling pathway | 23 | 2.33439E-09 |
| Measles | 54 | 6.71134E-06 | Retinol metabolism | 3 | 0.068701181 | Central carbon metabolism in cancer | 17 | 3.40613E-09 |
| Longevity regulating pathway - multiple species | 31 | 6.98148E-06 | Toll-like receptor signaling pathway | 4 | 0.068719986 | ErbB signaling pathway | 19 | 7.51167E-09 |
| MAPK signaling pathway | 89 | 8.56982E-06 | Caffeine metabolism | 1 | 0.070690054 | Thyroid hormone signaling pathway | 22 | 8.94929E-09 |
| B cell receptor signaling pathway | 33 | 1.56995E-05 | Galactose metabolism | 2 | 0.07435404 | Legionellosis | 15 | 9.84812E-09 |
| Calcium signaling pathway | 66 | 1.71523E-05 | Drug metabolism - cytochrome P450 | 3 | 0.079089173 | Estrogen signaling pathway | 20 | 1.01945E-08 |
| Malaria | 25 | 1.71976E-05 | Vitamin B6 metabolism | 1 | 0.084220649 | Inflammatory bowel disease (IBD) | 16 | 1.5907E-08 |
| Apoptosis - multiple species | 19 | 1.91546E-05 | Rap1 signaling pathway | 6 | 0.087612324 | Epstein-Barr virus infection | 29 | 1.64767E-08 |
| ECM-receptor interaction | 36 | 2.13554E-05 | Metabolism of xenobiotics by cytochrome P450 | 3 | 0.090117687 | Ovarian steroidogenesis | 14 | 2.12517E-08 |
| African trypanosomiasis | 19 | 3.4083E-05 | Chronic myeloid leukemia | 3 | 0.090117687 | Salmonella infection | 18 | 3.13714E-08 |
| Type II diabetes mellitus | 24 | 3.88941E-05 | Toxoplasmosis | 4 | 0.093230979 | Epithelial cell signaling in Helicobacter pylori infection | 16 | 3.18777E-08 |
| Dopaminergic synapse | 50 | 3.93632E-05 | Purine metabolism | 5 | 0.109985029 | Endometrial cancer | 14 | 3.69283E-08 |
| Long-term depression | 28 | 4.55043E-05 | Prostate cancer | 3 | 0.139871928 | Non-alcoholic fatty liver disease (NAFLD) | 24 | 4.8906E-08 |
| Sphingolipid signaling pathway | 47 | 4.88676E-05 | Drug metabolism - other enzymes | 2 | 0.144143822 | Rap1 signaling pathway | 29 | 5.53997E-08 |
| Signaling pathways regulating pluripotency of stem cells | 53 | 7.72723E-05 | Insulin signaling pathway | 4 | 0.144478988 | PPAR signaling pathway | 16 | 7.57265E-08 |
| Toll-like receptor signaling pathway | 42 | 8.86339E-05 | Sphingolipid metabolism | 2 | 0.149184286 | Glucagon signaling pathway | 19 | 8.02448E-08 |
| Aldosterone-regulated sodium reabsorption | 20 | 0.00010853 | NF-kappa B signaling pathway | 3 | 0.15006817 | Metabolism of xenobiotics by cytochrome P450 | 16 | 9.30703E-08 |
| Influenza A | 62 | 0.000117877 | Amino sugar and nucleotide sugar metabolism | 2 | 0.154260035 | Jak-STAT signaling pathway | 24 | 1.18771E-07 |
| Leishmaniasis | 31 | 0.00012197 | Ovarian steroidogenesis | 2 | 0.164509139 | Renal cell carcinoma | 15 | 1.7474E-07 |
| Jak-STAT signaling pathway | 57 | 0.000125311 | Mineral absorption | 2 | 0.174875162 | Insulin signaling pathway | 22 | 1.92077E-07 |
| Cell cycle | 47 | 0.000125437 | HIF-1 signaling pathway | 3 | 0.189304386 | Drug metabolism - cytochrome P450 | 15 | 2.63879E-07 |
| Choline metabolism in cancer | 40 | 0.000130782 | Starch and sucrose metabolism | 2 | 0.201204051 | Allograft rejection | 11 | 3.61876E-07 |
| Regulation of actin cytoskeleton | 73 | 0.000145991 | NOD-like receptor signaling pathway | 2 | 0.201204051 | Hepatitis C | 21 | 3.80391E-07 |
| Leukocyte transendothelial migration | 45 | 0.000146774 | Acute myeloid leukemia | 2 | 0.201204051 | Oxytocin signaling pathway | 23 | 4.25598E-07 |
| NF-kappa B signaling pathway | 37 | 0.000159214 | Regulation of actin cytoskeleton | 5 | 0.204068433 | Intestinal immune network for IgA production | 12 | 6.63385E-07 |
| Adipocytokine signaling pathway | 30 | 0.000172534 | TNF signaling pathway | 3 | 0.215508841 | Longevity regulating pathway | 17 | 6.98343E-07 |
| Hepatitis C | 49 | 0.000204214 | Primary bile acid biosynthesis | 1 | 0.22073915 | Type II diabetes mellitus | 12 | 8.49292E-07 |
| Adherens junction | 31 | 0.000223243 | Nitrogen metabolism | 1 | 0.22073915 | Acute myeloid leukemia | 13 | 9.28643E-07 |
| Longevity regulating pathway | 37 | 0.000266227 | 2-Oxocarboxylic acid metabolism | 1 | 0.232098199 | Leukocyte transendothelial migration | 19 | 1.00589E-06 |
| Cholinergic synapse | 42 | 0.000293178 | Cytosolic DNA-sensing pathway | 2 | 0.233337741 | Steroid hormone biosynthesis | 13 | 1.14908E-06 |
| Long-term potentiation | 28 | 0.000344529 | Shigellosis | 2 | 0.244126719 | Progesterone-mediated oocyte maturation | 17 | 1.28586E-06 |
| Hematopoietic cell lineage | 34 | 0.000541919 | Thyroid hormone signaling pathway | 3 | 0.246318805 | Platelet activation | 19 | 1.69394E-06 |
| Hippo signaling pathway | 53 | 0.000660716 | Pancreatic cancer | 2 | 0.249529548 | Prion diseases | 10 | 1.86875E-06 |
| Insulin resistance | 40 | 0.000824086 | Glycolysis / Gluconeogenesis | 2 | 0.254936215 | Type I diabetes mellitus | 11 | 1.91009E-06 |
| Melanogenesis | 37 | 0.001066075 | Epithelial cell signaling in Helicobacter pylori infection | 2 | 0.260345472 | GnRH signaling pathway | 16 | 2.19284E-06 |
| mTOR signaling pathway | 52 | 0.001209391 | AMPK signaling pathway | 3 | 0.26591801 | Herpes simplex infection | 24 | 2.26277E-06 |
| Retrograde endocannabinoid signaling | 37 | 0.001315797 | RIG-I-like receptor signaling pathway | 2 | 0.271166944 | Chemical carcinogenesis | 15 | 2.7217E-06 |
| Phospholipase D signaling pathway | 49 | 0.001352642 | PPAR signaling pathway | 2 | 0.281984675 | Adherens junction | 14 | 3.86774E-06 |
| Viral myocarditis | 24 | 0.001360158 | B cell receptor signaling pathway | 2 | 0.281984675 | Inflammatory mediator regulation of TRP channels | 16 | 5.98553E-06 |
| Legionellosis | 23 | 0.001434729 | Prolactin signaling pathway | 2 | 0.281984675 | Fc epsilon RI signaling pathway | 13 | 6.40274E-06 |
| Gap junction | 33 | 0.001493933 | Leishmaniasis | 2 | 0.281984675 | Viral myocarditis | 12 | 7.24479E-06 |
| Viral carcinogenesis | 65 | 0.002430154 | Renin-angiotensin system | 1 | 0.286472818 | Graft-versus-host disease | 10 | 8.97058E-06 |
| GnRH signaling pathway | 33 | 0.002824237 | Osteoclast differentiation | 3 | 0.297630648 | Autoimmune thyroid disease | 11 | 1.39499E-05 |
| Natural killer cell mediated cytotoxicity | 45 | 0.003237813 | FoxO signaling pathway | 3 | 0.305601741 | Glutathione metabolism | 11 | 1.39499E-05 |
| Epithelial cell signaling in Helicobacter pylori infection | 26 | 0.00333546 | Hepatitis C | 3 | 0.305601741 | Caffeine metabolism | 4 | 1.87239E-05 |
| Wnt signaling pathway | 47 | 0.003669213 | alpha-Linolenic acid metabolism | 1 | 0.307135817 | Longevity regulating pathway - multiple species | 12 | 2.09236E-05 |
| Rheumatoid arthritis | 32 | 0.003778558 | Huntington's disease | 4 | 0.30923765 | Sphingolipid signaling pathway | 17 | 2.16059E-05 |
| Chemokine signaling pathway | 59 | 0.004229213 | Measles | 3 | 0.313582373 | Amphetamine addiction | 12 | 3.38389E-05 |
| Axon guidance | 56 | 0.004289362 | Parkinson's disease | 3 | 0.341537917 | Galactose metabolism | 8 | 4.63476E-05 |
| Regulation of lipolysis in adipocytes | 22 | 0.004558589 | Thyroid cancer | 1 | 0.346695219 | Alzheimer's disease | 20 | 5.56899E-05 |
| Progesterone-mediated oocyte maturation | 34 | 0.00552602 | Pathways in cancer | 7 | 0.356965724 | cAMP signaling pathway | 22 | 7.44568E-05 |
| TGF-beta signaling pathway | 30 | 0.005529019 | Protein digestion and absorption | 2 | 0.377886292 | Arachidonic acid metabolism | 11 | 7.84934E-05 |
| NOD-like receptor signaling pathway | 22 | 0.005840768 | GnRH signaling pathway | 2 | 0.383082775 | TGF-beta signaling pathway | 13 | 7.91488E-05 |
| GABAergic synapse | 31 | 0.006102311 | Fructose and mannose metabolism | 1 | 0.384010064 | Natural killer cell mediated cytotoxicity | 17 | 9.03735E-05 |
| Transcriptional misregulation in cancer | 56 | 0.006343798 | African trypanosomiasis | 1 | 0.393003052 | Oocyte meiosis | 16 | 0.000107274 |
| Oxytocin signaling pathway | 50 | 0.006566756 | Glycerophospholipid metabolism | 2 | 0.403679351 | Choline metabolism in cancer | 14 | 0.00014473 |
| Insulin signaling pathway | 45 | 0.00692932 | Aldosterone-regulated sodium reabsorption | 1 | 0.436048671 | Thyroid cancer | 7 | 0.00022025 |
| Prion diseases | 15 | 0.007122321 | Tryptophan metabolism | 1 | 0.444286714 | Aldosterone-regulated sodium reabsorption | 8 | 0.000264162 |
| Shigellosis | 24 | 0.007783519 | Pyruvate metabolism | 1 | 0.444286714 | Insulin secretion | 12 | 0.00035909 |
| Basal cell carcinoma | 21 | 0.008053961 | Fat digestion and absorption | 1 | 0.452405204 | Serotonergic synapse | 14 | 0.000433655 |
| Salmonella infection | 30 | 0.008077165 | T cell receptor signaling pathway | 2 | 0.453672478 | Melanogenesis | 13 | 0.000470571 |
| Epstein-Barr virus infection | 61 | 0.008307949 | Cysteine and methionine metabolism | 1 | 0.483717729 | Gap junction | 12 | 0.000496801 |
| Caffeine metabolism | 4 | 0.011408119 | Ether lipid metabolism | 1 | 0.483717729 | Shigellosis | 10 | 0.000549631 |
| Tryptophan metabolism | 16 | 0.011969851 | Carbon metabolism | 2 | 0.491917371 | Signaling pathways regulating pluripotency of stem cells | 16 | 0.000566425 |
| Tuberculosis | 54 | 0.012385666 | Endocrine and other factor-regulated calcium reabsorption | 1 | 0.498700327 | Wnt signaling pathway | 16 | 0.000566425 |
| Non-alcoholic fatty liver disease (NAFLD) | 47 | 0.01286761 | Notch signaling pathway | 1 | 0.506028844 | Dopaminergic synapse | 15 | 0.000599126 |
| Bacterial invasion of epithelial cells | 27 | 0.012927071 | Herpes simplex infection | 3 | 0.507234208 | Carbohydrate digestion and absorption | 8 | 0.000852474 |
| Pathogenic Escherichia coli infection | 20 | 0.017198634 | Arginine and proline metabolism | 1 | 0.513250924 | Aldosterone synthesis and secretion | 11 | 0.000874712 |
| Inflammatory mediator regulation of TRP channels | 32 | 0.018050443 | Malaria | 1 | 0.513250924 | Bile secretion | 10 | 0.001117352 |
| Tyrosine metabolism | 14 | 0.018178994 | Vibrio cholerae infection | 1 | 0.527381896 | Fatty acid metabolism | 8 | 0.001140451 |
| Circadian entrainment | 31 | 0.019909945 | Glutathione metabolism | 1 | 0.534293792 | Long-term depression | 9 | 0.001243288 |
| Fc gamma R-mediated phagocytosis | 30 | 0.021968597 | Epstein-Barr virus infection | 3 | 0.560043751 | mTOR signaling pathway | 16 | 0.001274635 |
| Asthma | 12 | 0.027812619 | Systemic lupus erythematosus | 2 | 0.583944561 | Cocaine addiction | 8 | 0.001310976 |
| Arginine biosynthesis | 9 | 0.034365468 | VEGF signaling pathway | 1 | 0.592151402 | Hematopoietic cell lineage | 11 | 0.001590508 |
| D-Glutamine and D-glutamate metabolism | 3 | 0.040266404 | Apoptosis | 2 | 0.607899141 | Tryptophan metabolism | 7 | 0.00172274 |
| Complement and coagulation cascades | 25 | 0.048382661 | Longevity regulating pathway - multiple species | 1 | 0.6098035 | Arginine biosynthesis | 5 | 0.001926225 |
| AMPK signaling pathway | 36 | 0.0630487 | Renal cell carcinoma | 1 | 0.626696439 | Fat digestion and absorption | 7 | 0.001999311 |
| Dorso-ventral axis formation | 10 | 0.088654972 | Hepatitis B | 2 | 0.630800806 | cGMP-PKG signaling pathway | 16 | 0.003142646 |
| Cytokine-cytokine receptor interaction | 70 | 0.104640807 | Thyroid hormone synthesis | 1 | 0.648095314 | Regulation of lipolysis in adipocytes | 8 | 0.00314709 |
| Platelet activation | 34 | 0.12103033 | Non-alcoholic fatty liver disease (NAFLD) | 2 | 0.649085564 | RIG-I-like receptor signaling pathway | 9 | 0.003696096 |
| Allograft rejection | 12 | 0.122946982 | mTOR signaling pathway | 2 | 0.656197422 | Tyrosine metabolism | 6 | 0.004074599 |
| Morphine addiction | 26 | 0.127786451 | Biosynthesis of amino acids | 1 | 0.668275201 | Huntington's disease | 17 | 0.005607705 |
| Renin secretion | 19 | 0.13133331 | Platinum drug resistance | 1 | 0.668275201 | Cytosolic DNA-sensing pathway | 8 | 0.006547329 |
| Cell adhesion molecules (CAMs) | 39 | 0.131915262 | Pertussis | 1 | 0.668275201 | Vascular smooth muscle contraction | 12 | 0.007091212 |
| Biotin metabolism | 2 | 0.134391601 | Complement and coagulation cascades | 1 | 0.687305101 | Renin secretion | 8 | 0.007199853 |
| Adrenergic signaling in cardiomyocytes | 40 | 0.14215072 | Peroxisome | 1 | 0.705250138 | Fc gamma R-mediated phagocytosis | 10 | 0.007622292 |
| Ovarian steroidogenesis | 15 | 0.156124462 | Salmonella infection | 1 | 0.718034077 | Hippo signaling pathway | 14 | 0.008262178 |
| Type I diabetes mellitus | 13 | 0.170704738 | Influenza A | 2 | 0.727029081 | Linoleic acid metabolism | 5 | 0.008387862 |
| cGMP-PKG signaling pathway | 44 | 0.17257672 | HTLV-I infection | 3 | 0.72944417 | Long-term potentiation | 8 | 0.008650627 |
| Nitrogen metabolism | 6 | 0.176942142 | Tuberculosis | 2 | 0.732818686 | Asthma | 5 | 0.009705683 |
| Tight junction | 37 | 0.178581163 | Endocytosis | 3 | 0.734258144 | Cholinergic synapse | 11 | 0.010380265 |
| Staphylococcus aureus infection | 16 | 0.178782653 | PI3K-Akt signaling pathway | 4 | 0.734270182 | Peroxisome | 9 | 0.011260251 |
| Glutathione metabolism | 15 | 0.198426136 | Transcriptional misregulation in cancer | 2 | 0.738504429 | Cell adhesion molecules (CAMs) | 13 | 0.01127452 |
| Arginine and proline metabolism | 14 | 0.220407304 | Longevity regulating pathway | 1 | 0.749494651 | Phospholipase D signaling pathway | 13 | 0.01127452 |
| D-Arginine and D-ornithine metabolism | 1 | 0.230050116 | Chemokine signaling pathway | 2 | 0.760231536 | Carbon metabolism | 11 | 0.011785503 |
| Taurine and hypotaurine metabolism | 4 | 0.233337043 | Amoebiasis | 1 | 0.767360056 | Axon guidance | 15 | 0.011943669 |
| Alzheimer's disease | 43 | 0.235851419 | Metabolic pathways | 15 | 0.813396549 | Starch and sucrose metabolism | 7 | 0.012786086 |
| Carbohydrate digestion and absorption | 13 | 0.245038911 | Sphingolipid signaling pathway | 1 | 0.829566615 | Thyroid hormone synthesis | 8 | 0.013225157 |
| Proximal tubule bicarbonate reclamation | 7 | 0.265192807 | Neurotrophin signaling pathway | 1 | 0.829566615 | Drug metabolism - other enzymes | 6 | 0.015600627 |
| Phototransduction | 8 | 0.268893777 | Platelet activation | 1 | 0.834548523 | Renin-angiotensin system | 4 | 0.017522333 |
| Intestinal immune network for IgA production | 13 | 0.272205901 | Lysosome | 1 | 0.836984955 | Vitamin digestion and absorption | 4 | 0.020306039 |
| Glycine, serine and threonine metabolism | 11 | 0.303753863 | Cell cycle | 1 | 0.839385743 | Arginine and proline metabolism | 6 | 0.020841803 |
| Cysteine and methionine metabolism | 12 | 0.332376121 | Oxidative phosphorylation | 1 | 0.859471877 | Retinol metabolism | 7 | 0.024904849 |
| Linoleic acid metabolism | 8 | 0.344658338 | MicroRNAs in cancer | 2 | 0.935071457 | Circadian entrainment | 9 | 0.025316321 |
| Homologous recombination | 8 | 0.344658338 | Viral carcinogenesis | 1 | 0.951944945 | Maturity onset diabetes of the young | 4 | 0.026649037 |
| Phenylalanine metabolism | 5 | 0.350121979 | Olfactory transduction | 1 | 0.998020116 | Hypertrophic cardiomyopathy (HCM) | 8 | 0.031019549 |
| Synaptic vesicle cycle | 16 | 0.372098724 | Citrate cycle (TCA cycle) | 0 | 1 | Dorso-ventral axis formation | 4 | 0.034053233 |
| Protein digestion and absorption | 22 | 0.412411637 | Pentose phosphate pathway | 0 | 1 | Staphylococcus aureus infection | 6 | 0.034637752 |
| Alanine, aspartate and glutamate metabolism | 9 | 0.414846735 | Pentose and glucuronate interconversions | 0 | 1 | Retrograde endocannabinoid signaling | 9 | 0.035732904 |
| Autoimmune thyroid disease | 13 | 0.418418622 | Ascorbate and aldarate metabolism | 0 | 1 | Phenylalanine metabolism | 3 | 0.037457339 |
| Galactose metabolism | 8 | 0.422756415 | Fatty acid biosynthesis | 0 | 1 | Metabolic pathways | 68 | 0.038456805 |
| Herpes simplex infection | 44 | 0.428324404 | Fatty acid elongation | 0 | 1 | 2-Oxocarboxylic acid metabolism | 3 | 0.043505727 |
| Renin-angiotensin system | 6 | 0.441129976 | Fatty acid degradation | 0 | 1 | Fatty acid degradation | 5 | 0.044663146 |
| RIG-I-like receptor signaling pathway | 17 | 0.445129322 | Synthesis and degradation of ketone bodies | 0 | 1 | Calcium signaling pathway | 13 | 0.056437785 |
| Alcoholism | 42 | 0.4708873 | Steroid biosynthesis | 0 | 1 | Regulation of actin cytoskeleton | 15 | 0.056823559 |
| Graft-versus-host disease | 10 | 0.476531216 | Ubiquinone and other terpenoid-quinone biosynthesis | 0 | 1 | Bacterial invasion of epithelial cells | 7 | 0.058179287 |
| Insulin secretion | 20 | 0.496388273 | Pyrimidine metabolism | 0 | 1 | Complement and coagulation cascades | 7 | 0.061513106 |
| Hedgehog signaling pathway | 11 | 0.498840207 | Alanine, aspartate and glutamate metabolism | 0 | 1 | Alanine, aspartate and glutamate metabolism | 4 | 0.068461712 |
| One carbon pool by folate | 5 | 0.501723188 | Glycine, serine and threonine metabolism | 0 | 1 | ECM-receptor interaction | 7 | 0.072203021 |
| Metabolism of xenobiotics by cytochrome P450 | 17 | 0.522725476 | Valine, leucine and isoleucine degradation | 0 | 1 | Mineral absorption | 5 | 0.080481896 |
| Chemical carcinogenesis | 19 | 0.528884955 | Valine, leucine and isoleucine biosynthesis | 0 | 1 | Taurine and hypotaurine metabolism | 2 | 0.083407686 |
| Retinol metabolism | 15 | 0.54287269 | Lysine biosynthesis | 0 | 1 | Tight junction | 10 | 0.091297193 |
| Phagosome | 35 | 0.547061498 | Lysine degradation | 0 | 1 | GABAergic synapse | 7 | 0.096677354 |
| Taste transduction | 19 | 0.552720738 | Histidine metabolism | 0 | 1 | alpha-Linolenic acid metabolism | 3 | 0.097819118 |
| Biosynthesis of amino acids | 17 | 0.572847613 | Tyrosine metabolism | 0 | 1 | Alcoholism | 12 | 0.102954044 |
| Bile secretion | 16 | 0.58375272 | Phenylalanine metabolism | 0 | 1 | Protein digestion and absorption | 7 | 0.105739642 |
| Vascular smooth muscle contraction | 27 | 0.58818973 | Phenylalanine, tyrosine and tryptophan biosynthesis | 0 | 1 | Fatty acid biosynthesis | 2 | 0.111644562 |
| Steroid hormone biosynthesis | 13 | 0.593650498 | beta-Alanine metabolism | 0 | 1 | Ascorbate and aldarate metabolism | 3 | 0.116712824 |
| Primary immunodeficiency | 8 | 0.608881524 | Taurine and hypotaurine metabolism | 0 | 1 | Adrenergic signaling in cardiomyocytes | 10 | 0.123993843 |
| Cardiac muscle contraction | 17 | 0.643902164 | Selenocompound metabolism | 0 | 1 | ABC transporters | 4 | 0.130907622 |
| Drug metabolism - cytochrome P450 | 15 | 0.644984879 | D-Glutamine and D-glutamate metabolism | 0 | 1 | Pancreatic secretion | 7 | 0.13553134 |
| Valine, leucine and isoleucine biosynthesis | 1 | 0.648621829 | D-Arginine and D-ornithine metabolism | 0 | 1 | Pentose phosphate pathway | 3 | 0.136840315 |
| Riboflavin metabolism | 1 | 0.648621829 | N-Glycan biosynthesis | 0 | 1 | Hedgehog signaling pathway | 4 | 0.147182266 |
| Vibrio cholerae infection | 11 | 0.649946143 | Mucin type O-Glycan biosynthesis | 0 | 1 | beta-Alanine metabolism | 3 | 0.158046926 |
| Fructose and mannose metabolism | 7 | 0.662986951 | Other types of O-glycan biosynthesis | 0 | 1 | Valine, leucine and isoleucine degradation | 4 | 0.164192388 |
| Histidine metabolism | 5 | 0.677170758 | Glycosaminoglycan degradation | 0 | 1 | Riboflavin metabolism | 1 | 0.166579651 |
| Vitamin digestion and absorption | 5 | 0.677170758 | Glycosaminoglycan biosynthesis - chondroitin sulfate / dermatan sulfate | 0 | 1 | Propanoate metabolism | 3 | 0.16900647 |
| Phosphatidylinositol signaling system | 21 | 0.683127541 | Glycosaminoglycan biosynthesis - keratan sulfate | 0 | 1 | Base excision repair | 3 | 0.180177915 |
| Pentose phosphate pathway | 6 | 0.686550934 | Glycosaminoglycan biosynthesis - heparan sulfate / heparin | 0 | 1 | Endocytosis | 15 | 0.183794634 |
| Nicotinate and nicotinamide metabolism | 6 | 0.686550934 | Glycerolipid metabolism | 0 | 1 | Other glycan degradation | 2 | 0.189934245 |
| Inositol phosphate metabolism | 15 | 0.691330667 | Inositol phosphate metabolism | 0 | 1 | Protein processing in endoplasmic reticulum | 10 | 0.200577775 |
| Notch signaling pathway | 10 | 0.693862071 | Glycosylphosphatidylinositol(GPI)-anchor biosynthesis | 0 | 1 | Butirosin and neomycin biosynthesis | 1 | 0.203702694 |
| Arachidonic acid metabolism | 13 | 0.696166044 | Glycosphingolipid biosynthesis - lacto and neolacto series | 0 | 1 | Phenylalanine, tyrosine and tryptophan biosynthesis | 1 | 0.203702694 |
| Glycosaminoglycan biosynthesis - chondroitin sulfate / dermatan sulfate | 4 | 0.70889025 | Glycosphingolipid biosynthesis - globo series | 0 | 1 | Dilated cardiomyopathy | 6 | 0.204626938 |
| Phenylalanine, tyrosine and tryptophan biosynthesis | 1 | 0.729487592 | Glycosphingolipid biosynthesis - ganglio series | 0 | 1 | Inositol phosphate metabolism | 5 | 0.208553116 |
| Pancreatic secretion | 20 | 0.731137982 | Glyoxylate and dicarboxylate metabolism | 0 | 1 | Pentose and glucuronate interconversions | 3 | 0.214776083 |
| Maturity onset diabetes of the young | 5 | 0.747583892 | Propanoate metabolism | 0 | 1 | Primary immunodeficiency | 3 | 0.214776083 |
| beta-Alanine metabolism | 6 | 0.750425546 | Butanoate metabolism | 0 | 1 | Steroid biosynthesis | 2 | 0.223000703 |
| Gastric acid secretion | 15 | 0.753786105 | One carbon pool by folate | 0 | 1 | Basal cell carcinoma | 4 | 0.228494642 |
| Pentose and glucuronate interconversions | 7 | 0.754163634 | Thiamine metabolism | 0 | 1 | Gastric acid secretion | 5 | 0.232993237 |
| Collecting duct acid secretion | 5 | 0.778220597 | Riboflavin metabolism | 0 | 1 | Glutamatergic synapse | 7 | 0.24501026 |
| Endocrine and other factor-regulated calcium reabsorption | 9 | 0.785738258 | Nicotinate and nicotinamide metabolism | 0 | 1 | Systemic lupus erythematosus | 8 | 0.247155469 |
| Lysine degradation | 10 | 0.789046746 | Pantothenate and CoA biosynthesis | 0 | 1 | Phosphatidylinositol signaling system | 6 | 0.270903393 |
| Vitamin B6 metabolism | 1 | 0.791749006 | Biotin metabolism | 0 | 1 | Biosynthesis of unsaturated fatty acids | 2 | 0.273237362 |
| PPAR signaling pathway | 14 | 0.803555534 | Lipoic acid metabolism | 0 | 1 | Mismatch repair | 2 | 0.273237362 |
| Glyoxylate and dicarboxylate metabolism | 5 | 0.805907502 | Folate biosynthesis | 0 | 1 | Porphyrin and chlorophyll metabolism | 3 | 0.287256149 |
| Butanoate metabolism | 5 | 0.805907502 | Porphyrin and chlorophyll metabolism | 0 | 1 | Histidine metabolism | 2 | 0.289996902 |
| Mismatch repair | 4 | 0.81017306 | Terpenoid backbone biosynthesis | 0 | 1 | Proteasome | 3 | 0.311911492 |
| Fanconi anemia pathway | 10 | 0.827698811 | Sulfur metabolism | 0 | 1 | Cysteine and methionine metabolism | 3 | 0.324265975 |
| Carbon metabolism | 22 | 0.844084863 | Aminoacyl-tRNA biosynthesis | 0 | 1 | Phototransduction | 2 | 0.339872154 |
| Circadian rhythm | 5 | 0.852977617 | Biosynthesis of unsaturated fatty acids | 0 | 1 | Glycolysis / Gluconeogenesis | 4 | 0.34883931 |
| alpha-Linolenic acid metabolism | 4 | 0.860175934 | Fatty acid metabolism | 0 | 1 | Endocrine and other factor-regulated calcium reabsorption | 3 | 0.348962989 |
| Drug metabolism - other enzymes | 8 | 0.862123851 | EGFR tyrosine kinase inhibitor resistance | 0 | 1 | Sphingolipid metabolism | 3 | 0.348962989 |
| Thyroid hormone synthesis | 13 | 0.862299243 | Endocrine resistance | 0 | 1 | Butanoate metabolism | 2 | 0.356275245 |
| Aldosterone synthesis and secretion | 15 | 0.864737874 | ABC transporters | 0 | 1 | Glyoxylate and dicarboxylate metabolism | 2 | 0.356275245 |
| Glycolysis / Gluconeogenesis | 12 | 0.874848562 | Ribosome biogenesis in eukaryotes | 0 | 1 | Salivary secretion | 5 | 0.363622501 |
| Antigen processing and presentation | 14 | 0.875906656 | Ribosome | 0 | 1 | Phagosome | 8 | 0.372430677 |
| Lysosome | 23 | 0.896477179 | RNA transport | 0 | 1 | Circadian rhythm | 2 | 0.388616202 |
| Glycerolipid metabolism | 10 | 0.900495723 | mRNA surveillance pathway | 0 | 1 | Citrate cycle (TCA cycle) | 2 | 0.388616202 |
| Base excision repair | 5 | 0.905461106 | RNA degradation | 0 | 1 | Biosynthesis of amino acids | 4 | 0.430283825 |
| ABC transporters | 7 | 0.908128885 | RNA polymerase | 0 | 1 | Fructose and mannose metabolism | 2 | 0.435704743 |
| Vasopressin-regulated water reabsorption | 7 | 0.908128885 | Basal transcription factors | 0 | 1 | Pathogenic Escherichia coli infection | 3 | 0.445942102 |
| Glucagon signaling pathway | 18 | 0.916827871 | DNA replication | 0 | 1 | Antigen processing and presentation | 4 | 0.450294553 |
| Synthesis and degradation of ketone bodies | 1 | 0.926875751 | Spliceosome | 0 | 1 | Folate biosynthesis | 1 | 0.471691416 |
| Sulfur metabolism | 1 | 0.926875751 | Proteasome | 0 | 1 | Glycerolipid metabolism | 3 | 0.492318883 |
| Selenocompound metabolism | 2 | 0.92875666 | Protein export | 0 | 1 | Glycosaminoglycan biosynthesis - keratan sulfate | 1 | 0.495246576 |
| Oocyte meiosis | 22 | 0.931925985 | Base excision repair | 0 | 1 | Glycosphingolipid biosynthesis - ganglio series | 1 | 0.495246576 |
| Fat digestion and absorption | 6 | 0.934819983 | Nucleotide excision repair | 0 | 1 | Glycine, serine and threonine metabolism | 2 | 0.537272565 |
| Mineral absorption | 8 | 0.93560823 | Mismatch repair | 0 | 1 | Pyruvate metabolism | 2 | 0.537272565 |
| Glycosaminoglycan biosynthesis - heparan sulfate / heparin | 3 | 0.938393399 | Homologous recombination | 0 | 1 | Regulation of autophagy | 2 | 0.537272565 |
| Citrate cycle (TCA cycle) | 4 | 0.938600436 | Non-homologous end-joining | 0 | 1 | Selenocompound metabolism | 1 | 0.539259257 |
| Salivary secretion | 15 | 0.93897579 | Fanconi anemia pathway | 0 | 1 | Glycosaminoglycan degradation | 1 | 0.579441771 |
| 2-Oxocarboxylic acid metabolism | 2 | 0.942471511 | ErbB signaling pathway | 0 | 1 | Morphine addiction | 4 | 0.582096377 |
| Ubiquinone and other terpenoid-quinone biosynthesis | 1 | 0.943714221 | Cytokine-cytokine receptor interaction | 0 | 1 | Vasopressin-regulated water reabsorption | 2 | 0.589403917 |
| Valine, leucine and isoleucine degradation | 7 | 0.947371648 | Phosphatidylinositol signaling system | 0 | 1 | Ether lipid metabolism | 2 | 0.601735914 |
| Amino sugar and nucleotide sugar metabolism | 7 | 0.947371648 | Phospholipase D signaling pathway | 0 | 1 | Parkinson's disease | 6 | 0.610590032 |
| Other types of O-glycan biosynthesis | 4 | 0.948363586 | Oocyte meiosis | 0 | 1 | Terpenoid backbone biosynthesis | 1 | 0.633253711 |
| Propanoate metabolism | 4 | 0.956682859 | p53 signaling pathway | 0 | 1 | Amino sugar and nucleotide sugar metabolism | 2 | 0.637046786 |
| Fatty acid degradation | 6 | 0.958699485 | Ubiquitin mediated proteolysis | 0 | 1 | Arrhythmogenic right ventricular cardiomyopathy (ARVC) | 3 | 0.646406789 |
| Steroid biosynthesis | 2 | 0.962731282 | Sulfur relay system | 0 | 1 | Proximal tubule bicarbonate reclamation | 1 | 0.649618091 |
| Ether lipid metabolism | 6 | 0.964686662 | SNARE interactions in vesicular transport | 0 | 1 | Glycosaminoglycan biosynthesis - heparan sulfate / heparin | 1 | 0.665253794 |
| Fatty acid biosynthesis | 1 | 0.966654626 | Regulation of autophagy | 0 | 1 | Fatty acid elongation | 1 | 0.680193202 |
| Non-homologous end-joining | 1 | 0.966654626 | Protein processing in endoplasmic reticulum | 0 | 1 | Glycosphingolipid biosynthesis - lacto and neolacto series | 1 | 0.694467257 |
| Cytosolic DNA-sensing pathway | 9 | 0.969801806 | Phagosome | 0 | 1 | Neuroactive ligand-receptor interaction | 11 | 0.695136108 |
| Parkinson's disease | 24 | 0.970653849 | Apoptosis - multiple species | 0 | 1 | Homologous recombination | 1 | 0.733586517 |
| Folate biosynthesis | 1 | 0.974335356 | Wnt signaling pathway | 0 | 1 | Aminoacyl-tRNA biosynthesis | 2 | 0.799274116 |
| Nucleotide excision repair | 6 | 0.974344802 | Dorso-ventral axis formation | 0 | 1 | Glycerophospholipid metabolism | 3 | 0.801584092 |
| Terpenoid backbone biosynthesis | 2 | 0.976036103 | Hedgehog signaling pathway | 0 | 1 | Lysosome | 4 | 0.803742871 |
| Glycosaminoglycan biosynthesis - keratan sulfate | 1 | 0.980247487 | TGF-beta signaling pathway | 0 | 1 | DNA replication | 1 | 0.806513549 |
| Primary bile acid biosynthesis | 1 | 0.988300726 | Axon guidance | 0 | 1 | Ubiquitin mediated proteolysis | 4 | 0.865148264 |
| Regulation of autophagy | 4 | 0.990156807 | Hippo signaling pathway | 0 | 1 | Nucleotide excision repair | 1 | 0.883002723 |
| Glycosphingolipid biosynthesis - lacto and neolacto series | 2 | 0.990270738 | Hippo signaling pathway -multiple species | 0 | 1 | Notch signaling pathway | 1 | 0.888235828 |
| Ascorbate and aldarate metabolism | 2 | 0.992258555 | Focal adhesion | 0 | 1 | Vibrio cholerae infection | 1 | 0.902574792 |
| Glycosaminoglycan degradation | 1 | 0.993071403 | ECM-receptor interaction | 0 | 1 | Lysine degradation | 1 | 0.906934157 |
| N-Glycan biosynthesis | 5 | 0.993803275 | Cell adhesion molecules (CAMs) | 0 | 1 | Fanconi anemia pathway | 1 | 0.915077583 |
| Hippo signaling pathway -multiple species | 2 | 0.993847101 | Adherens junction | 0 | 1 | Ribosome | 3 | 0.945446188 |
| Endocytosis | 44 | 0.993934802 | Tight junction | 0 | 1 | Pyrimidine metabolism | 2 | 0.949544321 |
| Huntington's disease | 30 | 0.996263931 | Signaling pathways regulating pluripotency of stem cells | 0 | 1 | Purine metabolism | 4 | 0.954602795 |
| Peroxisome | 10 | 0.996343512 | Antigen processing and presentation | 0 | 1 | RNA degradation | 1 | 0.970411482 |
| Ubiquitin mediated proteolysis | 19 | 0.997446309 | Jak-STAT signaling pathway | 0 | 1 | Ribosome biogenesis in eukaryotes | 1 | 0.977535375 |
| Pyruvate metabolism | 3 | 0.997659516 | Hematopoietic cell lineage | 0 | 1 | Taste transduction | 1 | 0.977535375 |
| Purine metabolism | 25 | 0.998461039 | Natural killer cell mediated cytotoxicity | 0 | 1 | mRNA surveillance pathway | 1 | 0.984444305 |
| SNARE interactions in vesicular transport | 2 | 0.998479657 | Fc epsilon RI signaling pathway | 0 | 1 | RNA transport | 2 | 0.996466706 |
| Porphyrin and chlorophyll metabolism | 3 | 0.998483778 | Fc gamma R-mediated phagocytosis | 0 | 1 | Oxidative phosphorylation | 1 | 0.99775173 |
| Fatty acid elongation | 1 | 0.998561854 | Leukocyte transendothelial migration | 0 | 1 | Spliceosome | 1 | 0.997853136 |
| Glycosylphosphatidylinositol(GPI)-anchor biosynthesis | 1 | 0.998561854 | Intestinal immune network for IgA production | 0 | 1 | Olfactory transduction | 1 | 0.999999996 |
| Starch and sucrose metabolism | 5 | 0.99865076 | Circadian rhythm | 0 | 1 | Basal transcription factors | 0 | 1 |
| Pyrimidine metabolism | 12 | 0.999108667 | Synaptic vesicle cycle | 0 | 1 | Biotin metabolism | 0 | 1 |
| Basal transcription factors | 3 | 0.999215263 | Long-term depression | 0 | 1 | Cardiac muscle contraction | 0 | 1 |
| Glycerophospholipid metabolism | 10 | 0.999481841 | Phototransduction | 0 | 1 | Collecting duct acid secretion | 0 | 1 |
| RNA degradation | 7 | 0.999601399 | Progesterone-mediated oocyte maturation | 0 | 1 | D-Arginine and D-ornithine metabolism | 0 | 1 |
| RNA polymerase | 1 | 0.9997018 | Estrogen signaling pathway | 0 | 1 | D-Glutamine and D-glutamate metabolism | 0 | 1 |
| Protein processing in endoplasmic reticulum | 21 | 0.999732176 | Melanogenesis | 0 | 1 | Glycosaminoglycan biosynthesis - chondroitin sulfate / dermatan sulfate | 0 | 1 |
| Systemic lupus erythematosus | 15 | 0.99986157 | AGE-RAGE signaling pathway in diabetic complications | 0 | 1 | Glycosphingolipid biosynthesis - globo series | 0 | 1 |
| DNA replication | 1 | 0.999919694 | Type I diabetes mellitus | 0 | 1 | Glycosylphosphatidylinositol(GPI)-anchor biosynthesis | 0 | 1 |
| Fatty acid metabolism | 2 | 0.999946996 | Vasopressin-regulated water reabsorption | 0 | 1 | Hippo signaling pathway -multiple species | 0 | 1 |
| Proteasome | 1 | 0.999990172 | Vitamin digestion and absorption | 0 | 1 | Lipoic acid metabolism | 0 | 1 |
| Sphingolipid metabolism | 1 | 0.999995531 | Prion diseases | 0 | 1 | Lysine biosynthesis | 0 | 1 |
| Ribosome biogenesis in eukaryotes | 4 | 0.999999023 | Bacterial invasion of epithelial cells | 0 | 1 | Mucin type O-Glycan biosynthesis | 0 | 1 |
| Aminoacyl-tRNA biosynthesis | 2 | 0.99999937 | Pathogenic Escherichia coli infection | 0 | 1 | N-Glycan biosynthesis | 0 | 1 |
| Spliceosome | 7 | 0.999999997 | Legionellosis | 0 | 1 | Nicotinate and nicotinamide metabolism | 0 | 1 |
| mRNA surveillance pathway | 2 | 0.999999999 | Staphylococcus aureus infection | 0 | 1 | Nicotine addiction | 0 | 1 |
| Oxidative phosphorylation | 6 | 0.999999999 | Proteoglycans in cancer | 0 | 1 | Nitrogen metabolism | 0 | 1 |
| Olfactory transduction | 10 | 1 | Colorectal cancer | 0 | 1 | Non-homologous end-joining | 0 | 1 |
| Lysine biosynthesis | 0 | 1 | Endometrial cancer | 0 | 1 | One carbon pool by folate | 0 | 1 |
| Other glycan degradation | 0 | 1 | Glioma | 0 | 1 | Other types of O-glycan biosynthesis | 0 | 1 |
| Mucin type O-Glycan biosynthesis | 0 | 1 | Basal cell carcinoma | 0 | 1 | Pantothenate and CoA biosynthesis | 0 | 1 |
| Butirosin and neomycin biosynthesis | 0 | 1 | Melanoma | 0 | 1 | Primary bile acid biosynthesis | 0 | 1 |
| Glycosphingolipid biosynthesis - globo series | 0 | 1 | Bladder cancer | 0 | 1 | Protein export | 0 | 1 |
| Glycosphingolipid biosynthesis - ganglio series | 0 | 1 | Non-small cell lung cancer | 0 | 1 | RNA polymerase | 0 | 1 |
| Thiamine metabolism | 0 | 1 | Choline metabolism in cancer | 0 | 1 | SNARE interactions in vesicular transport | 0 | 1 |
| Pantothenate and CoA biosynthesis | 0 | 1 | Asthma | 0 | 1 | Sulfur metabolism | 0 | 1 |
| Lipoic acid metabolism | 0 | 1 | Autoimmune thyroid disease | 0 | 1 | Sulfur relay system | 0 | 1 |
| Biosynthesis of unsaturated fatty acids | 0 | 1 | Inflammatory bowel disease (IBD) | 0 | 1 | Synaptic vesicle cycle | 0 | 1 |
| Metabolic pathways | 182 | 1 | Rheumatoid arthritis | 0 | 1 | Synthesis and degradation of ketone bodies | 0 | 1 |
| Ribosome | 3 | 1 | Allograft rejection | 0 | 1 | Thiamine metabolism | 0 | 1 |
| RNA transport | 7 | 1 | Graft-versus-host disease | 0 | 1 | Ubiquinone and other terpenoid-quinone biosynthesis | 0 | 1 |
| Protein export | 0 | 1 | Primary immunodeficiency | 0 | 1 | Valine, leucine and isoleucine biosynthesis | 0 | 1 |
| Sulfur relay system | 0 | 1 | Viral myocarditis | 0 | 1 | Vitamin B6 metabolism | 0 | 1 |

Pathways in M0

| **symptom** | **overlap** | **p_value** | **drug** | **overlap** | **p_value** | **herb** | **overlap** | **p_value** |
| --- | --- | --- | --- | --- | --- | --- | --- | --- |
| Alzheimer's disease | 75 | 6.06054E-15 | Nicotine addiction | 19 | 1.58197E-41 | Pathways in cancer | 75 | 8.00666E-39 |
| Neuroactive ligand-receptor interaction | 104 | 5.79069E-14 | Retrograde endocannabinoid signaling | 20 | 7.41119E-35 | Hepatitis B | 46 | 2.93087E-34 |
| AGE-RAGE signaling pathway in diabetic complications | 52 | 8.24591E-14 | GABAergic synapse | 19 | 1.02727E-33 | PI3K-Akt signaling pathway | 59 | 2.87396E-28 |
| Calcium signaling pathway | 74 | 1.11127E-12 | Morphine addiction | 19 | 2.08674E-33 | AGE-RAGE signaling pathway in diabetic complications | 35 | 7.78729E-28 |
| Amyotrophic lateral sclerosis (ALS) | 32 | 4.32118E-12 | Neuroactive ligand-receptor interaction | 23 | 1.06117E-31 | Prostate cancer | 30 | 1.40761E-23 |
| Pathways in cancer | 130 | 5.33864E-12 | Taste transduction | 6 | 8.94556E-08 | Apoptosis | 35 | 1.90664E-22 |
| Pancreatic cancer | 37 | 1.16104E-11 | Serotonergic synapse | 5 | 1.29383E-05 | FoxO signaling pathway | 34 | 3.58406E-22 |
| Bladder cancer | 27 | 3.9968E-11 | Arachidonic acid metabolism | 3 | 0.000664558 | Endocrine resistance | 29 | 3.75199E-21 |
| Proteoglycans in cancer | 77 | 1.10564E-10 | Regulation of lipolysis in adipocytes | 2 | 0.010580131 | TNF signaling pathway | 30 | 1.46034E-20 |
| Colorectal cancer | 34 | 1.79644E-10 | Leishmaniasis | 2 | 0.01708342 | HIF-1 signaling pathway | 29 | 2.44852E-20 |
| Hepatitis B | 60 | 2.05891E-10 | Vascular smooth muscle contraction | 2 | 0.043956611 | Pancreatic cancer | 24 | 5.61501E-20 |
| EGFR tyrosine kinase inhibitor resistance | 40 | 3.19789E-10 | Terpenoid backbone biosynthesis | 1 | 0.059775517 | EGFR tyrosine kinase inhibitor resistance | 26 | 5.89869E-20 |
| Long-term potentiation | 35 | 3.34872E-10 | Renin-angiotensin system | 1 | 0.062408944 | Focal adhesion | 38 | 1.52294E-19 |
| cAMP signaling pathway | 74 | 4.36397E-10 | alpha-Linolenic acid metabolism | 1 | 0.067654452 | Proteoglycans in cancer | 38 | 2.17419E-19 |
| HIF-1 signaling pathway | 46 | 1.06818E-09 | Linoleic acid metabolism | 1 | 0.078060593 | Platinum drug resistance | 24 | 1.7988E-18 |
| Nicotine addiction | 25 | 1.11123E-09 | Calcium signaling pathway | 2 | 0.088733633 | Toxoplasmosis | 27 | 1.73616E-16 |
| Endocrine resistance | 44 | 1.34236E-09 | Fat digestion and absorption | 1 | 0.108610569 | Glioma | 21 | 2.0912E-16 |
| Glioma | 33 | 4.6267E-09 | Ether lipid metabolism | 1 | 0.118574929 | Tuberculosis | 32 | 3.81092E-16 |
| FoxO signaling pathway | 53 | 8.45491E-09 | Malaria | 1 | 0.128431702 | Transcriptional misregulation in cancer | 32 | 5.3503E-16 |
| Malaria | 27 | 1.1853E-08 | Ovarian steroidogenesis | 1 | 0.130879216 | Chagas disease (American trypanosomiasis) | 25 | 6.81442E-16 |
| Amphetamine addiction | 33 | 1.23303E-08 | Legionellosis | 1 | 0.143017584 | HTLV-I infection | 38 | 6.92755E-16 |
| Renal cell carcinoma | 33 | 1.23303E-08 | Staphylococcus aureus infection | 1 | 0.143017584 | Small cell lung cancer | 23 | 8.09562E-16 |
| Estrogen signaling pathway | 42 | 3.55515E-08 | Viral myocarditis | 1 | 0.150221863 | Colorectal cancer | 20 | 1.14547E-15 |
| Prostate cancer | 39 | 3.74745E-08 | VEGF signaling pathway | 1 | 0.15736765 | Bladder cancer | 17 | 1.2283E-15 |
| Thyroid cancer | 19 | 3.76083E-08 | Renin secretion | 1 | 0.164455403 | Rap1 signaling pathway | 33 | 1.07485E-14 |
| Oxytocin signaling pathway | 58 | 4.40123E-08 | Bile secretion | 1 | 0.180770353 | p53 signaling pathway | 20 | 1.18022E-14 |
| Hypertrophic cardiomyopathy (HCM) | 37 | 4.81705E-08 | Pertussis | 1 | 0.189954629 | Influenza A | 30 | 1.49146E-14 |
| Endometrial cancer | 27 | 6.35286E-08 | Complement and coagulation cascades | 1 | 0.199039453 | Insulin resistance | 24 | 2.20948E-14 |
| Serotonergic synapse | 45 | 8.357E-08 | Aldosterone synthesis and secretion | 1 | 0.203544896 | Leishmaniasis | 20 | 2.92581E-14 |
| Dopaminergic synapse | 49 | 1.80674E-07 | Chemical carcinogenesis | 1 | 0.205788431 | MAPK signaling pathway | 35 | 9.22414E-14 |
| Focal adhesion | 68 | 2.61085E-07 | Small cell lung cancer | 1 | 0.214701708 | Melanoma | 19 | 2.7668E-13 |
| Circadian entrainment | 39 | 3.09494E-07 | Rheumatoid arthritis | 1 | 0.221323204 | Non-small cell lung cancer | 17 | 5.19458E-13 |
| Chronic myeloid leukemia | 32 | 6.13417E-07 | NF-kappa B signaling pathway | 1 | 0.227890778 | Ras signaling pathway | 32 | 6.09885E-13 |
| Long-term depression | 28 | 6.47661E-07 | Glycerophospholipid metabolism | 1 | 0.234404853 | Malaria | 16 | 7.10035E-13 |
| Neurotrophin signaling pathway | 45 | 8.65348E-07 | Pancreatic secretion | 1 | 0.236564396 | Pertussis | 19 | 8.17595E-13 |
| Retrograde endocannabinoid signaling | 39 | 1.97116E-06 | Inflammatory mediator regulation of TRP channels | 1 | 0.240865851 | Ovarian steroidogenesis | 16 | 1.01506E-12 |
| Central carbon metabolism in cancer | 29 | 2.8044E-06 | Amoebiasis | 1 | 0.243007794 | NF-kappa B signaling pathway | 20 | 4.44502E-12 |
| cGMP-PKG signaling pathway | 56 | 2.81468E-06 | AGE-RAGE signaling pathway in diabetic complications | 1 | 0.247274188 | Chronic myeloid leukemia | 18 | 5.47434E-12 |
| Apoptosis | 49 | 2.98166E-06 | TNF signaling pathway | 1 | 0.266187359 | T cell receptor signaling pathway | 21 | 7.09445E-12 |
| Prolactin signaling pathway | 30 | 4.90163E-06 | Leukocyte transendothelial migration | 1 | 0.282613205 | Toll-like receptor signaling pathway | 21 | 8.60036E-12 |
| Influenza A | 57 | 6.3734E-06 | Platelet activation | 1 | 0.290692432 | MicroRNAs in cancer | 35 | 1.03294E-11 |
| Inflammatory mediator regulation of TRP channels | 37 | 6.63698E-06 | AMPK signaling pathway | 1 | 0.292698471 | African trypanosomiasis | 13 | 1.04479E-11 |
| Non-small cell lung cancer | 25 | 6.92027E-06 | Pathways in cancer | 2 | 0.305261547 | Osteoclast differentiation | 23 | 1.18819E-11 |
| GnRH signaling pathway | 35 | 7.23859E-06 | Natural killer cell mediated cytotoxicity | 1 | 0.314406483 | Jak-STAT signaling pathway | 25 | 1.60793E-11 |
| PI3K-Akt signaling pathway | 96 | 7.64571E-06 | Phospholipase D signaling pathway | 1 | 0.331687769 | Amyotrophic lateral sclerosis (ALS) | 15 | 2.09196E-11 |
| ErbB signaling pathway | 34 | 8.67838E-06 | Cell adhesion molecules (CAMs) | 1 | 0.331687769 | Measles | 23 | 2.26085E-11 |
| Thyroid hormone signaling pathway | 42 | 9.25483E-06 | Adrenergic signaling in cardiomyocytes | 1 | 0.341105956 | VEGF signaling pathway | 16 | 3.06354E-11 |
| Tuberculosis | 57 | 9.38356E-06 | Phagosome | 1 | 0.350395932 | Non-alcoholic fatty liver disease (NAFLD) | 24 | 3.76984E-11 |
| Renin secretion | 27 | 1.09721E-05 | Hippo signaling pathway | 1 | 0.350395932 | Prolactin signaling pathway | 17 | 4.57095E-11 |
| Melanoma | 29 | 1.1198E-05 | Oxytocin signaling pathway | 1 | 0.357736732 | Thyroid hormone signaling pathway | 21 | 7.32937E-11 |
| Rap1 signaling pathway | 65 | 1.18302E-05 | cGMP-PKG signaling pathway | 1 | 0.37574003 | Epstein-Barr virus infection | 27 | 1.06317E-10 |
| Chagas disease (American trypanosomiasis) | 38 | 1.22019E-05 | Tuberculosis | 1 | 0.393255606 | Apoptosis - multiple species | 12 | 1.33369E-10 |
| VEGF signaling pathway | 26 | 1.274E-05 | Rap1 signaling pathway | 1 | 0.449329537 | Estrogen signaling pathway | 19 | 1.54898E-10 |
| Viral myocarditis | 25 | 1.47229E-05 | Regulation of actin cytoskeleton | 1 | 0.455587705 | ErbB signaling pathway | 18 | 1.57483E-10 |
| Adrenergic signaling in cardiomyocytes | 49 | 1.70699E-05 | Metabolic pathways | 4 | 0.46241821 | Rheumatoid arthritis | 18 | 1.919E-10 |
| MicroRNAs in cancer | 85 | 1.90885E-05 | Ras signaling pathway | 1 | 0.475456075 | Endometrial cancer | 14 | 3.68459E-10 |
| Cocaine addiction | 22 | 2.18999E-05 | HTLV-I infection | 1 | 0.518670449 | Neurotrophin signaling pathway | 20 | 7.10036E-10 |
| Glutamatergic synapse | 40 | 2.20488E-05 | MicroRNAs in cancer | 1 | 0.572208245 | Hepatitis C | 21 | 7.41446E-10 |
| Dilated cardiomyopathy | 33 | 3.17316E-05 | Glycolysis / Gluconeogenesis | 0 | 1 | Cytokine-cytokine receptor interaction | 30 | 7.69886E-10 |
| Vascular smooth muscle contraction | 41 | 3.57924E-05 | Citrate cycle (TCA cycle) | 0 | 1 | Viral carcinogenesis | 26 | 9.55422E-10 |
| Hematopoietic cell lineage | 32 | 4.99858E-05 | Pentose phosphate pathway | 0 | 1 | Choline metabolism in cancer | 18 | 1.68188E-09 |
| Insulin signaling pathway | 45 | 6.8624E-05 | Pentose and glucuronate interconversions | 0 | 1 | Viral myocarditis | 14 | 1.78411E-09 |
| Adherens junction | 28 | 8.16274E-05 | Fructose and mannose metabolism | 0 | 1 | Herpes simplex infection | 24 | 2.73365E-09 |
| Pertussis | 28 | 0.000107373 | Galactose metabolism | 0 | 1 | Adipocytokine signaling pathway | 15 | 2.76864E-09 |
| Amoebiasis | 34 | 0.000143869 | Ascorbate and aldarate metabolism | 0 | 1 | Allograft rejection | 11 | 9.30925E-09 |
| Gap junction | 31 | 0.000164673 | Fatty acid biosynthesis | 0 | 1 | Amphetamine addiction | 14 | 1.336E-08 |
| MAPK signaling pathway | 71 | 0.000181974 | Fatty acid elongation | 0 | 1 | NOD-like receptor signaling pathway | 13 | 1.444E-08 |
| Fc epsilon RI signaling pathway | 25 | 0.000249461 | Fatty acid degradation | 0 | 1 | Aldosterone synthesis and secretion | 15 | 2.29157E-08 |
| Ras signaling pathway | 64 | 0.000290641 | Synthesis and degradation of ketone bodies | 0 | 1 | Longevity regulating pathway | 16 | 2.7289E-08 |
| Longevity regulating pathway - multiple species | 24 | 0.00030205 | Steroid biosynthesis | 0 | 1 | Sphingolipid signaling pathway | 18 | 2.86145E-08 |
| Leishmaniasis | 26 | 0.000346157 | Primary bile acid biosynthesis | 0 | 1 | AMPK signaling pathway | 18 | 4.24347E-08 |
| Inflammatory bowel disease (IBD) | 24 | 0.000395338 | Ubiquinone and other terpenoid-quinone biosynthesis | 0 | 1 | Cell cycle | 18 | 4.82562E-08 |
| T cell receptor signaling pathway | 34 | 0.000505061 | Steroid hormone biosynthesis | 0 | 1 | Inflammatory mediator regulation of TRP channels | 16 | 5.03402E-08 |
| African trypanosomiasis | 15 | 0.000559375 | Oxidative phosphorylation | 0 | 1 | Adherens junction | 14 | 5.11203E-08 |
| Toxoplasmosis | 37 | 0.000588156 | Arginine biosynthesis | 0 | 1 | Salmonella infection | 15 | 5.32362E-08 |
| Cholinergic synapse | 35 | 0.000729444 | Purine metabolism | 0 | 1 | Amoebiasis | 16 | 5.83729E-08 |
| Acute myeloid leukemia | 21 | 0.000924728 | Caffeine metabolism | 0 | 1 | Inflammatory bowel disease (IBD) | 13 | 7.7368E-08 |
| Non-alcoholic fatty liver disease (NAFLD) | 44 | 0.001073149 | Pyrimidine metabolism | 0 | 1 | Oxytocin signaling pathway | 20 | 8.13112E-08 |
| TNF signaling pathway | 34 | 0.001271794 | Alanine, aspartate and glutamate metabolism | 0 | 1 | Legionellosis | 12 | 8.90517E-08 |
| Osteoclast differentiation | 39 | 0.001294982 | Glycine, serine and threonine metabolism | 0 | 1 | Central carbon metabolism in cancer | 13 | 1.13015E-07 |
| Arrhythmogenic right ventricular cardiomyopathy (ARVC) | 25 | 0.001366107 | Cysteine and methionine metabolism | 0 | 1 | GnRH signaling pathway | 15 | 1.16389E-07 |
| Salmonella infection | 28 | 0.0013867 | Valine, leucine and isoleucine degradation | 0 | 1 | Steroid hormone biosynthesis | 12 | 1.66444E-07 |
| Type II diabetes mellitus | 18 | 0.001663952 | Valine, leucine and isoleucine biosynthesis | 0 | 1 | Type II diabetes mellitus | 11 | 1.81056E-07 |
| Sphingolipid signaling pathway | 36 | 0.001681075 | Lysine biosynthesis | 0 | 1 | B cell receptor signaling pathway | 13 | 2.7451E-07 |
| Melanogenesis | 31 | 0.001934949 | Lysine degradation | 0 | 1 | Insulin signaling pathway | 18 | 2.85457E-07 |
| Toll-like receptor signaling pathway | 32 | 0.00264836 | Arginine and proline metabolism | 0 | 1 | Graft-versus-host disease | 10 | 3.53439E-07 |
| Prion diseases | 14 | 0.002656359 | Histidine metabolism | 0 | 1 | Type I diabetes mellitus | 10 | 5.71897E-07 |
| Dorso-ventral axis formation | 12 | 0.002676145 | Tyrosine metabolism | 0 | 1 | Natural killer cell mediated cytotoxicity | 17 | 8.2463E-07 |
| Gastric acid secretion | 24 | 0.003126308 | Phenylalanine metabolism | 0 | 1 | Fc epsilon RI signaling pathway | 12 | 8.70328E-07 |
| Hepatitis C | 38 | 0.003308733 | Tryptophan metabolism | 0 | 1 | cAMP signaling pathway | 21 | 9.60618E-07 |
| Platinum drug resistance | 24 | 0.003800984 | Phenylalanine, tyrosine and tryptophan biosynthesis | 0 | 1 | Platelet activation | 16 | 1.11098E-06 |
| GABAergic synapse | 27 | 0.004296138 | beta-Alanine metabolism | 0 | 1 | Acute myeloid leukemia | 11 | 1.15087E-06 |
| Measles | 38 | 0.004367267 | Taurine and hypotaurine metabolism | 0 | 1 | Arachidonic acid metabolism | 11 | 2.75886E-06 |
| Apoptosis - multiple species | 13 | 0.004370634 | Selenocompound metabolism | 0 | 1 | Leukocyte transendothelial migration | 15 | 3.58967E-06 |
| Renin-angiotensin system | 10 | 0.005309842 | D-Glutamine and D-glutamate metabolism | 0 | 1 | Autoimmune thyroid disease | 10 | 3.67888E-06 |
| p53 signaling pathway | 22 | 0.005722684 | D-Arginine and D-ornithine metabolism | 0 | 1 | Renal cell carcinoma | 11 | 6.07061E-06 |
| Signaling pathways regulating pluripotency of stem cells | 39 | 0.006157069 | Glutathione metabolism | 0 | 1 | Epithelial cell signaling in Helicobacter pylori infection | 11 | 7.0438E-06 |
| Cytokine-cytokine receptor interaction | 66 | 0.00633617 | Starch and sucrose metabolism | 0 | 1 | Serotonergic synapse | 14 | 9.2599E-06 |
| Choline metabolism in cancer | 29 | 0.008816955 | N-Glycan biosynthesis | 0 | 1 | Carbohydrate digestion and absorption | 9 | 9.79257E-06 |
| B cell receptor signaling pathway | 22 | 0.009882949 | Other glycan degradation | 0 | 1 | Dopaminergic synapse | 15 | 1.08919E-05 |
| Phospholipase D signaling pathway | 38 | 0.011905104 | Mucin type O-Glycan biosynthesis | 0 | 1 | Intestinal immune network for IgA production | 9 | 1.17844E-05 |
| Cell adhesion molecules (CAMs) | 38 | 0.011905104 | Other types of O-glycan biosynthesis | 0 | 1 | Gap junction | 12 | 1.65904E-05 |
| Insulin resistance | 30 | 0.014534263 | Amino sugar and nucleotide sugar metabolism | 0 | 1 | Cocaine addiction | 9 | 1.68198E-05 |
| Taste transduction | 24 | 0.014846144 | Butirosin and neomycin biosynthesis | 0 | 1 | Vascular smooth muscle contraction | 14 | 2.06155E-05 |
| Salivary secretion | 25 | 0.01888074 | Glycosaminoglycan degradation | 0 | 1 | Aldosterone-regulated sodium reabsorption | 8 | 2.14101E-05 |
| Rheumatoid arthritis | 25 | 0.01888074 | Glycosaminoglycan biosynthesis - chondroitin sulfate / dermatan sulfate | 0 | 1 | Longevity regulating pathway - multiple species | 10 | 2.52927E-05 |
| Legionellosis | 17 | 0.019401649 | Glycosaminoglycan biosynthesis - keratan sulfate | 0 | 1 | Galactose metabolism | 7 | 3.66466E-05 |
| Longevity regulating pathway | 26 | 0.020521656 | Glycosaminoglycan biosynthesis - heparan sulfate / heparin | 0 | 1 | Calcium signaling pathway | 17 | 4.18871E-05 |
| Type I diabetes mellitus | 14 | 0.020588538 | Glycerolipid metabolism | 0 | 1 | Progesterone-mediated oocyte maturation | 12 | 4.96761E-05 |
| Protein digestion and absorption | 25 | 0.021646377 | Inositol phosphate metabolism | 0 | 1 | RIG-I-like receptor signaling pathway | 10 | 5.61484E-05 |
| Small cell lung cancer | 24 | 0.022821742 | Glycosylphosphatidylinositol(GPI)-anchor biosynthesis | 0 | 1 | Glucagon signaling pathway | 12 | 6.70623E-05 |
| Autoimmune thyroid disease | 16 | 0.023918753 | Sphingolipid metabolism | 0 | 1 | Alzheimer's disease | 16 | 6.8008E-05 |
| Morphine addiction | 25 | 0.024717406 | Glycosphingolipid biosynthesis - lacto and neolacto series | 0 | 1 | Chemokine signaling pathway | 17 | 7.27835E-05 |
| NOD-like receptor signaling pathway | 17 | 0.027434875 | Glycosphingolipid biosynthesis - globo series | 0 | 1 | mTOR signaling pathway | 15 | 8.23213E-05 |
| Adipocytokine signaling pathway | 20 | 0.028006664 | Glycosphingolipid biosynthesis - ganglio series | 0 | 1 | Prion diseases | 7 | 8.42006E-05 |
| Glucagon signaling pathway | 27 | 0.028345439 | Pyruvate metabolism | 0 | 1 | Huntington's disease | 17 | 0.000107709 |
| TGF-beta signaling pathway | 23 | 0.031482186 | Glyoxylate and dicarboxylate metabolism | 0 | 1 | Fc gamma R-mediated phagocytosis | 11 | 0.000127014 |
| Allograft rejection | 12 | 0.031773334 | Propanoate metabolism | 0 | 1 | Wnt signaling pathway | 14 | 0.000133365 |
| Aldosterone synthesis and secretion | 22 | 0.037934955 | Butanoate metabolism | 0 | 1 | Phospholipase D signaling pathway | 14 | 0.000143762 |
| Taurine and hypotaurine metabolism | 5 | 0.038320076 | One carbon pool by folate | 0 | 1 | Cholinergic synapse | 12 | 0.000168046 |
| Citrate cycle (TCA cycle) | 10 | 0.040015574 | Thiamine metabolism | 0 | 1 | Shigellosis | 9 | 0.000169001 |
| Circadian rhythm | 10 | 0.040015574 | Riboflavin metabolism | 0 | 1 | Long-term potentiation | 9 | 0.000190421 |
| ECM-receptor interaction | 22 | 0.04302708 | Vitamin B6 metabolism | 0 | 1 | Tryptophan metabolism | 7 | 0.000204257 |
| Wnt signaling pathway | 35 | 0.044425681 | Nicotinate and nicotinamide metabolism | 0 | 1 | cGMP-PKG signaling pathway | 15 | 0.000219807 |
| Cardiac muscle contraction | 21 | 0.045680433 | Pantothenate and CoA biosynthesis | 0 | 1 | Thyroid cancer | 6 | 0.000225086 |
| Caffeine metabolism | 3 | 0.048025608 | Biotin metabolism | 0 | 1 | Melanogenesis | 11 | 0.000268307 |
| Regulation of actin cytoskeleton | 50 | 0.050863495 | Lipoic acid metabolism | 0 | 1 | Drug metabolism - cytochrome P450 | 9 | 0.000268663 |
| Notch signaling pathway | 14 | 0.051068086 | Folate biosynthesis | 0 | 1 | Asthma | 6 | 0.000274072 |
| Jak-STAT signaling pathway | 38 | 0.052337163 | Retinol metabolism | 0 | 1 | Hematopoietic cell lineage | 10 | 0.000354931 |
| Tryptophan metabolism | 12 | 0.055996697 | Porphyrin and chlorophyll metabolism | 0 | 1 | Arginine biosynthesis | 5 | 0.00038229 |
| Graft-versus-host disease | 12 | 0.066251519 | Nitrogen metabolism | 0 | 1 | Tight junction | 13 | 0.00038758 |
| Natural killer cell mediated cytotoxicity | 32 | 0.076092069 | Sulfur metabolism | 0 | 1 | Metabolism of xenobiotics by cytochrome P450 | 9 | 0.000412744 |
| Alcoholism | 41 | 0.086482312 | Aminoacyl-tRNA biosynthesis | 0 | 1 | Long-term depression | 8 | 0.000504638 |
| Transcriptional misregulation in cancer | 41 | 0.086482312 | Metabolism of xenobiotics by cytochrome P450 | 0 | 1 | Renin-angiotensin system | 5 | 0.000600624 |
| Complement and coagulation cascades | 20 | 0.086857227 | Drug metabolism - cytochrome P450 | 0 | 1 | Cytosolic DNA-sensing pathway | 8 | 0.00070458 |
| RIG-I-like receptor signaling pathway | 18 | 0.089030843 | Drug metabolism - other enzymes | 0 | 1 | Chemical carcinogenesis | 9 | 0.000975618 |
| Mineral absorption | 14 | 0.091210683 | Biosynthesis of unsaturated fatty acids | 0 | 1 | Insulin secretion | 9 | 0.001263612 |
| Fanconi anemia pathway | 14 | 0.117089606 | Carbon metabolism | 0 | 1 | Metabolic pathways | 57 | 0.001491393 |
| Platelet activation | 28 | 0.134332281 | 2-Oxocarboxylic acid metabolism | 0 | 1 | Thyroid hormone synthesis | 8 | 0.001561173 |
| Shigellosis | 16 | 0.140947519 | Fatty acid metabolism | 0 | 1 | Bile secretion | 8 | 0.001561173 |
| Regulation of lipolysis in adipocytes | 14 | 0.146824466 | Biosynthesis of amino acids | 0 | 1 | Signaling pathways regulating pluripotency of stem cells | 12 | 0.001571873 |
| mTOR signaling pathway | 34 | 0.149667167 | EGFR tyrosine kinase inhibitor resistance | 0 | 1 | Regulation of lipolysis in adipocytes | 7 | 0.001660474 |
| HTLV-I infection | 55 | 0.150792073 | Endocrine resistance | 0 | 1 | Linoleic acid metabolism | 5 | 0.001818981 |
| Glutathione metabolism | 13 | 0.158092037 | Platinum drug resistance | 0 | 1 | Starch and sucrose metabolism | 7 | 0.001843471 |
| Thyroid hormone synthesis | 17 | 0.159093437 | ABC transporters | 0 | 1 | Circadian entrainment | 9 | 0.00275106 |
| Epstein-Barr virus infection | 43 | 0.16959977 | Ribosome biogenesis in eukaryotes | 0 | 1 | Renin secretion | 7 | 0.003599943 |
| Herpes simplex infection | 40 | 0.169921103 | Ribosome | 0 | 1 | Retrograde endocannabinoid signaling | 9 | 0.004158476 |
| Aldosterone-regulated sodium reabsorption | 10 | 0.177289911 | RNA transport | 0 | 1 | Peroxisome | 8 | 0.004198088 |
| Asthma | 8 | 0.182442391 | mRNA surveillance pathway | 0 | 1 | Hypertrophic cardiomyopathy (HCM) | 8 | 0.004198088 |
| Arginine biosynthesis | 6 | 0.183217905 | RNA degradation | 0 | 1 | TGF-beta signaling pathway | 8 | 0.004517795 |
| Tyrosine metabolism | 9 | 0.191921069 | RNA polymerase | 0 | 1 | Oocyte meiosis | 10 | 0.004982828 |
| Primary bile acid biosynthesis | 5 | 0.197336634 | Basal transcription factors | 0 | 1 | Vitamin digestion and absorption | 4 | 0.005983091 |
| Regulation of autophagy | 10 | 0.199357532 | DNA replication | 0 | 1 | Fatty acid biosynthesis | 3 | 0.006776541 |
| Insulin secretion | 19 | 0.225534856 | Spliceosome | 0 | 1 | PPAR signaling pathway | 7 | 0.0069154 |
| Hedgehog signaling pathway | 11 | 0.227356527 | Proteasome | 0 | 1 | Regulation of actin cytoskeleton | 14 | 0.007326104 |
| Pantothenate and CoA biosynthesis | 5 | 0.234040297 | Protein export | 0 | 1 | Arrhythmogenic right ventricular cardiomyopathy (ARVC) | 7 | 0.008015788 |
| Pancreatic secretion | 21 | 0.242581695 | PPAR signaling pathway | 0 | 1 | Caffeine metabolism | 2 | 0.009077225 |
| Chemokine signaling pathway | 39 | 0.24285528 | Base excision repair | 0 | 1 | Glutamatergic synapse | 9 | 0.0090987 |
| Fc gamma R-mediated phagocytosis | 20 | 0.258990789 | Nucleotide excision repair | 0 | 1 | Bacterial invasion of epithelial cells | 7 | 0.010596272 |
| Bacterial invasion of epithelial cells | 17 | 0.277538769 | Mismatch repair | 0 | 1 | Cell adhesion molecules (CAMs) | 10 | 0.013806559 |
| Pathogenic Escherichia coli infection | 12 | 0.322641492 | Homologous recombination | 0 | 1 | Adrenergic signaling in cardiomyocytes | 10 | 0.017205168 |
| Hippo signaling pathway | 31 | 0.334030038 | Non-homologous end-joining | 0 | 1 | Arginine and proline metabolism | 5 | 0.017633056 |
| Axon guidance | 35 | 0.365052434 | Fanconi anemia pathway | 0 | 1 | GABAergic synapse | 7 | 0.019610999 |
| Intestinal immune network for IgA production | 10 | 0.377676836 | MAPK signaling pathway | 0 | 1 | Dilated cardiomyopathy | 7 | 0.020738276 |
| Endocrine and other factor-regulated calcium reabsorption | 10 | 0.377676836 | ErbB signaling pathway | 0 | 1 | Glutathione metabolism | 5 | 0.022297482 |
| Porphyrin and chlorophyll metabolism | 9 | 0.380930196 | cAMP signaling pathway | 0 | 1 | Tyrosine metabolism | 4 | 0.022578546 |
| Huntington's disease | 38 | 0.382682412 | Cytokine-cytokine receptor interaction | 0 | 1 | Alcoholism | 11 | 0.02418808 |
| Phototransduction | 6 | 0.390225502 | Chemokine signaling pathway | 0 | 1 | Primary immunodeficiency | 4 | 0.024796515 |
| Viral carcinogenesis | 40 | 0.40240296 | HIF-1 signaling pathway | 0 | 1 | Gastric acid secretion | 6 | 0.027391996 |
| Phagosome | 30 | 0.411949954 | FoxO signaling pathway | 0 | 1 | Staphylococcus aureus infection | 5 | 0.027696437 |
| Endocytosis | 50 | 0.429005878 | Phosphatidylinositol signaling system | 0 | 1 | Pancreatic secretion | 7 | 0.029912502 |
| Phosphatidylinositol signaling system | 19 | 0.466070418 | Sphingolipid signaling pathway | 0 | 1 | Histidine metabolism | 3 | 0.037297684 |
| Progesterone-mediated oocyte maturation | 19 | 0.466070418 | Cell cycle | 0 | 1 | Fat digestion and absorption | 4 | 0.037811446 |
| Antigen processing and presentation | 15 | 0.471448431 | Oocyte meiosis | 0 | 1 | ECM-receptor interaction | 6 | 0.04225375 |
| Vitamin digestion and absorption | 5 | 0.472551114 | p53 signaling pathway | 0 | 1 | Maturity onset diabetes of the young | 3 | 0.045794815 |
| Alanine, aspartate and glutamate metabolism | 7 | 0.486299708 | Ubiquitin mediated proteolysis | 0 | 1 | Fatty acid degradation | 4 | 0.047182944 |
| Parkinson's disease | 27 | 0.489541026 | Sulfur relay system | 0 | 1 | Hippo signaling pathway | 9 | 0.04953187 |
| Epithelial cell signaling in Helicobacter pylori infection | 13 | 0.509571232 | SNARE interactions in vesicular transport | 0 | 1 | Neuroactive ligand-receptor interaction | 14 | 0.051105166 |
| Steroid biosynthesis | 4 | 0.527861698 | Regulation of autophagy | 0 | 1 | Glyoxylate and dicarboxylate metabolism | 3 | 0.0551605 |
| AMPK signaling pathway | 23 | 0.531173062 | Protein processing in endoplasmic reticulum | 0 | 1 | Endocrine and other factor-regulated calcium reabsorption | 4 | 0.05772599 |
| Galactose metabolism | 6 | 0.532201907 | Lysosome | 0 | 1 | Systemic lupus erythematosus | 8 | 0.057779358 |
| Biosynthesis of amino acids | 14 | 0.545446925 | Endocytosis | 0 | 1 | Salivary secretion | 6 | 0.058759142 |
| Carbon metabolism | 21 | 0.545573213 | Peroxisome | 0 | 1 | Fatty acid metabolism | 4 | 0.061497896 |
| Cell cycle | 23 | 0.548300039 | mTOR signaling pathway | 0 | 1 | Carbon metabolism | 7 | 0.062545928 |
| D-Glutamine and D-glutamate metabolism | 1 | 0.562076587 | PI3K-Akt signaling pathway | 0 | 1 | Endocytosis | 13 | 0.0628589 |
| Riboflavin metabolism | 1 | 0.562076587 | Apoptosis | 0 | 1 | Glycerophospholipid metabolism | 6 | 0.075585159 |
| Staphylococcus aureus infection | 10 | 0.590208367 | Longevity regulating pathway | 0 | 1 | Mineral absorption | 4 | 0.077844666 |
| Basal cell carcinoma | 10 | 0.590208367 | Longevity regulating pathway - multiple species | 0 | 1 | Fructose and mannose metabolism | 3 | 0.082143225 |
| Ovarian steroidogenesis | 9 | 0.604234184 | Apoptosis - multiple species | 0 | 1 | Base excision repair | 3 | 0.082143225 |
| Leukocyte transendothelial migration | 21 | 0.631628842 | Cardiac muscle contraction | 0 | 1 | Phosphatidylinositol signaling system | 6 | 0.084924152 |
| Ubiquinone and other terpenoid-quinone biosynthesis | 2 | 0.63643133 | Wnt signaling pathway | 0 | 1 | Pathogenic Escherichia coli infection | 4 | 0.091384989 |
| Phenylalanine metabolism | 3 | 0.639647465 | Dorso-ventral axis formation | 0 | 1 | Antigen processing and presentation | 5 | 0.091543029 |
| Butirosin and neomycin biosynthesis | 1 | 0.643775565 | Notch signaling pathway | 0 | 1 | Alanine, aspartate and glutamate metabolism | 3 | 0.094253617 |
| Linoleic acid metabolism | 5 | 0.651731831 | Hedgehog signaling pathway | 0 | 1 | Phenylalanine metabolism | 2 | 0.096671447 |
| 2-Oxocarboxylic acid metabolism | 3 | 0.679575642 | TGF-beta signaling pathway | 0 | 1 | Complement and coagulation cascades | 5 | 0.099468309 |
| Tight junction | 24 | 0.696545115 | Axon guidance | 0 | 1 | 2-Oxocarboxylic acid metabolism | 2 | 0.106600771 |
| Arginine and proline metabolism | 8 | 0.717824033 | Osteoclast differentiation | 0 | 1 | Riboflavin metabolism | 1 | 0.118839933 |
| Maturity onset diabetes of the young | 4 | 0.741714301 | Hippo signaling pathway -multiple species | 0 | 1 | Regulation of autophagy | 3 | 0.127406997 |
| Arachidonic acid metabolism | 10 | 0.743803079 | Focal adhesion | 0 | 1 | Retinol metabolism | 4 | 0.143690892 |
| One carbon pool by folate | 3 | 0.749337818 | ECM-receptor interaction | 0 | 1 | Butirosin and neomycin biosynthesis | 1 | 0.14628061 |
| NF-kappa B signaling pathway | 15 | 0.758158762 | Adherens junction | 0 | 1 | Protein digestion and absorption | 5 | 0.148758436 |
| Cysteine and methionine metabolism | 7 | 0.759820757 | Tight junction | 0 | 1 | Morphine addiction | 5 | 0.153680888 |
| Cytosolic DNA-sensing pathway | 10 | 0.762158052 | Gap junction | 0 | 1 | Parkinson's disease | 7 | 0.154357072 |
| Glycosphingolipid biosynthesis - globo series | 2 | 0.766145833 | Signaling pathways regulating pluripotency of stem cells | 0 | 1 | Glycolysis / Gluconeogenesis | 4 | 0.155326275 |
| Folate biosynthesis | 2 | 0.766145833 | Antigen processing and presentation | 0 | 1 | Hedgehog signaling pathway | 3 | 0.171704758 |
| Lysine degradation | 8 | 0.779215715 | Toll-like receptor signaling pathway | 0 | 1 | alpha-Linolenic acid metabolism | 2 | 0.181995285 |
| Oocyte meiosis | 20 | 0.785831544 | NOD-like receptor signaling pathway | 0 | 1 | Phagosome | 7 | 0.199962351 |
| Glyoxylate and dicarboxylate metabolism | 4 | 0.794009832 | RIG-I-like receptor signaling pathway | 0 | 1 | Phototransduction | 2 | 0.204783269 |
| Protein processing in endoplasmic reticulum | 27 | 0.803791173 | Cytosolic DNA-sensing pathway | 0 | 1 | Biosynthesis of amino acids | 4 | 0.205024086 |
| Pentose phosphate pathway | 4 | 0.816774312 | Jak-STAT signaling pathway | 0 | 1 | Dorso-ventral axis formation | 2 | 0.216292712 |
| Nicotinate and nicotinamide metabolism | 4 | 0.816774312 | Hematopoietic cell lineage | 0 | 1 | Pentose phosphate pathway | 2 | 0.22785818 |
| Primary immunodeficiency | 5 | 0.82785815 | T cell receptor signaling pathway | 0 | 1 | Protein processing in endoplasmic reticulum | 7 | 0.254845569 |
| Protein export | 3 | 0.830512918 | B cell receptor signaling pathway | 0 | 1 | Sulfur metabolism | 1 | 0.271219673 |
| Lysosome | 19 | 0.849930897 | Fc epsilon RI signaling pathway | 0 | 1 | Glycerolipid metabolism | 3 | 0.278333404 |
| Histidine metabolism | 3 | 0.852030335 | Fc gamma R-mediated phagocytosis | 0 | 1 | Taurine and hypotaurine metabolism | 1 | 0.293928147 |
| ABC transporters | 6 | 0.854616677 | Intestinal immune network for IgA production | 0 | 1 | Axon guidance | 7 | 0.30856125 |
| Vasopressin-regulated water reabsorption | 6 | 0.854616677 | Circadian rhythm | 0 | 1 | Pentose and glucuronate interconversions | 2 | 0.309262032 |
| beta-Alanine metabolism | 4 | 0.856091208 | Circadian entrainment | 0 | 1 | Glycine, serine and threonine metabolism | 2 | 0.355163911 |
| Sulfur metabolism | 1 | 0.873174289 | Long-term potentiation | 0 | 1 | Nicotine addiction | 2 | 0.355163911 |
| Other glycan degradation | 2 | 0.875379663 | Synaptic vesicle cycle | 0 | 1 | Porphyrin and chlorophyll metabolism | 2 | 0.377720761 |
| Inositol phosphate metabolism | 10 | 0.876272954 | Neurotrophin signaling pathway | 0 | 1 | Glycosaminoglycan biosynthesis - keratan sulfate | 1 | 0.377922558 |
| Bile secretion | 10 | 0.876272954 | Glutamatergic synapse | 0 | 1 | Proteasome | 2 | 0.399944026 |
| Carbohydrate digestion and absorption | 6 | 0.882544146 | Cholinergic synapse | 0 | 1 | Vasopressin-regulated water reabsorption | 2 | 0.399944026 |
| PPAR signaling pathway | 10 | 0.886760193 | Dopaminergic synapse | 0 | 1 | Ether lipid metabolism | 2 | 0.410916598 |
| Glycine, serine and threonine metabolism | 5 | 0.890699224 | Long-term depression | 0 | 1 | Selenocompound metabolism | 1 | 0.416105829 |
| Glycosaminoglycan degradation | 2 | 0.894116517 | Olfactory transduction | 0 | 1 | Nitrogen metabolism | 1 | 0.416105829 |
| Glycosaminoglycan biosynthesis - chondroitin sulfate / dermatan sulfate | 2 | 0.910203039 | Phototransduction | 0 | 1 | Drug metabolism - other enzymes | 2 | 0.421789863 |
| Butanoate metabolism | 3 | 0.915938838 | Insulin signaling pathway | 0 | 1 | Sphingolipid metabolism | 2 | 0.432559367 |
| Hippo signaling pathway -multiple species | 3 | 0.915938838 | Insulin secretion | 0 | 1 | Other glycan degradation | 1 | 0.434312009 |
| Drug metabolism - cytochrome P450 | 9 | 0.91752407 | GnRH signaling pathway | 0 | 1 | Amino sugar and nucleotide sugar metabolism | 2 | 0.443221024 |
| Chemical carcinogenesis | 11 | 0.918582375 | Progesterone-mediated oocyte maturation | 0 | 1 | Steroid biosynthesis | 1 | 0.469043979 |
| Peroxisome | 11 | 0.925697767 | Estrogen signaling pathway | 0 | 1 | Vibrio cholerae infection | 2 | 0.474523269 |
| Proximal tubule bicarbonate reclamation | 2 | 0.945758552 | Melanogenesis | 0 | 1 | Terpenoid backbone biosynthesis | 1 | 0.501649681 |
| Drug metabolism - other enzymes | 5 | 0.947709503 | Prolactin signaling pathway | 0 | 1 | Basal cell carcinoma | 2 | 0.514559663 |
| Sphingolipid metabolism | 5 | 0.954017862 | Thyroid hormone synthesis | 0 | 1 | Protein export | 1 | 0.517196055 |
| Glycosphingolipid biosynthesis - ganglio series | 1 | 0.954871537 | Thyroid hormone signaling pathway | 0 | 1 | Mismatch repair | 1 | 0.517196055 |
| Pyruvate metabolism | 4 | 0.956085485 | Adipocytokine signaling pathway | 0 | 1 | Glycosaminoglycan biosynthesis - heparan sulfate / heparin | 1 | 0.532258904 |
| Amino sugar and nucleotide sugar metabolism | 5 | 0.959624664 | Glucagon signaling pathway | 0 | 1 | Glycosphingolipid biosynthesis - lacto and neolacto series | 1 | 0.560993537 |
| Fructose and mannose metabolism | 3 | 0.960223316 | Type II diabetes mellitus | 0 | 1 | Ascorbate and aldarate metabolism | 1 | 0.574693932 |
| Base excision repair | 3 | 0.960223316 | Insulin resistance | 0 | 1 | Homologous recombination | 1 | 0.600829118 |
| alpha-Linolenic acid metabolism | 2 | 0.961510282 | Non-alcoholic fatty liver disease (NAFLD) | 0 | 1 | Circadian rhythm | 1 | 0.613289947 |
| Fat digestion and absorption | 4 | 0.96183366 | Type I diabetes mellitus | 0 | 1 | beta-Alanine metabolism | 1 | 0.625362955 |
| Synaptic vesicle cycle | 7 | 0.963113089 | Maturity onset diabetes of the young | 0 | 1 | Inositol phosphate metabolism | 2 | 0.653538685 |
| Selenocompound metabolism | 1 | 0.970154389 | Aldosterone-regulated sodium reabsorption | 0 | 1 | DNA replication | 1 | 0.680319091 |
| Nitrogen metabolism | 1 | 0.970154389 | Endocrine and other factor-regulated calcium reabsorption | 0 | 1 | Pyruvate metabolism | 1 | 0.718436008 |
| Collecting duct acid secretion | 2 | 0.972816813 | Vasopressin-regulated water reabsorption | 0 | 1 | Lysosome | 3 | 0.742066032 |
| Metabolism of xenobiotics by cytochrome P450 | 8 | 0.973971602 | Proximal tubule bicarbonate reclamation | 0 | 1 | ABC transporters | 1 | 0.752020465 |
| Glycerolipid metabolism | 6 | 0.974973361 | Collecting duct acid secretion | 0 | 1 | Cysteine and methionine metabolism | 1 | 0.759772825 |
| Pentose and glucuronate interconversions | 3 | 0.975057142 | Salivary secretion | 0 | 1 | Nucleotide excision repair | 1 | 0.774560171 |
| Ether lipid metabolism | 4 | 0.978521934 | Gastric acid secretion | 0 | 1 | Valine, leucine and isoleucine degradation | 1 | 0.78160994 |
| Systemic lupus erythematosus | 16 | 0.986714442 | Carbohydrate digestion and absorption | 0 | 1 | Notch signaling pathway | 1 | 0.78160994 |
| Starch and sucrose metabolism | 5 | 0.988186704 | Protein digestion and absorption | 0 | 1 | Lysine degradation | 1 | 0.80767835 |
| Terpenoid backbone biosynthesis | 1 | 0.989388256 | Vitamin digestion and absorption | 0 | 1 | Fanconi anemia pathway | 1 | 0.819524739 |
| Glycerophospholipid metabolism | 10 | 0.989521669 | Mineral absorption | 0 | 1 | Aminoacyl-tRNA biosynthesis | 1 | 0.876791556 |
| Vibrio cholerae infection | 4 | 0.991252927 | Alzheimer's disease | 0 | 1 | Purine metabolism | 3 | 0.911644304 |
| SNARE interactions in vesicular transport | 2 | 0.992189521 | Parkinson's disease | 0 | 1 | RNA degradation | 1 | 0.913202799 |
| Oxidative phosphorylation | 15 | 0.992553218 | Amyotrophic lateral sclerosis (ALS) | 0 | 1 | Ribosome biogenesis in eukaryotes | 1 | 0.928310834 |
| Glycosaminoglycan biosynthesis - heparan sulfate / heparin | 1 | 0.992984125 | Huntington's disease | 0 | 1 | Taste transduction | 1 | 0.928310834 |
| Glycosphingolipid biosynthesis - lacto and neolacto series | 1 | 0.995361919 | Prion diseases | 0 | 1 | Ubiquitin mediated proteolysis | 2 | 0.930317291 |
| Steroid hormone biosynthesis | 4 | 0.997066912 | Cocaine addiction | 0 | 1 | Pyrimidine metabolism | 1 | 0.963323914 |
| Glycolysis / Gluconeogenesis | 5 | 0.997274392 | Amphetamine addiction | 0 | 1 | RNA transport | 2 | 0.971785667 |
| Homologous recombination | 1 | 0.997507411 | Alcoholism | 0 | 1 | Oxidative phosphorylation | 1 | 0.985497836 |
| Other types of O-glycan biosynthesis | 1 | 0.998352556 | Bacterial invasion of epithelial cells | 0 | 1 | Olfactory transduction | 2 | 0.999978042 |
| RNA polymerase | 1 | 0.998352556 | Vibrio cholerae infection | 0 | 1 | Citrate cycle (TCA cycle) | 0 | 1 |
| Propanoate metabolism | 1 | 0.998660705 | Epithelial cell signaling in Helicobacter pylori infection | 0 | 1 | Fatty acid elongation | 0 | 1 |
| Proteasome | 2 | 0.998759147 | Pathogenic Escherichia coli infection | 0 | 1 | Synthesis and degradation of ketone bodies | 0 | 1 |
| Basal transcription factors | 2 | 0.998970563 | Shigellosis | 0 | 1 | Primary bile acid biosynthesis | 0 | 1 |
| Nucleotide excision repair | 2 | 0.999292377 | Salmonella infection | 0 | 1 | Ubiquinone and other terpenoid-quinone biosynthesis | 0 | 1 |
| Valine, leucine and isoleucine degradation | 2 | 0.999413676 | Chagas disease (American trypanosomiasis) | 0 | 1 | Valine, leucine and isoleucine biosynthesis | 0 | 1 |
| Ubiquitin mediated proteolysis | 12 | 0.999628842 | African trypanosomiasis | 0 | 1 | Lysine biosynthesis | 0 | 1 |
| Retinol metabolism | 3 | 0.999819219 | Toxoplasmosis | 0 | 1 | Phenylalanine, tyrosine and tryptophan biosynthesis | 0 | 1 |
| Pyrimidine metabolism | 7 | 0.999868038 | Hepatitis C | 0 | 1 | D-Glutamine and D-glutamate metabolism | 0 | 1 |
| Fatty acid degradation | 1 | 0.999888594 | Hepatitis B | 0 | 1 | D-Arginine and D-ornithine metabolism | 0 | 1 |
| Purine metabolism | 15 | 0.999938456 | Measles | 0 | 1 | N-Glycan biosynthesis | 0 | 1 |
| mRNA surveillance pathway | 5 | 0.999940043 | Influenza A | 0 | 1 | Mucin type O-Glycan biosynthesis | 0 | 1 |
| Fatty acid metabolism | 1 | 0.999951403 | Herpes simplex infection | 0 | 1 | Other types of O-glycan biosynthesis | 0 | 1 |
| N-Glycan biosynthesis | 1 | 0.999960508 | Epstein-Barr virus infection | 0 | 1 | Glycosaminoglycan degradation | 0 | 1 |
| RNA transport | 14 | 0.999966981 | Transcriptional misregulation in cancer | 0 | 1 | Glycosaminoglycan biosynthesis - chondroitin sulfate / dermatan sulfate | 0 | 1 |
| Aminoacyl-tRNA biosynthesis | 2 | 0.999981202 | Viral carcinogenesis | 0 | 1 | Glycosylphosphatidylinositol(GPI)-anchor biosynthesis | 0 | 1 |
| RNA degradation | 2 | 0.999997791 | Proteoglycans in cancer | 0 | 1 | Glycosphingolipid biosynthesis - globo series | 0 | 1 |
| Ribosome | 5 | 0.999999976 | Colorectal cancer | 0 | 1 | Glycosphingolipid biosynthesis - ganglio series | 0 | 1 |
| Spliceosome | 4 | 0.999999996 | Renal cell carcinoma | 0 | 1 | Propanoate metabolism | 0 | 1 |
| Olfactory transduction | 11 | 1 | Pancreatic cancer | 0 | 1 | Butanoate metabolism | 0 | 1 |
| Metabolic pathways | 137 | 1 | Endometrial cancer | 0 | 1 | One carbon pool by folate | 0 | 1 |
| Ascorbate and aldarate metabolism | 0 | 1 | Glioma | 0 | 1 | Thiamine metabolism | 0 | 1 |
| Fatty acid biosynthesis | 0 | 1 | Prostate cancer | 0 | 1 | Vitamin B6 metabolism | 0 | 1 |
| Fatty acid elongation | 0 | 1 | Thyroid cancer | 0 | 1 | Nicotinate and nicotinamide metabolism | 0 | 1 |
| Synthesis and degradation of ketone bodies | 0 | 1 | Basal cell carcinoma | 0 | 1 | Pantothenate and CoA biosynthesis | 0 | 1 |
| Valine, leucine and isoleucine biosynthesis | 0 | 1 | Melanoma | 0 | 1 | Biotin metabolism | 0 | 1 |
| Lysine biosynthesis | 0 | 1 | Bladder cancer | 0 | 1 | Lipoic acid metabolism | 0 | 1 |
| Phenylalanine, tyrosine and tryptophan biosynthesis | 0 | 1 | Chronic myeloid leukemia | 0 | 1 | Folate biosynthesis | 0 | 1 |
| D-Arginine and D-ornithine metabolism | 0 | 1 | Acute myeloid leukemia | 0 | 1 | Biosynthesis of unsaturated fatty acids | 0 | 1 |
| Mucin type O-Glycan biosynthesis | 0 | 1 | Non-small cell lung cancer | 0 | 1 | Ribosome | 0 | 1 |
| Glycosaminoglycan biosynthesis - keratan sulfate | 0 | 1 | Central carbon metabolism in cancer | 0 | 1 | mRNA surveillance pathway | 0 | 1 |
| Glycosylphosphatidylinositol(GPI)-anchor biosynthesis | 0 | 1 | Choline metabolism in cancer | 0 | 1 | RNA polymerase | 0 | 1 |
| Thiamine metabolism | 0 | 1 | Asthma | 0 | 1 | Basal transcription factors | 0 | 1 |
| Vitamin B6 metabolism | 0 | 1 | Autoimmune thyroid disease | 0 | 1 | Spliceosome | 0 | 1 |
| Biotin metabolism | 0 | 1 | Inflammatory bowel disease (IBD) | 0 | 1 | Non-homologous end-joining | 0 | 1 |
| Lipoic acid metabolism | 0 | 1 | Systemic lupus erythematosus | 0 | 1 | Sulfur relay system | 0 | 1 |
| Biosynthesis of unsaturated fatty acids | 0 | 1 | Allograft rejection | 0 | 1 | SNARE interactions in vesicular transport | 0 | 1 |
| Ribosome biogenesis in eukaryotes | 0 | 1 | Graft-versus-host disease | 0 | 1 | Cardiac muscle contraction | 0 | 1 |
| DNA replication | 0 | 1 | Primary immunodeficiency | 0 | 1 | Hippo signaling pathway -multiple species | 0 | 1 |
| Mismatch repair | 0 | 1 | Hypertrophic cardiomyopathy (HCM) | 0 | 1 | Synaptic vesicle cycle | 0 | 1 |
| Non-homologous end-joining | 0 | 1 | Arrhythmogenic right ventricular cardiomyopathy (ARVC) | 0 | 1 | Proximal tubule bicarbonate reclamation | 0 | 1 |
| Sulfur relay system | 0 | 1 | Dilated cardiomyopathy | 0 | 1 | Collecting duct acid secretion | 0 | 1 |

Pathways in M29

| **Symptom** | **overlap** | **p_value** | **Drug** | **overlap** | **p_value** | **Herb** | **overlap** | **p_value** |
| --- | --- | --- | --- | --- | --- | --- | --- | --- |
| MicroRNAs in cancer | 160 | 1.53025E-63 | Neuroactive ligand-receptor interaction | 59 | 2.73493E-48 | AGE-RAGE signaling pathway in diabetic complications | 26 | 4.48187E-24 |
| Pathways in cancer | 153 | 7.0296E-38 | Calcium signaling pathway | 38 | 1.21024E-30 | Hepatitis B | 26 | 1.09588E-19 |
| Non-small cell lung cancer | 47 | 9.45232E-33 | Nicotine addiction | 21 | 6.51921E-27 | Pathways in cancer | 37 | 8.33189E-18 |
| Small cell lung cancer | 58 | 5.4521E-31 | GABAergic synapse | 26 | 3.69913E-25 | HIF-1 signaling pathway | 19 | 6.16142E-15 |
| Proteoglycans in cancer | 94 | 2.22694E-30 | Morphine addiction | 26 | 9.83353E-25 | TNF signaling pathway | 19 | 2.18746E-14 |
| Prostate cancer | 58 | 9.1708E-30 | Retrograde endocannabinoid signaling | 26 | 1.96025E-23 | Leishmaniasis | 15 | 7.57318E-13 |
| EGFR tyrosine kinase inhibitor resistance | 53 | 1.991E-27 | Serotonergic synapse | 22 | 3.1567E-17 | Malaria | 13 | 9.51807E-13 |
| Bladder cancer | 36 | 8.80201E-27 | Adrenergic signaling in cardiomyocytes | 22 | 1.47199E-14 | Colorectal cancer | 14 | 1.39008E-12 |
| Chronic myeloid leukemia | 49 | 3.24023E-26 | Taste transduction | 17 | 7.09589E-14 | Chagas disease (American trypanosomiasis) | 17 | 1.39777E-12 |
| Pancreatic cancer | 45 | 1.2887E-24 | Hypertrophic cardiomyopathy (HCM) | 16 | 1.07786E-12 | Bladder cancer | 12 | 2.00567E-12 |
| Glioma | 44 | 6.7572E-24 | Dilated cardiomyopathy | 16 | 3.35067E-12 | PI3K-Akt signaling pathway | 28 | 2.8195E-12 |
| Hepatitis B | 68 | 9.66934E-23 | cAMP signaling pathway | 22 | 7.00837E-12 | Proteoglycans in cancer | 22 | 4.15439E-12 |
| Endometrial cancer | 38 | 1.04241E-22 | cGMP-PKG signaling pathway | 20 | 1.53187E-11 | FoxO signaling pathway | 18 | 8.26525E-12 |
| Apoptosis | 64 | 6.21112E-21 | Arrhythmogenic right ventricular cardiomyopathy (ARVC) | 14 | 3.86064E-11 | HTLV-I infection | 24 | 9.08118E-12 |
| Melanoma | 42 | 1.51526E-19 | Renin secretion | 13 | 7.66658E-11 | Amoebiasis | 15 | 9.48889E-11 |
| AGE-RAGE signaling pathway in diabetic complications | 51 | 2.68256E-19 | Cardiac muscle contraction | 14 | 8.14192E-11 | Toxoplasmosis | 16 | 1.22695E-10 |
| HIF-1 signaling pathway | 51 | 8.09708E-19 | Oxytocin signaling pathway | 17 | 2.6912E-09 | Prostate cancer | 14 | 2.45221E-10 |
| Toxoplasmosis | 55 | 1.20146E-18 | Type II diabetes mellitus | 10 | 9.74855E-09 | Rheumatoid arthritis | 14 | 2.45221E-10 |
| Endocrine resistance | 49 | 1.33435E-18 | Aldosterone synthesis and secretion | 12 | 1.85719E-08 | Insulin resistance | 15 | 3.86237E-10 |
| Colorectal cancer | 38 | 1.4673E-18 | Complement and coagulation cascades | 11 | 1.4306E-07 | NF-kappa B signaling pathway | 14 | 3.87302E-10 |
| PI3K-Akt signaling pathway | 105 | 1.12394E-17 | MAPK signaling pathway | 19 | 1.44429E-07 | Osteoclast differentiation | 16 | 6.0246E-10 |
| ErbB signaling pathway | 45 | 1.86408E-17 | Vascular smooth muscle contraction | 12 | 1.50675E-06 | Amyotrophic lateral sclerosis (ALS) | 11 | 6.4667E-10 |
| FoxO signaling pathway | 57 | 3.59425E-17 | Salivary secretion | 10 | 4.01311E-06 | Inflammatory bowel disease (IBD) | 12 | 7.18493E-10 |
| Focal adhesion | 73 | 1.58847E-16 | Cholinergic synapse | 11 | 4.55417E-06 | Endocrine resistance | 14 | 7.98666E-10 |
| Acute myeloid leukemia | 34 | 3.16171E-16 | Insulin secretion | 9 | 2.00703E-05 | EGFR tyrosine kinase inhibitor resistance | 13 | 8.49481E-10 |
| Platinum drug resistance | 39 | 1.26263E-15 | Regulation of lipolysis in adipocytes | 7 | 5.86696E-05 | Tuberculosis | 18 | 1.01429E-09 |
| Central carbon metabolism in cancer | 34 | 2.17936E-13 | Dopaminergic synapse | 10 | 0.000104559 | Legionellosis | 11 | 1.53196E-09 |
| VEGF signaling pathway | 32 | 3.36472E-13 | Bile secretion | 7 | 0.000268379 | Apoptosis | 16 | 1.62885E-09 |
| p53 signaling pathway | 34 | 6.55033E-13 | Steroid hormone biosynthesis | 6 | 0.000571449 | Small cell lung cancer | 13 | 1.82984E-09 |
| Neurotrophin signaling pathway | 46 | 5.12002E-12 | Gap junction | 7 | 0.000992519 | T cell receptor signaling pathway | 14 | 2.32907E-09 |
| T cell receptor signaling pathway | 41 | 3.71506E-11 | GnRH signaling pathway | 7 | 0.001209826 | Toll-like receptor signaling pathway | 14 | 2.64427E-09 |
| TNF signaling pathway | 42 | 5.02285E-11 | Carbohydrate digestion and absorption | 5 | 0.001325154 | African trypanosomiasis | 9 | 3.45041E-09 |
| Prolactin signaling pathway | 32 | 9.90422E-11 | Gastric acid secretion | 6 | 0.002065482 | Pertussis | 12 | 4.02257E-09 |
| Measles | 47 | 1.51237E-10 | HIF-1 signaling pathway | 7 | 0.002475062 | Focal adhesion | 18 | 9.18743E-09 |
| Thyroid hormone signaling pathway | 43 | 1.73032E-10 | Butirosin and neomycin biosynthesis | 2 | 0.003084545 | Salmonella infection | 12 | 2.00268E-08 |
| Renal cell carcinoma | 30 | 3.04777E-10 | Amphetamine addiction | 5 | 0.006904753 | Influenza A | 16 | 4.15555E-08 |
| Ras signaling pathway | 66 | 3.41195E-10 | Central carbon metabolism in cancer | 5 | 0.006904753 | Epstein-Barr virus infection | 17 | 4.55039E-08 |
| Chagas disease (American trypanosomiasis) | 39 | 4.59754E-10 | Circadian entrainment | 6 | 0.007121195 | Platinum drug resistance | 11 | 4.67548E-08 |
| Fc epsilon RI signaling pathway | 29 | 1.60751E-09 | Collecting duct acid secretion | 3 | 0.012083132 | Transcriptional misregulation in cancer | 16 | 5.71703E-08 |
| Signaling pathways regulating pluripotency of stem cells | 45 | 1.10195E-08 | Rap1 signaling pathway | 9 | 0.013705123 | MAPK signaling pathway | 19 | 5.95276E-08 |
| B cell receptor signaling pathway | 29 | 1.18923E-08 | Linoleic acid metabolism | 3 | 0.014706047 | Endometrial cancer | 9 | 1.86187E-07 |
| Thyroid cancer | 17 | 1.33984E-08 | Regulation of actin cytoskeleton | 9 | 0.015327946 | p53 signaling pathway | 10 | 2.18683E-07 |
| Rap1 signaling pathway | 58 | 3.20408E-08 | Prostate cancer | 5 | 0.021699438 | B cell receptor signaling pathway | 10 | 3.30046E-07 |
| Sphingolipid signaling pathway | 39 | 4.84548E-08 | AMPK signaling pathway | 6 | 0.02317052 | Thyroid hormone signaling pathway | 12 | 6.98051E-07 |
| Longevity regulating pathway - multiple species | 26 | 5.48546E-08 | Pancreatic secretion | 5 | 0.02894501 | VEGF signaling pathway | 9 | 7.69859E-07 |
| HTLV-I infection | 66 | 7.70694E-08 | Glioma | 4 | 0.02900334 | Sphingolipid signaling pathway | 12 | 8.37607E-07 |
| Hepatitis C | 41 | 1.16388E-07 | Inflammatory mediator regulation of TRP channels | 5 | 0.031264707 | Neurotrophin signaling pathway | 12 | 8.37607E-07 |
| Leishmaniasis | 27 | 2.18322E-07 | Aldosterone-regulated sodium reabsorption | 3 | 0.032307936 | Estrogen signaling pathway | 11 | 8.46047E-07 |
| mTOR signaling pathway | 44 | 3.71277E-07 | Glucagon signaling pathway | 5 | 0.034957634 | Pancreatic cancer | 9 | 1.52791E-06 |
| MAPK signaling pathway | 63 | 5.78149E-07 | Leishmaniasis | 4 | 0.040067176 | Non-alcoholic fatty liver disease (NAFLD) | 13 | 1.61725E-06 |
| Amyotrophic lateral sclerosis (ALS) | 21 | 7.97039E-07 | Pertussis | 4 | 0.045449501 | ErbB signaling pathway | 10 | 2.19882E-06 |
| Amoebiasis | 32 | 8.76301E-07 | Insulin resistance | 5 | 0.046082152 | Drug metabolism - cytochrome P450 | 9 | 2.2395E-06 |
| Apoptosis - multiple species | 16 | 1.18218E-06 | Pathways in cancer | 12 | 0.053520573 | Allograft rejection | 7 | 2.40271E-06 |
| Influenza A | 47 | 1.32562E-06 | Cocaine addiction | 3 | 0.057281497 | Cytokine-cytokine receptor interaction | 17 | 2.49101E-06 |
| Toll-like receptor signaling pathway | 33 | 1.54063E-06 | Ovarian steroidogenesis | 3 | 0.060164275 | Metabolism of xenobiotics by cytochrome P450 | 9 | 3.61965E-06 |
| NF-kappa B signaling pathway | 30 | 1.5544E-06 | Renin-angiotensin system | 2 | 0.063224379 | Chemical carcinogenesis | 9 | 9.587E-06 |
| Cell cycle | 36 | 3.29796E-06 | Proximal tubule bicarbonate reclamation | 2 | 0.063224379 | Intestinal immune network for IgA production | 7 | 1.27234E-05 |
| Choline metabolism in cancer | 31 | 4.42161E-06 | Small cell lung cancer | 4 | 0.068451822 | Glioma | 8 | 1.29399E-05 |
| Osteoclast differentiation | 37 | 4.93582E-06 | Staphylococcus aureus infection | 3 | 0.075562844 | Fc epsilon RI signaling pathway | 8 | 1.62461E-05 |
| Alzheimer's disease | 44 | 5.85931E-06 | Maturity onset diabetes of the young | 2 | 0.078460699 | Renal cell carcinoma | 8 | 1.62461E-05 |
| Malaria | 19 | 7.67853E-06 | Alzheimer's disease | 6 | 0.081606427 | Central carbon metabolism in cancer | 8 | 1.62461E-05 |
| Epstein-Barr virus infection | 49 | 1.30753E-05 | Caffeine metabolism | 1 | 0.086489671 | Measles | 11 | 1.77224E-05 |
| Hematopoietic cell lineage | 27 | 1.43987E-05 | Endocrine resistance | 4 | 0.096394061 | Serotonergic synapse | 10 | 1.94202E-05 |
| Inflammatory bowel disease (IBD) | 22 | 1.95138E-05 | Arachidonic acid metabolism | 3 | 0.099693935 | Adipocytokine signaling pathway | 8 | 2.25194E-05 |
| Natural killer cell mediated cytotoxicity | 36 | 2.22458E-05 | Estrogen signaling pathway | 4 | 0.101973879 | Prion diseases | 6 | 2.35943E-05 |
| Estrogen signaling pathway | 29 | 2.40903E-05 | Vitamin B6 metabolism | 1 | 0.102873136 | Melanoma | 8 | 2.5016E-05 |
| Longevity regulating pathway | 27 | 6.6352E-05 | Insulin signaling pathway | 5 | 0.104530166 | Alzheimer's disease | 12 | 2.77071E-05 |
| Pertussis | 23 | 7.50415E-05 | Galactose metabolism | 2 | 0.106019824 | Prolactin signaling pathway | 8 | 2.77405E-05 |
| Chemokine signaling pathway | 44 | 9.92096E-05 | Chagas disease (American trypanosomiasis) | 4 | 0.116553965 | Longevity regulating pathway | 9 | 2.92349E-05 |
| Progesterone-mediated oocyte maturation | 27 | 0.000144481 | Adipocytokine signaling pathway | 3 | 0.130503154 | Chronic myeloid leukemia | 8 | 3.07093E-05 |
| Legionellosis | 18 | 0.000178568 | Melanoma | 3 | 0.134568353 | NOD-like receptor signaling pathway | 7 | 4.6378E-05 |
| Tuberculosis | 41 | 0.000241636 | Adherens junction | 3 | 0.14702039 | Caffeine metabolism | 3 | 4.67958E-05 |
| NOD-like receptor signaling pathway | 18 | 0.000295671 | Bladder cancer | 2 | 0.166984796 | MicroRNAs in cancer | 16 | 4.75269E-05 |
| Leukocyte transendothelial migration | 30 | 0.000302094 | Platelet activation | 4 | 0.175670041 | Steroid hormone biosynthesis | 7 | 5.19974E-05 |
| Transcriptional misregulation in cancer | 41 | 0.000310926 | Oocyte meiosis | 4 | 0.17921865 | Graft-versus-host disease | 6 | 6.0076E-05 |
| Aldosterone-regulated sodium reabsorption | 14 | 0.000313758 | Chemical carcinogenesis | 3 | 0.18189376 | Rap1 signaling pathway | 13 | 6.04525E-05 |
| Type II diabetes mellitus | 16 | 0.000318731 | Amino sugar and nucleotide sugar metabolism | 2 | 0.212473475 | Herpes simplex infection | 12 | 7.12852E-05 |
| Prion diseases | 13 | 0.000347851 | FoxO signaling pathway | 4 | 0.215939003 | Oxytocin signaling pathway | 11 | 7.17752E-05 |
| Jak-STAT signaling pathway | 37 | 0.000380023 | Malaria | 2 | 0.219077389 | Arachidonic acid metabolism | 7 | 8.034E-05 |
| Insulin resistance | 28 | 0.000394788 | Ras signaling pathway | 6 | 0.225628743 | Hematopoietic cell lineage | 8 | 0.00010914 |
| Viral carcinogenesis | 45 | 0.000443729 | Longevity regulating pathway | 3 | 0.237667294 | Linoleic acid metabolism | 5 | 0.000113222 |
| Rheumatoid arthritis | 24 | 0.000468522 | Mineral absorption | 2 | 0.238983095 | Thyroid cancer | 5 | 0.000113222 |
| Melanogenesis | 26 | 0.000513869 | Nitrogen metabolism | 1 | 0.264897769 | Carbohydrate digestion and absorption | 6 | 0.000116671 |
| Cholinergic synapse | 28 | 0.000542301 | Non-small cell lung cancer | 2 | 0.265647178 | Ras signaling pathway | 13 | 0.000132687 |
| ECM-receptor interaction | 22 | 0.000855783 | AGE-RAGE signaling pathway in diabetic complications | 3 | 0.271452095 | Asthma | 5 | 0.000134014 |
| Wnt signaling pathway | 33 | 0.000884222 | Starch and sucrose metabolism | 2 | 0.272319313 | Epithelial cell signaling in Helicobacter pylori infection | 7 | 0.00014541 |
| Gap junction | 23 | 0.000977103 | Long-term depression | 2 | 0.292315829 | Galactose metabolism | 5 | 0.000157609 |
| African trypanosomiasis | 12 | 0.000993902 | Focal adhesion | 5 | 0.299659726 | Viral carcinogenesis | 12 | 0.000188958 |
| Epithelial cell signaling in Helicobacter pylori infection | 19 | 0.001112671 | Colorectal cancer | 2 | 0.305610542 | Apoptosis - multiple species | 5 | 0.000214196 |
| TGF-beta signaling pathway | 22 | 0.001211374 | Proteoglycans in cancer | 5 | 0.306675871 | Glutathione metabolism | 6 | 0.000233136 |
| Long-term depression | 17 | 0.001687966 | Synaptic vesicle cycle | 2 | 0.312241463 | AMPK signaling pathway | 9 | 0.000237629 |
| Basal cell carcinoma | 16 | 0.001689594 | Arginine biosynthesis | 1 | 0.316296212 | Cell cycle | 9 | 0.000252547 |
| Phospholipase D signaling pathway | 32 | 0.002028514 | Longevity regulating pathway - multiple species | 2 | 0.31885912 | Chemokine signaling pathway | 11 | 0.00033605 |
| Axon guidance | 37 | 0.003048224 | Retinol metabolism | 2 | 0.325461864 | Jak-STAT signaling pathway | 10 | 0.00035085 |
| GnRH signaling pathway | 22 | 0.003613693 | Terpenoid backbone biosynthesis | 1 | 0.32857713 | Non-small cell lung cancer | 6 | 0.000351574 |
| Regulation of actin cytoskeleton | 43 | 0.003961456 | Long-term potentiation | 2 | 0.332048109 | Arginine biosynthesis | 4 | 0.000382057 |
| Adipocytokine signaling pathway | 18 | 0.004035152 | Pancreatic cancer | 2 | 0.332048109 | Hepatitis C | 9 | 0.000425255 |
| Insulin signaling pathway | 30 | 0.004836708 | Glutamatergic synapse | 3 | 0.335248713 | Viral myocarditis | 6 | 0.000426189 |
| Non-alcoholic fatty liver disease (NAFLD) | 32 | 0.004941193 | Glycolysis / Gluconeogenesis | 2 | 0.33861633 | Tryptophan metabolism | 5 | 0.000539583 |
| Oxytocin signaling pathway | 33 | 0.004970979 | Renal cell carcinoma | 2 | 0.33861633 | TGF-beta signaling pathway | 7 | 0.000541324 |
| Adherens junction | 18 | 0.007516584 | Epithelial cell signaling in Helicobacter pylori infection | 2 | 0.34516506 | Longevity regulating pathway - multiple species | 6 | 0.000725992 |
| Dorso-ventral axis formation | 9 | 0.00845786 | Drug metabolism - cytochrome P450 | 2 | 0.351692891 | Type I diabetes mellitus | 5 | 0.000757647 |
| Shigellosis | 16 | 0.010102639 | Thyroid hormone signaling pathway | 3 | 0.354914048 | GnRH signaling pathway | 7 | 0.000876035 |
| Hippo signaling pathway | 31 | 0.010868638 | alpha-Linolenic acid metabolism | 1 | 0.364118839 | Platelet activation | 8 | 0.001088875 |
| Long-term potentiation | 16 | 0.011726437 | Thyroid hormone synthesis | 2 | 0.364680504 | Type II diabetes mellitus | 5 | 0.001258845 |
| Carbohydrate digestion and absorption | 12 | 0.015382595 | Sphingolipid signaling pathway | 3 | 0.364722986 | Inflammatory mediator regulation of TRP channels | 7 | 0.001356194 |
| Salmonella infection | 19 | 0.017356337 | PPAR signaling pathway | 2 | 0.371137744 | Arginine and proline metabolism | 5 | 0.001382917 |
| Fc gamma R-mediated phagocytosis | 20 | 0.017691307 | Prolactin signaling pathway | 2 | 0.371137744 | Cocaine addiction | 5 | 0.001382917 |
| Caffeine metabolism | 3 | 0.019324101 | Metabolism of xenobiotics by cytochrome P450 | 2 | 0.377568999 | Ovarian steroidogenesis | 5 | 0.001515781 |
| Allograft rejection | 10 | 0.020304091 | Purine metabolism | 4 | 0.379860501 | Metabolic pathways | 35 | 0.001559485 |
| Phototransduction | 8 | 0.021229127 | Phototransduction | 1 | 0.386767251 | Choline metabolism in cancer | 7 | 0.001616115 |
| cAMP signaling pathway | 37 | 0.022074343 | Platinum drug resistance | 2 | 0.390349035 | Autoimmune thyroid disease | 5 | 0.001809367 |
| Cytokine-cytokine receptor interaction | 47 | 0.024490432 | Tuberculosis | 4 | 0.392083005 | Natural killer cell mediated cytotoxicity | 8 | 0.001985162 |
| Pathogenic Escherichia coli infection | 13 | 0.02666695 | Dorso-ventral axis formation | 1 | 0.397788731 | cAMP signaling pathway | 10 | 0.002066494 |
| Bacterial invasion of epithelial cells | 17 | 0.026694425 | Alcoholism | 4 | 0.400213475 | Cholinergic synapse | 7 | 0.002771391 |
| Regulation of lipolysis in adipocytes | 13 | 0.030625007 | Transcriptional misregulation in cancer | 4 | 0.400213475 | Tyrosine metabolism | 4 | 0.002778596 |
| AMPK signaling pathway | 24 | 0.034725829 | Thyroid cancer | 1 | 0.40861317 | Long-term depression | 5 | 0.003410532 |
| Serotonergic synapse | 22 | 0.03921769 | PI3K-Akt signaling pathway | 7 | 0.409255024 | Cytosolic DNA-sensing pathway | 5 | 0.004214297 |
| Hypertrophic cardiomyopathy (HCM) | 17 | 0.045792443 | Osteoclast differentiation | 3 | 0.418112516 | Vascular smooth muscle contraction | 7 | 0.004276479 |
| Type I diabetes mellitus | 10 | 0.053473383 | EGFR tyrosine kinase inhibitor resistance | 2 | 0.427956421 | Fc gamma R-mediated phagocytosis | 6 | 0.004678771 |
| Platelet activation | 23 | 0.053555652 | ECM-receptor interaction | 2 | 0.434107988 | Retinol metabolism | 5 | 0.004820014 |
| Arrhythmogenic right ventricular cardiomyopathy (ARVC) | 15 | 0.062807761 | Fructose and mannose metabolism | 1 | 0.450009679 | Amphetamine addiction | 5 | 0.005485146 |
| RIG-I-like receptor signaling pathway | 14 | 0.077377218 | Apoptosis - multiple species | 1 | 0.450009679 | Huntington's disease | 9 | 0.005584514 |
| Autoimmune thyroid disease | 11 | 0.079342842 | Salmonella infection | 2 | 0.458353635 | Dopaminergic synapse | 7 | 0.006331911 |
| Graft-versus-host disease | 9 | 0.088217188 | Apoptosis | 3 | 0.460698616 | Progesterone-mediated oocyte maturation | 6 | 0.006356778 |
| Intestinal immune network for IgA production | 10 | 0.088831703 | Hematopoietic cell lineage | 2 | 0.464321602 | Renin-angiotensin system | 3 | 0.006619745 |
| Inflammatory mediator regulation of TRP channels | 18 | 0.098175897 | Parkinson's disease | 3 | 0.469986736 | Bile secretion | 5 | 0.0070055 |
| Notch signaling pathway | 10 | 0.099446059 | ErbB signaling pathway | 2 | 0.470250997 | PPAR signaling pathway | 5 | 0.00742731 |
| Galactose metabolism | 7 | 0.11060171 | Phospholipase D signaling pathway | 3 | 0.474604223 | Adherens junction | 5 | 0.008323479 |
| Arginine biosynthesis | 5 | 0.139179119 | Rheumatoid arthritis | 2 | 0.47614128 | Wnt signaling pathway | 7 | 0.010495939 |
| Viral myocarditis | 11 | 0.144367669 | Hepatitis B | 3 | 0.488345641 | Cell adhesion molecules (CAMs) | 7 | 0.010882919 |
| Herpes simplex infection | 30 | 0.149398827 | NF-kappa B signaling pathway | 2 | 0.493572532 | ECM-receptor interaction | 5 | 0.012658403 |
| Tight junction | 23 | 0.162143988 | HTLV-I infection | 5 | 0.49392614 | Peroxisome | 5 | 0.01328999 |
| Staphylococcus aureus infection | 10 | 0.192706344 | Endocytosis | 5 | 0.500755927 | Hypertrophic cardiomyopathy (HCM) | 5 | 0.01328999 |
| Primary immunodeficiency | 7 | 0.197990181 | Non-alcoholic fatty liver disease (NAFLD) | 3 | 0.510855994 | Leukocyte transendothelial migration | 6 | 0.015108679 |
| Cocaine addiction | 9 | 0.200849603 | Tryptophan metabolism | 1 | 0.515636524 | mTOR signaling pathway | 7 | 0.015342133 |
| Dilated cardiomyopathy | 15 | 0.20393083 | Pyruvate metabolism | 1 | 0.515636524 | Acute myeloid leukemia | 4 | 0.015725262 |
| Asthma | 6 | 0.204402813 | Hippo signaling pathway | 3 | 0.519715141 | Gap junction | 5 | 0.016767911 |
| cGMP-PKG signaling pathway | 26 | 0.229337291 | Fat digestion and absorption | 1 | 0.524352817 | Renin secretion | 4 | 0.023101372 |
| Glutathione metabolism | 9 | 0.253829658 | Progesterone-mediated oocyte maturation | 2 | 0.527319646 | Shigellosis | 4 | 0.02430015 |
| Drug metabolism - other enzymes | 8 | 0.267283674 | Amoebiasis | 2 | 0.532795617 | Melanogenesis | 5 | 0.027446812 |
| Cell adhesion molecules (CAMs) | 22 | 0.271228069 | Type I diabetes mellitus | 1 | 0.541320108 | Tight junction | 6 | 0.030895559 |
| Glycosaminoglycan biosynthesis - chondroitin sulfate / dermatan sulfate | 4 | 0.274201057 | Choline metabolism in cancer | 2 | 0.543617843 | Fat digestion and absorption | 3 | 0.031934121 |
| Circadian entrainment | 15 | 0.284071554 | Ether lipid metabolism | 1 | 0.55768528 | Phenylalanine metabolism | 2 | 0.03292442 |
| Antigen processing and presentation | 12 | 0.330245999 | Drug metabolism - other enzymes | 1 | 0.565648743 | Signaling pathways regulating pluripotency of stem cells | 6 | 0.033792095 |
| Pentose phosphate pathway | 5 | 0.344846549 | Hedgehog signaling pathway | 1 | 0.565648743 | 2-Oxocarboxylic acid metabolism | 2 | 0.03663429 |
| Calcium signaling pathway | 26 | 0.355262482 | Sphingolipid metabolism | 1 | 0.573469588 | Drug metabolism - other enzymes | 3 | 0.042789651 |
| Complement and coagulation cascades | 12 | 0.364032101 | Endocrine and other factor-regulated calcium reabsorption | 1 | 0.573469588 | Histidine metabolism | 2 | 0.061880316 |
| Retinol metabolism | 10 | 0.368200862 | Arginine and proline metabolism | 1 | 0.588693544 | cGMP-PKG signaling pathway | 6 | 0.064773016 |
| Renin-angiotensin system | 4 | 0.370303777 | TNF signaling pathway | 2 | 0.590144296 | Riboflavin metabolism | 1 | 0.066151339 |
| ABC transporters | 7 | 0.372908626 | Vibrio cholerae infection | 1 | 0.603376935 | Staphylococcus aureus infection | 3 | 0.066325268 |
| Metabolism of xenobiotics by cytochrome P450 | 11 | 0.384095959 | Carbon metabolism | 2 | 0.604854543 | Protein digestion and absorption | 4 | 0.066568748 |
| Amphetamine addiction | 10 | 0.406075411 | Endometrial cancer | 1 | 0.610521912 | Regulation of lipolysis in adipocytes | 3 | 0.069241065 |
| Valine, leucine and isoleucine biosynthesis | 1 | 0.436768533 | MicroRNAs in cancer | 5 | 0.625427536 | Starch and sucrose metabolism | 3 | 0.072214165 |
| D-Glutamine and D-glutamate metabolism | 1 | 0.436768533 | Legionellosis | 1 | 0.631197729 | Regulation of actin cytoskeleton | 7 | 0.073397485 |
| Riboflavin metabolism | 1 | 0.436768533 | Neurotrophin signaling pathway | 2 | 0.637622956 | Endocytosis | 8 | 0.075076581 |
| Fructose and mannose metabolism | 5 | 0.456609088 | Viral myocarditis | 1 | 0.650781539 | Systemic lupus erythematosus | 5 | 0.077633587 |
| Base excision repair | 5 | 0.456609088 | Lysosome | 2 | 0.651004013 | Circadian entrainment | 4 | 0.077840554 |
| Dopaminergic synapse | 18 | 0.461101536 | VEGF signaling pathway | 1 | 0.669330721 | Glycerolipid metabolism | 3 | 0.078328865 |
| Maturity onset diabetes of the young | 4 | 0.465486247 | Inflammatory bowel disease (IBD) | 1 | 0.692547121 | Insulin signaling pathway | 5 | 0.087571235 |
| Bile secretion | 10 | 0.481685414 | Hepatitis C | 2 | 0.692790593 | Fructose and mannose metabolism | 2 | 0.107378659 |
| Phagosome | 21 | 0.48404918 | p53 signaling pathway | 1 | 0.714141638 | RIG-I-like receptor signaling pathway | 3 | 0.115670313 |
| Retrograde endocannabinoid signaling | 14 | 0.485953776 | B cell receptor signaling pathway | 1 | 0.72934128 | Alanine, aspartate and glutamate metabolism | 2 | 0.118431001 |
| Steroid biosynthesis | 3 | 0.511924143 | Cell adhesion molecules (CAMs) | 2 | 0.730386748 | Phagosome | 5 | 0.118706602 |
| Pentose and glucuronate interconversions | 5 | 0.536842208 | Chronic myeloid leukemia | 1 | 0.734227006 | Hippo signaling pathway | 5 | 0.118706602 |
| Chemical carcinogenesis | 11 | 0.544243892 | Phagosome | 2 | 0.764027387 | Inositol phosphate metabolism | 3 | 0.119349416 |
| Mineral absorption | 7 | 0.553623919 | mTOR signaling pathway | 2 | 0.764027387 | Primary immunodeficiency | 2 | 0.124060021 |
| Nicotinate and nicotinamide metabolism | 4 | 0.554994246 | Peroxisome | 1 | 0.77850881 | Carbon metabolism | 4 | 0.125519896 |
| Glutamatergic synapse | 15 | 0.568334005 | Protein digestion and absorption | 1 | 0.805059447 | Biosynthesis of amino acids | 3 | 0.134486503 |
| Proximal tubule bicarbonate reclamation | 3 | 0.610439808 | Glycerophospholipid metabolism | 1 | 0.822062009 | Aldosterone-regulated sodium reabsorption | 2 | 0.141312627 |
| Adrenergic signaling in cardiomyocytes | 19 | 0.612442185 | Influenza A | 2 | 0.825481291 | Bacterial invasion of epithelial cells | 3 | 0.146252412 |
| Thyroid hormone synthesis | 9 | 0.62101774 | Phosphatidylinositol signaling system | 1 | 0.831546809 | Glycine, serine and threonine metabolism | 2 | 0.147173243 |
| Nicotine addiction | 5 | 0.634341435 | Melanogenesis | 1 | 0.837588896 | Protein processing in endoplasmic reticulum | 5 | 0.148994209 |
| Glycosaminoglycan biosynthesis - heparan sulfate / heparin | 3 | 0.640159007 | T cell receptor signaling pathway | 1 | 0.851767553 | Oocyte meiosis | 4 | 0.156256325 |
| Arginine and proline metabolism | 6 | 0.65480857 | Toll-like receptor signaling pathway | 1 | 0.854451929 | Sulfur metabolism | 1 | 0.157306649 |
| Gastric acid secretion | 9 | 0.671412934 | Epstein-Barr virus infection | 2 | 0.87762541 | Proteasome | 2 | 0.171073786 |
| Protein digestion and absorption | 11 | 0.672263394 | Leukocyte transendothelial migration | 1 | 0.883129836 | Taurine and hypotaurine metabolism | 1 | 0.171614389 |
| Steroid hormone biosynthesis | 7 | 0.672599472 | Toxoplasmosis | 1 | 0.883129836 | Insulin secretion | 3 | 0.174916097 |
| Phosphatidylinositol signaling system | 12 | 0.673134028 | Viral carcinogenesis | 2 | 0.886165741 | Cysteine and methionine metabolism | 2 | 0.177147545 |
| Ovarian steroidogenesis | 6 | 0.674553637 | Cell cycle | 1 | 0.89528472 | Hedgehog signaling pathway | 2 | 0.183254722 |
| Biosynthesis of amino acids | 9 | 0.687290896 | Oxidative phosphorylation | 1 | 0.911199212 | GABAergic synapse | 3 | 0.187653127 |
| Tyrosine metabolism | 4 | 0.706478243 | Natural killer cell mediated cytotoxicity | 1 | 0.912811723 | Calcium signaling pathway | 5 | 0.187984367 |
| Vascular smooth muscle contraction | 14 | 0.747952212 | Wnt signaling pathway | 1 | 0.924706052 | Alcoholism | 5 | 0.187984367 |
| Inositol phosphate metabolism | 8 | 0.749870913 | Signaling pathways regulating pluripotency of stem cells | 1 | 0.924706052 | Endocrine and other factor-regulated calcium reabsorption | 2 | 0.189392458 |
| Hedgehog signaling pathway | 5 | 0.754536406 | Olfactory transduction | 4 | 0.944280473 | Pancreatic secretion | 3 | 0.222684861 |
| Cytosolic DNA-sensing pathway | 7 | 0.755083238 | Herpes simplex infection | 1 | 0.965839838 | Phospholipase D signaling pathway | 4 | 0.224725815 |
| GABAergic synapse | 10 | 0.755784636 | Huntington's disease | 1 | 0.970521861 | Phosphatidylinositol signaling system | 3 | 0.231643692 |
| Sulfur metabolism | 1 | 0.762029371 | Cytokine-cytokine receptor interaction | 1 | 0.99221916 | Pathogenic Escherichia coli infection | 2 | 0.239278959 |
| One carbon pool by folate | 2 | 0.768584977 | Metabolic pathways | 12 | 0.995746547 | Basal cell carcinoma | 2 | 0.239278959 |
| Nucleotide excision repair | 5 | 0.771365042 | Citrate cycle (TCA cycle) | 0 | 1 | Retrograde endocannabinoid signaling | 3 | 0.245202597 |
| Oocyte meiosis | 14 | 0.779900048 | Pentose phosphate pathway | 0 | 1 | Glucagon signaling pathway | 3 | 0.245202597 |
| Circadian rhythm | 3 | 0.784548987 | Pentose and glucuronate interconversions | 0 | 1 | Other glycan degradation | 1 | 0.265227097 |
| Amino sugar and nucleotide sugar metabolism | 5 | 0.787298038 | Ascorbate and aldarate metabolism | 0 | 1 | Long-term potentiation | 2 | 0.308593451 |
| Protein processing in endoplasmic reticulum | 19 | 0.791396973 | Fatty acid biosynthesis | 0 | 1 | Terpenoid backbone biosynthesis | 1 | 0.313915629 |
| Ubiquinone and other terpenoid-quinone biosynthesis | 1 | 0.793870512 | Fatty acid elongation | 0 | 1 | Glycosaminoglycan biosynthesis - heparan sulfate / heparin | 1 | 0.337042981 |
| Taurine and hypotaurine metabolism | 1 | 0.793870512 | Fatty acid degradation | 0 | 1 | Vitamin digestion and absorption | 1 | 0.337042981 |
| Vibrio cholerae infection | 5 | 0.829922703 | Synthesis and degradation of ketone bodies | 0 | 1 | Thyroid hormone synthesis | 2 | 0.339792876 |
| Mismatch repair | 2 | 0.832593502 | Steroid biosynthesis | 0 | 1 | alpha-Linolenic acid metabolism | 1 | 0.348314275 |
| Non-homologous end-joining | 1 | 0.845348463 | Primary bile acid biosynthesis | 0 | 1 | Ascorbate and aldarate metabolism | 1 | 0.37028839 |
| Arachidonic acid metabolism | 6 | 0.853427605 | Ubiquinone and other terpenoid-quinone biosynthesis | 0 | 1 | Antigen processing and presentation | 2 | 0.376640582 |
| Lysosome | 13 | 0.85450546 | Pyrimidine metabolism | 0 | 1 | Glyoxylate and dicarboxylate metabolism | 1 | 0.380997564 |
| PPAR signaling pathway | 7 | 0.863767472 | Alanine, aspartate and glutamate metabolism | 0 | 1 | Dorso-ventral axis formation | 1 | 0.380997564 |
| Folate biosynthesis | 1 | 0.866047265 | Glycine, serine and threonine metabolism | 0 | 1 | Complement and coagulation cascades | 2 | 0.388741242 |
| Cysteine and methionine metabolism | 4 | 0.869303795 | Cysteine and methionine metabolism | 0 | 1 | Pentose phosphate pathway | 1 | 0.39152563 |
| Huntington's disease | 21 | 0.872914963 | Valine, leucine and isoleucine degradation | 0 | 1 | Aldosterone synthesis and secretion | 2 | 0.400738228 |
| Renin secretion | 6 | 0.873518369 | Valine, leucine and isoleucine biosynthesis | 0 | 1 | Circadian rhythm | 1 | 0.401875634 |
| Insulin secretion | 8 | 0.896713783 | Lysine biosynthesis | 0 | 1 | Parkinson's disease | 3 | 0.434165293 |
| Carbon metabolism | 11 | 0.904381268 | Lysine degradation | 0 | 1 | Salivary secretion | 2 | 0.447563312 |
| Glyoxylate and dicarboxylate metabolism | 2 | 0.904580185 | Histidine metabolism | 0 | 1 | Dilated cardiomyopathy | 2 | 0.447563312 |
| RNA degradation | 7 | 0.904687372 | Tyrosine metabolism | 0 | 1 | Pentose and glucuronate interconversions | 1 | 0.460400588 |
| Primary bile acid biosynthesis | 1 | 0.91296283 | Phenylalanine metabolism | 0 | 1 | Adrenergic signaling in cardiomyocytes | 3 | 0.460885858 |
| Phenylalanine metabolism | 1 | 0.91296283 | Phenylalanine, tyrosine and tryptophan biosynthesis | 0 | 1 | Glycerophospholipid metabolism | 2 | 0.481317168 |
| Nitrogen metabolism | 1 | 0.91296283 | beta-Alanine metabolism | 0 | 1 | Regulation of autophagy | 1 | 0.496216925 |
| Drug metabolism - cytochrome P450 | 6 | 0.913860452 | Taurine and hypotaurine metabolism | 0 | 1 | Porphyrin and chlorophyll metabolism | 1 | 0.513228369 |
| Linoleic acid metabolism | 2 | 0.914964316 | Selenocompound metabolism | 0 | 1 | ABC transporters | 1 | 0.529668535 |
| Homologous recombination | 2 | 0.914964316 | D-Glutamine and D-glutamate metabolism | 0 | 1 | Vasopressin-regulated water reabsorption | 1 | 0.529668535 |
| Tryptophan metabolism | 3 | 0.917629084 | D-Arginine and D-ornithine metabolism | 0 | 1 | Ether lipid metabolism | 1 | 0.537680375 |
| Regulation of autophagy | 3 | 0.917629084 | Glutathione metabolism | 0 | 1 | Sphingolipid metabolism | 1 | 0.553299198 |
| 2-Oxocarboxylic acid metabolism | 1 | 0.924616518 | N-Glycan biosynthesis | 0 | 1 | Axon guidance | 3 | 0.577300281 |
| Alcoholism | 18 | 0.927347196 | Other glycan degradation | 0 | 1 | Glutamatergic synapse | 2 | 0.579411689 |
| Glycosaminoglycan degradation | 1 | 0.934710831 | Mucin type O-Glycan biosynthesis | 0 | 1 | Mineral absorption | 1 | 0.59008861 |
| Endocytosis | 27 | 0.940211669 | Other types of O-glycan biosynthesis | 0 | 1 | Glycolysis / Gluconeogenesis | 1 | 0.683334866 |
| Fanconi anemia pathway | 4 | 0.94243355 | Glycosaminoglycan degradation | 0 | 1 | Neuroactive ligand-receptor interaction | 4 | 0.695979619 |
| Pyrimidine metabolism | 9 | 0.94834968 | Glycosaminoglycan biosynthesis - chondroitin sulfate / dermatan sulfate | 0 | 1 | Gastric acid secretion | 1 | 0.719304535 |
| Glycolysis / Gluconeogenesis | 5 | 0.955450496 | Glycosaminoglycan biosynthesis - keratan sulfate | 0 | 1 | RNA degradation | 1 | 0.733447763 |
| Terpenoid backbone biosynthesis | 1 | 0.957587186 | Glycosaminoglycan biosynthesis - heparan sulfate / heparin | 0 | 1 | Ribosome biogenesis in eukaryotes | 1 | 0.759643206 |
| Alanine, aspartate and glutamate metabolism | 2 | 0.958062749 | Glycerolipid metabolism | 0 | 1 | Taste transduction | 1 | 0.759643206 |
| Cardiac muscle contraction | 6 | 0.958953952 | Inositol phosphate metabolism | 0 | 1 | Morphine addiction | 1 | 0.790630191 |
| Endocrine and other factor-regulated calcium reabsorption | 3 | 0.960286562 | Glycosylphosphatidylinositol(GPI)-anchor biosynthesis | 0 | 1 | Purine metabolism | 2 | 0.798570685 |
| Ubiquitin mediated proteolysis | 12 | 0.963767338 | Glycosphingolipid biosynthesis - lacto and neolacto series | 0 | 1 | Citrate cycle (TCA cycle) | 0 | 1 |
| Glycerolipid metabolism | 4 | 0.964534907 | Glycosphingolipid biosynthesis - globo series | 0 | 1 | Fatty acid biosynthesis | 0 | 1 |
| Purine metabolism | 16 | 0.964801413 | Glycosphingolipid biosynthesis - ganglio series | 0 | 1 | Fatty acid elongation | 0 | 1 |
| Histidine metabolism | 1 | 0.968189638 | Glyoxylate and dicarboxylate metabolism | 0 | 1 | Fatty acid degradation | 0 | 1 |
| Vitamin digestion and absorption | 1 | 0.968189638 | Propanoate metabolism | 0 | 1 | Synthesis and degradation of ketone bodies | 0 | 1 |
| Morphine addiction | 7 | 0.968258028 | Butanoate metabolism | 0 | 1 | Steroid biosynthesis | 0 | 1 |
| Aldosterone synthesis and secretion | 6 | 0.968341618 | One carbon pool by folate | 0 | 1 | Primary bile acid biosynthesis | 0 | 1 |
| Parkinson's disease | 12 | 0.974374992 | Thiamine metabolism | 0 | 1 | Ubiquinone and other terpenoid-quinone biosynthesis | 0 | 1 |
| Glycosphingolipid biosynthesis - lacto and neolacto series | 1 | 0.97614309 | Riboflavin metabolism | 0 | 1 | Oxidative phosphorylation | 0 | 1 |
| Lysine degradation | 3 | 0.976914046 | Nicotinate and nicotinamide metabolism | 0 | 1 | Pyrimidine metabolism | 0 | 1 |
| Glycine, serine and threonine metabolism | 2 | 0.977119238 | Pantothenate and CoA biosynthesis | 0 | 1 | Valine, leucine and isoleucine degradation | 0 | 1 |
| Pancreatic secretion | 7 | 0.979211792 | Biotin metabolism | 0 | 1 | Valine, leucine and isoleucine biosynthesis | 0 | 1 |
| Ascorbate and aldarate metabolism | 1 | 0.979340147 | Lipoic acid metabolism | 0 | 1 | Lysine biosynthesis | 0 | 1 |
| Collecting duct acid secretion | 1 | 0.979340147 | Folate biosynthesis | 0 | 1 | Lysine degradation | 0 | 1 |
| Fat digestion and absorption | 2 | 0.979761218 | Porphyrin and chlorophyll metabolism | 0 | 1 | Phenylalanine, tyrosine and tryptophan biosynthesis | 0 | 1 |
| Porphyrin and chlorophyll metabolism | 2 | 0.982106343 | Sulfur metabolism | 0 | 1 | beta-Alanine metabolism | 0 | 1 |
| Hippo signaling pathway -multiple species | 1 | 0.982109034 | Aminoacyl-tRNA biosynthesis | 0 | 1 | Selenocompound metabolism | 0 | 1 |
| Salivary secretion | 6 | 0.98458067 | Biosynthesis of unsaturated fatty acids | 0 | 1 | D-Glutamine and D-glutamate metabolism | 0 | 1 |
| Glucagon signaling pathway | 7 | 0.986573884 | 2-Oxocarboxylic acid metabolism | 0 | 1 | D-Arginine and D-ornithine metabolism | 0 | 1 |
| Starch and sucrose metabolism | 3 | 0.986775217 | Fatty acid metabolism | 0 | 1 | N-Glycan biosynthesis | 0 | 1 |
| Other types of O-glycan biosynthesis | 1 | 0.988382452 | Biosynthesis of amino acids | 0 | 1 | Mucin type O-Glycan biosynthesis | 0 | 1 |
| RNA polymerase | 1 | 0.988382452 | ABC transporters | 0 | 1 | Other types of O-glycan biosynthesis | 0 | 1 |
| Peroxisome | 5 | 0.990303666 | Ribosome biogenesis in eukaryotes | 0 | 1 | Amino sugar and nucleotide sugar metabolism | 0 | 1 |
| Sphingolipid metabolism | 2 | 0.990393944 | Ribosome | 0 | 1 | Butirosin and neomycin biosynthesis | 0 | 1 |
| N-Glycan biosynthesis | 2 | 0.992529809 | RNA transport | 0 | 1 | Glycosaminoglycan degradation | 0 | 1 |
| Synaptic vesicle cycle | 3 | 0.993333425 | mRNA surveillance pathway | 0 | 1 | Glycosaminoglycan biosynthesis - chondroitin sulfate / dermatan sulfate | 0 | 1 |
| DNA replication | 1 | 0.994344744 | RNA degradation | 0 | 1 | Glycosaminoglycan biosynthesis - keratan sulfate | 0 | 1 |
| Ribosome biogenesis in eukaryotes | 4 | 0.997173032 | RNA polymerase | 0 | 1 | Glycosylphosphatidylinositol(GPI)-anchor biosynthesis | 0 | 1 |
| Taste transduction | 4 | 0.997173032 | Basal transcription factors | 0 | 1 | Glycosphingolipid biosynthesis - lacto and neolacto series | 0 | 1 |
| Proteasome | 1 | 0.998214112 | DNA replication | 0 | 1 | Glycosphingolipid biosynthesis - globo series | 0 | 1 |
| Vasopressin-regulated water reabsorption | 1 | 0.998214112 | Spliceosome | 0 | 1 | Glycosphingolipid biosynthesis - ganglio series | 0 | 1 |
| Systemic lupus erythematosus | 8 | 0.998270305 | Proteasome | 0 | 1 | Pyruvate metabolism | 0 | 1 |
| Ether lipid metabolism | 1 | 0.998453855 | Protein export | 0 | 1 | Propanoate metabolism | 0 | 1 |
| Basal transcription factors | 1 | 0.998453855 | Base excision repair | 0 | 1 | Butanoate metabolism | 0 | 1 |
| Valine, leucine and isoleucine degradation | 1 | 0.998996773 | Nucleotide excision repair | 0 | 1 | One carbon pool by folate | 0 | 1 |
| Neuroactive ligand-receptor interaction | 21 | 0.999221647 | Mismatch repair | 0 | 1 | Thiamine metabolism | 0 | 1 |
| Glycerophospholipid metabolism | 3 | 0.999860321 | Homologous recombination | 0 | 1 | Vitamin B6 metabolism | 0 | 1 |
| Aminoacyl-tRNA biosynthesis | 1 | 0.999925345 | Non-homologous end-joining | 0 | 1 | Nicotinate and nicotinamide metabolism | 0 | 1 |
| mRNA surveillance pathway | 1 | 0.999997994 | Fanconi anemia pathway | 0 | 1 | Pantothenate and CoA biosynthesis | 0 | 1 |
| Oxidative phosphorylation | 3 | 0.999998931 | Chemokine signaling pathway | 0 | 1 | Biotin metabolism | 0 | 1 |
| Spliceosome | 3 | 0.999999062 | Ubiquitin mediated proteolysis | 0 | 1 | Lipoic acid metabolism | 0 | 1 |
| RNA transport | 3 | 0.999999993 | Sulfur relay system | 0 | 1 | Folate biosynthesis | 0 | 1 |
| Ribosome | 1 | 0.999999997 | SNARE interactions in vesicular transport | 0 | 1 | Nitrogen metabolism | 0 | 1 |
| Olfactory transduction | 6 | 1 | Regulation of autophagy | 0 | 1 | Aminoacyl-tRNA biosynthesis | 0 | 1 |
| Citrate cycle (TCA cycle) | 0 | 1 | Protein processing in endoplasmic reticulum | 0 | 1 | Biosynthesis of unsaturated fatty acids | 0 | 1 |
| Fatty acid biosynthesis | 0 | 1 | Notch signaling pathway | 0 | 1 | Fatty acid metabolism | 0 | 1 |
| Fatty acid elongation | 0 | 1 | TGF-beta signaling pathway | 0 | 1 | Ribosome | 0 | 1 |
| Fatty acid degradation | 0 | 1 | Axon guidance | 0 | 1 | RNA transport | 0 | 1 |
| Synthesis and degradation of ketone bodies | 0 | 1 | Hippo signaling pathway -multiple species | 0 | 1 | mRNA surveillance pathway | 0 | 1 |
| Lysine biosynthesis | 0 | 1 | Tight junction | 0 | 1 | RNA polymerase | 0 | 1 |
| Phenylalanine, tyrosine and tryptophan biosynthesis | 0 | 1 | Antigen processing and presentation | 0 | 1 | Basal transcription factors | 0 | 1 |
| beta-Alanine metabolism | 0 | 1 | NOD-like receptor signaling pathway | 0 | 1 | DNA replication | 0 | 1 |
| Selenocompound metabolism | 0 | 1 | RIG-I-like receptor signaling pathway | 0 | 1 | Spliceosome | 0 | 1 |
| D-Arginine and D-ornithine metabolism | 0 | 1 | Cytosolic DNA-sensing pathway | 0 | 1 | Protein export | 0 | 1 |
| Other glycan degradation | 0 | 1 | Jak-STAT signaling pathway | 0 | 1 | Base excision repair | 0 | 1 |
| Mucin type O-Glycan biosynthesis | 0 | 1 | Fc epsilon RI signaling pathway | 0 | 1 | Nucleotide excision repair | 0 | 1 |
| Butirosin and neomycin biosynthesis | 0 | 1 | Fc gamma R-mediated phagocytosis | 0 | 1 | Mismatch repair | 0 | 1 |
| Glycosaminoglycan biosynthesis - keratan sulfate | 0 | 1 | Intestinal immune network for IgA production | 0 | 1 | Homologous recombination | 0 | 1 |
| Glycosylphosphatidylinositol(GPI)-anchor biosynthesis | 0 | 1 | Circadian rhythm | 0 | 1 | Non-homologous end-joining | 0 | 1 |
| alpha-Linolenic acid metabolism | 0 | 1 | Vasopressin-regulated water reabsorption | 0 | 1 | Fanconi anemia pathway | 0 | 1 |
| Glycosphingolipid biosynthesis - globo series | 0 | 1 | Vitamin digestion and absorption | 0 | 1 | Ubiquitin mediated proteolysis | 0 | 1 |
| Glycosphingolipid biosynthesis - ganglio series | 0 | 1 | Amyotrophic lateral sclerosis (ALS) | 0 | 1 | Sulfur relay system | 0 | 1 |
| Pyruvate metabolism | 0 | 1 | Prion diseases | 0 | 1 | SNARE interactions in vesicular transport | 0 | 1 |
| Propanoate metabolism | 0 | 1 | Bacterial invasion of epithelial cells | 0 | 1 | Lysosome | 0 | 1 |
| Butanoate metabolism | 0 | 1 | Pathogenic Escherichia coli infection | 0 | 1 | Cardiac muscle contraction | 0 | 1 |
| Thiamine metabolism | 0 | 1 | Shigellosis | 0 | 1 | Notch signaling pathway | 0 | 1 |
| Vitamin B6 metabolism | 0 | 1 | African trypanosomiasis | 0 | 1 | Hippo signaling pathway -multiple species | 0 | 1 |
| Pantothenate and CoA biosynthesis | 0 | 1 | Measles | 0 | 1 | Synaptic vesicle cycle | 0 | 1 |
| Biotin metabolism | 0 | 1 | Basal cell carcinoma | 0 | 1 | Olfactory transduction | 0 | 1 |
| Lipoic acid metabolism | 0 | 1 | Acute myeloid leukemia | 0 | 1 | Phototransduction | 0 | 1 |
| Biosynthesis of unsaturated fatty acids | 0 | 1 | Asthma | 0 | 1 | Maturity onset diabetes of the young | 0 | 1 |
| Metabolic pathways | 88 | 1 | Autoimmune thyroid disease | 0 | 1 | Proximal tubule bicarbonate reclamation | 0 | 1 |
| Fatty acid metabolism | 0 | 1 | Systemic lupus erythematosus | 0 | 1 | Collecting duct acid secretion | 0 | 1 |
| Protein export | 0 | 1 | Allograft rejection | 0 | 1 | Nicotine addiction | 0 | 1 |
| Sulfur relay system | 0 | 1 | Graft-versus-host disease | 0 | 1 | Vibrio cholerae infection | 0 | 1 |
| SNARE interactions in vesicular transport | 0 | 1 | Primary immunodeficiency | 0 | 1 | Arrhythmogenic right ventricular cardiomyopathy (ARVC) | 0 | 1 |

Pathways in M4

| **symptom** | **overlap** | **p_value** | **drug** | **overlap** | **p_value** | **herb** | **overlap** | **p_value** |
| --- | --- | --- | --- | --- | --- | --- | --- | --- |
| AGE-RAGE signaling pathway in diabetic complications | 33 | 9.50162E-12 | Aldosterone synthesis and secretion | 6 | 9.26967E-09 | Pathways in cancer | 75 | 4.58757E-36 |
| HIF-1 signaling pathway | 31 | 4.21666E-10 | Adrenergic signaling in cardiomyocytes | 7 | 1.02503E-08 | AGE-RAGE signaling pathway in diabetic complications | 38 | 2.65306E-30 |
| Inflammatory bowel disease (IBD) | 23 | 2.31694E-09 | GABAergic synapse | 6 | 1.53444E-08 | Hepatitis B | 44 | 3.24E-30 |
| ECM-receptor interaction | 26 | 3.1633E-09 | Oxytocin signaling pathway | 7 | 1.54586E-08 | FoxO signaling pathway | 41 | 1.23074E-28 |
| Hypertrophic cardiomyopathy (HCM) | 26 | 4.22897E-09 | Retrograde endocannabinoid signaling | 6 | 3.53069E-08 | Endocrine resistance | 34 | 5.36995E-26 |
| Malaria | 19 | 1.00313E-08 | Vascular smooth muscle contraction | 6 | 9.93773E-08 | PI3K-Akt signaling pathway | 56 | 1.05321E-23 |
| Hematopoietic cell lineage | 26 | 1.28169E-08 | Renin secretion | 5 | 1.43614E-07 | Prostate cancer | 31 | 1.08557E-23 |
| Amoebiasis | 28 | 1.37578E-08 | Arrhythmogenic right ventricular cardiomyopathy (ARVC) | 5 | 2.99533E-07 | Tuberculosis | 40 | 2.498E-22 |
| Focal adhesion | 42 | 9.89381E-08 | Cardiac muscle contraction | 5 | 3.90527E-07 | Apoptosis | 36 | 3.14326E-22 |
| Proteoglycans in cancer | 41 | 3.68258E-07 | MAPK signaling pathway | 7 | 4.32024E-07 | Pancreatic cancer | 26 | 1.19935E-21 |
| African trypanosomiasis | 14 | 3.72848E-07 | Hypertrophic cardiomyopathy (HCM) | 5 | 5.33643E-07 | Platinum drug resistance | 26 | 5.55363E-20 |
| PI3K-Akt signaling pathway | 58 | 4.96554E-07 | Dilated cardiomyopathy | 5 | 7.57009E-07 | Proteoglycans in cancer | 40 | 7.68452E-20 |
| Toxoplasmosis | 28 | 7.79518E-07 | GnRH signaling pathway | 5 | 8.45953E-07 | Toxoplasmosis | 31 | 1.41208E-19 |
| Viral myocarditis | 18 | 1.20356E-06 | Calcium signaling pathway | 6 | 1.06079E-06 | TNF signaling pathway | 30 | 1.73266E-19 |
| Dilated cardiomyopathy | 23 | 1.55247E-06 | Amphetamine addiction | 4 | 8.74944E-06 | Leishmaniasis | 25 | 2.81661E-19 |
| Legionellosis | 17 | 2.53195E-06 | cGMP-PKG signaling pathway | 5 | 1.68001E-05 | Chagas disease (American trypanosomiasis) | 29 | 3.61685E-19 |
| Amyotrophic lateral sclerosis (ALS) | 16 | 4.01189E-06 | Circadian entrainment | 4 | 3.50272E-05 | Colorectal cancer | 23 | 1.39632E-18 |
| Rheumatoid arthritis | 22 | 5.76369E-06 | cAMP signaling pathway | 5 | 3.91045E-05 | Bladder cancer | 19 | 9.21029E-18 |
| Cytokine-cytokine receptor interaction | 45 | 1.07882E-05 | Cholinergic synapse | 4 | 6.45223E-05 | HTLV-I infection | 42 | 1.00971E-17 |
| Asthma | 11 | 2.52325E-05 | Serotonergic synapse | 4 | 6.6824E-05 | Pertussis | 24 | 1.32399E-17 |
| Arrhythmogenic right ventricular cardiomyopathy (ARVC) | 18 | 5.0605E-05 | Nicotine addiction | 3 | 6.7278E-05 | Small cell lung cancer | 25 | 3.68297E-17 |
| Thyroid cancer | 10 | 0.00010814 | Neuroactive ligand-receptor interaction | 5 | 0.000187308 | Osteoclast differentiation | 29 | 3.34278E-16 |
| Leishmaniasis | 17 | 0.000121014 | Alzheimer's disease | 4 | 0.000320019 | p53 signaling pathway | 22 | 3.36844E-16 |
| Salmonella infection | 19 | 0.000128089 | Insulin secretion | 3 | 0.000634317 | HIF-1 signaling pathway | 26 | 3.91076E-16 |
| Bladder cancer | 12 | 0.000137338 | Morphine addiction | 3 | 0.00077413 | Adipocytokine signaling pathway | 22 | 4.76234E-16 |
| TNF signaling pathway | 22 | 0.000184712 | Dopaminergic synapse | 3 | 0.002119725 | EGFR tyrosine kinase inhibitor resistance | 23 | 1.23904E-15 |
| Pathways in cancer | 56 | 0.000222826 | Type II diabetes mellitus | 2 | 0.004160808 | MAPK signaling pathway | 39 | 1.42072E-15 |
| Colorectal cancer | 15 | 0.0002248 | Colorectal cancer | 2 | 0.006857471 | Insulin resistance | 26 | 1.74292E-15 |
| Inflammatory mediator regulation of TRP channels | 20 | 0.000269598 | Pathways in cancer | 4 | 0.007531107 | Transcriptional misregulation in cancer | 32 | 6.35031E-15 |
| Apoptosis | 25 | 0.000441849 | Long-term potentiation | 2 | 0.007740842 | Toll-like receptor signaling pathway | 25 | 8.21737E-15 |
| EGFR tyrosine kinase inhibitor resistance | 17 | 0.000541134 | Pertussis | 2 | 0.009905481 | Chronic myeloid leukemia | 21 | 1.66103E-14 |
| Jak-STAT signaling pathway | 27 | 0.000547748 | Taste transduction | 2 | 0.012029758 | Prolactin signaling pathway | 20 | 1.48934E-13 |
| Chagas disease (American trypanosomiasis) | 20 | 0.000608358 | Glycerophospholipid metabolism | 2 | 0.015555913 | Malaria | 17 | 1.72101E-13 |
| Prion diseases | 10 | 0.000610391 | Estrogen signaling pathway | 2 | 0.016818987 | Focal adhesion | 32 | 2.46058E-13 |
| Type II diabetes mellitus | 12 | 0.000683308 | AGE-RAGE signaling pathway in diabetic complications | 2 | 0.017466564 | NF-kappa B signaling pathway | 22 | 2.5905E-13 |
| Tuberculosis | 29 | 0.000707661 | Glutamatergic synapse | 2 | 0.021929716 | VEGF signaling pathway | 18 | 7.98539E-13 |
| Type I diabetes mellitus | 11 | 0.000921765 | Neurotrophin signaling pathway | 2 | 0.024133781 | Apoptosis - multiple species | 14 | 9.4501E-13 |
| Allograft rejection | 10 | 0.000985952 | Osteoclast differentiation | 2 | 0.028401194 | AMPK signaling pathway | 24 | 2.43563E-12 |
| Fc epsilon RI signaling pathway | 14 | 0.001704125 | Apoptosis | 2 | 0.032102876 | Glioma | 18 | 2.67739E-12 |
| Intestinal immune network for IgA production | 11 | 0.002019637 | Hepatitis B | 2 | 0.034671843 | Cell cycle | 24 | 2.92799E-12 |
| VEGF signaling pathway | 13 | 0.002043245 | Renin-angiotensin system | 1 | 0.045575197 | T cell receptor signaling pathway | 22 | 4.56827E-12 |
| Renin-angiotensin system | 7 | 0.002713461 | alpha-Linolenic acid metabolism | 1 | 0.049443363 | Influenza A | 28 | 5.66652E-12 |
| Small cell lung cancer | 16 | 0.002939888 | Phototransduction | 1 | 0.053296595 | Rheumatoid arthritis | 20 | 1.11663E-11 |
| Complement and coagulation cascades | 15 | 0.0032001 | Linoleic acid metabolism | 1 | 0.057134948 | Melanoma | 18 | 1.38662E-11 |
| Longevity regulating pathway - multiple species | 13 | 0.003200567 | Thyroid cancer | 1 | 0.057134948 | MicroRNAs in cancer | 36 | 2.75973E-11 |
| Glioma | 13 | 0.003687782 | Apoptosis - multiple species | 1 | 0.064767237 | Neurotrophin signaling pathway | 22 | 7.4259E-11 |
| Hepatitis B | 23 | 0.004026323 | Ras signaling pathway | 2 | 0.076809469 | Measles | 23 | 1.28281E-10 |
| MicroRNAs in cancer | 40 | 0.004439378 | Fat digestion and absorption | 1 | 0.079855658 | Non-alcoholic fatty liver disease (NAFLD) | 24 | 2.24897E-10 |
| Mineral absorption | 11 | 0.004681971 | Ether lipid metabolism | 1 | 0.087312651 | Viral carcinogenesis | 28 | 2.66977E-10 |
| MAPK signaling pathway | 35 | 0.005003925 | Carbohydrate digestion and absorption | 1 | 0.089167886 | Non-small cell lung cancer | 15 | 3.09519E-10 |
| Platinum drug resistance | 14 | 0.005071485 | Cocaine addiction | 1 | 0.094712066 | Thyroid hormone signaling pathway | 21 | 3.612E-10 |
| Pertussis | 14 | 0.005071485 | Arachidonic acid metabolism | 1 | 0.11836761 | NOD-like receptor signaling pathway | 15 | 4.07275E-10 |
| Long-term depression | 12 | 0.005178783 | Glioma | 1 | 0.123742362 | Salmonella infection | 18 | 4.27577E-10 |
| NF-kappa B signaling pathway | 16 | 0.005850091 | Inflammatory bowel disease (IBD) | 1 | 0.123742362 | Ovarian steroidogenesis | 14 | 6.40441E-10 |
| Thyroid hormone signaling pathway | 19 | 0.006701324 | Renal cell carcinoma | 1 | 0.127308178 | Amoebiasis | 19 | 6.67229E-10 |
| Calcium signaling pathway | 26 | 0.007010522 | Epithelial cell signaling in Helicobacter pylori infection | 1 | 0.129085899 | Epstein-Barr virus infection | 27 | 7.35808E-10 |
| Melanoma | 13 | 0.008015095 | PPAR signaling pathway | 1 | 0.136162356 | Amyotrophic lateral sclerosis (ALS) | 14 | 8.55953E-10 |
| Sphingolipid signaling pathway | 19 | 0.008028067 | B cell receptor signaling pathway | 1 | 0.136162356 | Rap1 signaling pathway | 27 | 2.45491E-09 |
| Prostate cancer | 15 | 0.009984343 | Leishmaniasis | 1 | 0.136162356 | Legionellosis | 14 | 2.54807E-09 |
| Chronic myeloid leukemia | 13 | 0.010121119 | Gastric acid secretion | 1 | 0.139680016 | Ras signaling pathway | 28 | 3.15154E-09 |
| Protein digestion and absorption | 15 | 0.011042152 | Platinum drug resistance | 1 | 0.141433725 | Hepatitis C | 21 | 3.50706E-09 |
| Transcriptional misregulation in cancer | 25 | 0.013052743 | Salmonella infection | 1 | 0.160501186 | Fc epsilon RI signaling pathway | 15 | 4.68717E-09 |
| Endometrial cancer | 10 | 0.013526395 | ErbB signaling pathway | 1 | 0.163924381 | Central carbon metabolism in cancer | 15 | 4.68717E-09 |
| Leukocyte transendothelial migration | 18 | 0.014059123 | Salivary secretion | 1 | 0.16563099 | Estrogen signaling pathway | 18 | 4.70865E-09 |
| Pantothenate and CoA biosynthesis | 5 | 0.016652958 | Rheumatoid arthritis | 1 | 0.16563099 | African trypanosomiasis | 11 | 8.32729E-09 |
| Aldosterone-regulated sodium reabsorption | 8 | 0.017928148 | Longevity regulating pathway | 1 | 0.174114385 | Endometrial cancer | 13 | 1.21241E-08 |
| Alzheimer's disease | 23 | 0.02084693 | Pancreatic secretion | 1 | 0.177484688 | cAMP signaling pathway | 25 | 1.42762E-08 |
| Endocrine resistance | 15 | 0.021106992 | Endocrine resistance | 1 | 0.179164923 | Jak-STAT signaling pathway | 22 | 1.62936E-08 |
| Non-small cell lung cancer | 10 | 0.022199971 | Phosphatidylinositol signaling system | 1 | 0.18084189 | Sphingolipid signaling pathway | 19 | 1.89294E-08 |
| FoxO signaling pathway | 19 | 0.022624099 | Inflammatory mediator regulation of TRP channels | 1 | 0.18084189 | Platelet activation | 19 | 2.49979E-08 |
| Graft-versus-host disease | 8 | 0.02385931 | Melanogenesis | 1 | 0.184186038 | Inflammatory bowel disease (IBD) | 14 | 2.62277E-08 |
| NOD-like receptor signaling pathway | 10 | 0.024885484 | Glucagon signaling pathway | 1 | 0.185853233 | Progesterone-mediated oocyte maturation | 17 | 2.65181E-08 |
| Measles | 19 | 0.026029738 | Choline metabolism in cancer | 1 | 0.185853233 | ErbB signaling pathway | 16 | 3.42976E-08 |
| Pancreatic cancer | 11 | 0.027237835 | Chagas disease (American trypanosomiasis) | 1 | 0.190835371 | Acute myeloid leukemia | 13 | 4.00851E-08 |
| Ovarian steroidogenesis | 9 | 0.027894693 | T cell receptor signaling pathway | 1 | 0.192489621 | Type II diabetes mellitus | 12 | 4.46433E-08 |
| Renal cell carcinoma | 11 | 0.030108004 | Toll-like receptor signaling pathway | 1 | 0.19414065 | Epithelial cell signaling in Helicobacter pylori infection | 14 | 4.83138E-08 |
| Central carbon metabolism in cancer | 11 | 0.030108004 | TNF signaling pathway | 1 | 0.200712672 | Steroid hormone biosynthesis | 13 | 5.01017E-08 |
| TGF-beta signaling pathway | 13 | 0.030279041 | Oocyte meiosis | 1 | 0.221721031 | GnRH signaling pathway | 16 | 5.62384E-08 |
| Dorso-ventral axis formation | 6 | 0.031471416 | AMPK signaling pathway | 1 | 0.221721031 | Longevity regulating pathway | 16 | 9.0313E-08 |
| Arginine biosynthesis | 5 | 0.03179242 | Insulin signaling pathway | 1 | 0.24685597 | B cell receptor signaling pathway | 14 | 1.03573E-07 |
| Influenza A | 23 | 0.031874261 | Wnt signaling pathway | 1 | 0.251481879 | Choline metabolism in cancer | 16 | 2.53478E-07 |
| Longevity regulating pathway | 14 | 0.033787643 | Phospholipase D signaling pathway | 1 | 0.253017825 | Herpes simplex infection | 22 | 2.86135E-07 |
| Serotonergic synapse | 16 | 0.034465033 | Non-alcoholic fatty liver disease (NAFLD) | 1 | 0.265197797 | Thyroid cancer | 9 | 2.95129E-07 |
| Regulation of actin cytoskeleton | 27 | 0.035749474 | Hippo signaling pathway | 1 | 0.268213104 | Oxytocin signaling pathway | 20 | 3.28291E-07 |
| Estrogen signaling pathway | 14 | 0.049323649 | Influenza A | 1 | 0.300613114 | Drug metabolism - cytochrome P450 | 13 | 4.37751E-07 |
| cGMP-PKG signaling pathway | 21 | 0.058052327 | Tuberculosis | 1 | 0.303489837 | Chemical carcinogenesis | 14 | 5.60141E-07 |
| Neurotrophin signaling pathway | 16 | 0.058879214 | Alcoholism | 1 | 0.306355288 | Metabolism of xenobiotics by cytochrome P450 | 13 | 8.64234E-07 |
| Caffeine metabolism | 2 | 0.064122164 | Transcriptional misregulation in cancer | 1 | 0.306355288 | Insulin signaling pathway | 18 | 1.00607E-06 |
| Osteoclast differentiation | 17 | 0.064899739 | Herpes simplex infection | 1 | 0.314884434 | Shigellosis | 12 | 1.53435E-06 |
| Phagosome | 19 | 0.075984301 | Huntington's disease | 1 | 0.326101359 | Amphetamine addiction | 12 | 2.14907E-06 |
| p53 signaling pathway | 10 | 0.076830396 | Epstein-Barr virus infection | 1 | 0.335772494 | Renal cell carcinoma | 12 | 2.14907E-06 |
| Primary immunodeficiency | 6 | 0.090320592 | Focal adhesion | 1 | 0.339876673 | Leukocyte transendothelial migration | 16 | 2.18333E-06 |
| Adrenergic signaling in cardiomyocytes | 18 | 0.096034682 | Viral carcinogenesis | 1 | 0.342599363 | PPAR signaling pathway | 12 | 4.72603E-06 |
| Ras signaling pathway | 26 | 0.098400607 | Rap1 signaling pathway | 1 | 0.350703409 | Galactose metabolism | 8 | 6.44895E-06 |
| Fc gamma R-mediated phagocytosis | 12 | 0.105294742 | HTLV-I infection | 1 | 0.410973921 | Longevity regulating pathway - multiple species | 11 | 8.68201E-06 |
| Rap1 signaling pathway | 24 | 0.111158672 | Olfactory transduction | 1 | 0.576893918 | Oocyte meiosis | 15 | 1.69076E-05 |
| Endocrine and other factor-regulated calcium reabsorption | 7 | 0.113106109 | Metabolic pathways | 1 | 0.931126206 | Prion diseases | 8 | 1.70851E-05 |
| Regulation of lipolysis in adipocytes | 8 | 0.113359771 | Glycolysis / Gluconeogenesis | 0 | 1 | Cholinergic synapse | 14 | 2.1972E-05 |
| Lysosome | 15 | 0.118633492 | Citrate cycle (TCA cycle) | 0 | 1 | TGF-beta signaling pathway | 12 | 2.41068E-05 |
| Acute myeloid leukemia | 8 | 0.122354971 | Pentose phosphate pathway | 0 | 1 | Inflammatory mediator regulation of TRP channels | 13 | 2.5033E-05 |
| T cell receptor signaling pathway | 13 | 0.127950963 | Pentose and glucuronate interconversions | 0 | 1 | Hematopoietic cell lineage | 12 | 3.45365E-05 |
| ErbB signaling pathway | 11 | 0.145620208 | Fructose and mannose metabolism | 0 | 1 | Glucagon signaling pathway | 13 | 3.46848E-05 |
| Insulin resistance | 13 | 0.156807295 | Galactose metabolism | 0 | 1 | Adherens junction | 11 | 3.60319E-05 |
| Porphyrin and chlorophyll metabolism | 6 | 0.157732664 | Ascorbate and aldarate metabolism | 0 | 1 | Type I diabetes mellitus | 8 | 8.30461E-05 |
| Apoptosis - multiple species | 5 | 0.157951526 | Fatty acid biosynthesis | 0 | 1 | Signaling pathways regulating pluripotency of stem cells | 15 | 9.2008E-05 |
| Autoimmune thyroid disease | 7 | 0.166734599 | Fatty acid elongation | 0 | 1 | Cytokine-cytokine receptor interaction | 22 | 9.71459E-05 |
| Choline metabolism in cancer | 12 | 0.172046451 | Fatty acid degradation | 0 | 1 | Serotonergic synapse | 13 | 0.0001032 |
| PPAR signaling pathway | 9 | 0.176373508 | Synthesis and degradation of ketone bodies | 0 | 1 | RIG-I-like receptor signaling pathway | 10 | 0.000114966 |
| Phospholipase D signaling pathway | 16 | 0.185330291 | Steroid biosynthesis | 0 | 1 | Dopaminergic synapse | 14 | 0.000117449 |
| Natural killer cell mediated cytotoxicity | 15 | 0.19441708 | Primary bile acid biosynthesis | 0 | 1 | Viral myocarditis | 9 | 0.000131596 |
| Biosynthesis of amino acids | 9 | 0.208034288 | Ubiquinone and other terpenoid-quinone biosynthesis | 0 | 1 | Chemokine signaling pathway | 17 | 0.000206099 |
| Oxytocin signaling pathway | 17 | 0.212885249 | Steroid hormone biosynthesis | 0 | 1 | Cocaine addiction | 8 | 0.000216303 |
| Long-term potentiation | 8 | 0.218397972 | Oxidative phosphorylation | 0 | 1 | Arachidonic acid metabolism | 9 | 0.000222508 |
| Glyoxylate and dicarboxylate metabolism | 4 | 0.226624797 | Arginine biosynthesis | 0 | 1 | Aldosterone-regulated sodium reabsorption | 7 | 0.000293411 |
| Antigen processing and presentation | 9 | 0.230327702 | Purine metabolism | 0 | 1 | Caffeine metabolism | 3 | 0.000367937 |
| Cardiac muscle contraction | 9 | 0.241792098 | Caffeine metabolism | 0 | 1 | Regulation of actin cytoskeleton | 18 | 0.000374956 |
| Non-alcoholic fatty liver disease (NAFLD) | 16 | 0.248032415 | Pyrimidine metabolism | 0 | 1 | Graft-versus-host disease | 7 | 0.000404606 |
| Taurine and hypotaurine metabolism | 2 | 0.249958096 | Alanine, aspartate and glutamate metabolism | 0 | 1 | Hypertrophic cardiomyopathy (HCM) | 10 | 0.000477162 |
| Cocaine addiction | 6 | 0.256164277 | Glycine, serine and threonine metabolism | 0 | 1 | cGMP-PKG signaling pathway | 15 | 0.000547986 |
| Cell adhesion molecules (CAMs) | 15 | 0.269776954 | Cysteine and methionine metabolism | 0 | 1 | Alzheimer's disease | 15 | 0.000583575 |
| Platelet activation | 13 | 0.270201018 | Valine, leucine and isoleucine degradation | 0 | 1 | Natural killer cell mediated cytotoxicity | 13 | 0.000614513 |
| AMPK signaling pathway | 13 | 0.279929713 | Valine, leucine and isoleucine biosynthesis | 0 | 1 | mTOR signaling pathway | 14 | 0.00069216 |
| Other types of O-glycan biosynthesis | 4 | 0.28614697 | Lysine biosynthesis | 0 | 1 | Gap junction | 10 | 0.000762358 |
| Riboflavin metabolism | 1 | 0.307044195 | Lysine degradation | 0 | 1 | Intestinal immune network for IgA production | 7 | 0.000950733 |
| Circadian entrainment | 10 | 0.319477118 | Arginine and proline metabolism | 0 | 1 | Phospholipase D signaling pathway | 13 | 0.001131754 |
| Gastric acid secretion | 8 | 0.320752942 | Histidine metabolism | 0 | 1 | Renin secretion | 8 | 0.001365201 |
| Toll-like receptor signaling pathway | 11 | 0.323750567 | Tyrosine metabolism | 0 | 1 | Allograft rejection | 6 | 0.001393796 |
| HTLV-I infection | 25 | 0.32746869 | Phenylalanine metabolism | 0 | 1 | Glutathione metabolism | 7 | 0.001749619 |
| Renin secretion | 7 | 0.327528872 | Tryptophan metabolism | 0 | 1 | Tryptophan metabolism | 6 | 0.002112915 |
| Huntington's disease | 19 | 0.330952911 | Phenylalanine, tyrosine and tryptophan biosynthesis | 0 | 1 | Bile secretion | 8 | 0.002673119 |
| Staphylococcus aureus infection | 6 | 0.350448965 | beta-Alanine metabolism | 0 | 1 | Regulation of lipolysis in adipocytes | 7 | 0.002701778 |
| Phosphatidylinositol signaling system | 10 | 0.355182847 | Taurine and hypotaurine metabolism | 0 | 1 | Starch and sucrose metabolism | 7 | 0.002992379 |
| Cysteine and methionine metabolism | 5 | 0.359153595 | Selenocompound metabolism | 0 | 1 | Calcium signaling pathway | 14 | 0.003094297 |
| Butirosin and neomycin biosynthesis | 1 | 0.367774703 | D-Glutamine and D-glutamate metabolism | 0 | 1 | Wnt signaling pathway | 12 | 0.003203103 |
| Salivary secretion | 9 | 0.377522418 | D-Arginine and D-ornithine metabolism | 0 | 1 | Cell adhesion molecules (CAMs) | 12 | 0.003392845 |
| Cholinergic synapse | 11 | 0.380549516 | Glutathione metabolism | 0 | 1 | Long-term depression | 7 | 0.004008914 |
| Epstein-Barr virus infection | 19 | 0.390340995 | Starch and sucrose metabolism | 0 | 1 | Carbohydrate digestion and absorption | 6 | 0.004349005 |
| Adipocytokine signaling pathway | 7 | 0.415868106 | N-Glycan biosynthesis | 0 | 1 | Aldosterone synthesis and secretion | 8 | 0.006036143 |
| Inositol phosphate metabolism | 7 | 0.430610483 | Other glycan degradation | 0 | 1 | Huntington's disease | 14 | 0.00603847 |
| Bile secretion | 7 | 0.430610483 | Mucin type O-Glycan biosynthesis | 0 | 1 | Tyrosine metabolism | 5 | 0.006134868 |
| Tight junction | 13 | 0.445237804 | Other types of O-glycan biosynthesis | 0 | 1 | Melanogenesis | 9 | 0.006774717 |
| Prolactin signaling pathway | 7 | 0.445304175 | Amino sugar and nucleotide sugar metabolism | 0 | 1 | Long-term potentiation | 7 | 0.006802642 |
| Primary bile acid biosynthesis | 2 | 0.446209876 | Butirosin and neomycin biosynthesis | 0 | 1 | Vascular smooth muscle contraction | 10 | 0.00756237 |
| Nitrogen metabolism | 2 | 0.446209876 | Glycosaminoglycan degradation | 0 | 1 | Alcoholism | 13 | 0.007926377 |
| Herpes simplex infection | 17 | 0.455581447 | Glycosaminoglycan biosynthesis - chondroitin sulfate / dermatan sulfate | 0 | 1 | Autoimmune thyroid disease | 6 | 0.007978522 |
| Dopaminergic synapse | 12 | 0.458402057 | Glycosaminoglycan biosynthesis - keratan sulfate | 0 | 1 | Dilated cardiomyopathy | 8 | 0.010505311 |
| Glycine, serine and threonine metabolism | 4 | 0.46942248 | Glycosaminoglycan biosynthesis - heparan sulfate / heparin | 0 | 1 | Fat digestion and absorption | 5 | 0.012006928 |
| Pentose phosphate pathway | 3 | 0.473004987 | Glycerolipid metabolism | 0 | 1 | Fc gamma R-mediated phagocytosis | 8 | 0.012692998 |
| Adherens junction | 7 | 0.47447139 | Inositol phosphate metabolism | 0 | 1 | Dorso-ventral axis formation | 4 | 0.014002865 |
| 2-Oxocarboxylic acid metabolism | 2 | 0.476330011 | Glycosylphosphatidylinositol(GPI)-anchor biosynthesis | 0 | 1 | Hippo signaling pathway | 11 | 0.015011277 |
| Protein processing in endoplasmic reticulum | 15 | 0.480168078 | Sphingolipid metabolism | 0 | 1 | Linoleic acid metabolism | 4 | 0.015819407 |
| Citrate cycle (TCA cycle) | 3 | 0.49600488 | Glycosphingolipid biosynthesis - lacto and neolacto series | 0 | 1 | Bacterial invasion of epithelial cells | 7 | 0.016371482 |
| Gap junction | 8 | 0.510876259 | Glycosphingolipid biosynthesis - globo series | 0 | 1 | Asthma | 4 | 0.017777404 |
| Galactose metabolism | 3 | 0.518489657 | Glycosphingolipid biosynthesis - ganglio series | 0 | 1 | Drug metabolism - other enzymes | 5 | 0.019153594 |
| Retrograde endocannabinoid signaling | 9 | 0.529934736 | Pyruvate metabolism | 0 | 1 | Cytosolic DNA-sensing pathway | 6 | 0.019553427 |
| Bacterial invasion of epithelial cells | 7 | 0.531452355 | Glyoxylate and dicarboxylate metabolism | 0 | 1 | ECM-receptor interaction | 7 | 0.021023837 |
| One carbon pool by folate | 2 | 0.53345531 | Propanoate metabolism | 0 | 1 | Retrograde endocannabinoid signaling | 8 | 0.021251362 |
| Amphetamine addiction | 6 | 0.540153119 | Butanoate metabolism | 0 | 1 | Peroxisome | 7 | 0.022317006 |
| ABC transporters | 4 | 0.545926108 | One carbon pool by folate | 0 | 1 | Arginine and proline metabolism | 5 | 0.02455275 |
| Glutamatergic synapse | 10 | 0.546809663 | Thiamine metabolism | 0 | 1 | Insulin secretion | 7 | 0.025066432 |
| Insulin signaling pathway | 12 | 0.56550563 | Riboflavin metabolism | 0 | 1 | Pathogenic Escherichia coli infection | 5 | 0.038062753 |
| Aldosterone synthesis and secretion | 7 | 0.572540374 | Vitamin B6 metabolism | 0 | 1 | Staphylococcus aureus infection | 5 | 0.038062753 |
| Hedgehog signaling pathway | 4 | 0.58196083 | Nicotinate and nicotinamide metabolism | 0 | 1 | Vitamin digestion and absorption | 3 | 0.046251198 |
| Parkinson's disease | 12 | 0.59605841 | Pantothenate and CoA biosynthesis | 0 | 1 | Taurine and hypotaurine metabolism | 2 | 0.051558118 |
| Peroxisome | 7 | 0.598963927 | Biotin metabolism | 0 | 1 | Endocytosis | 14 | 0.059427381 |
| Sphingolipid metabolism | 4 | 0.599347156 | Lipoic acid metabolism | 0 | 1 | Metabolic pathways | 52 | 0.060438924 |
| Sulfur metabolism | 1 | 0.600383718 | Folate biosynthesis | 0 | 1 | Adrenergic signaling in cardiomyocytes | 9 | 0.064676228 |
| Alanine, aspartate and glutamate metabolism | 3 | 0.602590605 | Retinol metabolism | 0 | 1 | Retinol metabolism | 5 | 0.069060615 |
| Tyrosine metabolism | 3 | 0.602590605 | Porphyrin and chlorophyll metabolism | 0 | 1 | Phagosome | 9 | 0.07620262 |
| Pancreatic secretion | 8 | 0.61208953 | Terpenoid backbone biosynthesis | 0 | 1 | Neuroactive ligand-receptor interaction | 14 | 0.089064401 |
| B cell receptor signaling pathway | 6 | 0.612202508 | Nitrogen metabolism | 0 | 1 | Mineral absorption | 4 | 0.099129432 |
| Amino sugar and nucleotide sugar metabolism | 4 | 0.616291704 | Sulfur metabolism | 0 | 1 | Tight junction | 8 | 0.100318363 |
| Insulin secretion | 7 | 0.624521183 | Aminoacyl-tRNA biosynthesis | 0 | 1 | Circadian entrainment | 6 | 0.103508732 |
| Arginine and proline metabolism | 4 | 0.632782122 | Metabolism of xenobiotics by cytochrome P450 | 0 | 1 | Arrhythmogenic right ventricular cardiomyopathy (ARVC) | 5 | 0.106003421 |
| N-Glycan biosynthesis | 4 | 0.632782122 | Drug metabolism - cytochrome P450 | 0 | 1 | Phenylalanine metabolism | 2 | 0.111732054 |
| Vitamin digestion and absorption | 2 | 0.63432105 | Drug metabolism - other enzymes | 0 | 1 | Pentose and glucuronate interconversions | 3 | 0.121840922 |
| Systemic lupus erythematosus | 11 | 0.634854571 | Biosynthesis of unsaturated fatty acids | 0 | 1 | Other glycan degradation | 2 | 0.122994224 |
| Ubiquinone and other terpenoid-quinone biosynthesis | 1 | 0.63542628 | Carbon metabolism | 0 | 1 | Riboflavin metabolism | 1 | 0.128965095 |
| Arachidonic acid metabolism | 5 | 0.642847387 | 2-Oxocarboxylic acid metabolism | 0 | 1 | Complement and coagulation cascades | 5 | 0.129979422 |
| alpha-Linolenic acid metabolism | 2 | 0.656705566 | Fatty acid metabolism | 0 | 1 | Glycosaminoglycan degradation | 2 | 0.134515937 |
| Synaptic vesicle cycle | 5 | 0.657002119 | Biosynthesis of amino acids | 0 | 1 | Axon guidance | 9 | 0.144336463 |
| Carbon metabolism | 9 | 0.666998013 | EGFR tyrosine kinase inhibitor resistance | 0 | 1 | Nicotine addiction | 3 | 0.153207925 |
| Shigellosis | 5 | 0.684210981 | ABC transporters | 0 | 1 | Arginine biosynthesis | 2 | 0.158217993 |
| Tryptophan metabolism | 3 | 0.693176336 | Ribosome biogenesis in eukaryotes | 0 | 1 | Butirosin and neomycin biosynthesis | 1 | 0.158525863 |
| Regulation of autophagy | 3 | 0.693176336 | Ribosome | 0 | 1 | Porphyrin and chlorophyll metabolism | 3 | 0.169752597 |
| Nicotine addiction | 3 | 0.693176336 | RNA transport | 0 | 1 | Mismatch repair | 2 | 0.182615287 |
| GnRH signaling pathway | 7 | 0.695430988 | mRNA surveillance pathway | 0 | 1 | Renin-angiotensin system | 2 | 0.182615287 |
| Non-homologous end-joining | 1 | 0.696570609 | RNA degradation | 0 | 1 | ABC transporters | 3 | 0.186782212 |
| Collecting duct acid secretion | 2 | 0.698165746 | RNA polymerase | 0 | 1 | Glycolysis / Gluconeogenesis | 4 | 0.192096732 |
| Fat digestion and absorption | 3 | 0.709292711 | Basal transcription factors | 0 | 1 | Histidine metabolism | 2 | 0.195012022 |
| Butanoate metabolism | 2 | 0.717291085 | DNA replication | 0 | 1 | Parkinson's disease | 7 | 0.207520759 |
| Vascular smooth muscle contraction | 9 | 0.734945141 | Spliceosome | 0 | 1 | Sphingolipid metabolism | 3 | 0.213093173 |
| Linoleic acid metabolism | 2 | 0.735386351 | Proteasome | 0 | 1 | Maturity onset diabetes of the young | 2 | 0.220089306 |
| Nicotinate and nicotinamide metabolism | 2 | 0.735386351 | Protein export | 0 | 1 | Pancreatic secretion | 5 | 0.22654627 |
| Glycerophospholipid metabolism | 7 | 0.73755483 | Base excision repair | 0 | 1 | Glyoxylate and dicarboxylate metabolism | 2 | 0.245410903 |
| Taste transduction | 6 | 0.745906066 | Nucleotide excision repair | 0 | 1 | Circadian rhythm | 2 | 0.270833119 |
| Glycosaminoglycan biosynthesis - keratan sulfate | 1 | 0.747469514 | Mismatch repair | 0 | 1 | Basal cell carcinoma | 3 | 0.286286949 |
| Glycosphingolipid biosynthesis - ganglio series | 1 | 0.747469514 | Homologous recombination | 0 | 1 | Fructose and mannose metabolism | 2 | 0.308884593 |
| Starch and sucrose metabolism | 4 | 0.747644681 | Non-homologous end-joining | 0 | 1 | Base excision repair | 2 | 0.308884593 |
| Circadian rhythm | 2 | 0.752485028 | Fanconi anemia pathway | 0 | 1 | Glycerolipid metabolism | 3 | 0.323695226 |
| Vasopressin-regulated water reabsorption | 3 | 0.75372599 | Cytokine-cytokine receptor interaction | 0 | 1 | Protein processing in endoplasmic reticulum | 7 | 0.327656955 |
| Progesterone-mediated oocyte maturation | 7 | 0.766362528 | Chemokine signaling pathway | 0 | 1 | Glutamatergic synapse | 5 | 0.344619272 |
| Ether lipid metabolism | 3 | 0.767267288 | NF-kappa B signaling pathway | 0 | 1 | GABAergic synapse | 4 | 0.349358109 |
| beta-Alanine metabolism | 2 | 0.76862264 | HIF-1 signaling pathway | 0 | 1 | Salivary secretion | 4 | 0.357123835 |
| Axon guidance | 13 | 0.779709231 | FoxO signaling pathway | 0 | 1 | Fatty acid biosynthesis | 1 | 0.361693163 |
| Carbohydrate digestion and absorption | 3 | 0.780195646 | Sphingolipid signaling pathway | 0 | 1 | Folate biosynthesis | 1 | 0.38337453 |
| Selenocompound metabolism | 1 | 0.78983816 | Cell cycle | 0 | 1 | Glycine, serine and threonine metabolism | 2 | 0.395589242 |
| GABAergic synapse | 6 | 0.794393133 | p53 signaling pathway | 0 | 1 | Glycerophospholipid metabolism | 4 | 0.403594059 |
| mTOR signaling pathway | 11 | 0.795435024 | Ubiquitin mediated proteolysis | 0 | 1 | Glycosaminoglycan biosynthesis - keratan sulfate | 1 | 0.404321467 |
| Hippo signaling pathway | 11 | 0.795435024 | Sulfur relay system | 0 | 1 | Glycosphingolipid biosynthesis - ganglio series | 1 | 0.404321467 |
| Fructose and mannose metabolism | 2 | 0.798163393 | SNARE interactions in vesicular transport | 0 | 1 | Vasopressin-regulated water reabsorption | 2 | 0.442901969 |
| Notch signaling pathway | 3 | 0.804275193 | Regulation of autophagy | 0 | 1 | Gastric acid secretion | 3 | 0.461549391 |
| Signaling pathways regulating pluripotency of stem cells | 10 | 0.80797578 | Protein processing in endoplasmic reticulum | 0 | 1 | Pantothenate and CoA biosynthesis | 1 | 0.462999645 |
| SNARE interactions in vesicular transport | 2 | 0.811642722 | Lysosome | 0 | 1 | Hedgehog signaling pathway | 2 | 0.465782777 |
| Cytosolic DNA-sensing pathway | 4 | 0.814141515 | Endocytosis | 0 | 1 | Endocrine and other factor-regulated calcium reabsorption | 2 | 0.477013426 |
| Morphine addiction | 6 | 0.819884536 | Phagosome | 0 | 1 | Systemic lupus erythematosus | 5 | 0.478996349 |
| Endocytosis | 19 | 0.828536199 | Peroxisome | 0 | 1 | Fatty acid metabolism | 2 | 0.488099395 |
| Hepatitis C | 9 | 0.834652982 | mTOR signaling pathway | 0 | 1 | Steroid biosynthesis | 1 | 0.498879371 |
| Glycosaminoglycan biosynthesis - chondroitin sulfate / dermatan sulfate | 1 | 0.84045482 | PI3K-Akt signaling pathway | 0 | 1 | Terpenoid backbone biosynthesis | 1 | 0.532368145 |
| cAMP signaling pathway | 14 | 0.840670146 | Longevity regulating pathway - multiple species | 0 | 1 | Carbon metabolism | 4 | 0.537166999 |
| Chemokine signaling pathway | 13 | 0.845228253 | Dorso-ventral axis formation | 0 | 1 | Proximal tubule bicarbonate reclamation | 1 | 0.548266002 |
| Lysine degradation | 3 | 0.845802832 | Notch signaling pathway | 0 | 1 | Glycosaminoglycan biosynthesis - heparan sulfate / heparin | 1 | 0.56362487 |
| Neuroactive ligand-receptor interaction | 20 | 0.848431498 | Hedgehog signaling pathway | 0 | 1 | alpha-Linolenic acid metabolism | 1 | 0.578462974 |
| Epithelial cell signaling in Helicobacter pylori infection | 4 | 0.857935608 | TGF-beta signaling pathway | 0 | 1 | Glycosphingolipid biosynthesis - lacto and neolacto series | 1 | 0.59279792 |
| Terpenoid backbone biosynthesis | 1 | 0.867235061 | Axon guidance | 0 | 1 | Morphine addiction | 3 | 0.601152304 |
| Viral carcinogenesis | 14 | 0.869346415 | VEGF signaling pathway | 0 | 1 | Ascorbate and aldarate metabolism | 1 | 0.606646725 |
| Protein export | 1 | 0.878890782 | Hippo signaling pathway -multiple species | 0 | 1 | Phototransduction | 1 | 0.606646725 |
| Mismatch repair | 1 | 0.878890782 | ECM-receptor interaction | 0 | 1 | Butanoate metabolism | 1 | 0.62002583 |
| Proximal tubule bicarbonate reclamation | 1 | 0.878890782 | Cell adhesion molecules (CAMs) | 0 | 1 | Pentose phosphate pathway | 1 | 0.632951121 |
| Melanogenesis | 6 | 0.881551774 | Adherens junction | 0 | 1 | Homologous recombination | 1 | 0.632951121 |
| Glucagon signaling pathway | 6 | 0.887157305 | Tight junction | 0 | 1 | Citrate cycle (TCA cycle) | 1 | 0.645437948 |
| Glycosylphosphatidylinositol(GPI)-anchor biosynthesis | 1 | 0.899225027 | Gap junction | 0 | 1 | Phosphatidylinositol signaling system | 3 | 0.651272919 |
| Glycerolipid metabolism | 3 | 0.900245913 | Signaling pathways regulating pluripotency of stem cells | 0 | 1 | beta-Alanine metabolism | 1 | 0.657501146 |
| Glycosphingolipid biosynthesis - lacto and neolacto series | 1 | 0.908074843 | Complement and coagulation cascades | 0 | 1 | Ubiquitin mediated proteolysis | 4 | 0.688302993 |
| Maturity onset diabetes of the young | 1 | 0.908074843 | Platelet activation | 0 | 1 | Inositol phosphate metabolism | 2 | 0.699725662 |
| Spliceosome | 8 | 0.910177186 | Antigen processing and presentation | 0 | 1 | Thyroid hormone synthesis | 2 | 0.699725662 |
| Basal transcription factors | 2 | 0.914525129 | NOD-like receptor signaling pathway | 0 | 1 | Alanine, aspartate and glutamate metabolism | 1 | 0.701797131 |
| Drug metabolism - other enzymes | 2 | 0.920631015 | RIG-I-like receptor signaling pathway | 0 | 1 | DNA replication | 1 | 0.711947736 |
| Cell cycle | 7 | 0.926216835 | Cytosolic DNA-sensing pathway | 0 | 1 | Primary immunodeficiency | 1 | 0.711947736 |
| Wnt signaling pathway | 8 | 0.937994645 | Jak-STAT signaling pathway | 0 | 1 | Antigen processing and presentation | 2 | 0.741412252 |
| Glycolysis / Gluconeogenesis | 3 | 0.940791427 | Hematopoietic cell lineage | 0 | 1 | Pyruvate metabolism | 1 | 0.749219139 |
| Vibrio cholerae infection | 2 | 0.94546236 | Natural killer cell mediated cytotoxicity | 0 | 1 | Regulation of autophagy | 1 | 0.749219139 |
| Glutathione metabolism | 2 | 0.949448741 | Fc epsilon RI signaling pathway | 0 | 1 | Fatty acid degradation | 1 | 0.781679868 |
| Base excision repair | 1 | 0.951706016 | Fc gamma R-mediated phagocytosis | 0 | 1 | Proteasome | 1 | 0.781679868 |
| Thyroid hormone synthesis | 3 | 0.954754349 | Leukocyte transendothelial migration | 0 | 1 | Cysteine and methionine metabolism | 1 | 0.789117797 |
| Pathogenic Escherichia coli infection | 2 | 0.95980615 | Intestinal immune network for IgA production | 0 | 1 | Ether lipid metabolism | 1 | 0.789117797 |
| Oocyte meiosis | 6 | 0.96426645 | Circadian rhythm | 0 | 1 | Lysosome | 3 | 0.792856287 |
| Steroid hormone biosynthesis | 2 | 0.968111311 | Synaptic vesicle cycle | 0 | 1 | Nucleotide excision repair | 1 | 0.803244098 |
| Pyruvate metabolism | 1 | 0.974639754 | Long-term depression | 0 | 1 | Amino sugar and nucleotide sugar metabolism | 1 | 0.809949305 |
| Chemical carcinogenesis | 3 | 0.978905756 | Regulation of actin cytoskeleton | 0 | 1 | Notch signaling pathway | 1 | 0.809949305 |
| Ribosome biogenesis in eukaryotes | 3 | 0.980349439 | Ovarian steroidogenesis | 0 | 1 | Protein digestion and absorption | 2 | 0.815005261 |
| Fatty acid degradation | 1 | 0.982452584 | Progesterone-mediated oocyte maturation | 0 | 1 | Vibrio cholerae infection | 1 | 0.828728286 |
| Proteasome | 1 | 0.982452584 | Prolactin signaling pathway | 0 | 1 | Fanconi anemia pathway | 1 | 0.845656457 |
| Aminoacyl-tRNA biosynthesis | 2 | 0.982959011 | Thyroid hormone synthesis | 0 | 1 | Aminoacyl-tRNA biosynthesis | 1 | 0.898242602 |
| RNA transport | 8 | 0.985927259 | Thyroid hormone signaling pathway | 0 | 1 | Biosynthesis of amino acids | 1 | 0.925572494 |
| Nucleotide excision repair | 1 | 0.986688856 | Adipocytokine signaling pathway | 0 | 1 | RNA degradation | 1 | 0.930572414 |
| RIG-I-like receptor signaling pathway | 2 | 0.987598573 | Regulation of lipolysis in adipocytes | 0 | 1 | Ribosome biogenesis in eukaryotes | 1 | 0.943650245 |
| Fatty acid metabolism | 1 | 0.987860298 | Insulin resistance | 0 | 1 | Taste transduction | 1 | 0.943650245 |
| Alcoholism | 8 | 0.990956725 | Type I diabetes mellitus | 0 | 1 | mRNA surveillance pathway | 1 | 0.957346469 |
| RNA degradation | 2 | 0.992932809 | Maturity onset diabetes of the young | 0 | 1 | Purine metabolism | 2 | 0.983098017 |
| Fanconi anemia pathway | 1 | 0.993016413 | Aldosterone-regulated sodium reabsorption | 0 | 1 | Oxidative phosphorylation | 1 | 0.99014944 |
| Basal cell carcinoma | 1 | 0.993631418 | Endocrine and other factor-regulated calcium reabsorption | 0 | 1 | Spliceosome | 1 | 0.990487954 |
| mRNA surveillance pathway | 2 | 0.997749815 | Vasopressin-regulated water reabsorption | 0 | 1 | RNA transport | 1 | 0.997398088 |
| Oxidative phosphorylation | 4 | 0.997902984 | Proximal tubule bicarbonate reclamation | 0 | 1 | Olfactory transduction | 1 | 0.999999569 |
| Ubiquitin mediated proteolysis | 4 | 0.998428377 | Collecting duct acid secretion | 0 | 1 | Fatty acid elongation | 0 | 1 |
| Metabolism of xenobiotics by cytochrome P450 | 1 | 0.99879023 | Protein digestion and absorption | 0 | 1 | Synthesis and degradation of ketone bodies | 0 | 1 |
| Metabolic pathways | 81 | 0.99907944 | Bile secretion | 0 | 1 | Primary bile acid biosynthesis | 0 | 1 |
| Purine metabolism | 5 | 0.999557652 | Vitamin digestion and absorption | 0 | 1 | Ubiquinone and other terpenoid-quinone biosynthesis | 0 | 1 |
| Olfactory transduction | 1 | 1 | Mineral absorption | 0 | 1 | Pyrimidine metabolism | 0 | 1 |
| Pentose and glucuronate interconversions | 0 | 1 | Parkinson's disease | 0 | 1 | Valine, leucine and isoleucine degradation | 0 | 1 |
| Ascorbate and aldarate metabolism | 0 | 1 | Amyotrophic lateral sclerosis (ALS) | 0 | 1 | Valine, leucine and isoleucine biosynthesis | 0 | 1 |
| Fatty acid biosynthesis | 0 | 1 | Prion diseases | 0 | 1 | Lysine biosynthesis | 0 | 1 |
| Fatty acid elongation | 0 | 1 | Bacterial invasion of epithelial cells | 0 | 1 | Lysine degradation | 0 | 1 |
| Synthesis and degradation of ketone bodies | 0 | 1 | Vibrio cholerae infection | 0 | 1 | Phenylalanine, tyrosine and tryptophan biosynthesis | 0 | 1 |
| Steroid biosynthesis | 0 | 1 | Pathogenic Escherichia coli infection | 0 | 1 | Selenocompound metabolism | 0 | 1 |
| Pyrimidine metabolism | 0 | 1 | Shigellosis | 0 | 1 | D-Glutamine and D-glutamate metabolism | 0 | 1 |
| Valine, leucine and isoleucine degradation | 0 | 1 | Legionellosis | 0 | 1 | D-Arginine and D-ornithine metabolism | 0 | 1 |
| Valine, leucine and isoleucine biosynthesis | 0 | 1 | African trypanosomiasis | 0 | 1 | N-Glycan biosynthesis | 0 | 1 |
| Lysine biosynthesis | 0 | 1 | Malaria | 0 | 1 | Mucin type O-Glycan biosynthesis | 0 | 1 |
| Histidine metabolism | 0 | 1 | Toxoplasmosis | 0 | 1 | Other types of O-glycan biosynthesis | 0 | 1 |
| Phenylalanine metabolism | 0 | 1 | Amoebiasis | 0 | 1 | Glycosaminoglycan biosynthesis - chondroitin sulfate / dermatan sulfate | 0 | 1 |
| Phenylalanine, tyrosine and tryptophan biosynthesis | 0 | 1 | Staphylococcus aureus infection | 0 | 1 | Glycosylphosphatidylinositol(GPI)-anchor biosynthesis | 0 | 1 |
| D-Glutamine and D-glutamate metabolism | 0 | 1 | Hepatitis C | 0 | 1 | Glycosphingolipid biosynthesis - globo series | 0 | 1 |
| D-Arginine and D-ornithine metabolism | 0 | 1 | Measles | 0 | 1 | Propanoate metabolism | 0 | 1 |
| Other glycan degradation | 0 | 1 | Chemical carcinogenesis | 0 | 1 | One carbon pool by folate | 0 | 1 |
| Mucin type O-Glycan biosynthesis | 0 | 1 | Proteoglycans in cancer | 0 | 1 | Thiamine metabolism | 0 | 1 |
| Glycosaminoglycan degradation | 0 | 1 | MicroRNAs in cancer | 0 | 1 | Vitamin B6 metabolism | 0 | 1 |
| Glycosaminoglycan biosynthesis - heparan sulfate / heparin | 0 | 1 | Pancreatic cancer | 0 | 1 | Nicotinate and nicotinamide metabolism | 0 | 1 |
| Glycosphingolipid biosynthesis - globo series | 0 | 1 | Endometrial cancer | 0 | 1 | Biotin metabolism | 0 | 1 |
| Propanoate metabolism | 0 | 1 | Prostate cancer | 0 | 1 | Lipoic acid metabolism | 0 | 1 |
| Thiamine metabolism | 0 | 1 | Basal cell carcinoma | 0 | 1 | Nitrogen metabolism | 0 | 1 |
| Vitamin B6 metabolism | 0 | 1 | Melanoma | 0 | 1 | Sulfur metabolism | 0 | 1 |
| Biotin metabolism | 0 | 1 | Bladder cancer | 0 | 1 | Biosynthesis of unsaturated fatty acids | 0 | 1 |
| Lipoic acid metabolism | 0 | 1 | Chronic myeloid leukemia | 0 | 1 | 2-Oxocarboxylic acid metabolism | 0 | 1 |
| Folate biosynthesis | 0 | 1 | Acute myeloid leukemia | 0 | 1 | Ribosome | 0 | 1 |
| Retinol metabolism | 0 | 1 | Small cell lung cancer | 0 | 1 | RNA polymerase | 0 | 1 |
| Drug metabolism - cytochrome P450 | 0 | 1 | Non-small cell lung cancer | 0 | 1 | Basal transcription factors | 0 | 1 |
| Biosynthesis of unsaturated fatty acids | 0 | 1 | Central carbon metabolism in cancer | 0 | 1 | Protein export | 0 | 1 |
| Ribosome | 0 | 1 | Asthma | 0 | 1 | Non-homologous end-joining | 0 | 1 |
| RNA polymerase | 0 | 1 | Autoimmune thyroid disease | 0 | 1 | Sulfur relay system | 0 | 1 |
| DNA replication | 0 | 1 | Systemic lupus erythematosus | 0 | 1 | SNARE interactions in vesicular transport | 0 | 1 |
| Homologous recombination | 0 | 1 | Allograft rejection | 0 | 1 | Cardiac muscle contraction | 0 | 1 |
| Sulfur relay system | 0 | 1 | Graft-versus-host disease | 0 | 1 | Hippo signaling pathway -multiple species | 0 | 1 |
| Hippo signaling pathway -multiple species | 0 | 1 | Primary immunodeficiency | 0 | 1 | Synaptic vesicle cycle | 0 | 1 |
| Phototransduction | 0 | 1 | Viral myocarditis | 0 | 1 | Collecting duct acid secretion | 0 | 1 |
